# The use of comparative genomic hybridization to characterize genome dynamics and diversity among the serotypes of *Shigella*

**DOI:** 10.1186/1471-2164-7-218

**Published:** 2006-08-29

**Authors:** Junping Peng, Xiaobing Zhang, Jian Yang, Jing Wang, E Yang, Wen Bin, Candong Wei, Meisheng Sun, Qi Jin

**Affiliations:** 1 State Key Laboratory for Molecular Virology and Genetic Engineering, Chinese Center for Disease Control and Prevention, Beijing 100176, China; 2 Institute of Pathogen Biology, Chinese Academy of Medical Sciences, Beijing 100730, China

## Abstract

**Background:**

Compelling evidence indicates that *Shigella *species, the etiologic agents of bacillary dysentery, as well as *enteroinvasive Escherichia coli*, are derived from multiple origins of *Escherichia coli *and form a single pathovar. To further understand the genome diversity and virulence evolution of *Shigella*, comparative genomic hybridization microarray analysis was employed to compare the gene content of *E. coli *K-12 with those of 43 *Shigella *strains from all lineages.

**Results:**

For the 43 strains subjected to CGH microarray analyses, the common backbone of the *Shigella *genome was estimated to contain more than 1,900 open reading frames (ORFs), with a mean number of 726 undetectable ORFs. The mosaic distribution of absent regions indicated that insertions and/or deletions have led to the highly diversified genomes of pathogenic strains.

**Conclusion:**

These results support the hypothesis that by gain and loss of functions, *Shigella *species became successful human pathogens through convergent evolution from diverse genomic backgrounds. Moreover, we also found many specific differences between different lineages, providing a window into understanding bacterial speciation and taxonomic relationships.

## Background

Gram-negative, facultative anaerobes of the genus *Shigella*, the principal etiologic agents of bacillary dysentery, continue to pose a threat to public health, with an estimated annual incidence of 164.7 million and 1.1 million deaths worldwide [1]. They are sub-grouped into four species: *Shigella dysenteriae*, *Shigella flexneri*, *Shigella boydii *and *Shigella sonnei*. However, classification based upon serotype and other physiological properties has provided limited information regarding the genetic relationship between the species and, moreover, is not sufficient for making disease associations. The results of multilocus enzyme electrophoresis and multilocus sequence typing (MLST) argue that *Shigella *diverged from *Escherichia coli *in eight independent events and, therefore, may not constitute a separate genus [2,3]. However, these results can't reflect the influence of horizontal gene transfer and gene loss. And comparison of genomic differences between different flora and strains will be helpful in revealing gene acquisition and gene loss in bacteria genome evolution, and in revealing the genetic basis of the diversity of biological activities [4].

What remains particularly intriguing about *Shigella*, are the unique epidemiological and pathological features that each of the species exhibits. For example, *Shigella dysenteriae *serotype 1 can cause fatal epidemics in Africa; however, *Shigella boydii *is restricted to the Indian sub-continent, whereas *Shigella flexneri *and *Shigella sonnei *are prevalent in developing and developed countries [1]. Development of a vaccine remains a significant task. Comparative genome information will assist achieving this goal as well as enhance our understanding of the pathogenesis of *Shigella*. Reports on the genome of the two *S. flexneri *2a strains previously revealed a dynamic nature and unique characteristics when compared to the genomes of close relatives, the non-pathogenic K-12 strain and enterohemorrhagic O157:H7 strain of *E. coli *[5,6]. Furthermore, we also have completed a project, which involved sequencing strains of *S. dysenteriae *Sd197 serotype 1, *S. boydii *Sb227 serotype 4 and *S. sonnei *Ss046, all epidemic isolates from the 1950s in China [7]. The release of five *Shigella *sequences has initiated a new era of comparative genomics in *Shigella *biology. However, sequencing remains a laborious and expensive technique, making it difficult to obtain answers concerning the genetic composition of serotypes, or newly emerged variants of interest in a timely manner. The technique of microarray-based comparative genomic hybridization (CGH) provides a valuable adjunct to current protocols used for the assessment of differences and changes in bacterial genetic content. Indeed, this approach has already been utilized in a variety of bacteria to probe for differences between clinical isolates, vaccine strains, species diversity, and disease endemicity [8-11]. There is currently data on five *Shigella *genomes, which can reflect four lineage gene contents of *Shigella*. Such diversified genomic compositions stated previously [5-7] have prompted us to investigate gene distributions among all lineages of *Shigella *using a CGH microarray approach. Herein, we present the results of a genomic comparison of 43 *Shigella *strains based on CGH analysis, which maximizes and extends the information gained from genome sequencing efforts to closely related strains. And this sequence information will provide a valuable resource from which we can begin to dissect shared and distinct features of *Shigella *between different lineages and start exploring how and why these differences arose. In addition, the pattern of acquisitions and deletions detected on the DNA arrays may, to some extent, reflect the gene contents of eight lineages and the evolution of a strain's genome.

## Results

### Analysis of control hybridizations indicates the level of sensitivity of the microarray

Results for the four sequenced *Shigella *strains' hybridization were directly compared to expected hybridization results as assessed by the percent identity of each MG1655 and *Shigella *amplicon to the four sequenced *Shigella *genome sequences. From this analysis, we were able to determine that genes with ≥ 75% identity to the amplicon could be detected as present/conserved on our array, whereas genes that diverged by more than 74% could be assigned as absent/divergent (For details, see Methods). It is noted that positive signals in the CGH analysis may not indicate the presence of functional genes or a pathway. For example, although *fec *genes are present in Sd197, Ss046 and a few other strains, only Ss046 possesses an intact set.

### Genome order analysis of E. coli dataset reveals discrete regions among Shigella serotypes

Generally, the results of the CGH demonstrated that the genome contents of *Shigella *spp. isolates differ markedly from that of *E. coli *strain MG1655. The number of ORFs, which comprised the backbone sequence of *Shigella *spp. strains used in this study, was 1955 (include 12 ORFs which were not present in MG1655), which was less than the number cited in previous reports [10,12]. These conserved ORFs included all 231 essential protein-encoding genes listed in the PEC database except 19 ORFs not spotted on the microarrays [13].

**Figure 1:**
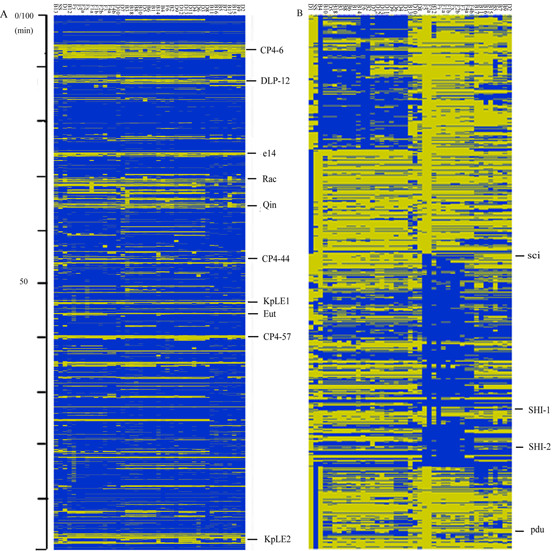
**Genome composition analysis**. Each row corresponds to a specific spot on the array, whereas columns represent strains analyzed and are labelled according to the designations in Table 1. The ORFs status is color-coded: blue, present/conserved; yellow, absent/divergent. (A) The *E. coli *dataset. (B) The *Shigella *ORFs which were not present in MG1655. The region of the *Shigella *ORFs which were not present in MG1655 was enlarged. Ten prophage regions in the MG1655 genome and several selected gene clusters are indicated. SHI-1 and SHI-2 are *Shigella *pathogenicity islands. Sci island is the possible pathogenicity island that has been found in genome Sf301. The *pdu *gene cluster is correlated with propanediol utilization. Strains are labelled according to the designations in Table 1.

2,245 ORFs of MG1655 were found to be absent in at least one strain (see Additional file 1). These ORFs accounted for 52.5% and 53.6% of all MG1655 ORFs annotated and spotted on the slides, respectively. The numbers of absent ORFs ranged from 476 in B5 to 956 in B3 (Table 1). The mean number of absent genes was 726, which is consistent with data from a previous study [14]. There were 137 MG1655 specific ORFs absent in all strains. Among which 64 ORFs were absent in pathogenic *E. coli *too [10]. The mosaic distribution of the absent regions and some gene clusters is shown in Figure 1A. Genes for cell motility, cell envelope, and carbohydrate transport and metabolism genes in the MG1655 genome (Table 2) were frequently found missing in the *Shigella *spp. strains (P < 0.001 based on the two-tailed Student t-test). For example, among the 43 strains of *Shigella *in our study, 35 showed a loss of several flagellar genes to different extents, while the other 8 strains had almost complete flagellar gene sequences. Moreover, *E. coli *surface pili have about 15 gene clusters, among which only 3 (*hofCB-ppdD*, *ppdC-ygdB-ppdBA*, and *hofQ-yrfABCD*) are found in most *Shigella *strains, which is in accordance with previous results [10]. 399 identified ORFs of MG1655 have been annotated with regulatory functions in the PEC database [13]. Out of the 2,245 missing ORFs, 167 are regulatory genes, the majority of which are regulatory transcription factors, including a few global regulatory factors. This result is consistent with the findings from the previous study [10,15]. In addition, *ompT*, encoding outer-membrane protease, was found lost in all the strains, and *cadA*, encoding lysine decarboxylase, was not found in most strains, either.

**Table 1:** *Shigella *spp. strains used in this study and number of MG1655 ORFs absent/divergent

Subgroup	Type No.	Strain	Abbreviation	Source*	No. of absent ORFs^#^
*Dysenteriae*	1	197	D1	CCDC	954
	2	G1252	D2	NKU	535
	3	G1281	D3	NKU	705
	4	G1190	D4	NKU	802
	5	G1213	D5	NKU	792
	6	G1192	D6	NKU	720
	7	G1222	D7	NKU	813
	8	G1221	D8	NKU	561
	9	G1274	D9	NKU	687
	10	G1292	D10	NKU	655
	11	G1246	D11	NKU	745
	12	G1263	D12	NKU	826
	13	G1271	D13	NKU	777
*Flexneri*	1a	571	F1a	CCDC	729
	1b	572	F1b	CCDC	694
	2a	301	F2a	CCDC	675
	2b	251	F2b	CCDC	685
	3	575	F3	CCDC	791
	4a	576	F4a	CCDC	668
	4b	577	F4b	CCDC	687
	5	246	F5	CCDC	678
	6	579	F6	CCDC	784
	Variant x	580	Fx	CCDC	735
	Variant y	581	Fy	CCDC	795
*Boydii*	1	G1228	B1	NKU	736
	2	G1184	B2	NKU	754
	3	G1232	B3	NKU	956
	4	227	B4	CCDC	774
	5	G1186	B5	NKU	476
	6	G1227	B6	NKU	765
	7	G1187	B7	NKU	549
	8	G1268	B8	NKU	835
	9	G1236	B9	NKU	528
	10	G1294	B10	NKU	850
	11	G1191	B11	NKU	588
	12	G1287	B12	NKU	794
	13	G1226	B13	NKU	896
	14	G1300	B14	NKU	907
	15	G1282	B15	NKU	595
	16	G1219	B16	NKU	589
	17	G1214	B17	NKU	663
	18	G1224	B18	NKU	881
Sonnei		46	SS	NICPBP	584

* NKU, NanKai University, Tianjin, China; CCDC, Chinese Center for Disease Control and prevention, Beijing, China; NICPBP, National Institute of the Control of Pharmaceutical and Biological Products, Beijing, China.

# Altogether, 2,245 ORFs were absent in at least one strain, and 137 ORFs were commonly absent in 43 strains.

**Table 2:** Classification according to the function categories of absent ORFs in *Shigella *spp. strains

Description	MG1655 total ORFs	Absent MG1655 ORFs in *Shigella *spp. strains*								
		
		Total (%) #	C1 (%) #	C2 (%) #	C3 (%) #	SS (%) #	D1 (%) #	D8 (%) #	D10 (%) #	B13 (%) #
**Information storage and processing**										
Translation, ribosomal structure and biogenesis	171	22(12.9)	16(9.4)	6(3.5)	8(4.7)	4(2.3)	5(2.9)	2(1.2)	3(1.8)	4(2.3)
RNA processing and modification	2	1(50.0)	1(50.0)	1(50.0)	1(50.0)	0	0	0	1(50.0)	0
Transcription	280	173(61.8)	126(45.0)	91(32.5)	108(38.6)	46(16.4)	70(25.0)	38(13.6)	52(18.6)	83(29.6)
Replication, recombination and repair	220	79(35.9)	64(29.1)	49(22.3)	49(22.3)	24(10.9)	31(14.1)	24(10.9)	35(15.9)	35(15.9)
**Cellular processes**										
cell division and chromosome partitioning	34	7(20.6)	4(11.8)	6(17.6)	2(5.9)	1(2.9)	2(5.9)	1(2.9)	0	3(8.8)
Defense mechanisms	48	22(45.8)	12(25.0)	12(25.0)	13(27.1)	7(14.6)	13(27.1)	7(14.6)	9(18.8)	12(25.0)
Signal transduction mechanisms	134	67(50.0)	54(40.3)	29(21.6)	37(27.6)	8(6.0)	27(20.1)	13(9.7)	10(7.5)	19(14.2)
cell envelope biogenesis, outer membrane	235	122(51.9)	90(38.3)	71(30.2)	69(29.4)	28(11.9)	48(20.4)	26(11.1)	35(14.9)	42(17.9)
cell motility and secretion	107	89(83.2)	79(73.8)	45(42.1)	70(65.4)	29(27.1)	65(60.7)	35(32.7)	36(33.6)	51(41.7)
Intracellular trafficking, secretion, and vesicular transport	37	14(37.8)	7(18.9)	9(24.3)	10(27.0)	7(18.9)	6(16.2)	4(10.8)	5(13.5)	5(13.5)
Posttranslational modification, protein turnover, chaperones	128	30(23.4)	15(11.7)	8(6.3)	8(6.3)	1(0.8)	6(4.7)	4(3.1)	2(1.6)	7(5.5)
**Metabolism**										
Energy production and conversion	275	151(54.9)	94(34.2)	59(21.5)	87(31.6)	24(8.7)	47(17.1)	17(6.2)	31(11.3)	34(12.4)
Carbohydrate transport and metabolism	368	249(67.7)	182(49.4)	109(29.6)	133(36.1)	69(18.8)	116(31.5)	58(15.8)	58(15.8)	97(26.4)
Amino acid transport and metabolism	350	152(43.4)	106(30.3)	58(16.6)	85(24.3)	19(5.4)	38(10.9)	24(6.9)	29(8.3)	44(12.6)
Nucleotide transport and metabolism	87	33(37.9)	22(25.3)	13(14.9)	17(19.5)	6(6.9)	12(13.8)	7(8.0)	8(9.2)	11(12.6)
Coenzyme transport and metabolism	123	26(21.1)	17(13.8)	7(5.7)	14(11.4)	2(1.6)	7(5.7)	0	2(1.6)	4(3.3)
Lipid transport and metabolism	83	36(43.4)	26(31.3)	19(22.9)	20(24.1)	13(15.7)	17(20.5)	6(7.2)	10(12.0)	13(15.7)
Inorganic ion transport and metabolism	191	98(51.3)	64(33.5)	55(28.8)	59(30.9)	11(5.8)	25(13.1)	21(11.0)	19(9.9)	39(20.4)
Secondary metabolites biosynthesis, transport and catabolism	68	46(67.6)	29(42.6)	22(32.4)	25(36.8)	6(8.8)	16(23.5)	5(7.4)	13(19.1)	21(30.9)
**Poorly characterized**										
General function prediction only	338	178(52.7)	135(39.9)	95(28.1)	90(26.6)	31(9.2)	59(17.5)	37(10.9)	43(12.7)	63(18.6)
Function unknown	308	154(50.0)	101(32.8)	64(20.8)	85(27.6)	29(9.4)	49(15.9)	16(5.2)	33(10.7)	43(14.0)
**Not in COGs**	692	496(71.7)	413(59.7)	305(44.1)	359(51.9)	219(31.6)	295(42.6)	216(31.2)	221(31.9)	266(38.4)
**Total**	4279	2245(52.5)	1657(38.7)	1133(26.5)	1349(31.5)	584(13.6)	954(22.3)	561(13.1)	655(15.3)	896(20.9)

* C1 contains D strains (D3, D4, D5, D6, D7, D9, D11, D12, and D13) and B strains (B1, B2, B3, B4, B6, B8, B10, B14, and B18) with F6 as minorities. C2 is mainly composed of B strains (B5, B7, B9, B11, B15, B16, B17) and D2. C3 consists mostly of F strains (F1a, F1b, F2a, F2b, F3, F4a, F4b, F5, Fx, Fy) and B12. *Shigella *strains are labelled according to the designations in Table 1.

^#^The % indicated in parenthesis is relative to MG1655 total ORFs.

In general, what we found was that the gene contents of most categories are more variable in C1 strains than those in others. For instance, 49.4% and 40.3% genes coding for carbohydrate and amino acid transport proteins and signal transduction proteins respectively, were absent from at least one strain of C1. In contrast, strains from C2 shared relatively more core genes of most categories than those from the other two groups (Table 2). However, it should be noted that the three *Shigella *groups consist of different number of strains which may also be a key influence to the count of conserved genes here.

The alterations were observed scattered over the entire *E. coli *MG1655 chromosome. However, the prophages of strain MG1655 represent chromosomal variation "hot spots." At least 10 prophages and phage like regions were identified in MG1655 [16,17], but few were intact among *Shigella *spp. strains.

### Genes for transport and metabolism of carbohydrates

*E. coli *is able to utilize several types of carbohydrates. However, *Shigella *has lost the capability to use some carbohydrates due to the loss of several transport and conjugated genes, which are also the basis for the biochemical typing of *Shigella *spp. strains. CGH Result indicated that the missing genes were different in different strains. By means of CGH analysis, it was found that the *lac *operon is only intact in B9 and B15, while *lacA *and *lacY *are absent in all other strains. This result conforms to the observation that *Shigella *cannot ferment lactose. Moreover, the metabolism of mannitol, melitose and glycerol are also in accordance with the CGH results, however, results from galactitol and rhamnose show discordance with the CGH data. The reason for this discrepancy requires further investigation. Furthermore, the fact that *Shigella *is unable to use xylose differs from the CGH result with *S. flexneri*, and may be explained to a certain extent by a terminating mutation found occurring in *xylA*, the gene encoding D-xylose isomerase [5]. The CGH results for genes related to transport and metabolism of some carbohydrates are shown in additional file 2.

### Analysis of known and putative virulence-associated genes

**Figure 2:**
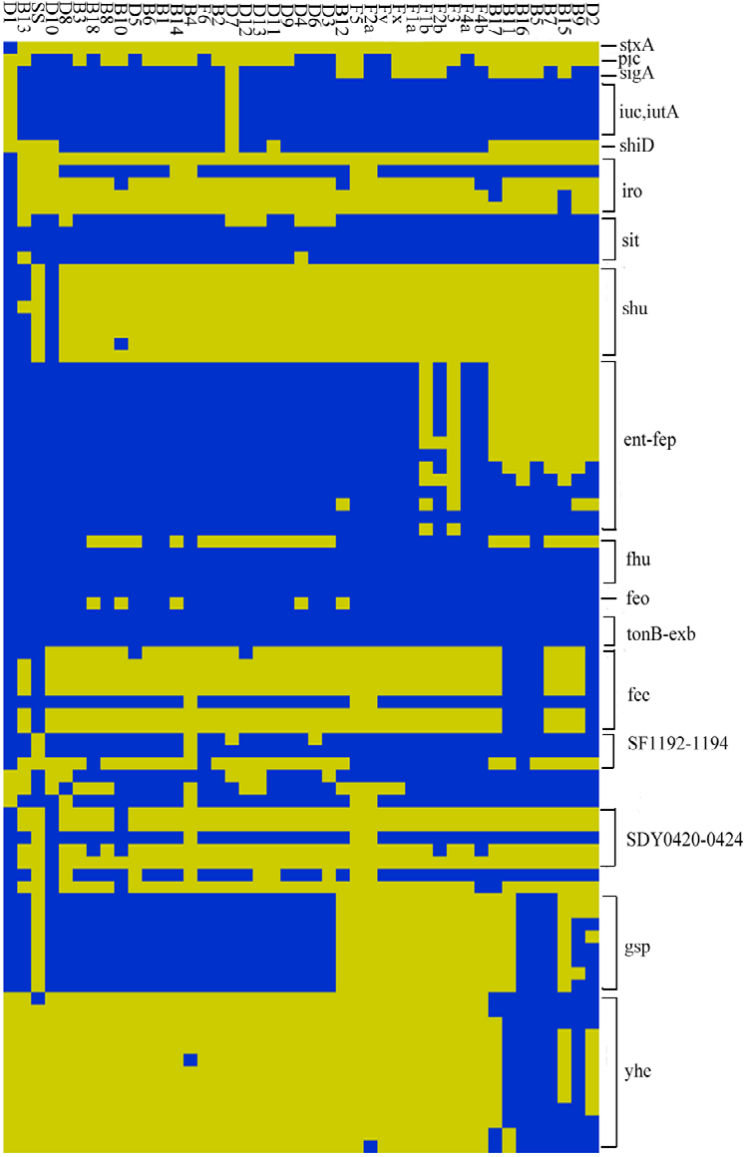
**The distribution of the known and putativevirulence related genes among *Shigella *spp. strains**. The ORFs status is color-coded: blue, present/conserved; yellow, absent/divergent. The designations of these genes are indicated on the right. Strains are labelled according to the designations in Table 1. The product of stxA is shiga toxin. SigA and Pic are serine proteases. The gene clusters of *iuc*, *iutA*, *iro*, *sit*, *shu*, *ent-fep*, *fhu*, *feo*, *tonB-exb*, *fec *and SF1192-1194 are all iron acquisition associated. Two type II secretion systems are encoded by *yhe *and *gsp *respectively. The products of SDY0420-0424 are exoproteins.

There were 886 *Shigella *ORFs that were not present in MG1655 (non-pathogenic *Escherichia coli *strain) on the microarray (see Figure 1B and Additional file 3 for the details). Since part of them were also present in some pathogenic *Escherichia coli *strains, those ORFs may be putative virulence-associated genes. Shiga toxin was only found in D1. Previously identified *Shigella *pathogenicity islands SHI-1 and SHI-2 are absent in Sd197 but present in other sequenced strains (SHI-3 from *S. boydii *is essentially identical as SHI-2) [5-7]. SHI-1 encodes an enterotoxin ShET1 and proteases SigA and Pic, all implicated in virulence [18] and SHI-2 (SHI-3) encode an aerobactin system for iron acquisition [19,20]. Variants of the SHI-1 and SHI-2 (SHI-3) that were missing one or more marker regions were found in most *Shigella *strains. The *sigA *gene was conserved in most strains, while the *pic *gene was absent. SHI-2 is almost complete in *S. flexneri *while lost in D1 and D7. The *iuc *and *iutA *genes that encode siderophore and its receptor in the SHI-2 (SHI-3) island are present in all of the other strains. Sci island, the possible pathogenicity island that has been found in genome Sf301 [5], almost exist in all *S. flexneri *strains except F6, but are all missing in the other strains. And the *pdu *gene cluster, which is correlated with propanediol utilization, only exists in SS. The result of complete *Shigella *islands is showed in Figure 1B, while the result of selected virulence genes is showed in Figure 2.

Compared with *E. coli *k-12, most *Shigella *strains are missing *fec*, but have retained *feo*, *fep*, and *fhu*, and have even developed their own iron transport system (*sit*, *iuc*, *iro *and *shu *etc.). The type II secretion system (T2SS) encoded by genes of the general secretion pathway is widely distributed in Gram-negative bacteria [7]. The well-known *E. coli *T2SS, encoded by the *yhe *genes at 74.5 min of the MG1655 chromosome, is absent in all sequenced *Shigella *genomes but present in several tested strains. Moreover, there is a novel set of *gsp *genes in the Sd197 and Sb227 chromosomes, which is also present in all C1 strains (see below for details) and several other strains (Figure 2).

### Lineage relationships are revealed by phylogenic analysis

**Figure 3:**
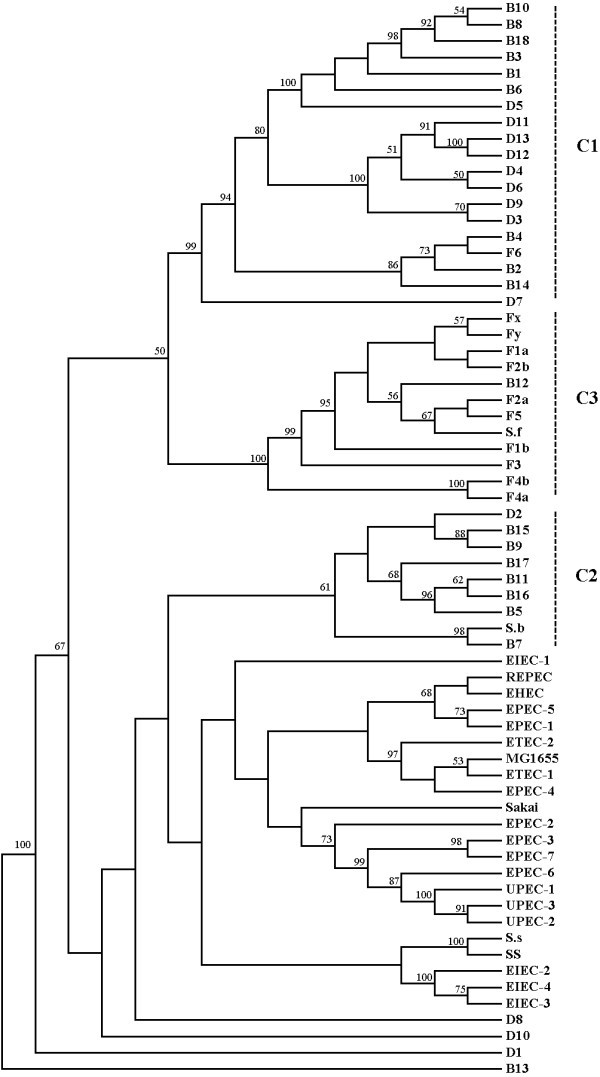
**Phylogenic analysis of *Shigella *strains. Data were compiled from this study, and [10]. Details on tree generation are described in Materials and Methods**. Phylogenetic tree generated by the neighbor-joining method for the combined data of 66 strains (Table 1 and additional file 5). For *Shigella *strains used in our study, *S. dysenteriae*, *S. flexneri*, *S. boydii *and *S. sonnei *are abbreviated to D, F, B, and SS respectively, followed by the serotype number. *E. coli *k-12 MG1655 is abbreviated to MG1655. For pathogenic *E. coli *and *Shigella *strains used in previous study, the strains' abbreviations are according to the additional file 5. Bootstrap values greater than 50% are indicated at the nodes. The three major clusters of *Shigella *were indicated by vertical dashed lines in the right.

Using the above CGH microarray data and the previously published data [10], we performed phylogenic analysis (Figure 3). The phylogenic tree shows that most of the *Shigella *strains can be grouped into three clusters (C1, C2 and C3) leaving SS, D1, D8, D10 and B13 as additional minor branches. SS is closer than D8, D1, D10 and B13 to the main clusters. C1 contains D strains (D3, D4, D5, D6, D7, D9, D11, D12, and D13) and B strains (B1, B2, B3, B4, B6, B8, B10, B14, and B18) with F6 as minorities. C2 is mainly composed of B strains (B5, B7, B9, B11, B15, B16, B17) and D2. C3 consists mostly of F strains (F1a, F1b, F2a, F2b, F3, F4a, F4b, F5, Fx, and Fy) and B12. The results are in good agreement with the MLST result [3], supporting the hypothesis that *Shigella *species originated from multiple *E. coli *strains with diverse genetic backgrounds. Furthermore, two *sonnei *strains are grouped with three EIEC strains, and other pathogenic *E. coli *strains are grouped together. The high bootstrap values of most branches confirm the robustness of the tree.

In addition to what was discussed above, we also found many specific differences between different lineages, providing a window into understanding bacterial speciation and taxonomic relationships (Additional file 1). For example, *ybcZ/ylcA*, which encodes a two-component signal transduction system that is responsive to copper ions, is absent in three lineages (D1, D8 and cluster 1 strains), and the locus *glc*, which is associated with the glycolate utilization trait in *E. coli*, is only present in cluster 3 strains (except B12), several cluster 2 strains and B13. The locus *aga*, which is related to acetylgalactosamine metabolism, is only conservative in cluster 2, B13 and D10.

F6 sits in a different cluster as compared to the other *flexneri *strains and we found many differences that set it apart (Additional file 4). For example, *gsp *genes, *yea *genes, *waaWYJI*, *rhsABC *and several phage-related genes were present in F6 and absent in other *S. flexneri strains*, while the *cai-fix *gene cluster, *ybcZ-ylcA-ylcB*, *marAB*, *yehABCDE*, *glc *genes, *dgoTAK*, *bglBFG*, *yih *genes, *malGFE *and *safABC *were absent in F6 but present in other members of *S. flexneri*.

## Discussion

Abundant information is currently available for research on the genome evolutions of different organisms and the exploration of the roles played by different genes in their life processes. However, more and more evidence has revealed that most bacteria have shown unexpected diversity during the evolutionary process, even within one species [21].

### Advantages and disadvantages of CGH technique

In order to systematically assess the genetic variability of bacteria, several genome comparison techniques, e.g., multilocus enzyme electrophoresis, MLST, pulsed-field gel electrophoresis and restriction fragment length polymorphisms, have been used. Microarray-based CGH has emerged as a revolutionary platform for comparative genomics that has recently been used for the analysis of genome variability among bacterial species or closely related bacteria. Given that the sequencing of strains on a large scale is time-consuming, laborious and unfeasible at present, CGH may resolve the problem to some extent by applying the available genome sequence information to closely related species. And this method can supply more information about genome composition and provide opportunity to analysis unsequenced strain on genomic scale. CGH proved a useful tool in this study for identifying genetic differences between different *Shigella *lineages over other techniques. Even though this technique has several limitations as described before [14], we believe, based on the criteria for data analysis, which we described before, that the use of CGH technology allows sufficient assessment of the genetic diversity and gene content among *Shigella *spp. strains.

### Genomic diversity of Shigella

An MLST study [3] and five reported *Shigella *genomes [5-7] have suggested strongly that *Shigella *is derived from multiple origins of *E. coli*. And the five *Shigella *genomes vary in size from 4.3 to 4.8 Mb. Our study has revealed extensive diversity among *Shigella *genomes, forming a genetic basis to explain species/strain specific epidemiological and pathological features. The phylogenic analysis results supporting the hypothesis that *Shigella *have emerged from multiple independent origins. The fact that two *sonnei *strains are grouped with three EIEC strains supports the hypothesis that EIEC strains are in an intermediate stage and are a potential precursor of "full-blown" *Shigella *strains [22]. Based on our results, we believe that the reason why *Shigella *exhibits unique epidemiological and pathological features is due to the loss of several genes and gene acquisition as described above. We found many specific differences between different lineages that never been described before. Previous study showed that there was over-representation of regulatory genes in the missing ORFs in *Shigella*/EIEC strains [15]. Our result reconfirmed this observation. How these genomic differences account for the differences in epidemiology and pathology remain to be elucidated. A comparison with respect to the new group relationship is particularly significant in providing insight into the virulence of *Shigella*, such as F6, D8, D10, B13, etc. Furthermore, F6, which is distinct from other *flexneri *strains in several ways, should not be considered a *flexneri *member. And our results support the Brenner's suggestion to transfer F6 to the Boydii subgroup [23].

Currently, there are data on five *Shigella *genomes, which can reflect only four lineage gene contents of *Shigella*. Our CGH results may, to some extent, reflect the entire genome diversity within *Shigella *and the evolution of a strain's genome. In addition, we found a large number of deletions in different genes of the relevant operon in carbohydrate utilization, emphasizing strong evidence for the extinction of these metabolic pathways. After *Shigella *became pathogenic to humans, the host presented a constant environment rich in metabolic intermediates, some genes were rendered useless by adoption of a strictly pathogenic life-style. These superfluous sequences were eliminated through mutational bias favouring deletions, a process apparently universal in bacterial lineages [24].

The findings that diverse mechanisms appear to be responsible for same biochemical characteristics reconfirm the convergent evolution that *Shigella *might have experienced. For example, *Shigella *strains cannot decompose lactose, but each has resulted from a different mechanism. Some strains lost the *lac *operon completely, while others only lost *lacA *and *lacY*. The reasons to no flagellum and no motility of *Shigella *are also diverse.

### Genetic basis for variation in virulence

Understanding the virulence of *Shigella*, in addition to the CGH data, facilitates our understanding of the scenario that underlies the difference between these organisms. The pattern of acquisitions and deletions detected, may explain these differences to some extent. On loss and gain functions, it is important to recognize these differences may enhance the virulence or lead to variation in virulence. Variants of SHI-1 that were missing one or more marker regions were found in most *Shigella *strains. The SHI-2 island is only absent in strains D1 and D7, indicating its importance to most of the strains, and the unique iron acquisition mechanisms in the two D strains. Furthermore, our results indicate that SHI-1 and SHI-2 are genetic elements that have disseminated throughout *Shigella *and diverged into distinct structural forms, emphasizing their importance in *Shigella *pathogenesis.

Previous study argues that the *gsp *genes in *S. dysenteriae*, encoding the T2SS, ought to contribute significantly to pathogenicity as it enables Stx to reach the target host cells from proliferating bacteria[7]. Our CGH result indicates a wide distribution of the *gsp *genes among strains from all phylogenetic groups which suggests that many strains possessed Stx before their subsequent loss. Perhaps, loss of Stx genes has provided advantages to the bacteria for a better adaptation to the human hosts as causing severer disease offers little benefit to the organisms for long term survival. It is known that the deletion of certain genomic regions present in *E. coli *(so-called "black holes") enhances the virulence of *Shigella *[25,26]. These pathoadaptive deletions could be identified as "absent" on the arrays. Our CGH results demonstrated the existence of those "black holes" in all lineages.

The comparative genomic analysis between pathogenic and non-pathogenic *E. coli *strains reveals that a specific genetic background is required for acquisition and expression of virulence factors [27]. Furthermore, we found that the iron transport system, which is virulence related, is reinforced in *Shigella *by the development of its own iron transport system, in addition to the retention of most of the iron transport system from *E. coli*. *Shigella *species express numerous iron acquisition systems, reflecting the importance of obtaining iron. A previous study which focused on transcriptome polymorphism of *Shigella*/EIEC also indicated that it was important to acquire iron for *Shigella*/EIEC [15]. These results indicate that the acquisition of some genes has enhanced the ability of *Shigella *species to adapt to the complicated host environment during its evolution into an intestinal pathogen.

## Conclusion

In conclusion, the comparisons performed in this study are necessary for further understanding the implications of genetic background in the evolution of *Shigella *pathogenicity. These findings provide an invaluable genetic basis for future studies examining bacterial evolution, as well as pathogenicity, and the development of novel strategies for the prevention and treatment of shigellosis.

## Methods

### Bacterial strains and growth condition

We used 43 *Shigella *strains to represent the known serotypes. Details are given in Table 1. *Shigella *strains were routinely grown at 37°C overnight on Luria-Bertani agar plates containing 0.01% congo red. Red colonies were inoculated into Luria-Bertani broth without antibiotics and grown overnight at 37°C with shaking (260 rpm) for isolating genomic DNA. And *E. coli *K-12 MG1655 strain was routinely grown at 37°C overnight on Luria-Bertani agar plates for isolating genomic DNA. Genomic DNA was extracted by using Wizard^® ^Genomic DNA Purification Kit (Promega).

### Microarray fabrication

The microarrays used in this study featured 4,188 of the 4,279 ORFs identified in *E. coli *K-12 strain MG1655 [17], because 91 ORFs were not on the microarray of the inability to amplify these products. Furthermore, there were 934 ORFs from 4 *Shigella *strains (Sf301, Sd197, Sb227 and Ss046) which were not present in MG1655 also in the microarray. The ORFs cover the known and putative virulence genes from the *Shigella *chromosome.

ORFs were amplified with specific primer pairs (Invitrogen). To ensure that the elements of our array would detect specifically their corresponding genes and no others, the ORF coordinates fed into the primer program were circumscribed such that they would exclude regions of any ORF that contained significant similarity to any other ORF (70% identity over 70 nucleotides was considered to be significant). Since there were ORFs from different strains, different genomic DNA was used as target according to the dataset.

Agarose gel electrophoresis was used to perform quality control on all PCR products. Oligonucleotides were removed from the PCR mix by PCR 96 cleanup plate (Millipore). DNA was resuspended in 12 μl of spotting solution containing 50% dimethyl sulfoxide. PCR products were spotted onto gamma amino propylsilan coated GAPII slides (Corning) with a Cartesian^® ^arrayer (Cartesian). And arrayed slides were blocked prior to hybridization as described previously [28].

### Probe preparation and hybridization

For each microarray hybridization reaction, two micrograms of genomic DNA (for test strains) and two microgram reference sample (mixed genomic DNA of MG1655, Sf301, Sd197, Sb227 and Ss046) were fluorescently labelled with Cy5- dCTP or Cy3-dCTP (Amersham) respectively. The separate labelling reactions were pooled after each respective Cy dye incorporation step and then again divided into aliquots to minimize inconsistencies in probe generation. Hybridizations were performed as described previously [29]. Competitive hybridization was done at least three times for one strain.

### Microarray data analysis

The processed slides were scanned with a GenePix 4100A scanner (Axon). Fluorescent spots and the local background intensities were quantified with GenePix Pro 5.0 software (Axon). The local background value was subtracted from the intensity of each spot. The mean of the signal intensities of the control spots hybridized with labelled reference genomic DNA in each experiment was calculated. Ten different *A. thaliana *genes and human β-actin gene from SpotReport™ cDNA Array Validation System (Stratagene) were used for the controls. The spots that showed intensity with labelled reference genomic DNA that was lower than the mean value of the control spots were excluded from further analysis. The mean log_2 _Cy5/Cy3 (sample/reference) ratios of signal intensity were calculated for analysis. In addition, spots that gave invalid results for more than 20% of the strains were removed. The final data set was 5,122 ORFs. The multiple arrays for each strain were averaged across the datasets. CGH data analysis was done by using Microsoft Excel and a microarray genomic analysis program called GACK according to the previous reports [30]. GACK is capable of dynamically generating cut-offs for conserved/divergent gene analysis for each array hybridization and functions independently of any normalization process that would otherwise be strongly influenced by differences between the reference strain and test strains.

The hybridization data for the four Sf301, Sd197, Sb227 and Ss046 arrays were filtered by using the same parameters indicated above to avoid copy number effect. The percent similarity for the highest high-scoring profile of each amplicon in the four Shigella genomes was obtained by using WU-BLAST. This percent similarity was compared to the averaged logRAT2N of the hybridization data. Of the 5,122 ORFs that were retrieved, 60 ORFs had hybridization results contrary to what was expected from the percent similarity analysis. A total of 12 of these were false positives, whereas 48 of were false negatives. This list was used as an additional filter for the data. Genome order analysis was performed by organizing the spots for the entire data set in their genome order according to the MG1655 and Sb227, Sd197, Sf301 and Ss046 annotation and viewed with TMEV [31].

### Dataset used in the study

Final datasets can be obtained at ShiBASE [32]. In addition, the microarray data has been deposited in the Gene Expression Omnibus (GEO) under accession number GSE5212.

Apart from the CGH dataset mentioned above, a similar array CGH data previously published on 19 pathogenic *E. coli *strains and three *Shigella *strains was also included in the analysis[10]. The designation of these strains are showed in additional file 5.

### Phylogenic analysis

The *E. coli *data set obtained from the CGH analysis (0 = absent and 1 = present) was fed into the Phylip software (version 3.6 by Joseph Felsenstein, Department of Genetics, University of Washington, Seattle). Maximum parsimony (MP) trees with equal weighting of characters were drawn by a branch-and-bound search. Tree reliability was assessed using non-parametric bootstrap re-sampling of 100 replicates.

## Authors' contributions

The first three authors contributed equally. JP carried out experiment design, analysed data, and prepared the manuscript. XZ did microarray fabrication and hybridization. JY analysed data. JW cultured strains and did microarray fabrication. EY did PCR amplification and genomic DNA extraction. WB did purification PCR products. CW did part of the data analysis. MS did part of the microarray fabrication and image acquirement. QJ designed the study, analysed data, and reviewed the manuscript. All authors read and approved the final manuscript.

## Supplementary Material

Additional File 1 Absent ORFs of *E. coli *K-12 strain MG1655 among *Shigella *spp. strains. Click here for file

Additional File 2 ORFs in each *Shigella *genome related to main clinical biochemical reaction. Click here for file

Additional File 3 *Shigella *ORFs dataset which were not present in MG1655. Click here for file

Additional File 4 Difference between F6 and other *S. flexneri *members. Click here for file

Additional File 5 Strains used in previous study. Click here for file
