# Supplementary material for: The use of comparative genomic hybridization to characterize genome dynamics and diversity among the serotypes of Shigella
Source: BMC Genomics. 2006 Aug 29;7:218. doi: 10.1186/1471-2164-7-218 (PMC3225857; doi:10.1186/1471-2164-7-218)
Supplement: Additional File 1 — Absent ORFs of E. coli K-12 strain MG1655 among Shigella spp. strains. [file 1471-2164-7-218-S1.pdf]

Name	D1	B13	SS	D10	D8	B3	B18	B8	B10	D5	B6	B1	B14	B4	F6	B2	D7	D12	D13	D11	D9	D4	D6	D3	B17	B11	B16	B5	B7	B15	B9	D2	B12	F5	F2a	Fy	Fx	F1a	F1b	F2b	F3	F4a	F4b	Gene	Product
b0033a	1	1	1	1	1	1	1	1	1	0	1	1	1	0	0	1	1	0	0	0	1	0	1	0	1	1	1	1	1	1	1	1	1	1	1	1	1	1	1	1	1	1	1	yaaV	Uncharacterized conserved protein
b0034	1	1	1	1	1	0	0	0	0	0	0	0	0	0	0	0	1	0	0	0	0	0	0	0	1	1	1	1	1	1	1	1	1	1	1	1	1	1	1	1	1	1	1	caiF	transcriptional regulator of cai operon
b0035	1	1	1	1	1	0	0	0	0	0	0	0	0	0	0	0	1	0	0	0	0	0	0	0	1	1	1	1	1	1	1	1	1	1	1	1	1	1	1	0	1	1	1	caiE	possible synthesis of cofactor for carnitine racemase and dehydratase
b0036	1	1	1	0	1	0	0	0	0	0	0	1	0	0	0	0	1	0	0	0	0	0	0	0	1	1	1	1	1	1	1	1	1	1	1	1	1	1	1	1	1	1	1	caiD	carnitine racemase
b0037	1	1	1	1	1	0	0	0	0	0	0	0	0	0	0	0	1	0	0	0	0	0	0	0	1	1	1	1	1	1	1	1	1	1	1	1	1	1	1	1	1	1	1	caiC	probable crotonobetaine/carnitine-CoA ligase
b0038	1	1	1	1	1	0	0	0	0	0	0	0	0	0	0	0	1	0	0	0	0	0	0	0	1	1	1	1	1	1	1	1	1	1	1	1	1	1	1	1	1	1	1	caiB	l-carnitine dehydratase
b0039	1	1	1	1	1	0	0	0	0	0	0	0	0	0	0	0	1	0	0	0	0	0	0	0	1	1	1	1	1	1	1	1	1	1	1	1	1	1	1	1	1	1	1	caiA	probable carnitine operon oxidoreductase
b0040	1	1	1	1	1	0	0	0	0	0	0	0	0	0	0	0	1	0	0	0	0	0	0	0	1	1	1	1	1	1	1	1	1	1	1	1	1	1	1	1	1	1	1	caiT	probable carnitine transporter
b0041	1	1	1	1	1	1	1	1	1	0	1	1	1	0	1	1	1	0	0	0	1	1	1	0	1	1	1	1	1	1	1	1	1	1	1	1	0	1	1	1	1	1	1	fixA	probable flavoprotein subunit, carnitine metabolism
b0042	1	1	1	1	1	0	0	0	0	0	0	1	0	0	0	0	1	0	0	0	0	0	0	0	1	1	1	1	1	1	1	1	1	1	1	1	1	1	1	0	1	1	1	fixB	probable flavoprotein subunit, carnitine metabolism
b0043	1	1	1	1	1	0	0	0	0	0	0	1	0	0	0	0	1	0	0	0	0	0	0	0	1	1	1	1	1	1	1	1	1	1	1	1	1	1	1	0	1	1	1	fixC	flavoprotein; electron transport
b0044	1	1	1	1	1	0	0	0	0	0	0	0	0	0	0	0	1	0	0	0	0	0	0	0	1	1	1	1	1	1	1	1	0	1	1	1	1	1	1	1	1	1	1	fixX	putative ferredoxin
b0056	1	0	0	1	0	0	0	0	0	0	0	0	0	0	0	0	0	0	0	0	0	0	0	0	0	0	0	0	0	0	0	0	0	0	0	0	0	0	0	0	0	0	0	yabP	orf, hypothetical protein
b0057	0	0	0	0	0	0	0	0	0	0	0	0	0	0	0	0	0	0	0	0	0	0	0	0	0	0	0	0	0	0	0	0	0	0	0	0	0	0	0	0	0	0	0	yabQ	orf, hypothetical protein
b0070	0	0	1	0	1	1	1	1	1	1	1	1	1	1	1	1	1	1	1	1	1	1	1	1	1	0	0	0	0	0	0	1	1	1	1	1	1	1	1	1	1	1	1	yabM	putative transport protein
b0081	1	1	1	1	1	1	1	1	1	1	1	1	1	1	1	1	1	1	1	1	1	1	1	1	1	1	1	1	1	1	1	1	1	1	1	1	0	1	1	1	1	1	1	yabB	orf, hypothetical protein
b0135	1	0	1	0	0	0	0	0	0	0	0	0	0	0	0	0	0	0	0	0	0	0	0	0	0	0	0	0	0	0	0	0	0	1	1	0	0	0	0	0	0	0	0	yadC	putative fimbrial-like protein
b0136	0	1	1	1	1	0	1	1	1	1	1	1	1	0	1	1	1	1	1	1	1	1	1	1	0	0	0	1	0	0	0	1	0	0	0	1	1	1	1	1	1	1	1	yadK	putative fimbrial protein
b0137	0	0	1	0	0	0	0	0	0	0	0	0	0	0	0	0	1	0	0	0	0	0	0	0	1	1	1	1	1	1	1	1	0	0	0	0	0	0	0	0	0	0	0	yadL	putative fimbrial protein
b0138	0	0	1	0	0	0	0	0	0	0	0	0	0	0	0	0	1	1	1	0	0	0	0	0	1	1	1	1	1	1	1	1	0	0	0	0	0	0	0	0	0	0	0	yadM	putative fimbrial-like protein
b0139	0	0	1	0	0	0	0	1	1	1	1	1	1	1	1	1	0	0	1	1	1	1	1	1	1	1	1	1	1	1	1	1	0	0	0	0	0	0	0	0	0	0	0	htrE	probable outer membrane porin protein involved in fimbrial assembly
b0140	0	0	1	0	0	0	0	0	0	0	0	0	1	1	1	1	0	1	1	1	1	1	1	1	1	1	1	1	1	1	1	1	0	0	0	0	0	0	0	0	0	0	0	ecpD	probable pilin chaperone similar to PapD
b0141	0	0	1	0	0	1	1	1	1	1	1	1	0	1	1	1	1	1	1	1	1	1	1	0	1	1	1	1	1	1	1	1	0	0	0	0	0	0	0	0	0	0	0	yadN	putative fimbrial-like protein
b0150	1	1	1	1	1	1	0	0	0	0	1	1	0	1	0	0	0	0	0	0	0	0	0	0	0	0	0	1	0	0	0	0	1	1	1	1	1	1	1	1	1	1	1	fhuA	outer membrane protein receptor for ferrichrome, colicin M, and phages T1, T5, and phi80
b0164	0	1	1	0	1	1	1	1	1	1	1	1	1	1	1	1	1	1	1	1	1	1	1	1	1	1	1	1	1	1	1	1	0	0	0	0	0	0	0	0	0	0	0	yaeI	orf, hypothetical protein
b0217	0	1	1	1	1	1	1	1	1	1	1	1	1	0	1	1	1	0	0	1	1	1	1	1	1	1	1	1	1	1	1	1	1	0	0	1	0	1	1	1	1	1	1	yafT	putative aminopeptidase
b0218	0	1	1	0	0	0	0	0	0	0	0	0	0	0	0	0	0	0	0	0	0	0	0	0	1	1	1	1	1	1	0	1	0	0	0	0	0	0	0	0	0	0	0	yafU	orf, hypothetical protein
b0219	1	1	1	1	1	1	0	1	1	1	1	1	1	1	1	1	0	1	1	1	1	1	1	1	1	0	0	1	1	1	1	1	1	1	1	1	1	1	1	1	1	1	1	yafV	putative EC 3.5. amidase-type enzyme
b0220	1	1	1	1	1	1	1	1	1	1	1	1	1	1	1	1	0	1	1	1	1	1	1	1	1	1	1	1	1	1	1	1	1	1	1	1	1	1	1	1	1	1	1	ykfE	orf, hypothetical protein
b0221	1	1	1	1	1	1	1	1	1	1	1	1	1	1	1	1	1	1	1	1	1	1	1	1	1	0	1	1	1	1	1	1	1	1	1	1	1	1	1	1	1	1	1	yafH	putative acyl-CoA dehydrogenase (EC 1.3.99.-)
b0225	1	1	1	0	1	0	0	0	0	0	0	0	0	0	0	0	0	0	0	0	0	0	0	0	1	1	1	1	1	1	1	1	0	0	0	0	0	0	0	0	0	0	0	yafQ	orf, hypothetical protein
b0226	1	0	1	0	1	0	0	0	0	0	0	0	0	0	0	0	0	0	0	0	0	0	0	0	1	1	1	1	1	1	1	1	0	0	0	0	0	0	0	0	0	0	0	dinJ	damage-inducible protein J
b0227	1	1	1	1	1	1	1	1	1	1	1	1	1	1	1	1	1	1	1	1	1	1	1	1	0	0	0	0	1	1	1	1	1	1	1	1	1	1	1	1	1	1	1	yafL	putative lipoprotein
b0228	1	1	1	0	0	0	0	0	0	1	0	1	1	0	1	1	1	1	1	1	1	1	1	1	0	0	0	0	1	1	1	0	1	1	1	1	1	1	1	1	1	1	1	yafM	orf, hypothetical protein
b0229	0	1	1	1	0	1	0	0	1	0	1	1	1	0	1	1	1	1	1	1	1	1	1	1	1	0	0	1	1	1	1	1	1	1	1	1	1	1	1	1	1	1	1	fhiA	flagellar biosynthesis
b0230	1	1	1	1	0	1	0	0	0	0	0	1	1	0	1	1	1	1	1	1	1	1	1	1	0	0	0	0	0	1	1	1	1	1	1	1	1	1	1	1	1	1	1	mbhA	putative motility protein
b0231	1	1	1	1	1	1	1	1	1	1	1	1	1	0	1	0	1	1	1	1	1	1	1	1	1	1	1	1	1	1	0	1	1	1	1	1	1	1	1	1	1	1	1	dinP	damage-inducible protein P; putative tRNA synthetase
b0232	0	0	0	1	0	0	0	0	0	0	0	0	0	0	0	0	1	0	0	0	1	0	0	0	0	0	0	0	0	0	0	0	1	1	1	1	1	1	1	1	1	1	1	yafN	orf, hypothetical protein
b0233	0	0	0	1	0	0	0	0	0	0	0	0	0	0	0	0	0	0	0	0	0	0	0	0	0	0	0	0	0	0	0	0	1	1	1	1	1	1	1	1	1	1	1	yafO	orf, hypothetical protein
b0234	1	1	1	1	1	1	1	1	1	1	1	1	1	0	1	0	1	1	1	1	1	1	1	1	1	1	1	1	1	1	0	1	1	1	1	1	1	1	1	1	1	1	1	yafP	orf, hypothetical protein
b0235	1	1	1	1	1	1	1	1	1	1	1	1	1	0	1	0	1	1	1	1	1	1	1	1	1	1	1	1	1	1	1	1	1	1	1	1	1	1	1	0	1	1	1	b0235	orf, hypothetical protein
b0241	0	1	1	1	1	1	1	1	1	1	1	1	1	1	1	1	1	1	1	1	1	1	1	1	1	1	1	1	1	1	1	1	1	1	1	1	1	1	1	1	1	1	1	phoE	outer membrane pore protein E (E,Ic,NmpAB)
b0245	0	0	0	0	0	0	0	0	0	0	0	0	0	0	0	0	0	0	0	0	0	0	0	0	0	0	0	0	0	0	0	0	0	0	0	0	0	0	0	0	0	0	0	b0245	orf, hypothetical protein
b0246	0	0	0	1	1	0	0	0	0	0	0	0	0	0	0	1	1	0	0	0	1	0	1	1	1	1	1	1	1	1	1	1	1	0	0	0	0	1	1	0	0	0	0	yafW	orf, hypothetical protein
b0247	1	0	0	0	1	0	0	1	1	0	0	0	0	0	0	0	0	0	0	0	0	0	0	0	0	0	0	0	0	0	0	0	0	0	1	0	0	0	0	0	0	0	0	ykfG	putative DNA repair protein
b0248	0	0	0	0	0	0	0	0	0	0	0	0	0	0	0	0	0	0	0	0	0	0	0	0	0	0	0	0	0	0	0	0	0	0	0	0	0	0	0	0	0	0	0	yafX	orf, hypothetical protein
b0249	0	0	0	0	0	0	0	0	0	0	0	0	0	0	0	0	0	0	0	0	0	0	0	0	0	0	0	0	0	0	0	0	0	0	0	0	0	0	0	0	0	0	0	ykfF	orf, hypothetical protein
b0250	0	0	0	0	0	0	0	0	0	0	0	0	0	0	0	0	0	0	0	0	0	0	0	0	0	0	0	0	0	0	0	0	0	0	0	0	0	0	0	0	0	0	0	ykfB	orf, hypothetical protein
b0251	0	0	0	0	0	0	0	0	0	0	0	0	0	0	0	0	0	0	0	0	0	0	0	0	0	0	0	0	0	0	0	0	0	0	0	0	0	0	0	0	0	0	0	yafY	orf, hypothetical protein
b0252	0	0	0	0	0	0	0	0	0	0	0	0	0	0	0	0	0	0	0	0	0	0	0	0	0	0	0	0	0	0	0	0	0	0	0	0	0	0	0	0	0	0	0	yafZ	orf, hypothetical protein
b0253	0	0	0	0	0	0	0	0	0	0	0	0	0	0	0	0	0	0	0	0	0	0	0	0	0	0	0	0	0	0	0	0	0	0	0	0	0	0	0	0	0	0	0	ykfA	putative GTP-binding protein
b0254	0	0	0	0	0	0	0	0	0	0	0	0	0	0	0	0	0	0	0	0	0	0	0	0	0	0	0	0	0	0	0	0	0	0	0	0	0	0	0	0	0	0	0	perR	putative transcriptional regulator LYSR-type
b0257	1	0	1	0	1	1	1	1	1	1	1	1	1	1	1	1	1	1	1	1	1	1	1	1	1	1	1	1	1	1	1	1	1	1	1	1	1	1	1	1	1	1	1	b0257	putative transposase
b0258	0	0	0	0	0	1	0	1	0	0	1	1	1	0	0	1	1	0	0	0	1	1	1	1	1	0	0	0	0	0	0	1	0	0	1	1	0	0	0	0	0	0	0	ykfC	orf, hypothetical protein
b0260	0	0	0	0	0	0	0	0	0	0	0	0	0	0	0	0	0	0	0	0	0	0	0	0	0	0	0	0	0	0	0	0	0	0	0	0	0	0	0	0	0	0	0	ykfD	putative amino acid/amine transport protein
b0261	0	0	0	0	0	0	0	0	0	0	0	0	0	0	0	0	0	0	0	0	0	0	0	0	0	0	0	0	0	0	0	0	0	0	0	0	0	0	0	0	0	0	0	yagD	putative enzyme
b0262	0	0	0	0	0	0	0	0	0	0	0	0	0	0	0	0	0	0	0	0	0	0	0	0	0	0	0	0	0	0	0	0	0	0	0	0	0	0	0	0	0	0	0	afuC	putative ATP-binding component of a transport system
b0263	0	0	0	0	0	0	0	0	0	0	0	0	0	0	0	0	0	0	0	0	0	0	0	0	0	0	0	0	0	0	0	0	0	0	0	0	0	0	0	0	0	0	0	b0263	putative transport system permease protein
b0267	0	0	0	0	0	0	0	0	0	0	0	0	0	0	0	0	0	0	0	0	0	0	0	0	0	0	0	0	0	0	0	0	0	0	0	0	0	0	0	0	0	0	0	yagA	orf, hypothetical protein
b0268	0	0	0	0	0	0	0	0	0	0	0	0	0	0	0	0	0	0	0	0	0	0	0	0	0	0	0	0	0	0	0	0	0	0	0	0	0	0	0	0	0	0	0	yagE	putative lyase/synthase
b0269	0	0	0	1	0	0	0	1	0	0	0	1	0	0	1	0	1	1	1	0	0	0	0	0	0	1	0	1	1	0	0	0	0	0	0	0	0	0	0	0	0	0	0	yagF	putative dehydratase
b0270	0	1	0	0	0	1	0	0	0	0	0	1	0	0	0	0	1	0	0	0	1	0	0	1	0	0	0	0	0	0	0	0	0	0	0	0	0	0	0	0	0	0	0	yagG	putative permease
b0271	0	1	0	1	1	1	1	1	1	1	1	1	1	0	1	1	1	1	1	1	1	1	1	1	1	1	1	1	1	1	1	1	1	0	0	1	1	1	1	1	1	1	1	yagH	putative beta-xylosidase (EC 3.2.1.37)
b0272	0	1	0	0	0	1	1	0	1	0	1	1	1	0	1	1	1	0	0	0	1	0	0	0	0	1	0	1	1	1	1	1	0	0	0	0	0	0	0	0	0	0	0	yagI	putative regulator
b0273	1	1	1	0	0	1	0	0	0	0	1	1	0	1	0	1	1	0	0	0	1	1	1	1	0	0	0	0	0	0	0	1	0	1	1	0	0	0	0	0	0	0	0	argF	ornithine carbamoyltransferase 2, chain F
b0276	0	1	0	0	1	1	1	1	1	1	1	1	0	0	0	0	0	1	1	1	1	0	0	1	1	0	0	0	0	1	1	1	1	0	0	0	0	0	0	0	0	0	0	yagJ	orf, hypothetical protein
b0277	0	0	0	0	0	0	0	0	0	0	0	0	0	0	0	0	0	0	0	0	0	0	0	0	0	0	0	0	0	0	0	0	0	0	0	0	0	0	0	0	0	0	0	yagK	orf, hypothetical protein
b0278	0	0	0	0	0	0	0	0	0	0	0	0	0	0	0	0	0	0	0	0	0	0	0	0	0	0	0	0	0	0	0	0	0	0	0	0	0	0	0	0	0	0	0	yagL	DNA-binding protein
b0279	0	0	0	0	0	0	0	0	0	0	0	0	0	0	0	0	0	0	0	0	0	0	0	0	0	0	0	0	0	0	0	0	0	0	0	0	0	0	0	0	0	0	0	yagM	orf, hypothetical protein
b0280	0	0	0	0	0	0	0	0	0	0	0	0	0	0	0	0	0	0	0	0	0	0	0	0	0	0	0	0	0	0	0	0	0	0	0	0	0	0	0	0	0	0	0	yagN	orf, hypothetical protein
b0281	0	0	0	0	0	0	0	0	0	0	0	0	0	0	0	0	0	0	0	0	0	0	0	0	0	0	0	0	0	0	0	0	0	0	0	0	0	0	0	0	0	0	0	intF	putative phage integrase
b0282	0	0	0	0	0	0	0	0	0	0	0	0	0	0	0	0	0	0	0	0	0	0	0	0	0	0	0	0	0	0	0	0	0	0	0	0	0	0	0	0	0	0	0	yagP	putative transcriptional regulator LYSR-type
b0283	0	1	0	1	0	1	0	0	0	1	1	1	1	0	0	1	1	0	0	0	1	0	1	1	1	0	0	1	0	0	1	1	1	0	0	0	0	0	0	0	0	0	0	yagQ	orf, hypothetical protein
b0284	0	0	0	0	0	0	0	0	0	0	0	0	0	0	0	0	0	0	0	0	0	0	1	1	0	0	0	0	0	0	0	1	0	0	0	0	0	0	0	0	0	0	0	yagR	orf, hypothetical protein
b0285	0	0	0	0	0	0	0	0	0	0	0	0	0	0	0	0	0	0	0	0	0	0	0	0	0	0	0	0	0	0	0	0	0	0	0	0	0	0	0	0	0	0	0	yagS	orf, hypothetical protein
b0286	0	0	0	0	0	0	0	0	0	0	0	0	0	0	0	0	0	0	0	0	0	0	0	0	0	0	0	0	0	0	0	0	0	0	0	0	0	0	0	0	0	0	0	yagT	putative xanthine dehydrogenase (EC 1.1.1.20)
b0287	0	1	0	0	0	0	0	0	0	0	0	0	0	0	0	0	0	0	0	0	0	0	0	0	0	0	0	0	0	0	0	1	0	0	0	0	0	0	0	0	0	0	0	yagU	orf, hypothetical protein
b0288	0	1	0	0	0	1	1	1	1	1	1	1	1	1	1	1	1	0	0	1	1	1	1	0	0	0	0	0	0	0	0	0	0	0	0	0	0	0	0	0	0	0	0	ykgJ	putative ferredoxin
b0289	0	0	0	0	0	0	0	0	0	0	0	0	0	0	0	0	0	0	0	0	0	0	0	0	0	0	0	0	0	0	0	0	0	0	0	0	0	0	0	0	0	0	0	yagV	orf, hypothetical protein
b0290	0	0	0	0	0	1	0	0	0	1	0	0	0	1	1	1	0	1	1	1	0	1	1	0	0	0	0	0	0	0	0	0	0	0	0	0	0	0	0	0	0	0	0	yagW	putative receptor
b0291	0	1	0	0	1	1	1	1	1	1	1	1	1	1	0	1	1	1	1	1	1	1	1	1	1	0	1	1	0	0	1	1	1	0	0	1	1	1	1	1	1	1	1	yagX	putative enzyme
b0292	0	0	0	0	0	1	0	0	0	1	0	0	0	1	1	1	0	1	1	1	0	1	1	0	0	0	0	0	0	0	0	0	0	0	0	0	0	0	0	0	0	0	0	yagY	orf, hypothetical protein
b0293	0	0	0	0	0	1	0	0	0	1	0	0	0	1	1	1	0	1	1	1	0	1	1	0	0	0	0	0	0	0	0	0	0	0	0	0	0	0	0	0	0	0	0	yagZ	orf, hypothetical protein
b0294	0	0	0	0	0	1	0	0	0	1	0	0	0	1	1	1	0	1	1	1	0	1	1	0	0	0	0	0	0	0	0	0	0	0	0	0	0	0	0	0	0	0	0	ykgK	putative regulator
b0295	0	0	0	1	1	1	0	0	0	1	0	0	0	1	1	1	1	1	1	1	1	1	1	1	0	1	0	1	1	0	0	1	0	0	0	0	0	1	1	0	0	1	1	ykgL	orf, hypothetical protein
b0296	0	0	0	0	0	0	0	0	0	1	0	0	0	1	0	1	0	1	1	1	0	1	1	1	0	0	0	0	1	1	0	0	0	0	0	0	0	0	0	0	0	0	0	ykgM	putative ribosomal protein
b0297	0	0	0	0	0	0	0	0	0	0	0	0	0	1	1	1	0	1	1	1	0	1	1	1	0	0	1	1	1	0	0	0	0	0	0	0	0	0	0	0	0	0	0	eaeH	attaching and effacing protein, pathogenesis factor
b0300	0	0	0	0	0	1	0	1	1	1	1	1	0	0	1	0	0	1	1	1	1	1	1	1	0	0	1	1	1	0	0	0	0	0	0	0	0	0	0	0	0	0	0	ykgA	putative ARAC-type regulatory protein
b0301	0	1	1	0	0	1	1	1	0	1	0	1	0	0	0	0	0	1	1	1	0	1	1	0	0	0	1	1	1	0	0	0	0	0	0	0	0	0	0	0	0	0	0	ykgB	orf, hypothetical protein
b0302	0	1	1	0	1	1	1	1	0	1	1	1	0	0	0	1	1	1	1	1	1	1	1	1	0	0	1	1	1	0	0	0	0	0	0	0	0	0	0	0	0	0	0	b0302	orf, hypothetical protein
b0303	0	1	1	0	0	1	1	1	0	1	0	1	0	0	0	0	0	1	1	1	0	1	1	1	0	0	1	1	1	0	0	0	0	0	0	0	0	0	0	0	0	0	0	ykgI	orf, hypothetical protein
b0304	0	1	1	0	0	1	1	1	0	1	0	0	0	0	0	0	0	1	1	1	0	1	1	1	0	0	1	1	1	0	0	0	0	0	0	0	0	0	0	0	0	0	0	ykgC	putative oxidoreductase
b0305	0	1	1	0	0	1	1	1	0	1	0	0	0	0	0	0	0	1	1	1	1	1	1	1	0	0	1	1	1	0	0	0	0	0	0	0	0	0	0	0	0	0	0	ykgD	putative ARAC-type regulatory protein
b0306	0	1	1	0	0	1	1	1	0	1	0	1	1	0	1	1	0	1	1	1	1	1	1	1	0	0	1	1	0	0	0	0	1	1	1	1	1	1	1	1	1	1	1	ykgE	putative dehydrogenase subunit
b0307	0	1	1	0	0	1	1	1	0	1	0	1	1	0	1	1	0	1	1	1	1	1	1	1	0	0	1	1	0	0	0	0	1	1	1	1	1	1	1	1	1	1	1	ykgF	orf, hypothetical protein
b0308	0	1	1	0	0	1	1	1	0	1	0	1	1	0	1	1	0	1	1	1	1	1	1	1	0	0	1	1	1	0	0	0	1	1	1	1	1	1	1	1	1	1	1	ykgG	putative transporter
b0309	0	1	1	0	0	1	1	1	0	1	0	1	0	0	0	1	0	0	1	1	0	1	1	0	0	0	1	1	1	0	0	0	0	1	1	0	0	0	0	0	0	0	0	b0309	orf, hypothetical protein
b0310	0	0	0	0	0	0	0	0	0	0	0	0	1	0	0	1	0	0	0	0	0	0	0	0	0	1	1	1	1	0	0	0	1	1	1	1	1	1	1	1	0	1	1	ykgH	orf, hypothetical protein
b0311	0	0	0	0	0	0	0	0	0	0	0	0	0	0	0	0	0	0	0	0	0	0	0	0	0	1	1	1	0	0	0	0	1	1	0	0	0	0	0	0	0	1	1	betA	choline dehydrogenase, a flavoprotein
b0312	0	1	0	0	0	1	1	1	0	1	0	1	1	0	1	1	0	1	1	1	1	1	1	1	0	0	1	1	0	0	0	0	1	1	0	1	1	0	1	0	0	1	1	betB	NAD+-dependent betaine aldehyde dehydrogenase
b0313	0	0	0	0	0	1	1	1	0	1	0	0	0	0	0	0	0	0	1	1	0	1	1	1	0	0	0	1	1	0	0	0	1	1	0	0	0	0	0	0	0	1	1	betI	probably transcriptional repressor of bet genes
b0314	0	0	0	0	0	0	0	0	0	0	0	0	0	0	0	0	0	0	0	0	1	0	0	1	0	0	0	0	0	0	0	1	1	1	0	0	0	0	0	0	0	1	1	betT	high-affinity choline transport
b0315	0	0	0	0	0	0	0	0	0	0	0	0	0	0	0	0	0	0	0	0	0	0	0	0	0	0	0	0	0	0	0	0	0	0	0	0	0	0	0	0	0	0	0	yahA	orf, hypothetical protein
b0316	0	1	0	1	1	1	1	1	1	1	1	1	1	0	1	1	1	1	0	1	1	1	1	1	1	1	1	1	1	1	1	1	1	0	0	0	0	1	1	1	1	1	1	yahB	putative transcriptional regulator LYSR-type

b0317	0	0	0	0	0	0	0	0	0	0	0	0	0	0	0	0	0	0	0	0	0	0	0	0	0	0	0	0	0	0	0	0	0	0	0	0	0	0	0	0	0	0	0	0	yahC	orf, hypothetical protein
b0318	0	0	1	0	0	0	0	0	0	0	0	0	0	0	0	0	0	0	0	0	1	0	0	0	0	0	0	0	0	0	0	0	0	0	0	0	0	0	0	0	0	0	0	0	yahD	putative transcription factor
b0319	0	0	1	0	0	0	0	0	0	0	0	0	0	0	0	0	0	0	0	0	0	0	0	0	0	0	0	0	0	0	0	0	0	0	0	0	0	0	0	0	0	0	0	0	yahE	orf, hypothetical protein
b0320	0	0	1	0	0	1	0	0	0	1	0	1	0	0	0	0	0	0	0	0	1	0	1	1	0	0	0	0	0	0	0	0	0	0	0	0	0	0	0	0	0	0	0	0	yahF	putative oxidoreductase subunit
b0321	0	0	1	0	0	0	0	0	0	0	0	0	0	0	0	0	0	0	0	0	0	0	0	0	0	0	0	0	0	0	0	0	0	0	0	0	0	0	0	0	0	0	0	0	yahG	orf, hypothetical protein
b0322	0	1	1	1	1	1	1	1	1	1	1	1	0	1	1	1	1	1	1	1	1	1	1	1	1	1	1	1	1	1	1	1	1	1	1	0	1	1	1	1	1	1	1	1	yahH	orf, hypothetical protein
b0323	0	0	1	0	0	0	0	0	0	0	0	0	0	0	0	0	0	0	0	0	0	0	0	0	0	0	0	0	0	0	0	0	0	0	0	0	0	0	0	0	0	0	0	0	yahI	putative kinase (EC 2.7.2.2).
b0324	0	0	1	0	0	0	0	0	0	0	0	0	0	0	0	0	0	0	0	0	0	0	0	0	0	0	0	0	0	0	0	0	0	0	0	0	0	0	0	0	0	0	0	0	yahJ	putative deaminase
b0325	0	0	1	0	0	0	0	0	0	0	0	0	0	0	0	0	0	0	0	0	0	0	0	0	0	0	0	0	0	0	0	0	0	0	0	0	0	0	0	0	0	0	0	0	yahK	putative oxidoreductase
b0326	0	0	1	0	0	0	0	0	0	0	0	0	0	0	0	0	0	0	0	0	0	0	0	0	0	0	0	0	0	0	0	0	0	0	0	0	0	0	0	0	0	0	0	0	yahL	orf, hypothetical protein
b0327	0	0	1	0	0	0	0	0	0	0	0	0	0	0	0	0	0	0	0	0	0	0	0	0	0	0	0	0	0	0	0	0	0	0	0	0	0	0	0	0	0	0	0	0	yahM	orf, hypothetical protein
b0328	0	0	1	0	0	0	0	0	0	0	0	0	0	0	0	0	0	0	0	0	0	0	0	0	0	0	0	0	0	0	1	0	0	0	0	0	0	0	0	0	0	0	0	0	yahN	putative cytochrome subunit of dehydrogenase
b0329	0	1	0	0	0	0	0	0	0	0	0	0	0	0	0	0	0	0	0	0	0	0	0	0	0	0	0	0	0	0	1	0	0	0	0	0	0	0	0	0	0	0	0	0	yahO	orf, hypothetical protein
b0330	0	1	0	0	1	1	1	1	1	1	1	1	0	0	1	1	1	1	0	1	1	1	1	1	1	1	1	1	1	1	1	1	1	0	0	0	1	1	1	1	1	1	1	1	prpR	regulator for prp operon
b0331	0	1	0	0	0	0	0	0	0	0	0	0	0	0	0	0	0	0	0	0	0	0	0	0	0	0	0	0	0	1	1	0	0	0	0	0	0	0	0	0	0	0	0	0	prpB	putative phosphonomutase 2
b0332	1	1	0	0	0	0	0	0	0	0	0	0	0	1	0	0	0	0	0	0	0	0	0	0	0	0	0	0	0	1	1	0	0	0	0	0	0	0	0	0	0	0	0	0	b0332	orf, hypothetical protein
b0333	0	1	0	0	0	0	0	0	0	0	0	0	0	0	0	0	0	0	0	0	0	0	0	0	0	0	0	0	0	1	1	0	0	0	0	0	0	0	0	0	0	0	0	0	prpC	putative citrate synthase; propionate metabolism?
b0334	0	1	0	0	0	0	0	0	0	0	0	0	0	0	0	0	0	0	0	0	0	0	0	0	0	0	0	0	0	1	1	0	0	0	0	0	0	0	0	0	0	0	0	0	prpD	orf, hypothetical protein
b0335	0	1	0	0	1	1	0	0	0	0	1	1	1	0	0	1	1	0	0	0	1	1	1	1	0	0	0	1	1	1	1	1	0	0	0	0	0	0	0	0	0	1	0	prpE	putative propionyl-CoA synthetase
b0336	0	0	0	0	0	0	0	0	0	0	0	0	0	0	0	0	0	0	0	0	0	0	0	0	0	0	0	0	0	1	1	0	0	0	0	0	0	0	0	0	0	0	0	0	codB	cytosine permease/transport
b0337	0	0	0	0	0	0	0	0	0	0	0	0	0	0	0	0	0	0	0	0	0	0	0	0	0	0	0	0	0	1	1	0	0	0	0	0	0	0	0	0	0	0	0	0	codA	cytosine deaminase
b0338	0	0	0	0	0	0	0	0	0	0	0	0	0	0	0	0	0	0	0	0	0	0	0	0	0	0	0	0	0	1	1	0	0	0	0	0	0	0	0	0	0	0	0	0	cynR	cyn operon positive regulator
b0339	0	0	0	0	0	0	0	0	0	0	0	0	0	0	0	0	0	0	0	0	0	0	0	0	0	0	0	0	0	1	1	0	0	0	0	0	0	0	0	0	0	0	0	0	cynT	carbonic anhydrase
b0340	0	0	0	0	0	0	0	0	0	0	0	0	0	0	0	0	0	0	0	0	0	0	0	0	0	0	0	0	0	1	1	0	0	0	0	0	0	0	0	0	0	0	0	0	cynS	cyanate aminohydrolase, cyanase
b0341	0	1	0	1	1	1	1	1	1	1	1	1	1	0	1	1	1	1	1	1	1	1	1	1	0	1	0	1	1	1	1	1	0	0	0	1	1	1	1	1	1	1	1	cynX	cyanate transport
b0342	0	0	0	0	0	0	0	0	0	0	0	0	0	0	0	0	0	0	0	0	0	0	0	0	0	0	0	0	0	1	1	0	0	0	0	0	0	0	0	0	0	0	0	0	lacA	thiogalactoside acetyltransferase
b0343	0	0	0	0	0	0	0	0	0	0	0	0	0	0	0	0	0	0	0	0	0	0	0	0	0	0	0	0	0	1	1	0	0	0	0	0	0	0	0	0	0	0	0	0	lacY	galactoside permease (M protein)
b0344	1	1	1	0	1	1	1	1	1	0	1	1	1	0	0	1	1	0	0	1	1	0	0	0	1	0	0	0	1	1	1	0	1	0	0	0	0	0	1	1	1	1	0	lacZ	beta-D-galactosidase
b0345	1	0	1	0	1	0	0	0	0	0	0	0	0	0	0	0	0	0	0	0	0	0	0	0	0	0	0	0	0	0	1	0	0	0	0	0	0	0	0	0	0	0	0	0	lacI	transcriptional repressor of the lac operon
b0346	0	0	1	0	1	0	0	0	0	0	0	0	0	0	0	0	0	0	0	0	0	0	0	0	0	0	0	0	0	0	1	0	0	0	0	0	0	0	0	0	0	0	0	0	mhpR	transcriptional regulator for mhp operon
b0347	0	0	1	0	1	0	0	0	0	0	0	0	0	0	0	0	0	0	0	0	0	0	0	0	0	0	0	0	0	0	1	0	0	0	0	0	0	0	0	0	0	0	0	0	mhpA	3-(3-hydroxyphenyl)propionate hydroxylase
b0348	0	0	1	0	1	0	0	0	0	0	0	0	0	0	0	0	0	0	0	0	0	0	0	0	0	0	0	0	0	0	1	0	0	0	0	0	0	0	0	0	0	0	0	0	mhpB	2,3-dihydroxyphenylpropionate 1,2-dioxygenase
b0349	0	0	1	0	1	0	0	0	0	0	0	0	0	0	0	0	0	0	0	0	0	0	0	0	0	0	0	0	0	0	1	0	0	0	0	0	0	0	0	0	0	0	0	0	mhpC	2-hydroxy-6-ketonona-2,4-dienedioic acid hydrolase
b0350	0	0	1	0	1	0	0	0	0	0	0	0	0	0	0	0	0	0	0	0	0	0	0	0	0	0	0	0	0	0	1	0	0	0	0	0	0	0	0	0	0	0	0	0	mhpD	2-keto-4-pentenoate hydratase
b0351	0	0	1	0	1	0	0	0	0	0	0	0	0	0	0	0	1	0	0	0	1	0	0	0	0	0	0	0	0	0	1	1	0	0	0	0	0	0	0	0	0	0	0	mhpF	acetaldehyde dehydrogenase
b0352	0	0	1	0	1	0	0	0	0	0	0	0	0	0	0	0	0	0	0	0	0	0	0	0	0	0	0	0	0	0	1	0	0	0	0	0	0	0	0	0	0	0	0	0	mhpE	4-hydroxy-2-ketovalerate aldolase
b0353	0	0	1	0	1	0	0	0	0	0	0	0	0	0	0	0	0	0	0	0	0	0	0	0	0	0	0	0	0	1	1	0	0	0	0	0	0	0	0	0	0	0	0	0	mhpT	putative transport protein
b0354	0	1	1	0	1	0	0	0	0	0	0	0	0	0	0	0	0	0	0	0	0	0	0	0	0	0	0	0	0	1	1	0	0	0	0	0	0	0	0	0	0	0	0	0	yaiL	nucleoprotein/polynucleotide-associated enzyme
b0355	0	1	1	0	1	1	1	1	1	1	1	1	1	0	0	0	0	0	0	0	0	0	0	0	0	0	0	0	0	1	1	0	0	0	0	0	0	0	0	0	0	0	0	0	yaiM	putative esterase (EC 3.1.1.1)
b0356	0	1	1	1	1	1	1	1	1	1	1	1	1	0	0	1	1	0	0	1	1	1	1	1	0	0	0	0	1	1	1	1	0	0	0	0	0	0	0	0	0	0	0	adhC	alcohol dehydrogenase class III; formaldehyde dehydrogenase, glutathione-dependent
b0357	0	1	1	0	1	1	1	1	1	1	1	1	1	0	0	0	0	0	0	0	0	0	0	0	0	0	0	0	0	1	1	0	0	0	0	0	0	0	0	0	0	0	0	0	yaiN	putative alpha helix chain
b0358	0	1	1	1	1	1	1	1	1	1	1	1	1	0	0	0	0	0	0	0	1	0	0	0	0	0	0	0	0	1	1	1	0	0	0	0	0	0	0	0	0	0	0	yaiO	orf, hypothetical protein
b0359	0	0	1	1	1	1	1	1	1	1	1	1	1	0	0	0	0	0	0	0	0	0	0	0	0	0	0	0	0	1	1	1	0	0	0	0	0	0	0	0	0	0	0	b0359	putative transferase
b0362	0	0	1	1	1	1	1	1	1	1	1	1	1	0	0	0	0	0	0	1	0	1	1	1	0	0	0	0	0	1	1	1	0	0	0	0	0	0	0	0	0	0	0	b0362	orf, hypothetical protein
b0363	0	0	1	1	1	1	1	1	0	1	1	1	1	0	0	0	0	1	1	1	1	1	1	1	0	0	0	0	0	1	1	1	0	0	0	0	0	0	0	0	0	0	0	yaiP	polysaccharide metabolism
b0364	0	0	1	1	1	1	1	1	0	1	1	1	1	0	0	0	0	1	1	1	1	1	1	1	0	0	0	0	0	1	1	1	0	0	0	0	0	0	0	0	0	0	0	yaiS	orf, hypothetical protein
b0365	1	1	1	1	1	1	1	1	0	1	1	1	1	1	1	1	1	1	1	1	1	1	1	1	0	0	0	0	0	1	1	1	1	1	1	1	1	1	1	0	1	1	1	tauA	taurine transport system periplasmic protein
b0366	1	1	1	1	1	1	1	1	0	1	1	1	1	1	1	1	1	1	1	1	1	1	1	1	0	0	0	0	0	1	1	1	1	1	1	1	1	1	1	1	1	1	1	tauB	taurine ATP-binding component of a transport system
b0367	1	1	1	1	1	0	0	1	0	1	1	1	0	1	1	1	1	1	1	1	1	1	1	1	0	0	0	0	0	0	1	1	0	1	1	1	1	1	1	1	0	1	1	tauC	taurine transport system permease protein
b0368	1	1	1	1	1	1	1	1	0	1	1	1	0	1	1	1	1	1	1	1	1	1	1	1	0	0	0	0	0	1	1	1	1	1	1	1	1	1	1	1	1	1	1	tauD	taurine dioxygenase, 2-oxoglutarate-dependent
b0370	0	1	1	0	1	1	1	1	1	1	1	1	1	1	1	1	1	1	1	1	1	1	1	1	1	1	1	0	1	1	1	1	0	1	1	1	1	1	1	1	0	1	1	b0370	orf, hypothetical protein
b0371	0	0	0	0	0	0	0	0	0	0	0	0	0	0	0	0	0	0	0	0	0	0	0	0	0	0	0	0	0	0	0	1	0	0	0	0	0	0	0	0	0	0	0	yaiT	orf, hypothetical protein
b0374	1	1	1	1	1	1	1	1	1	1	1	1	1	1	1	1	1	1	1	1	0	1	1	1	1	1	1	1	1	1	1	1	1	1	1	1	1	1	1	0	1	1	1	yaiU	putative flagellin structural protein
b0375	1	1	1	1	1	1	1	1	1	1	1	1	1	1	1	1	1	1	1	1	1	1	1	1	1	1	1	1	1	1	1	1	1	0	0	1	1	1	1	1	1	1	1	yaiV	orf, hypothetical protein
b0376	1	1	1	1	1	1	1	1	1	1	1	1	1	1	1	1	1	1	1	1	1	1	1	1	1	1	1	1	1	1	1	1	1	1	1	1	1	1	1	0	1	1	1	yaiH	putative enzyme
b0392	1	0	1	1	1	1	0	1	0	1	1	1	0	1	1	1	1	0	1	1	1	1	1	1	1	1	1	1	1	1	1	1	1	1	1	1	1	1	1	1	1	1	1	b0392	orf, hypothetical protein
b0417	1	1	1	1	1	1	1	1	1	1	1	0	1	1	1	1	1	1	1	1	1	1	1	1	1	1	1	1	1	1	1	1	1	1	1	1	1	1	1	1	1	1	1	thiL	thiamin-monophosphate kinase
b0427	1	1	1	1	1	1	1	1	1	1	1	1	1	1	0	1	1	1	1	1	1	1	1	1	1	1	1	1	1	1	1	1	1	1	0	0	0	0	0	0	1	0	0	yajR	putative transport protein
b0428	0	1	1	1	1	1	1	1	1	1	1	1	1	1	1	1	1	1	1	1	1	1	1	1	1	1	1	1	1	1	0	1	1	1	1	1	1	1	1	1	1	1	1	cyoE	protoheme IX farnesyltransferase (haeme O biosynthesis)
b0429	0	1	1	1	1	1	1	1	1	1	1	1	1	1	1	1	1	1	1	1	1	1	1	1	1	1	1	1	1	1	1	1	1	1	1	1	1	1	1	1	1	1	1	cyoD	cytochrome o ubiquinol oxidase subunit IV
b0430	0	1	1	1	1	1	1	1	1	1	1	1	1	1	1	1	1	1	1	1	1	1	1	1	1	1	1	1	1	1	1	1	1	1	1	1	1	1	1	1	1	1	1	cyoC	cytochrome o ubiquinol oxidase subunit III
b0431	0	1	1	1	1	1	1	1	1	1	1	1	1	1	1	1	1	1	1	1	1	1	1	1	1	1	1	1	1	1	1	1	1	1	1	1	1	1	1	1	1	1	1	cyoB	cytochrome o ubiquinol oxidase subunit I
b0432	0	1	1	1	1	1	1	1	1	1	1	1	1	1	1	1	1	1	1	1	1	1	1	1	1	1	1	1	1	1	1	1	1	1	1	1	1	1	1	1	0	1	1	cyoA	cytochrome o ubiquinol oxidase subunit II
b0433	1	1	1	1	1	1	1	1	1	1	1	1	1	1	1	1	1	1	1	1	1	1	1	1	1	1	1	1	1	1	1	1	1	1	1	1	1	1	1	0	1	1	1	ampG	regulates beta-lactamase synthesis
b0442	1	1	1	1	1	0	0	0	0	1	1	1	0	1	1	1	1	0	1	1	1	1	1	1	1	1	0	1	1	1	1	1	1	1	1	1	1	1	1	1	1	1	1	ybaV	orf, hypothetical protein
b0472	1	1	1	1	1	1	1	1	1	1	1	1	1	1	1	1	1	1	1	1	1	1	1	1	1	1	1	1	1	1	1	1	1	1	1	1	1	1	1	0	1	1	1	recR	recombination and repair
b0483	1	0	1	0	1	1	1	1	1	1	1	1	1	1	1	1	1	1	1	1	1	1	1	1	1	1	1	1	1	1	1	1	1	1	1	1	1	1	1	1	1	1	1	ybaQ	orf, hypothetical protein
b0494	1	1	1	0	1	1	1	1	1	1	1	1	1	1	1	1	1	1	1	1	1	1	1	1	1	1	1	1	1	1	1	1	1	1	1	1	1	1	1	1	1	1	1	tesA	acyl-CoA thioesterase I; also functions as protease I
b0495	1	1	1	0	1	1	1	1	1	1	1	1	1	1	1	1	1	1	1	1	1	1	1	1	1	1	1	1	1	1	1	1	1	1	1	1	1	1	1	1	1	1	1	ybbA	putative ATP-binding component of a transport system
b0496	1	1	1	0	1	1	1	1	1	1	1	1	1	1	1	1	1	1	1	1	1	1	1	1	1	1	1	1	1	1	1	1	1	1	1	1	1	1	1	1	1	1	1	ybbP	putative oxidoreductase
b0497	1	1	1	1	1	1	1	1	1	1	1	1	0	1	1	1	1	1	1	1	1	1	1	1	1	1	1	1	1	0	1	1	0	0	0	1	1	1	1	1	1	1	1	rhsD	rhsD protein in rhs element
b0498	0	0	0	0	0	0	0	0	0	0	0	0	0	0	0	0	0	0	0	0	0	0	0	0	0	0	0	0	0	0	0	0	0	0	0	0	0	0	0	0	0	0	0	ybbC	orf, hypothetical protein
b0499	0	0	0	0	1	1	1	1	1	0	1	1	1	1	1	1	1	0	1	1	1	1	1	1	0	0	0	0	0	0	0	0	0	0	0	0	0	0	0	0	0	0	0	b0499	orf, hypothetical protein
b0500	0	0	0	0	1	1	1	1	1	1	1	1	1	1	1	1	1	1	1	1	1	1	1	1	0	0	0	0	0	0	0	0	0	0	0	0	0	0	0	0	0	0	0	ybbD	orf, hypothetical protein
b0501	1	0	0	0	1	1	1	1	1	1	1	1	1	1	1	1	1	1	1	1	1	1	1	1	0	0	0	0	0	0	0	0	0	0	0	0	0	0	0	0	0	0	0	b0501	orf, hypothetical protein
b0502	1	0	0	0	1	1	1	1	1	1	1	1	1	1	1	1	1	1	1	1	1	1	1	1	0	0	0	0	0	0	0	0	0	0	0	0	0	0	0	0	0	0	0	b0502	orf, hypothetical protein
b0503	1	1	1	0	1	1	1	1	1	1	1	1	1	1	1	1	1	1	1	1	1	1	1	1	1	1	1	1	1	1	1	1	1	1	1	1	1	1	1	1	1	1	1	ybbB	putative capsule anchoring protein
b0504	1	0	1	0	1	1	1	1	1	1	1	1	1	1	1	1	1	1	1	1	1	1	1	1	1	1	1	1	1	1	1	1	1	1	1	1	1	1	1	1	1	1	1	ybbS	putative transcriptional regulator LYSR-type
b0505	1	1	0	0	1	1	1	1	1	1	1	1	1	1	1	1	1	1	1	1	1	1	1	1	1	1	1	1	1	1	1	1	1	1	1	1	1	1	1	1	1	1	1	ybbT	orf, hypothetical protein
b0506	1	0	0	0	1	1	1	1	1	1	1	1	1	1	1	1	1	1	1	1	1	1	1	1	1	1	1	1	1	1	1	1	1	1	1	1	1	1	1	1	1	1	1	ybbU	putative regulator
b0507	1	0	0	0	1	1	1	1	1	1	1	1	1	1	1	1	1	1	1	1	1	1	1	1	1	0	0	1	1	0	0	1	1	1	1	1	1	1	1	1	1	1	1	gcl	glyoxylate carboligase
b0508	0	0	1	0	0	0	0	0	0	0	0	0	0	0	0	0	0	0	0	0	0	0	0	0	0	1	1	1	1	1	1	1	1	0	0	0	0	0	0	0	0	1	1	gip	glyoxylate-induced protein
b0509	0	0	0	0	0	0	0	0	0	0	0	1	0	0	0	0	0	0	0	0	1	0	0	0	0	1	1	1	1	1	1	1	0	0	0	0	0	0	0	0	0	1	1	ybbQ	putative oxidoreductase
b0510	0	0	0	0	0	0	0	0	0	0	0	0	0	0	0	0	0	0	0	0	0	0	0	0	0	1	1	1	1	1	1	1	0	0	0	0	0	0	0	0	0	0	0	ybbV	orf, hypothetical protein
b0511	0	0	0	0	1	0	0	0	0	0	0	0	0	0	0	0	0	0	0	0	0	0	0	0	0	1	1	1	1	1	1	1	0	0	0	0	0	0	0	0	0	0	0	ybbW	putative transport protein
b0512	0	0	1	0	1	0	0	0	0	0	0	0	0	0	0	0	0	0	0	0	0	0	0	0	0	1	1	1	1	1	1	1	0	0	0	0	0	0	0	0	0	0	0	ybbX	putative hydrolase
b0513	0	0	1	0	1	0	0	0	0	0	0	0	0	0	0	0	0	0	0	0	0	0	0	0	0	1	1	1	1	1	1	1	0	0	0	0	0	0	0	0	0	0	0	ybbY	putative transport
b0514	0	0	0	0	1	0	0	0	0	0	0	0	0	0	0	0	0	0	0	0	0	0	0	0	0	1	1	1	1	1	1	1	0	0	0	0	0	0	0	0	0	1	1	ybbZ	orf, hypothetical protein
b0515	0	0	0	0	1	0	0	0	0	0	0	0	0	0	0	0	0	0	0	0	0	0	0	0	0	1	1	1	1	1	1	1	1	1	1	1	1	1	1	0	1	1	1	ylbA	orf, hypothetical protein
b0516	0	0	0	0	1	0	0	0	0	0	0	0	0	0	0	0	0	0	0	0	0	0	0	0	0	1	1	1	1	1	1	1	1	1	1	1	1	1	1	1	1	1	1	ylbB	putative hydantoin utilization protein
b0517	0	1	0	0	1	0	0	0	0	0	0	0	0	0	0	0	1	0	0	0	1	1	1	1	0	1	1	1	1	1	1	1	0	0	0	0	0	0	0	0	0	1	1	ylbC	putative malate dehydrogenase (EC 1.1.1.37)
b0518	1	0	0	0	1	1	0	1	1	1	1	1	0	1	1	1	1	1	1	1	1	1	1	1	0	0	0	0	1	1	1	1	0	0	0	0	0	0	0	0	0	1	1	fdrA	involved in protein transport; multicopy suppressor of dominant negative ftsH mutants
b0519	1	0	1	1	1	1	1	1	1	1	1	1	1	1	1	1	1	1	1	1	1	1	1	1	0	1	0	1	1	1	1	1	0	0	0	0	0	0	0	0	0	1	1	ylbE	orf, hypothetical protein
b0520	1	0	1	1	1	1	1	1	1	1	1	1	1	1	1	1	1	1	1	1	1	1	1	1	0	1	1	1	1	1	1	1	1	1	1	1	1	1	1	1	1	1	1	ylbF	putative carboxylase
b0530	0	1	0	1	0	0	0	0	0	0	0	0	0	0	0	0	0	1	1	0	0	0	0	0	1	1	1	1	0	1	1	1	0	1	1	1	1	1	1	1	1	0	0	sfmA	putative fimbrial-like protein
b0531	1	1	0	0	0	0	0	0	0	0	0	0	0	0	0	0	0	1	1	0	0	0	0	0	1	1	1	1	0	1	1	1	0	1	1	1	1	1	1	1	1	0	0	sfmC	putative chaperone
b0532	0	0	1	0	1	0	0	0	0	0	0	0	0	0	0	0	0	1	1	0	0	0	0	0	1	1	1	1	0	1	1	1	0	0	0	0	0	0	0	0	0	1	1	sfmD	putative outer membrane protein, export function
b0533	0	0	1	0	1	0	0	0	0	0	0	0	0	0	0	0	1	1	1	0	0	0	0	0	1	1	1	1	1	1	1	1	1	1	0	0	0	0	0	0	0	1	1	sfmH	involved in fimbrial asembly
b0534	0	0	1	0	1	0	0	0	0	0	0	0	0	0	0	0	1	1	0	0	0	0	0	0	1	1	1	1	1	1	1	1	1	1	0	0	0	0	0	0	0	1	1	sfmF	putative fimbrial-like protein
b0535	0	0	1	0	1	1	1	1	1	0	1	1	1	1	1	1	1	1	1	1	1	1	1	1	1	1	1	1	1	1	1	1	1	1	0	0	0	0	0	0	0	1	1	fimZ	fimbrial Z protein; probable signal transducer
b0537	0	1	0	0	1	0	0	0	0	0	0	0	0	1	0	1	1	0	0	0	0	0	0	0	0	0	0	0	0	0	0	0	0	0	1	0	0	0	0	0	0	0	0	intD	prophage DLP12 integrase
b0538	1	0	0	1	0	0	0	0	0	0	0	0	0	0	0	0	0	0	0	0	0	0	0	0	0	0	0	0	1	0	1	1	0	0	0	0	1	0	0	1	0	0	0	b0538	putative sensory transduction regulator
b0539	1	0	0	1	0	0	0	0	0	0	0	0	0	0	0	0	0	0	0	0	0	0	0	0	0	0	0	0	1	0	1	1	0	0	0	0	1	0	0	1	0	0	0	b0539	putative exonuclease (EC 3.1.11.3) similar to lambda
b0543	0	1	1	0	0	0	0	0	0	0	0	0	0	0	0	0	1	0	1	1	1	0	1	1	0	0	0	0	0	0	0	0	0	1	1	1	1	1	1	1	1	0	0	emrE	methylviologen resistance
b0544	0	0	0	0	0	0	0	0	0	0	0	0	0	0	0	0	0	0	0	0	0	0	0	0	0	0	0	0	0	0	0	0	0	0	0	0	0	0	0	0	0	0	0	ybcK	orf, hypothetical protein
b0545	0	1	1	1	0	0	0	0	0	0	0	0	0	0	0	0	1	0	1	1	1	1	1	1	0	0	0	0	0	0	0	0	1	1	1	1	1	1	1	1	1	0	0	ybcL	orf, hypothetical protein

b0546	0	0	1	0	0	0	0	0	0	0	0	0	0	0	0	0	1	1	1	1	1	1	1	1	0	0	0	0	0	0	0	0	1	1	1	1	1	1	1	1	1	0	0	ybcM	putative ARAC-type regulatory protein
b0547	0	0	0	0	1	0	0	0	0	0	0	0	0	0	0	0	0	0	0	0	0	0	0	0	0	0	0	0	0	0	0	0	0	0	0	0	0	0	0	1	0	0	0	ybcN	orf, hypothetical protein
b0548	0	0	1	0	1	0	0	0	0	0	0	0	0	0	0	0	0	0	0	0	0	0	0	0	0	0	0	0	0	0	0	0	0	0	0	0	0	0	0	1	0	0	0	ninE	similar to phage 82 and lambda proteins
b0549	0	1	1	0	1	0	0	0	0	0	0	0	0	0	0	0	0	0	0	0	0	0	0	0	0	0	0	0	0	0	0	0	0	0	0	0	0	0	0	1	0	0	0	ybcO	orf, hypothetical protein
b0550	0	1	1	0	1	0	0	0	0	0	0	0	0	0	0	0	0	0	0	0	0	0	0	0	0	0	0	0	0	0	0	0	0	0	0	0	0	0	0	1	0	0	0	rus	endodeoxyribonuclease RUS (Holliday junction resolvase)
b0551	0	0	1	1	1	0	0	0	0	0	0	0	0	0	0	0	0	0	0	0	0	0	0	0	0	0	0	0	0	0	0	0	1	1	1	0	0	1	1	1	0	0	0	ybcQ	orf, hypothetical protein
b0553	1	1	1	1	1	1	0	1	1	0	1	1	0	1	0	1	1	0	0	0	1	1	1	1	0	0	0	1	1	0	1	0	1	1	1	1	1	1	1	1	1	1	1	nmpC	outer membrane porin protein; locus of qsr prophage
b0554	1	0	1	1	1	0	0	1	1	1	1	0	0	0	0	0	0	0	0	0	0	1	1	1	0	0	0	1	1	0	1	1	0	1	1	0	0	0	0	0	0	1	1	ybcR	orf, hypothetical protein
b0555	0	0	1	1	1	0	0	1	1	1	1	0	0	0	0	0	1	0	0	1	1	1	1	1	1	1	1	1	1	1	1	1	0	1	0	1	0	1	1	0	1	1	1	ybcS	bacteriophage lambda lysozyme homolog
b0556	1	1	1	1	1	1	1	1	1	1	1	1	0	1	0	0	1	1	1	1	1	1	1	1	1	1	1	1	1	1	1	1	0	1	1	0	0	1	1	1	0	1	1	ybcT	bacteriophage lambda endopeptidase homolog
b0557	0	1	0	1	0	0	0	0	0	1	0	0	0	0	0	0	0	0	0	0	0	0	0	1	0	0	0	1	1	0	1	1	0	0	0	0	0	0	0	0	0	0	0	ybcU	bacteriophage lambda Bor protein homolog
b0558	0	0	1	0	0	0	0	0	0	0	0	0	0	0	0	0	0	0	0	0	0	0	0	0	0	0	0	0	0	0	0	0	0	0	0	0	0	0	0	0	0	0	0	ybcV	putative an envelop protein
b0559	0	0	0	0	0	0	0	0	0	0	0	0	0	0	0	0	0	0	0	0	0	0	0	0	0	0	0	1	1	0	0	0	0	0	0	0	0	0	0	0	0	0	0	ybcW	orf, hypothetical protein
b0560	0	0	1	0	0	0	0	0	0	0	0	0	0	0	0	0	0	0	0	0	0	0	0	0	0	0	0	1	1	0	0	0	0	0	0	0	0	0	0	0	0	0	0	nohB	bacteriophage DNA packaging protein
b0561	0	0	1	0	0	0	0	0	0	0	0	0	0	0	0	0	0	0	0	0	0	0	0	0	0	0	0	1	1	0	0	0	0	0	0	0	0	0	0	0	0	0	0	ybcX	orf, hypothetical protein
b0562	1	0	1	1	1	1	1	1	1	1	1	1	1	1	1	1	1	1	1	1	1	1	1	1	1	1	1	1	1	1	1	1	1	1	1	1	1	1	1	1	1	1	1	ybcY	orf, hypothetical protein
b0563	1	0	1	1	1	0	1	0	0	1	1	1	1	1	1	1	1	1	1	1	1	1	1	1	1	1	1	0	1	1	0	1	1	1	1	1	1	1	1	0	1	1	1	ylcE	orf, hypothetical protein
b0564	0	0	0	0	0	0	0	0	0	0	0	0	0	0	0	0	0	0	0	0	0	0	0	0	0	0	0	0	0	0	0	0	0	0	0	0	0	0	0	0	0	0	0	appY	regulatory protein affecting appA and other genes
b0565	0	0	0	0	0	0	0	0	0	0	0	0	0	0	0	0	0	0	0	0	0	0	0	0	0	0	0	0	0	0	0	0	0	0	0	0	0	0	0	0	0	0	0	ompT	outer membrane protein 3b (a), protease VII
b0566	1	1	1	1	1	0	1	1	1	1	1	1	1	1	1	1	0	1	1	1	1	1	0	1	1	1	1	1	1	1	1	1	1	1	1	1	1	1	1	1	1	1	1	envY	envelope protein; thermoregulation of porin biosynthesis
b0567	1	1	1	1	1	1	0	1	1	1	1	1	1	0	0	1	1	1	1	1	1	1	1	1	1	1	1	1	1	1	1	1	1	1	1	1	1	1	1	1	1	1	1	ybcH	orf, hypothetical protein
b0568	1	1	1	1	1	0	0	0	0	0	0	0	0	0	0	0	0	0	0	0	0	0	0	0	1	1	1	1	1	1	1	1	1	1	1	1	1	1	1	1	1	1	1	nfrA	bacteriophage N4 receptor, outer membrane protein
b0569	1	1	0	1	0	0	0	0	0	0	0	0	0	0	0	0	0	0	0	0	0	0	0	0	1	1	1	1	1	1	1	1	1	1	1	1	1	1	1	1	1	1	1	nfrB	bacteriophage N4 receptor, outer membrane protein
b0570	0	1	1	1	0	0	0	0	0	0	0	0	0	0	0	0	0	0	0	0	0	0	0	0	1	1	1	1	1	1	1	1	1	1	1	1	1	1	1	1	1	1	1	ybcZ	putative 2-component sensor protein
b0571	0	1	1	1	0	0	0	0	0	0	0	0	0	0	0	0	0	0	0	0	1	0	0	0	1	1	1	1	1	1	1	1	1	1	1	1	1	1	1	1	1	1	1	ylcA	putative 2-component transcriptional regulator
b0572	0	1	1	1	0	0	0	0	0	0	0	0	0	0	0	0	0	0	0	0	0	0	0	0	1	1	1	1	1	1	0	1	1	1	1	1	1	1	1	1	1	1	1	ylcB	putative resistance protein
b0573	0	1	1	1	0	0	0	0	0	0	0	0	0	0	0	0	0	0	0	0	0	0	0	0	1	1	1	1	1	1	1	1	1	1	1	1	1	1	1	1	0	1	1	ylcC	orf, hypothetical protein
b0574	0	1	1	1	0	1	0	0	0	1	1	1	0	1	1	1	1	1	1	1	1	1	0	1	1	1	1	1	1	1	1	1	1	1	1	1	1	1	1	1	1	1	1	ylcD	putative resistance protein
b0575	0	1	1	1	0	1	1	1	1	1	1	1	1	1	1	1	1	1	1	1	1	1	1	1	1	1	1	1	1	1	1	1	1	1	1	1	1	1	1	1	1	1	1	ybdE	putative inner membrane component for iron transport
b0581	1	0	1	1	1	1	1	1	1	1	1	1	1	1	1	1	1	1	1	1	1	1	1	1	1	1	1	1	1	1	1	1	1	1	1	1	1	1	0	1	1	1	1	ybdK	orf, hypothetical protein
b0583	1	1	1	1	1	1	1	1	1	1	1	1	1	1	1	1	1	1	1	1	1	1	1	1	0	0	0	0	0	0	0	0	1	1	1	1	1	1	0	1	0	1	1	entD	enterochelin synthetase, component D
b0584	1	1	1	1	1	1	1	1	1	1	1	1	1	1	1	1	1	1	1	1	1	1	1	1	0	0	0	0	0	0	0	0	1	1	1	1	1	1	0	1	0	1	1	fepA	outer membrane receptor for ferric enterobactin (enterochelin) and colicins B and D
b0585	1	1	1	1	1	1	1	1	1	1	1	1	1	1	1	1	1	1	1	1	1	1	1	1	0	0	0	0	0	0	0	0	1	1	1	1	1	1	0	1	0	1	1	fes	enterochelin esterase
b0586	1	1	1	1	1	1	1	1	1	1	1	1	1	1	1	1	1	1	1	1	1	1	1	1	0	0	0	0	0	0	0	0	1	1	1	1	1	1	0	1	0	1	1	entF	ATP-dependent serine activating enzyme
b0587	1	1	1	1	1	1	1	1	1	1	1	1	1	1	1	1	1	1	1	1	1	1	1	1	0	0	0	0	0	0	0	0	1	1	1	1	1	1	0	1	0	1	1	fepE	ferric enterobactin (enterochelin) transport
b0588	1	1	1	1	1	1	1	1	1	1	1	1	1	1	1	1	1	1	1	1	1	1	1	1	0	0	0	0	0	0	0	0	1	1	1	1	1	1	0	1	0	1	1	fepC	ATP-binding component of ferric enterobactin transport
b0589	1	1	1	1	1	1	1	1	1	1	1	1	1	1	1	1	1	1	1	1	1	1	1	1	0	0	0	0	0	0	0	0	1	1	1	1	1	1	0	0	0	1	1	fepG	ferric enterobactin transport protein
b0590	1	1	1	1	1	1	1	1	1	1	1	1	1	1	1	1	1	1	1	1	1	1	1	1	0	0	0	0	0	0	0	0	1	1	1	1	1	1	1	1	0	1	1	fepD	ferric enterobactin (enterochelin) transport
b0591	1	1	1	1	1	1	1	1	1	1	1	1	1	1	1	1	1	1	1	1	1	1	1	1	1	0	0	1	0	0	0	1	1	1	1	1	1	1	0	1	0	1	1	ybdA	putative transport
b0592	1	1	1	1	1	1	1	1	1	1	1	1	1	1	1	1	1	1	1	1	1	1	1	1	1	1	0	1	1	0	1	1	1	1	1	1	1	1	0	0	0	1	1	fepB	ferric enterobactin (enterochelin) binding protein; periplasmic component
b0593	1	1	1	1	1	1	1	1	1	1	1	1	1	1	1	1	1	1	1	1	1	1	1	1	1	1	1	1	1	1	1	1	1	1	1	1	1	1	1	1	0	1	1	entC	isochorismate hydroxymutase 2, enterochelin biosynthesis
b0594	1	1	1	1	1	1	1	1	1	1	1	1	1	1	1	1	1	1	1	1	1	1	1	1	1	1	1	1	1	1	0	0	0	1	1	1	1	1	0	1	0	1	1	entE	2,3-dihydroxybenzoate-AMP ligase
b0596	1	1	1	1	1	1	1	1	1	1	1	1	1	1	1	1	1	1	1	1	1	1	1	1	1	1	1	1	1	1	1	1	1	1	1	1	1	1	0	1	0	1	1	entA	2,3-dihydro-2,3-dihydroxybenzoate dehydrogenase, enterochelin biosynthesis
b0597	1	1	1	1	1	0	1	1	0	1	1	1	1	1	1	1	1	1	1	1	1	1	1	1	1	1	1	1	1	1	0	1	1	1	1	1	1	1	0	1	0	1	1	ybdB	orf, hypothetical protein
b0598	1	1	1	1	1	1	1	1	1	1	1	1	1	1	1	1	1	1	1	1	1	1	1	1	1	1	1	1	1	1	0	1	1	1	1	1	1	1	0	1	0	1	1	cstA	carbon starvation protein
b0598a	1	1	1	1	1	1	1	1	1	1	1	1	1	1	1	1	1	1	1	1	1	1	1	1	1	1	1	1	1	1	1	1	1	1	1	1	1	1	0	1	1	1	1	ybdD	Uncharacterized conserved protein
b0599	1	1	1	1	1	1	1	1	1	1	1	1	1	1	1	1	1	1	1	1	1	1	1	1	1	1	1	1	1	1	0	1	1	1	1	1	1	1	0	1	0	1	1	ybdH	putative oxidoreductase
b0600	1	1	1	1	1	1	1	1	1	1	1	1	1	1	1	1	1	1	1	1	1	1	1	1	1	1	1	1	1	1	0	1	1	1	1	1	1	1	0	1	0	1	1	ybdL	putative aminotransferase
b0601	1	0	1	1	1	1	1	1	1	1	1	1	0	1	1	1	1	1	1	1	1	1	1	1	1	1	1	1	1	1	0	1	1	1	1	1	1	1	0	1	0	1	1	ybdM	orf, hypothetical protein
b0602	1	0	1	1	1	1	1	1	1	1	1	1	0	1	1	1	1	0	0	0	1	0	1	1	1	1	1	1	1	1	0	1	1	1	1	1	1	1	1	1	1	0	0	ybdN	orf, hypothetical protein
b0603	0	0	0	1	1	1	0	1	0	0	0	1	0	0	0	0	1	0	0	0	0	0	1	0	1	1	1	1	1	1	0	1	1	1	1	1	1	1	1	1	1	1	1	ybdO	putative transcriptional regulator LYSR-type
b0604	1	1	1	1	1	1	1	1	1	1	1	1	1	1	1	1	1	1	1	1	1	1	1	1	1	1	1	1	1	1	0	1	1	1	1	1	1	1	1	1	1	1	1	dsbG	thiol:disulfide interchange protein
b0608	0	0	1	0	1	1	1	1	1	1	1	1	0	1	1	1	0	1	1	1	1	1	1	1	1	1	1	1	1	1	1	1	1	1	1	1	1	1	1	1	1	1	1	ybdR	putative oxidoreductase
b0609	0	1	1	0	1	1	1	1	1	1	1	1	1	1	1	1	1	1	1	1	1	1	1	1	1	1	1	1	1	1	1	1	1	1	1	1	1	1	1	1	1	1	1	b0609	orf, hypothetical protein
b0610	0	1	1	1	1	1	1	1	1	1	1	1	1	1	1	1	1	1	1	1	1	1	1	1	1	1	1	1	1	1	1	1	1	1	1	1	1	1	1	1	1	1	1	rnk	regulator of nucleoside diphosphate kinase
b0611	0	1	1	0	1	1	1	1	1	1	1	1	1	1	1	1	0	1	1	1	1	1	1	1	1	1	1	1	1	1	1	1	1	1	1	1	1	1	1	1	1	1	1	rna	RNase I, cleaves phosphodiester bond between any two nucleotides
b0612	0	1	1	1	1	1	1	1	1	1	1	1	1	1	1	1	0	0	0	0	0	0	0	0	1	1	1	1	1	1	1	0	1	1	1	1	1	1	1	1	1	1	1	ybdS	putative a membrane protein
b0613	0	1	1	1	1	1	1	1	1	1	1	1	1	1	1	1	1	0	0	0	0	0	0	0	1	1	1	1	1	1	1	1	1	1	1	1	1	1	1	1	1	1	1	citG	orf, hypothetical protein
b0614	0	1	1	1	1	1	1	1	1	1	1	1	1	1	1	1	1	0	0	0	1	0	1	1	1	1	1	1	1	1	1	1	1	1	1	1	1	1	1	1	1	1	1	ybdU	orf, hypothetical protein
b0615	0	1	1	1	1	1	1	1	1	1	1	1	1	1	1	1	1	1	1	1	1	1	1	1	0	1	1	1	1	1	1	1	1	1	1	1	1	1	1	1	1	1	1	citF	citrate lyase alpha chain
b0616	0	1	1	1	1	1	1	1	1	1	1	1	1	1	1	1	0	0	0	0	0	0	0	0	1	1	1	1	1	1	1	1	1	1	1	1	1	1	1	1	1	1	1	citE	citrate lyase beta chain (acyl lyase subunit)
b0617	0	1	1	1	1	1	1	1	1	1	1	1	1	1	1	1	1	1	1	1	1	1	1	1	1	1	1	1	1	1	1	1	1	1	1	1	1	1	1	1	1	1	1	citD	citrate lyase acyl carrier protein (gamma chain)
b0618	0	1	1	1	1	1	1	1	1	1	1	1	1	1	1	1	0	1	1	1	1	1	0	1	1	1	1	1	1	1	1	1	1	1	1	1	1	1	1	1	1	1	1	citC	citrate lyase synthetase (citrate (pro-3S)-lyase ligase
b0619	0	1	1	1	1	1	1	1	1	1	1	1	1	1	1	1	0	1	1	1	1	1	0	1	1	1	1	1	1	1	1	1	1	0	0	0	0	0	0	0	1	0	0	citA	putative sensor-type protein
b0620	0	1	1	1	1	1	1	1	1	1	1	1	1	1	1	1	0	1	1	1	1	1	0	1	1	1	1	1	1	1	1	1	1	1	1	1	1	1	1	1	1	1	1	citB	sequence similarity to Shigella regulator
b0621	0	1	1	1	1	1	1	1	1	1	1	1	1	1	1	1	0	1	1	1	1	1	0	1	1	1	1	1	1	1	1	1	1	1	1	1	1	1	1	1	1	1	1	dcuC	transport of dicarboxylates
b0645	0	1	1	0	1	1	1	1	1	1	1	1	1	1	1	1	1	1	1	1	1	1	1	1	1	1	1	1	1	1	1	1	1	1	1	1	1	1	1	1	1	1	1	ybeR	orf, hypothetical protein
b0646	0	1	1	0	1	1	1	1	1	1	1	1	1	1	1	1	1	1	1	1	1	1	1	1	1	1	1	1	1	1	1	1	1	1	1	1	1	1	1	1	1	1	1	ybeS	putative enzyme of polynucleotide modification
b0647	0	1	1	0	1	0	0	0	0	0	0	0	0	0	0	0	0	0	0	0	0	0	1	0	1	1	1	1	1	1	1	1	1	1	1	1	1	1	1	1	1	1	1	ybeT	orf, hypothetical protein
b0648	0	1	1	0	1	1	1	1	1	1	1	1	0	0	0	1	1	1	1	1	1	1	1	1	1	1	1	1	1	1	1	1	1	1	1	1	1	1	1	1	1	1	1	ybeU	putative tRNA ligase
b0649	0	1	1	0	1	1	1	1	1	1	1	1	0	0	0	0	0	1	1	1	1	1	1	1	1	1	1	1	1	1	1	1	1	1	1	1	1	1	1	0	1	1	1	ybeV	orf, hypothetical protein
b0650	0	1	1	0	1	1	1	1	1	1	1	1	1	1	1	1	0	1	1	1	1	1	1	1	1	1	1	1	1	1	1	1	1	1	1	1	1	1	1	1	1	1	1	ybeW	putative dnaK protein
b0651	0	1	1	1	1	1	1	1	1	1	1	1	1	1	1	1	0	1	1	1	1	1	1	1	1	1	1	1	1	1	1	1	1	1	1	1	1	1	1	1	1	1	1	ybeK	putative tRNA synthetase
b0656	0	0	0	0	0	0	0	0	0	0	0	0	0	0	0	0	0	0	0	0	0	0	0	0	0	0	0	0	0	0	0	0	0	0	0	0	0	0	0	0	0	0	0	trs5_3	IS5 transposase
b0689	0	0	0	0	0	1	0	0	0	1	1	1	1	1	1	1	1	1	1	1	1	1	1	1	0	0	0	0	0	0	0	0	0	0	0	0	0	0	0	0	0	0	0	ybfP	putative pectinase
b0690	0	0	0	0	0	1	0	1	0	1	0	0	1	1	1	1	1	0	1	1	1	1	1	1	0	0	0	0	0	0	0	0	0	0	0	0	0	0	0	0	0	0	0	ybfG	orf, hypothetical protein
b0691	0	0	0	0	0	0	0	0	0	1	0	0	1	1	1	1	1	1	1	1	1	1	1	1	0	0	0	0	0	0	0	0	0	0	0	0	0	0	0	0	0	0	0	ybfH	orf, hypothetical protein
b0691a	1	1	1	1	1	0	0	0	0	0	1	1	1	1	1	1	1	1	1	1	1	1	1	1	1	1	1	1	1	1	1	0	1	1	1	1	1	1	1	1	1	1	1	ybfI	Predicted DNA-binding protein
b0692	0	1	1	0	1	0	0	0	0	0	0	0	1	1	1	1	1	1	1	1	1	1	1	1	1	1	1	1	1	1	1	1	1	1	1	1	1	1	1	1	1	1	1	potE	putrescine transport protein
b0693	0	1	1	0	1	0	0	0	0	0	0	0	1	1	1	1	1	1	1	1	1	1	1	1	1	1	1	1	1	1	1	1	1	1	1	1	1	1	1	1	1	1	1	speF	ornithine decarboxylase isozyme, inducible
b0693a	0	1	1	0	1	0	1	1	1	0	1	1	1	1	1	0	1	1	1	1	1	1	1	1	1	1	1	1	1	1	1	1	0	1	1	0	0	0	0	0	0	0	0	ybfK	Uncharacterized protein
b0695	1	1	1	1	1	0	1	1	1	0	1	1	1	1	1	1	1	1	1	1	1	1	1	1	1	1	1	1	1	1	1	1	1	1	1	1	1	1	1	1	1	1	1	kdpD	sensor for high-affinity potassium transport system
b0696	1	1	1	1	1	0	1	1	1	0	1	1	1	0	1	1	1	1	1	1	1	1	1	1	1	1	1	1	1	1	1	1	1	1	1	1	1	1	1	1	1	1	1	kdpC	high-affinity potassium transport system
b0697	1	1	1	1	1	0	1	1	1	0	1	1	1	1	1	1	1	1	1	1	1	1	1	1	1	1	1	1	1	1	1	1	1	1	1	1	1	1	1	1	1	1	1	kdpB	ATPase of high-affinity potassium transport system, B chain
b0698	1	1	1	1	1	0	1	1	1	0	1	1	1	1	1	1	1	1	1	1	1	1	1	1	1	1	1	1	1	1	1	1	1	1	1	1	1	1	1	1	1	1	1	kdpA	ATPase of high-affinity potassium transport system, A chain
b0699	1	1	1	1	1	0	1	1	1	0	1	1	1	1	1	1	1	1	1	1	1	1	1	1	1	1	1	1	1	1	1	1	1	1	1	1	1	1	1	1	1	1	1	ybfA	orf, hypothetical protein
b0700	1	1	1	0	1	1	1	1	1	1	1	1	0	1	1	1	1	1	1	1	1	1	1	1	1	1	1	1	1	1	1	1	0	0	0	0	0	0	0	0	0	0	0	rhsC	rhsC protein in rhs element
b0702	0	0	0	0	0	0	0	0	0	0	0	0	0	0	0	0	0	0	0	0	0	0	0	0	1	1	0	0	0	1	1	0	0	0	0	0	0	0	0	0	0	0	0	ybfB	orf, hypothetical protein
b0703	1	1	1	0	1	1	1	1	1	1	1	1	0	1	1	1	1	1	1	1	1	1	1	1	1	1	1	1	1	1	1	1	0	0	0	0	0	0	0	0	0	0	0	b0703	orf, hypothetical protein
b0704	0	0	0	0	1	0	0	0	0	0	0	0	0	0	0	0	0	0	0	0	0	0	0	0	1	1	1	1	1	0	1	0	0	0	0	0	0	0	0	0	0	0	0	ybfC	orf, hypothetical protein
b0705	1	1	1	1	1	0	1	0	0	0	0	0	0	0	0	0	0	0	0	0	1	0	0	0	1	1	1	1	1	1	1	1	0	0	0	0	0	0	0	0	0	0	0	ybfL	putative receptor protein
b0706	1	1	1	1	1	0	1	0	1	0	1	1	0	0	0	0	0	0	0	0	1	1	1	1	1	1	1	1	1	1	1	1	0	0	0	0	0	0	0	0	0	0	0	ybfD	putative DNA ligase
b0707	1	1	1	1	1	1	1	1	1	0	1	1	1	1	1	1	1	1	1	1	1	1	1	1	0	1	1	1	1	1	0	1	0	1	1	1	1	1	1	1	1	1	1	ybgA	orf, hypothetical protein
b0708	1	1	1	1	1	1	1	1	1	0	1	1	1	1	1	1	1	1	1	1	1	1	1	1	1	1	1	1	1	1	1	1	1	1	1	1	1	1	1	1	1	1	1	phrB	deoxyribodipyrimidine photolyase (photoreactivation)
b0709	1	1	1	1	1	1	1	1	1	0	1	1	1	1	1	1	1	1	1	1	1	1	1	1	1	1	1	1	1	1	1	1	1	1	1	1	1	1	1	1	1	1	1	ybgH	putative transport protein
b0710	1	1	1	1	1	1	1	1	1	0	1	1	1	1	1	1	1	1	1	1	1	1	1	1	1	1	1	1	1	1	1	1	1	1	1	1	1	1	1	1	1	1	1	ybgI	orf, hypothetical protein
b0711	1	1	1	1	1	1	1	1	1	0	1	1	1	1	1	1	1	1	1	1	1	1	1	1	1	1	1	1	1	1	1	1	1	1	1	1	1	1	1	1	1	1	1	ybgJ	putative carboxylase
b0712	1	1	1	1	1	1	1	1	1	0	1	1	1	1	1	1	1	1	1	1	1	1	1	1	1	1	1	1	1	1	1	1	1	1	1	1	1	1	1	1	1	1	1	ybgK	putative carboxylase
b0713	1	1	1	1	1	1	1	1	1	0	1	1	1	1	1	1	1	1	1	1	1	1	1	1	1	1	1	1	1	1	1	1	1	1	1	1	1	1	1	1	1	1	1	ybgL	putative lactam utilization protein
b0714	1	1	1	1	1	1	1	1	1	0	1	1	1	1	1	1	1	1	1	1	1	1	1	1	1	1	1	1	1	1	1	1	1	1	1	1	1	1	1	1	1	1	1	nei	endonuclease VIII and DNA N-glycosylase with an AP lyase activity
b0715	1	1	1	1	1	1	1	1	1	0	1	1	1	1	1	1	1	1	1	1	1	1	1	1	1	1	1	1	1	1	1	1	1	1	1	1	1	1	1	1	1	1	1	abrB	putative transport protein
b0716	1	0	1	1	1	1	1	1	1	0	1	1	1	0	1	0	1	1	1	1	1	1	1	1	1	1	1	1	1	1	1	1	1	1	1	1	1	1	1	1	1	1	1	ybgO	orf, hypothetical protein
b0717	1	0	1	1	1	1	1	1	1	0	1	1	1	0	1	0	1	1	1	1	1	1	1	1	1	1	1	1	1	1	1	1	1	1	1	1	1	1	1	1	1	1	1	ybgP	putative chaperone
b0718	1	0	1	1	1	1	1	1	1	0	1	1	1	0	1	0	1	1	1	1	1	1	1	1	1	1	1	1	1	1	1	1	1	1	1	1	1	1	1	1	1	1	1	ybgQ	putative outer membrane protein
b0719	0	0	1	0	0	0	0	0	0	0	0	0	0	0	0	0	0	1	1	0	1	0	0	0	0	0	0	0	0	0	0	0	0	1	1	0	0	0	0	0	0	0	0	ybgD	putative fimbrial-like protein
b0730	0	0	1	0	1	1	1	1	1	1	1	1	1	1	1	1	1	1	1	1	1	1	1	1	1	1	1	1	1	1	1	1	1	1	1	1	1	1	1	1	1	1	1	farR	transcriptional regulator of succinylCoA synthetase operon

b0731	0	0	1	0	1	1	1	1	1	1	1	1	1	1	0	1	1	1	1	1	1	1	1	1	1	1	1	1	1	1	1	1	1	1	1	1	1	1	1	1	1	1	1	hrsA	protein modification enzyme, induction of ompC
b0732	0	0	1	0	1	1	1	1	1	1	1	0	1	1	1	1	1	1	1	1	1	1	1	1	1	1	1	1	1	0	1	1	1	1	1	1	1	1	1	1	1	1	1	ybgG	putative sugar hydrolase
b0739	1	1	1	1	1	1	1	1	1	1	1	1	1	1	1	1	1	1	1	1	1	1	1	1	1	1	1	1	1	1	1	1	1	1	1	1	1	1	1	1	0	1	1	tolA	membrane spanning protein, required for outer membrane integrity
b0753	1	0	1	1	1	1	1	1	1	1	1	1	1	1	1	1	1	1	1	1	1	1	1	1	1	1	1	1	1	1	1	1	1	1	1	1	1	1	1	1	1	1	1	b0753	putative homeobox protein
b0766	1	1	0	1	1	1	1	1	1	1	1	1	1	1	1	1	1	1	1	1	1	1	1	1	1	1	1	1	1	1	1	1	1	1	1	1	1	1	1	1	1	1	1	ybhA	putative phosphatase
b0768	1	1	1	1	1	1	1	1	1	1	1	1	1	1	1	1	1	1	1	1	1	1	1	1	0	0	1	1	1	0	0	1	0	1	1	1	1	1	1	1	1	1	1	ybhD	putative transcriptional regulator LYSR-type
b0769	0	1	0	1	1	1	1	1	1	1	1	1	0	0	0	0	0	1	1	0	1	0	1	0	1	1	1	1	1	1	1	1	0	1	1	1	1	1	1	1	1	1	1	ybhH	orf, hypothetical protein
b0770	0	1	0	1	1	1	1	1	1	1	1	1	0	0	0	1	1	1	1	0	1	0	1	1	1	1	1	1	1	1	1	1	0	1	1	1	1	1	1	1	1	1	1	ybhI	putative membrane pump protein
b0771	0	1	1	1	1	1	1	1	1	1	1	1	1	1	1	1	1	1	1	1	1	1	1	1	1	1	1	1	1	1	1	1	1	1	1	1	1	1	1	1	1	1	1	ybhJ	putative enzyme
b0772	0	1	1	1	1	1	1	1	1	1	1	1	1	1	1	1	1	1	1	1	1	1	1	1	1	1	1	1	1	1	1	1	1	1	1	1	1	1	1	1	1	1	1	ybhC	putative pectinesterase
b0787	0	0	1	1	1	1	1	1	1	1	1	1	1	1	1	1	1	1	1	1	1	1	1	1	1	1	1	1	1	1	1	1	1	1	1	1	1	1	1	1	1	1	1	ybhM	orf, hypothetical protein
b0788	1	0	1	1	1	1	1	1	1	1	1	1	1	1	1	1	1	1	1	1	1	1	1	1	1	1	1	1	1	1	1	1	1	1	1	1	1	1	1	1	1	1	1	ybhN	orf, hypothetical protein
b0789	1	0	1	1	1	1	1	1	1	1	1	1	1	1	1	1	1	1	1	1	1	1	1	1	1	1	1	1	1	1	1	1	1	1	1	1	1	1	1	1	1	1	1	ybhO	putative synthetase
b0798	0	1	1	0	1	1	1	1	1	1	1	1	1	1	1	1	1	1	1	1	1	1	1	1	1	1	1	1	1	1	1	1	1	1	1	1	1	1	1	1	1	1	1	ybiA	orf, hypothetical protein
b0803	1	0	1	1	1	1	1	1	1	1	1	1	1	1	0	1	1	0	1	0	1	1	1	1	1	1	1	1	1	1	1	1	1	1	1	1	1	1	1	1	1	1	1	ybiI	orf, hypothetical protein
b0804	1	0	1	1	1	1	1	1	1	1	1	1	1	1	1	1	1	1	1	1	1	1	1	1	1	1	1	1	1	1	0	1	1	1	1	1	1	1	1	1	1	1	1	ybiX	putative enzyme
b0805	1	0	1	1	1	1	1	1	1	1	1	1	1	1	0	1	1	1	1	1	1	1	1	1	1	1	1	1	1	1	1	1	1	1	1	1	1	1	1	1	1	1	1	b0805	putative outer membrane receptor for iron transport
b0806	1	1	1	1	1	1	1	1	1	1	1	1	1	1	0	1	1	1	1	1	1	1	1	1	1	1	1	1	1	1	1	1	1	1	1	1	1	1	1	1	1	1	1	ybiM	orf, hypothetical protein
b0821	1	0	1	1	1	1	1	1	1	1	1	1	1	1	1	1	1	1	1	1	1	1	1	1	1	1	1	1	1	1	1	1	1	1	1	1	1	1	1	1	1	1	1	ybiU	orf, hypothetical protein
b0833	0	1	1	1	0	1	1	1	1	1	1	1	0	1	1	1	1	1	1	1	1	1	1	1	1	1	1	1	1	1	1	1	1	1	1	1	1	1	1	1	1	1	1	b0833	orf, hypothetical protein
b0834	0	1	1	0	1	1	1	1	1	1	1	1	1	1	1	1	1	1	1	1	1	1	1	1	1	1	1	1	1	1	1	1	1	1	1	1	1	1	1	1	1	1	1	b0834	orf, hypothetical protein
b0835	0	1	1	1	1	1	1	1	1	1	1	1	1	1	1	1	1	1	1	1	1	1	1	1	1	1	1	1	1	1	1	1	1	1	1	1	1	1	1	1	1	1	1	yliG	orf, hypothetical protein
b0836	0	1	1	1	1	1	1	1	0	1	1	1	1	1	1	1	1	1	1	1	1	1	1	1	1	1	1	1	1	1	1	1	1	1	1	1	1	1	1	1	1	1	1	b0836	putative receptor
b0837	0	0	1	1	1	1	1	1	1	1	1	1	1	1	1	1	1	1	1	1	1	1	1	1	1	1	1	1	1	1	1	1	1	1	1	1	1	1	1	1	1	1	1	yliI	putative dehydrogenase
b0838	1	1	1	0	1	1	1	1	1	1	1	1	1	1	1	1	1	1	1	1	1	1	1	1	1	1	1	1	1	1	1	1	1	1	1	1	1	1	1	1	1	1	1	yliJ	putative transferase
b0843	0	1	1	1	1	1	1	1	1	1	1	1	1	1	1	1	1	1	1	1	1	1	1	1	1	1	1	1	1	1	1	1	1	1	1	1	1	1	1	1	1	1	1	ybjH	orf, hypothetical protein
b0844	0	1	1	1	1	1	1	1	1	1	1	1	1	1	1	1	1	1	1	1	1	1	1	1	1	1	1	1	1	1	1	1	1	1	1	1	1	1	1	1	1	1	1	b0844	orf, hypothetical protein
b0865	1	1	1	1	1	1	1	1	1	1	1	1	1	1	1	1	1	1	1	1	1	1	1	1	1	1	0	1	1	0	1	1	0	1	1	1	1	1	1	1	1	1	1	ybjP	putative enzyme
b0875	1	1	0	1	1	1	1	1	1	1	1	1	1	1	1	1	1	1	1	1	1	1	1	1	1	1	1	1	1	1	1	1	1	1	1	1	1	1	1	1	1	1	1	aqpZ	transmembrane water channel; aquaporin Z
b0878	1	1	1	1	1	1	1	1	1	1	1	1	1	1	1	1	1	1	1	1	1	1	1	1	0	0	0	0	1	1	1	1	1	1	1	1	1	1	1	1	1	1	1	b0878	putative membrane protein
b0879	1	1	1	1	1	1	1	1	1	1	1	1	1	1	1	1	1	1	1	1	1	1	1	1	0	0	0	0	1	1	1	1	1	1	1	1	1	1	1	1	1	1	1	ybjZ	putative ATP-binding component of a transport system
b0895	1	1	1	1	1	1	1	1	1	1	1	1	1	1	1	1	1	1	1	0	1	1	1	1	1	1	1	1	1	1	1	1	1	1	1	1	1	1	1	1	1	1	1	dmsB	anaerobic dimethyl sulfoxide reductase subunit B
b0896	1	1	1	1	1	1	1	1	1	1	1	1	1	1	1	0	1	0	0	0	1	1	1	1	1	1	0	1	1	1	1	1	1	1	1	1	1	1	1	1	1	1	1	dmsC	anaerobic dimethyl sulfoxide reductase subunit C
b0897	1	1	1	1	1	1	1	1	1	1	1	1	1	1	1	0	1	0	0	0	1	1	1	0	1	1	1	1	1	1	1	1	1	1	1	1	1	1	1	1	1	1	1	ycaC	orf, hypothetical protein
b0898	1	1	1	1	1	0	0	0	0	0	0	0	0	1	1	1	1	0	0	0	0	1	1	1	1	1	1	1	1	0	1	0	1	1	1	1	1	1	1	1	1	1	1	ycaD	putative transport
b0899	1	1	1	1	1	0	0	0	0	0	0	0	1	1	1	1	0	0	0	0	1	1	1	1	1	1	1	1	1	1	1	1	0	0	0	0	0	0	0	0	1	1	1	b0899	putative transport
b0900	0	0	1	0	1	1	0	0	1	1	1	1	1	1	1	1	1	0	1	1	1	1	1	1	0	1	1	1	1	1	1	1	1	1	0	1	1	1	1	1	1	1	1	ycaN	putative transcriptional regulator LYSR-type
b0901	0	0	1	0	1	1	1	1	1	1	1	1	1	1	1	1	0	1	1	1	1	1	1	1	0	1	1	1	1	1	1	1	0	1	0	1	1	1	1	1	0	1	1	ycaK	orf, hypothetical protein
b0906	1	0	1	1	1	1	1	1	1	1	1	1	1	1	1	1	1	1	1	1	1	1	1	1	1	1	1	1	1	1	1	1	1	1	1	1	1	1	1	1	1	1	1	ycaP	orf, hypothetical protein
b0909	1	0	1	1	1	1	1	1	1	1	1	1	1	1	1	1	1	1	1	1	1	1	1	1	1	1	1	1	1	1	1	1	1	1	1	1	1	1	1	1	1	1	1	ycaL	putative heat shock protein
b0913	1	1	1	0	1	1	1	1	1	1	1	1	1	1	1	1	1	1	1	1	1	1	1	1	1	1	1	1	1	1	1	1	1	1	1	1	1	1	1	1	1	1	1	ycaI	orf, hypothetical protein
b0929	1	1	1	1	1	1	1	1	1	1	1	1	1	1	1	1	1	0	1	1	1	1	1	1	1	1	1	1	1	1	1	1	1	1	1	1	1	1	1	1	1	1	1	ompF	outer membrane protein 1a (Ia;b;F)
b0933	1	0	1	1	1	1	1	1	1	1	1	1	1	1	1	1	1	1	1	1	1	1	1	1	1	1	1	1	1	1	1	1	1	1	1	1	1	1	1	1	1	1	1	ycbE	putative ATP-binding component of a transport system
b0934	1	0	1	1	1	1	1	1	1	1	1	1	1	1	1	1	1	1	1	1	1	1	1	1	1	1	1	1	1	1	1	1	0	1	1	1	1	1	1	1	1	1	1	ycbM	putative transport system permease protein
b0935	1	1	1	1	1	1	1	1	1	1	1	1	1	1	1	1	1	1	1	1	1	1	1	1	1	1	1	1	1	1	1	1	0	1	1	1	1	1	1	1	1	1	1	ycbN	orf, hypothetical protein
b0936	1	0	1	1	1	1	1	1	1	1	1	1	1	1	1	1	1	1	1	1	1	1	1	1	1	1	1	1	1	1	0	1	0	1	1	1	1	1	1	1	1	1	1	ycbO	orf, hypothetical protein
b0937	1	0	1	1	1	1	1	1	1	1	1	1	1	1	1	1	1	1	1	1	1	1	1	1	1	0	0	1	0	0	0	1	0	1	1	1	1	1	1	1	1	1	1	ycbP	orf, hypothetical protein
b0938	0	0	1	1	1	0	1	1	1	1	1	1	0	1	0	1	1	1	1	1	0	1	1	0	1	1	1	1	1	1	1	0	1	1	1	0	0	0	0	0	1	0	0	ycbQ	putative fimbrial-like protein
b0939	0	0	1	1	1	1	1	1	1	1	1	1	0	0	0	0	1	1	1	1	0	1	1	0	1	1	1	1	1	1	1	1	1	1	0	0	0	0	0	0	1	1	1	ycbR	putative chaperone
b0940	0	0	1	1	1	1	1	1	1	1	1	1	0	0	0	0	1	0	0	0	0	1	1	0	1	1	1	1	1	1	1	1	1	1	1	1	1	1	1	1	1	1	1	ycbS	putative outer membrane protein
b0941	1	0	1	1	1	1	1	1	1	1	1	1	1	0	0	0	1	1	1	1	1	1	1	1	1	1	1	1	1	1	1	1	1	1	1	1	1	1	1	1	1	1	1	b0941	homolog of Salmonella FimH protein
b0942	1	0	1	1	1	1	1	1	1	1	1	1	1	0	0	0	1	1	1	1	1	1	1	1	1	1	1	1	1	0	1	1	1	1	1	1	1	1	1	1	1	1	1	b0942	putative fimbrial-like protein
b0943	1	0	1	1	1	1	1	1	1	1	1	1	1	0	0	0	1	1	1	1	1	1	1	1	1	1	1	1	1	0	1	1	1	1	1	1	1	1	1	1	1	1	1	b0943	putative fimbrial-like protein
b0981	1	1	1	1	1	0	0	1	0	1	1	1	1	1	1	1	1	1	1	1	1	1	1	1	1	1	1	1	1	1	1	1	1	1	1	1	1	1	1	1	0	1	1	yccC	orf, hypothetical protein
b0982	1	1	1	1	1	1	1	1	1	1	1	1	1	1	1	1	1	1	1	1	1	1	1	1	1	1	1	1	1	1	1	1	1	1	1	1	1	1	1	1	0	1	1	yccY	putative phosphatase
b0983	1	1	1	1	1	1	1	1	1	1	1	1	1	1	1	1	1	1	1	1	1	1	1	1	1	1	1	1	1	1	1	1	1	1	1	1	1	1	1	1	0	1	1	yccZ	putative function in exopolysaccharide production
b0984	1	1	1	1	1	1	1	1	1	1	1	1	0	1	1	1	1	1	1	1	1	1	1	1	1	1	1	1	1	1	1	1	1	1	1	1	1	1	1	1	0	1	1	ymcA	orf, hypothetical protein
b0985	1	1	1	1	1	0	1	1	1	1	1	1	1	1	1	1	1	1	1	1	1	1	1	1	1	1	1	1	1	1	1	1	1	1	1	1	1	1	0	1	0	1	1	ymcB	orf, hypothetical protein
b0986	1	1	1	1	1	1	1	1	1	1	1	1	1	1	1	1	1	1	1	1	1	1	1	1	1	1	1	1	1	1	1	1	1	1	1	1	1	1	0	1	0	1	1	ymcC	putative regulator
b0989	1	1	1	1	1	1	1	1	1	1	1	1	1	1	1	1	1	1	1	1	1	1	1	1	1	1	1	1	1	1	1	1	1	1	1	1	1	1	0	1	0	1	1	cspH	cold shock-like protein
b0990	0	1	1	1	1	1	1	1	1	1	1	1	1	1	1	1	1	1	1	1	1	1	1	1	1	1	1	1	1	1	1	1	1	1	1	1	1	1	0	1	0	1	1	cspG	homolog of Salmonella cold shock protein
b0991	0	1	1	1	1	1	1	1	1	1	1	1	1	1	1	1	1	1	1	1	1	1	1	1	1	1	1	1	1	1	1	1	1	1	1	1	0	0	0	0	0	1	1	sfa	suppresses fabA and ts growth mutation
b0992	1	1	1	1	1	1	1	1	1	1	0	1	1	1	1	1	1	1	1	1	1	1	1	1	0	0	0	0	1	1	1	1	1	1	1	1	0	0	0	0	0	1	1	yccM	orf, hypothetical protein
b0993	1	1	1	1	1	1	1	1	1	1	0	1	1	1	1	1	1	0	0	0	0	0	0	0	0	0	0	0	1	1	1	1	0	1	1	1	0	0	0	0	0	1	1	torS	sensor protein torS (regulator TorR)
b0995	1	1	1	1	1	1	1	1	1	1	0	1	1	1	1	1	1	1	1	1	1	1	1	1	0	0	0	0	1	1	1	1	0	1	1	1	1	1	1	1	0	1	1	torR	response transcriptional regulator for torA (sensor TorS)
b0996	1	1	1	1	1	1	1	1	1	1	0	1	1	1	1	1	1	1	1	1	1	1	1	1	0	0	0	0	1	1	1	1	0	1	1	1	1	1	1	1	0	1	1	torC	trimethylamine N-oxide reductase, cytochrome c-type subunit
b0997	1	1	1	1	1	1	1	1	1	1	0	1	1	1	1	1	1	1	1	1	1	1	1	1	0	0	0	0	1	1	1	1	0	1	1	1	1	1	1	1	0	1	1	torA	trimethylamine N-oxide reductase subunit
b0998	1	1	1	1	1	1	1	1	1	1	0	1	1	1	1	1	1	1	1	1	1	1	1	1	1	1	1	1	1	1	1	1	1	1	1	1	1	1	1	1	1	1	1	torD	part of trimethylamine-N-oxide oxidoreductase
b0999	1	1	1	1	1	1	1	1	1	1	0	1	1	1	1	1	1	1	1	1	1	1	1	1	1	1	1	1	1	1	1	1	1	1	1	1	1	1	1	1	1	1	1	yccD	orf, hypothetical protein
b1000	1	1	1	1	1	1	1	1	1	1	0	1	1	1	1	1	1	1	1	1	1	1	1	1	1	1	1	1	1	1	1	1	1	1	1	1	1	1	1	1	1	1	1	cbpA	curved DNA-binding protein; functions closely related to DnaJ
b1001	1	0	1	1	1	1	0	1	1	1	0	1	0	0	1	1	1	1	1	1	0	1	1	1	0	0	1	1	1	1	0	0	1	1	1	1	1	1	1	1	1	1	1	yccE	orf, hypothetical protein
b1002	1	1	1	1	1	0	0	0	0	0	0	0	0	0	0	0	0	0	0	0	0	0	0	0	1	1	1	1	1	1	1	0	1	1	1	1	1	1	1	1	1	1	1	agp	periplasmic glucose-1-phosphatase
b1003	1	1	1	1	1	0	1	1	1	1	1	1	0	0	1	1	1	1	1	1	1	1	1	1	1	1	1	1	1	1	1	0	1	1	1	1	1	1	1	1	1	1	1	yccJ	orf, hypothetical protein
b1004	1	1	1	1	1	0	0	1	0	0	0	0	0	0	0	0	0	0	0	0	1	1	1	0	1	1	1	1	1	1	1	0	1	1	1	1	1	1	1	1	1	1	1	wrbA	trp repressor binding protein; affects association of trp repressor and operator
b1005	1	1	1	1	1	0	0	0	0	0	0	0	0	0	0	0	0	0	0	0	0	0	0	0	1	1	1	1	1	1	1	1	1	1	1	1	1	1	1	1	1	1	1	ycdF	orf, hypothetical protein
b1006	1	0	1	1	1	0	0	0	0	0	0	0	0	0	0	0	0	0	0	0	0	0	0	0	1	1	1	1	1	1	1	1	1	1	1	1	1	1	1	1	1	1	1	ycdG	putative transport protein
b1007	1	1	1	1	1	0	0	0	0	0	0	0	0	0	0	0	0	0	0	0	0	0	0	0	1	1	1	1	1	1	1	1	1	1	1	1	1	1	1	1	1	1	1	b1007	orf, hypothetical protein
b1008	1	1	1	1	1	1	1	1	1	1	1	1	0	0	1	1	1	1	1	1	1	1	1	1	1	1	1	1	1	1	1	1	1	1	1	1	1	1	1	1	1	1	1	b1008	putative enzyme
b1009	1	0	1	1	1	0	0	0	0	0	0	0	0	0	0	0	0	0	0	0	0	0	0	0	1	1	1	1	1	1	1	1	1	1	1	1	1	1	1	1	1	1	1	b1009	putative acetyltransferase
b1010	1	1	1	1	1	0	0	0	0	0	0	0	0	0	0	0	0	0	0	0	0	0	0	0	1	1	1	1	1	1	1	1	1	1	1	1	1	1	1	1	1	1	1	b1010	orf, hypothetical protein
b1011	1	1	1	1	1	0	0	0	0	0	0	1	0	0	0	0	0	0	0	0	0	0	0	0	1	1	1	1	1	1	1	1	1	1	1	1	1	1	1	1	1	1	1	b1011	putative synthetase
b1012	1	1	1	1	1	1	1	1	1	0	1	1	1	0	0	1	1	0	0	0	1	1	1	1	1	1	1	1	1	1	1	1	1	1	1	1	1	1	1	1	1	1	1	b1012	orf, hypothetical protein
b1014	1	1	1	1	1	1	1	1	1	1	1	1	1	1	1	1	1	1	1	1	1	1	1	1	1	1	1	1	1	1	1	1	0	0	0	0	0	0	0	0	0	0	0	putA	proline dehydrogenase, P5C dehydrogenase
b1015	1	1	1	1	1	1	1	1	1	1	1	1	1	1	1	1	1	1	1	1	1	1	1	1	1	1	1	1	1	1	1	1	0	0	0	0	0	0	0	0	0	0	0	putP	major sodium/proline symporter
b1016m	0	1	1	1	1	0	0	0	0	0	0	0	1	1	1	1	1	1	1	1	1	1	1	1	0	0	0	0	1	1	0	1	1	1	1	1	1	1	1	1	0	1	1	ycdN	High-affinity iron permease
b1018	0	1	1	1	1	0	0	0	0	0	0	0	1	1	1	1	1	1	1	1	1	1	1	1	0	0	0	0	1	1	0	1	1	1	1	1	1	1	1	1	0	1	1	ycdO	orf, hypothetical protein
b1019	0	1	1	1	1	0	0	0	0	0	0	0	1	1	1	1	1	1	1	1	1	1	1	1	0	0	0	0	1	1	0	1	1	1	1	1	1	1	1	1	0	1	1	ycdB	orf, hypothetical protein
b1020	0	1	1	1	1	1	1	1	1	1	1	1	1	1	1	1	0	1	1	1	1	1	1	1	0	0	0	0	1	1	0	1	1	1	1	1	1	1	1	1	0	1	1	phoH	PhoB-dependent, ATP-binding pho regulon component; may be helicase; induced by P starvation
b1021	0	0	0	0	1	0	0	0	0	0	0	0	0	0	0	0	0	0	0	0	0	0	0	0	0	0	0	0	0	0	0	1	1	1	0	0	1	1	1	1	0	1	1	ycdP	orf, hypothetical protein
b1022	0	0	0	0	1	0	0	0	0	0	0	0	0	0	0	0	0	0	0	0	0	0	0	0	0	0	0	0	0	0	0	0	1	1	0	0	1	1	1	1	0	1	1	ycdQ	orf, hypothetical protein
b1023	0	0	0	0	0	0	0	0	0	0	0	0	0	0	0	0	0	0	0	0	0	0	0	0	0	0	0	0	0	0	0	0	1	1	0	0	0	0	1	0	0	1	1	ycdR	orf, hypothetical protein
b1024	0	0	0	0	1	0	0	0	0	0	0	0	0	0	0	0	0	0	0	0	1	0	1	0	0	0	0	0	0	0	0	1	0	1	0	0	1	0	1	1	0	1	1	ycdS	putative outer membrane protein
b1025	0	0	0	0	0	0	0	0	0	0	0	0	0	0	0	0	0	0	0	0	0	0	0	0	0	0	0	0	0	0	0	0	0	1	0	0	1	0	1	1	0	0	0	ycdT	orf, hypothetical protein
b1028	0	0	0	0	0	0	0	0	0	0	0	0	0	0	0	0	0	0	0	0	0	0	0	0	0	0	0	0	0	0	0	0	1	1	1	1	1	0	1	1	1	0	0	b1028	orf, hypothetical protein
b1029	0	1	0	1	1	1	1	1	1	1	1	1	0	0	1	1	1	1	1	1	1	1	1	1	1	1	1	1	1	1	1	0	1	1	1	1	1	1	1	1	1	1	1	ycdU	orf, hypothetical protein
b1030	0	1	1	1	0	1	1	1	1	1	1	1	1	1	1	1	1	1	1	1	1	1	1	1	0	0	0	1	1	1	0	1	1	1	1	0	0	0	0	0	0	0	0	b1030	orf, hypothetical protein
b1031	0	1	1	1	0	1	1	1	1	1	1	1	1	1	1	1	1	0	1	1	1	1	1	1	0	0	0	1	1	1	0	1	0	1	1	0	0	0	0	0	0	0	0	ycdV	putative ribosomal protein
b1037	1	1	1	1	1	1	1	1	1	1	1	1	1	1	1	1	1	1	1	1	1	1	1	1	1	1	1	1	1	1	1	1	1	1	1	1	1	1	1	1	1	0	0	csgG	curli production assembly/transport component, 2nd curli operon
b1038	1	1	1	1	1	1	1	1	1	1	1	1	0	0	0	1	1	1	1	1	1	1	1	1	1	1	1	1	1	1	1	1	1	1	1	1	1	1	1	1	1	0	0	csgF	curli production assembly/transport component, 2nd curli operon
b1039	1	1	1	1	1	1	1	1	1	1	1	1	0	0	1	0	0	1	1	1	1	1	1	1	1	1	1	1	1	1	1	1	1	1	1	1	1	1	1	1	1	0	0	csgE	curli production assembly/transport component, 2nd curli operon
b1040	1	1	1	1	1	0	0	0	0	1	0	0	1	1	1	0	1	1	1	1	1	1	1	1	1	1	1	1	1	1	1	1	1	1	1	1	1	1	1	1	1	0	0	csgD	putative 2-component transcriptional regulator for 2nd curli operon
b1041	0	1	1	1	1	0	0	0	0	0	0	0	1	1	1	1	0	1	1	1	1	1	1	1	1	1	1	1	1	1	1	1	1	1	1	1	1	1	1	1	1	0	0	csgB	minor curlin subunit precursor, similar ro CsgA
b1042	0	1	1	1	1	0	0	0	0	0	0	0	1	1	1	1	0	1	1	1	1	1	1	1	1	1	1	1	1	1	0	1	1	1	1	1	1	1	1	1	1	0	0	csgA	curlin major subunit, coiled surface structures; cryptic
b1043	0	1	1	0	1	1	1	1	1	1	1	1	1	1	1	1	0	1	1	1	1	1	1	1	1	1	1	1	1	1	1	1	1	1	1	1	1	1	1	1	1	0	0	csgC	putative curli production protein
b1044	1	0	1	0	1	1	1	1	1	1	1	1	1	1	1	1	0	1	1	1	1	1	1	1	1	1	1	1	1	1	1	1	1	1	1	1	1	1	1	1	1	0	0	b1044	orf, hypothetical protein
b1045	1	1	1	0	1	1	1	1	1	1	1	1	1	1	1	1	0	1	1	1	1	1	1	1	1	1	1	1	1	1	1	1	1	1	1	1	1	1	1	1	1	1	1	b1045	putative polyprotein

b1047	1	1	1	1	1	0	1	1	1	1	1	1	0	1	1	1	1	1	1	1	1	1	1	1	1	1	1	1	1	1	1	1	1	1	1	1	1	1	1	1	1	1	1	ymdD	orf, hypothetical protein
b1050	1	1	1	1	1	1	1	1	1	1	1	1	1	1	1	1	1	1	1	1	1	1	1	1	1	1	0	1	1	0	0	1	0	0	0	1	1	1	1	1	1	1	1	yceK	orf, hypothetical protein
b1051	1	1	1	1	1	1	1	1	1	1	1	1	1	1	1	1	1	1	1	1	1	1	1	1	0	0	0	0	0	0	0	0	0	0	0	0	0	0	0	0	0	0	0	msyB	acidic protein suppresses mutants lacking function of protein export
b1056	1	0	1	1	1	1	1	1	1	1	1	1	1	1	1	1	1	1	1	1	1	1	1	1	1	1	1	1	1	1	1	1	1	1	1	1	1	1	1	1	1	1	1	yceI	orf, hypothetical protein
b1057	1	0	1	1	1	1	1	1	1	1	1	1	1	1	1	1	1	1	1	1	1	1	1	1	1	1	1	1	1	1	1	1	1	1	1	1	1	1	1	1	1	1	1	b1057	putative cytochrome
b1062	1	1	1	1	1	0	0	0	0	1	1	1	0	1	1	1	1	1	1	1	1	1	1	1	1	1	1	1	1	1	1	1	1	1	1	1	1	1	1	1	1	1	1	pyrC	dihydro-orotase
b1064	1	0	1	1	1	1	1	1	1	1	1	1	1	1	1	1	1	1	1	1	1	1	1	1	1	1	1	1	1	1	1	1	1	1	1	1	1	1	1	1	1	1	1	grxB	glutaredoxin 2
b1066	1	1	1	1	1	1	1	1	0	1	0	1	1	1	1	1	1	1	1	1	1	1	1	1	1	1	1	1	1	1	1	1	1	1	1	1	1	1	1	1	1	1	1	rimJ	acetylation of N-terminal alanine of 30S ribosomal subunit protein S5
b1073	1	1	1	1	1	1	1	1	1	1	1	1	0	0	1	0	1	1	1	1	1	1	1	1	1	1	1	1	1	1	1	1	1	1	1	1	1	1	1	1	1	1	1	flgB	flagellar biosynthesis, cell-proximal portion of basal-body rod
b1080	1	1	1	1	1	1	1	1	1	1	1	1	1	1	1	1	0	1	1	1	1	1	1	1	1	1	1	1	1	1	1	1	1	1	1	1	1	1	1	1	1	1	1	flgI	homolog of Salmonella P-ring of flagella basal body
b1102	1	0	1	1	1	1	1	1	1	1	1	1	1	1	1	1	1	1	1	1	1	1	1	1	1	1	1	1	1	1	1	1	1	1	1	1	1	1	1	1	1	1	1	fhuE	outer membrane receptor for ferric iron uptake
b1107	1	1	1	1	1	0	0	0	0	1	1	1	0	1	1	0	1	1	1	1	1	1	1	1	1	1	1	1	1	1	1	1	1	1	1	1	1	1	1	1	1	1	1	ycfO	orf, hypothetical protein
b1115	1	0	1	1	1	1	1	1	1	1	1	1	1	1	1	1	1	1	1	1	1	1	1	1	1	1	1	1	1	1	1	1	1	1	1	1	1	1	1	1	1	1	1	ycfT	orf, hypothetical protein
b1119	1	1	1	1	1	1	1	1	1	1	1	1	1	1	1	1	1	1	1	1	1	1	1	1	0	1	1	1	0	1	1	1	1	1	1	1	1	1	1	1	1	1	1	ycfX	putative NAGC-like transcriptional regulator
b1120	1	1	1	1	1	1	1	1	1	1	1	1	1	1	1	1	1	1	1	1	1	1	1	1	1	1	1	1	1	1	1	1	0	1	1	1	1	1	1	1	1	1	1	cobB	putative nicotinic acid mononucleotide:5,6-dimethylbenzimidazole (DMB) phosphoribosyltransferase
b1121	0	1	0	1	0	0	0	0	0	0	0	0	0	0	0	0	0	0	0	0	0	0	0	0	0	0	0	0	0	0	0	0	0	0	0	0	0	0	0	0	0	0	0	b1121	homolog of virulence factor
b1122	0	1	0	1	0	0	0	0	0	0	0	0	0	0	0	0	0	0	0	0	0	0	0	0	0	0	0	0	0	0	0	0	0	0	0	0	0	0	0	0	0	0	0	ymfA	orf, hypothetical protein
b1125	1	0	1	1	1	1	1	1	1	1	1	1	1	1	1	1	1	1	1	1	1	1	1	1	1	1	1	1	1	1	1	1	1	1	1	1	1	1	1	1	1	1	1	potB	spermidine/putrescine transport system permease
b1126	1	0	1	1	1	1	1	1	1	1	1	1	1	1	1	1	1	1	1	1	1	1	1	1	1	1	1	1	1	1	1	1	1	1	1	1	1	1	1	1	1	1	1	potA	ATP-binding component of spermidine/putrescine transport
b1127	1	0	1	1	1	1	1	1	1	1	1	1	1	1	1	1	1	1	1	1	1	1	1	1	1	1	1	1	1	1	1	1	1	1	1	1	1	1	1	1	1	1	1	pepT	putative peptidase T
b1128	1	0	1	1	1	1	1	1	1	1	1	1	1	1	1	1	1	1	1	1	1	1	1	1	1	1	1	1	1	1	1	1	1	1	1	1	1	1	1	1	1	1	1	ycfD	orf, hypothetical protein
b1137	0	0	0	0	0	0	0	0	0	0	0	0	0	0	0	0	0	0	0	0	0	0	0	0	0	0	0	0	0	0	0	0	0	0	0	0	0	0	0	0	0	0	0	ymfD	orf, hypothetical protein
b1138	0	0	0	0	0	0	0	0	0	0	0	0	0	0	0	0	0	0	0	0	0	0	0	0	0	0	0	0	0	0	0	0	0	0	0	0	0	0	0	0	0	0	0	ymfE	orf, hypothetical protein
b1139	0	0	0	0	0	0	0	0	0	0	0	0	0	0	0	0	0	0	0	0	0	0	0	0	0	0	0	0	0	0	0	0	0	0	0	0	0	0	0	0	0	0	0	lit	phage T4 late gene expression; at locus of e14 element
b1140	0	0	0	0	0	0	0	0	0	0	0	0	0	0	0	0	0	0	0	0	0	0	0	0	0	0	0	0	0	0	0	0	0	0	0	0	0	0	0	0	0	0	0	intE	prophage e14 integrase
b1141	0	0	0	0	0	0	0	0	0	0	0	0	0	0	0	0	0	0	0	0	0	0	0	0	0	0	0	0	0	0	0	0	0	0	0	0	0	0	0	0	0	0	0	b1141	orf, hypothetical protein
b1142	0	0	0	0	0	0	0	0	0	0	0	0	0	0	0	0	0	0	0	0	0	0	0	0	0	0	0	0	0	0	0	0	0	0	0	0	0	0	0	0	0	0	0	ymfH	orf, hypothetical protein
b1143	0	0	0	0	0	0	0	0	0	0	0	0	0	0	0	0	0	0	0	0	0	0	0	0	0	0	0	0	0	0	0	0	0	0	0	0	0	0	0	0	0	0	0	ymfI	orf, hypothetical protein
b1144	0	0	0	0	0	0	0	0	0	0	0	0	0	0	0	0	0	0	0	0	0	0	0	0	0	0	0	0	0	0	0	0	0	0	0	0	0	0	0	0	0	0	0	ymfJ	orf, hypothetical protein
b1145	0	0	0	0	0	1	1	1	1	1	1	1	0	1	0	0	0	0	0	1	0	1	1	0	0	0	0	0	0	0	0	0	0	0	0	0	0	0	0	0	0	1	1	b1145	putative phage repressor
b1146	0	0	0	0	0	1	1	1	1	1	1	1	0	1	0	0	0	0	0	1	1	1	1	0	0	0	0	0	0	0	0	0	0	0	0	0	0	0	0	0	0	1	1	b1146	orf, hypothetical protein
b1147	0	0	0	0	1	1	1	1	1	1	1	1	0	1	0	0	1	0	0	1	1	1	1	0	0	0	0	0	0	0	0	0	0	0	0	0	0	0	0	0	0	1	1	ymfL	orf, hypothetical protein
b1148	0	0	0	0	0	1	1	1	0	1	0	1	0	0	0	0	0	0	0	1	0	0	1	0	0	0	0	1	0	0	0	0	0	0	0	0	0	0	0	0	0	0	0	ymfM	orf, hypothetical protein
b1149	0	0	1	0	0	0	0	0	0	0	0	0	0	0	0	0	0	0	0	0	0	0	0	0	0	0	0	0	0	0	0	0	0	1	0	0	0	0	0	1	0	1	1	ymfN	orf, hypothetical protein
b1150	0	0	1	0	0	1	1	1	0	1	0	1	0	0	0	0	0	0	0	0	0	0	1	0	0	0	0	1	0	0	0	0	0	1	0	0	0	0	0	1	1	1	1	ymfR	orf, hypothetical protein
b1151	0	0	0	0	0	1	0	0	0	0	0	0	0	0	0	0	0	0	0	0	0	0	1	1	0	0	0	0	0	0	1	1	0	1	0	0	0	0	0	1	0	1	1	ymfO	orf, hypothetical protein
b1152	0	0	0	0	0	0	0	0	0	0	0	0	0	0	1	0	0	0	0	0	0	0	0	0	0	0	0	0	0	0	0	0	0	1	0	0	0	0	0	1	0	1	1	b1152	orf, hypothetical protein
b1153	0	0	0	0	0	0	0	0	0	0	0	0	0	0	1	0	0	0	0	0	0	0	0	0	0	0	0	0	0	0	0	0	0	1	0	0	0	0	0	1	0	1	1	b1153	orf, hypothetical protein
b1154	0	0	0	0	1	0	0	0	0	0	0	0	0	0	0	0	0	0	0	0	0	0	0	0	0	0	0	0	0	0	0	0	0	1	1	0	0	0	0	0	0	0	0	ycfK	orf, hypothetical protein
b1155	0	0	0	0	0	0	0	0	0	0	0	0	0	0	0	0	0	0	0	0	0	0	0	0	0	0	0	0	0	0	0	0	0	0	0	0	0	0	0	0	0	0	0	b1155	orf, hypothetical protein
b1156	0	0	0	0	0	1	0	0	0	1	1	1	0	0	1	1	0	0	0	0	0	0	0	0	0	0	0	0	0	0	0	1	0	0	0	0	0	0	0	0	0	0	0	ycfA	orf, hypothetical protein
b1157	0	0	0	0	0	0	0	0	0	0	0	0	1	0	1	0	0	0	1	1	1	1	1	1	0	0	0	0	0	0	0	0	0	0	0	0	0	0	0	0	0	0	0	b1157	putative tail fiber protein
b1158	1	0	1	0	0	0	0	0	0	0	0	0	0	1	0	0	0	0	0	0	0	0	0	0	1	0	0	1	1	0	1	1	0	1	1	1	1	1	1	1	1	0	0	pin	inversion of adjacent DNA; at locus of e14 element
b1159	0	0	0	0	0	0	0	0	0	0	0	0	0	0	0	0	0	0	0	0	0	0	0	0	0	0	0	0	0	0	0	0	0	0	0	0	0	0	0	0	0	0	0	mcrA	restriction of DNA at 5-methylcytosine residues; at locus of e14 element
b1160	1	0	1	1	1	1	1	1	1	1	1	1	1	1	1	1	1	0	1	1	1	1	1	1	1	1	1	1	1	1	1	1	0	0	0	0	0	0	0	0	1	1	1	ycgW	orf, hypothetical protein
b1161	0	0	1	0	0	1	1	1	1	1	1	1	0	0	0	0	1	0	0	0	0	0	0	0	1	1	1	1	0	1	1	1	0	0	0	0	0	0	0	0	1	0	0	ycgX	orf, hypothetical protein
b1162	0	0	1	0	0	1	1	1	1	1	1	1	0	0	0	1	1	0	0	1	0	1	1	0	1	1	1	1	0	1	1	1	0	0	0	0	0	0	0	0	1	0	0	ycgE	putative transcriptional regulator
b1163	0	0	1	0	0	1	1	1	1	1	1	1	0	0	0	1	0	0	0	0	0	0	0	0	1	1	1	1	0	1	1	1	0	0	0	0	0	0	0	0	1	0	0	b1163	orf, hypothetical protein
b1164	0	0	0	0	1	1	1	1	1	1	1	1	0	0	1	1	1	0	0	0	1	0	0	0	1	1	1	1	0	1	1	1	0	0	0	0	0	0	0	0	1	0	0	ycgZ	orf, hypothetical protein
b1165	0	1	0	1	1	1	1	1	1	1	1	1	1	0	1	1	1	1	1	1	1	1	1	1	1	1	1	1	1	1	1	1	1	0	0	1	1	1	1	1	1	1	1	ymgA	orf, hypothetical protein
b1166	0	0	0	1	1	1	1	1	1	1	1	1	1	0	1	1	1	0	0	1	1	1	1	1	1	1	1	1	0	1	1	1	1	0	0	0	0	1	1	0	1	1	1	ymgB	orf, hypothetical protein
b1167	0	0	1	0	0	0	0	0	0	0	0	0	0	0	0	0	1	0	0	0	0	0	0	0	1	1	1	1	0	1	1	1	0	0	0	0	0	0	0	0	1	1	1	ymgC	orf, hypothetical protein
b1168	0	0	0	0	1	0	0	0	0	0	0	0	0	0	0	0	0	0	0	0	0	0	0	0	1	1	1	0	0	1	1	1	1	0	1	1	1	1	1	1	1	1	1	b1168	putative proteases
b1169	0	0	0	0	0	1	1	1	1	1	1	1	1	1	1	1	1	0	0	0	0	0	0	0	0	0	0	0	0	0	0	0	0	0	0	0	0	0	0	0	0	0	0	b1169	putative ATP-binding component of a transport system
b1170	0	0	0	0	1	1	1	1	1	1	1	1	1	1	1	1	1	0	0	0	0	0	0	0	1	1	1	1	0	1	1	1	1	1	1	1	1	1	1	1	1	1	1	b1170	putative part of putative ATP-binding component of a transport system
b1171	0	1	1	0	1	1	1	1	1	1	1	1	1	1	1	1	1	0	0	0	0	0	0	0	1	1	1	1	1	1	1	1	1	1	1	1	1	1	1	1	1	1	1	b1171	orf, hypothetical protein
b1172	0	1	1	0	1	1	1	1	1	1	1	1	1	1	1	1	1	0	0	0	0	0	0	0	1	1	1	1	1	1	1	1	1	1	1	1	1	1	1	1	1	1	1	b1172	orf, hypothetical protein
b1173	1	1	0	1	1	1	0	0	0	1	1	1	0	1	1	0	1	1	1	1	1	1	1	1	1	1	1	1	1	1	1	1	1	1	1	1	1	1	1	1	1	1	1	b1173	orf, hypothetical protein
b1177	1	0	1	1	1	1	1	1	1	1	1	1	1	1	1	1	1	1	1	1	1	1	1	1	1	1	1	1	1	1	1	1	1	1	1	1	1	1	1	1	1	1	1	ycgJ	orf, hypothetical protein
b1179	1	1	1	1	1	1	1	1	1	1	1	1	1	1	1	1	1	1	1	1	1	1	1	1	0	1	0	1	1	1	1	1	1	1	1	1	1	1	1	1	1	1	1	ycgL	orf, hypothetical protein
b1182	0	1	1	1	1	1	0	1	1	1	1	1	1	0	1	1	1	1	1	1	1	1	1	1	1	1	1	1	1	1	1	1	1	1	1	1	1	1	1	1	1	1	1	hlyE	hemolysin E
b1183	1	0	1	1	1	1	1	1	1	1	1	1	1	1	1	1	1	1	1	1	0	1	1	1	1	1	1	1	1	1	1	1	1	1	1	1	1	1	1	1	1	1	1	umuD	SOS mutagenesis; error-prone repair; processed to UmuD'; forms complex with UmuC
b1184	1	0	1	1	1	1	1	1	1	1	1	1	1	1	1	1	1	1	1	1	1	1	1	1	1	1	1	1	1	1	1	1	1	1	1	1	1	1	1	1	1	1	1	umuC	SOS mutagenesis and repair
b1190	1	1	1	1	1	1	1	1	1	1	1	1	1	1	1	1	1	1	1	1	1	1	1	1	1	1	1	1	0	1	1	1	1	1	1	1	1	1	1	1	1	1	1	dadX	alanine racemase 2, catabolic
b1195	1	1	1	1	1	1	1	1	1	1	1	1	1	1	1	1	1	1	1	1	1	1	1	1	1	1	1	1	1	1	1	1	1	1	1	1	1	1	1	1	1	0	0	ymgE	orf, hypothetical protein
b1196	0	0	0	0	1	1	1	1	1	1	1	1	1	1	1	0	1	1	1	1	1	1	1	1	1	1	1	1	1	1	1	1	1	1	1	1	1	0	1	1	1	0	0	ycgY	orf, hypothetical protein
b1197	1	1	0	1	1	1	1	1	1	1	1	1	1	1	1	0	1	1	1	1	1	1	1	1	1	1	1	1	1	1	1	1	1	1	1	1	1	0	1	1	1	0	0	treA	trehalase, periplasmic
b1198	1	0	0	0	1	1	1	1	1	1	1	1	0	1	1	1	1	1	1	1	1	1	1	1	1	1	1	1	1	1	1	1	1	1	1	1	1	0	1	1	1	1	1	ycgC	putative PTS system enzyme I
b1199	1	0	0	0	1	1	1	1	1	1	1	1	0	1	1	1	1	1	1	1	1	1	1	1	1	1	1	1	1	1	1	1	1	1	1	1	1	0	1	1	1	1	1	b1199	putative dihydroxyacetone kinase (EC 2.7.1.2)
b1200	0	0	0	0	1	1	1	1	1	1	1	1	0	1	1	1	1	1	1	1	1	1	1	1	1	1	1	1	1	1	1	1	1	1	1	1	1	0	1	1	1	1	1	b1200	putative dihydroxyacetone kinase (EC 2.7.1.2)
b1201	0	0	0	0	1	1	1	1	1	1	1	1	0	1	1	1	1	1	1	1	1	1	1	1	1	1	1	1	1	1	1	1	1	1	1	1	1	0	1	1	1	1	1	b1201	putative sensor-type regulator
b1202	1	0	0	0	1	0	0	0	1	1	1	1	0	1	1	0	1	1	1	1	1	1	1	1	1	1	1	1	1	1	1	1	1	1	1	1	1	0	1	1	1	1	1	b1202	putative adhesion and penetration protein
b1217	1	0	1	1	1	0	1	1	1	1	1	1	1	1	1	1	1	1	1	1	1	1	1	1	1	1	1	1	1	1	1	0	1	1	1	1	1	1	1	1	1	1	1	chaB	cation transport regulator
b1224	1	1	1	1	1	0	1	1	1	1	1	1	1	1	1	1	1	1	1	1	1	1	1	1	1	1	1	1	1	1	1	1	1	1	1	1	1	1	1	1	1	1	1	narG	nitrate reductase 1, alpha subunit
b1225	1	1	1	1	1	1	1	1	1	1	1	1	1	1	1	1	1	1	1	1	1	1	1	1	0	1	0	1	1	1	0	1	0	1	1	1	1	1	1	1	1	1	1	narH	nitrate reductase 1, beta subunit
b1228	1	1	1	1	1	0	1	1	0	1	1	1	1	1	1	1	1	1	1	1	1	1	1	1	0	1	0	1	1	1	1	0	1	1	1	1	1	1	1	1	1	1	1	b1228	orf, hypothetical protein
b1238	1	1	1	1	1	1	1	1	1	1	1	1	1	1	1	1	1	1	1	1	1	1	1	1	1	1	1	1	0	1	1	1	1	1	1	1	1	1	1	1	1	1	1	tdk	thymidine kinase
b1239	0	0	0	0	1	1	1	1	1	1	1	1	1	1	1	1	1	1	1	1	1	1	1	1	0	0	0	0	0	0	0	0	0	0	0	0	0	0	0	0	0	0	0	ychG	orf, hypothetical protein
b1240	0	1	0	1	1	1	1	1	1	1	1	1	1	1	1	1	1	1	1	1	1	1	1	1	1	1	1	1	1	1	1	1	1	0	0	0	1	1	1	1	1	1	1	b1240	orf, hypothetical protein
b1241	1	1	1	1	1	1	1	1	1	1	1	1	1	1	1	1	1	1	1	1	1	1	1	1	1	1	1	1	0	1	1	1	1	1	1	1	1	1	1	1	1	1	1	adhE	CoA-linked acetaldehyde dehydrogenase and iron-dependent alcohol dehydrogenase
b1242	0	1	1	1	1	1	1	1	1	1	1	1	1	1	1	1	1	1	1	1	1	1	1	1	1	1	1	1	0	1	1	1	1	1	1	1	1	1	1	1	1	1	1	ychE	putative channel protein
b1251	1	1	1	1	1	1	1	1	1	1	1	1	1	1	1	1	1	1	1	1	1	1	1	1	1	0	1	0	1	1	1	1	1	1	1	1	1	1	1	1	1	1	1	yciI	orf, hypothetical protein
b1257	1	1	0	1	1	1	1	1	1	1	1	1	1	0	1	1	1	1	1	1	1	1	1	1	1	0	1	0	1	1	1	1	1	1	1	1	1	1	1	1	1	1	1	yciE	orf, hypothetical protein
b1258	0	1	0	1	1	1	1	0	1	1	0	1	1	0	1	0	1	1	1	1	1	1	1	1	1	1	1	1	1	1	1	1	1	1	1	1	1	1	1	1	1	1	1	yciF	putative structural proteins
b1259	1	1	1	1	1	0	0	0	0	0	0	0	0	0	0	0	1	0	0	0	1	0	0	0	1	1	1	1	1	1	1	1	1	1	1	1	1	1	1	1	1	1	1	yciG	orf, hypothetical protein
b1263	1	1	1	1	1	0	1	1	1	1	1	1	1	1	1	1	1	1	1	1	1	1	1	1	1	1	1	1	1	1	1	1	1	1	1	1	1	1	1	1	1	1	1	trpD	anthranilate synthase component II, glutamine amidotransferase and phosphoribosylanthranilate transferase
b1264	1	1	1	1	1	0	1	1	1	1	1	1	1	1	1	1	1	1	1	1	1	1	1	1	1	1	1	1	1	1	1	1	1	1	1	1	1	1	1	1	1	1	1	trpE	anthranilate synthase component I
b1268	0	0	1	1	0	1	1	1	1	1	1	1	1	1	1	1	1	1	1	1	1	1	1	1	1	1	1	1	1	1	1	1	0	0	0	0	0	0	0	0	0	0	0	yciQ	orf, hypothetical protein
b1269	1	1	1	1	1	0	1	1	1	1	1	1	1	1	1	1	1	1	1	1	1	1	1	1	1	1	1	1	1	1	1	1	1	1	1	1	1	1	1	1	1	1	1	yciL	orf, hypothetical protein
b1283	0	1	1	1	1	1	1	1	1	1	1	1	1	1	1	1	1	1	1	1	1	1	1	1	1	1	1	1	1	1	1	1	1	1	1	1	1	1	1	1	1	1	1	osmB	osmotically inducible lipoprotein
b1289	0	0	1	0	1	1	1	1	1	1	1	1	1	1	1	1	1	1	1	1	1	1	1	1	1	1	1	1	1	1	1	1	1	1	1	1	1	1	1	1	1	1	1	ycjD	orf, hypothetical protein
b1300	1	1	1	1	1	1	1	1	1	1	1	1	1	1	1	1	1	1	1	1	1	1	1	1	1	1	1	1	0	1	1	1	1	1	1	1	1	1	1	1	1	1	1	aldH	aldehyde dehydrogenase, prefers NADP over NAD
b1302	1	1	1	1	1	0	1	1	1	1	1	1	1	1	1	1	1	1	1	1	1	1	1	1	1	1	1	1	1	1	1	1	1	1	1	1	1	1	1	1	1	1	1	goaG	4-aminobutyrate aminotransferase
b1308	1	1	1	1	1	1	1	1	1	1	1	1	1	1	1	1	1	1	1	1	0	1	1	1	1	1	1	1	1	1	1	1	1	1	1	1	1	1	1	1	1	1	1	pspE	phage shock protein
b1309	1	0	1	1	0	0	0	0	0	0	0	0	0	0	0	0	1	0	0	0	1	0	0	0	1	1	1	1	1	1	1	1	1	1	1	1	1	1	1	1	0	1	1	ycjM	putative polysaccharide hydrolase
b1310	0	0	1	1	0	0	0	0	0	0	0	0	0	0	0	0	1	0	0	0	0	0	0	0	1	1	1	1	1	0	0	1	1	1	1	1	1	1	1	1	0	1	1	b1310	putative transport periplasmic protein
b1311	0	0	1	1	0	0	0	0	0	0	0	0	0	0	0	0	1	0	0	0	0	0	0	0	1	1	1	1	0	1	0	1	1	1	1	1	1	1	1	1	0	1	1	ycjO	putative binding-protein dependent transport protein
b1312	0	0	1	1	0	0	0	0	0	0	0	0	0	0	0	0	1	0	0	0	0	0	0	0	1	1	1	1	1	1	0	1	1	1	1	1	1	1	1	1	0	1	1	ycjP	putative transport system permease protein
b1313	0	0	1	1	0	0	0	0	0	0	0	0	0	0	0	0	1	0	0	0	0	0	0	0	1	1	1	1	1	1	0	1	1	1	1	1	1	1	1	1	0	1	1	ycjQ	putative oxidoreductase
b1314	0	0	1	1	0	0	0	0	0	0	0	0	0	0	0	0	1	0	0	0	0	0	0	0	1	1	1	1	1	1	0	1	1	1	1	1	1	1	1	1	0	1	1	b1314	putative transient receptor potential locus
b1315	0	0	1	1	0	0	0	0	0	0	0	0	0	0	0	0	1	0	0	0	1	0	0	1	1	1	1	1	1	1	0	1	1	1	1	1	1	1	1	1	0	1	1	ycjS	putative dehydrogenase
b1316	0	0	1	1	0	0	0	0	0	0	0	0	0	0	0	0	1	0	0	0	0	0	0	0	1	1	1	1	1	1	0	1	1	1	1	1	1	1	1	1	0	1	1	ycjT	orf, hypothetical protein
b1317	0	0	1	1	0	0	0	0	0	0	0	0	0	0	0	0	1	0	0	0	0	0	0	0	1	1	1	1	1	1	0	1	1	1	1	1	1	1	1	1	1	1	1	ycjU	putative beta-phosphoglucomutase
b1318	0	1	1	1	0	0	1	1	1	1	1	1	0	0	0	0	1	0	0	1	1	0	1	0	1	1	1	1	1	1	0	1	1	1	1	1	1	1	1	1	1	1	1	ycjV	putative ATP-binding component of a transport system
b1319	0	0	1	1	0	1	1	1	1	1	1	1	1	0	0	0	1	1	1	1	1	1	1	1	1	1	1	1	1	1	1	1	1	1	1	1	1	1	1	1	1	1	1	ompG	outer membrane protein
b1320	0	0	1	1	0	1	1	1	1	1	1	1	1	0	0	0	1	1	1	1	1	1	1	1	1	1	1	1	1	1	1	1	1	1	1	1	0	1	1	1	1	1	1	ycjW	putative LACI-type transcriptional regulator

b1321	1	1	1	1	1	1	0	1	0	1	1	1	0	1	0	0	1	0	1	1	1	1	1	1	1	1	0	1	1	1	1	1	1	1	1	1	1	1	1	1	1	1	ycjX	putative EC 2.1 enzymes
b1327	0	0	1	1	1	1	1	1	1	0	1	1	0	1	1	1	1	1	1	1	1	1	1	1	0	0	1	1	1	1	1	1	1	1	1	1	0	0	1	0	1	1	1	b1327	orf, hypothetical protein
b1328	1	0	1	1	0	1	1	1	1	0	1	1	1	1	1	1	1	1	1	1	1	1	1	1	0	1	1	1	1	1	1	1	1	1	1	1	1	1	1	1	0	1	1	ycjZ	putative transcriptional regulator LYSR-type
b1329	1	1	1	1	1	1	1	1	1	0	1	1	1	1	1	1	1	1	1	1	1	1	1	1	0	1	1	1	1	1	1	1	1	1	1	1	1	1	1	1	1	1	1	b1329	putative transport periplasmic protein
b1330	1	1	1	1	1	1	1	1	1	0	1	1	1	1	1	1	1	1	1	1	1	1	1	1	0	1	1	1	1	1	1	1	1	1	1	1	1	1	1	1	1	1	1	b1330	orf, hypothetical protein
b1332	1	1	1	1	1	1	1	0	0	0	0	1	0	1	1	0	1	1	1	1	1	1	1	1	0	1	0	0	1	0	1	1	1	1	1	1	1	1	1	1	1	1	1	ynaJ	orf, hypothetical protein
b1333	1	1	1	1	1	1	1	1	1	0	0	1	1	1	1	1	1	1	1	1	1	1	1	1	0	1	1	1	1	1	1	1	1	1	1	1	1	0	1	1	1	1	1	ydaA	orf, hypothetical protein
b1334	1	1	1	1	1	1	1	1	1	0	1	1	1	1	1	1	0	1	1	1	1	1	1	1	0	1	1	1	1	1	1	1	1	1	1	1	1	0	1	1	1	1	1	fnr	transcriptional regulation of aerobic, anaerobic respiration, osmotic balance
b1335	1	1	1	1	1	1	1	1	1	0	1	1	1	1	1	1	0	1	1	1	1	1	1	1	0	1	1	1	1	1	1	1	1	1	1	1	1	0	1	1	1	1	1	ogt	O-6-alkylguanine-DNA/cysteine-protein methyltransferase
b1336	0	0	1	1	1	1	1	1	1	0	1	1	1	1	1	1	0	1	1	1	1	1	1	1	0	1	1	1	1	1	1	1	0	0	0	0	0	0	0	0	0	0	0	ydaH	putative pump protein (transport)
b1337	0	0	1	1	1	1	1	1	1	0	1	1	0	1	1	1	0	1	1	1	1	1	1	1	0	1	1	1	1	0	1	1	0	0	0	0	0	0	1	0	1	1	0	b1337	orf, hypothetical protein
b1338	0	0	1	1	1	1	1	1	1	0	1	1	0	1	1	1	0	1	1	1	1	1	1	1	0	1	1	1	1	0	1	0	0	0	0	0	0	0	0	0	0	0	0	ydaJ	putative aminohydrolase (EC 3.5.1.14)
b1339	0	0	1	1	1	1	1	1	1	0	1	1	0	1	1	1	0	1	1	1	1	1	1	1	0	1	1	1	1	1	1	1	0	0	0	0	0	0	0	0	0	0	0	ydaK	putative transcriptional regulator LYSR-type
b1340	1	1	1	1	1	0	1	1	1	0	1	1	1	1	1	1	0	1	1	1	1	1	1	1	0	1	1	1	1	1	1	1	1	1	1	1	1	0	1	1	1	1	1	ydaL	orf, hypothetical protein
b1341	1	1	1	1	1	1	1	1	1	0	1	1	1	1	1	1	0	1	1	1	1	1	1	1	0	1	1	1	1	1	1	1	1	1	1	1	1	0	1	1	1	1	1	b1341	orf, hypothetical protein
b1342	1	1	1	1	1	1	1	1	1	0	1	1	1	1	1	1	0	1	1	1	1	1	1	1	0	1	1	1	1	1	1	1	1	1	1	1	1	0	1	1	1	1	1	b1342	orf, hypothetical protein
b1343	1	1	1	1	1	1	1	1	1	0	1	1	1	1	1	1	0	1	1	1	1	1	1	1	0	1	1	1	1	1	1	1	1	1	1	1	1	0	1	1	1	1	1	dbpA	ATP-dependent RNA helicase
b1344	1	1	1	1	1	1	1	1	1	0	1	1	1	1	1	1	0	1	1	1	1	1	1	1	0	1	1	1	1	1	1	1	1	1	1	1	1	0	1	1	1	1	1	ydaO	orf, hypothetical protein
b1345	0	1	1	0	1	1	1	1	1	0	1	1	1	1	1	1	0	0	1	1	1	1	1	1	0	1	1	1	1	1	1	1	1	1	1	1	1	1	1	1	0	1	1	b1345	putative transposase
b1346	0	0	0	0	0	0	0	0	0	0	0	0	1	1	1	1	0	0	0	0	0	0	0	0	0	0	1	1	1	0	0	1	0	0	1	1	1	1	1	1	0	1	1	ydaQ	orf, hypothetical protein
b1347	0	0	0	1	0	0	0	0	0	0	0	0	1	1	1	1	0	0	0	0	0	0	0	0	0	1	1	1	1	1	1	1	1	1	0	0	1	1	0	1	0	1	1	ydaC	orf, hypothetical protein
b1348	0	0	0	0	0	0	0	0	0	0	0	0	1	0	0	0	0	0	0	0	0	0	0	0	0	0	0	0	1	0	0	1	0	0	0	0	0	0	0	0	0	1	0	lar	restriction alleviation and modification enhancement
b1349	0	0	0	0	0	0	0	0	0	0	0	0	0	0	0	0	0	0	0	0	0	0	0	0	0	0	0	0	0	0	0	0	0	0	0	0	0	0	0	0	0	0	0	recT	recombinase, DNA renaturation
b1350	0	1	0	1	0	0	0	0	0	0	0	0	1	1	1	1	1	1	1	1	1	1	1	1	0	0	0	0	0	0	0	1	0	0	0	0	0	0	0	0	0	0	0	recE	exonuclease VIII, ds DNA exonuclease, 5' --> 3' specific
b1351	0	0	0	0	0	0	0	0	0	0	0	0	1	0	0	0	0	1	1	1	1	1	1	1	0	1	1	1	1	1	0	1	0	0	1	1	1	1	1	1	0	0	0	racC	defective prophage rac; contains recE and oriJ
b1351a	0	0	0	0	0	0	0	0	0	0	0	0	0	0	0	0	1	1	1	1	1	1	1	1	0	0	1	0	1	1	1	1	0	0	0	1	1	1	1	1	0	0	0	ydaE	Uncharacterized conserved protein
b1352	0	0	0	0	0	0	0	0	0	0	0	0	0	0	0	0	0	0	0	0	0	0	1	0	0	0	0	0	1	0	0	1	0	0	1	1	1	1	1	1	0	0	0	ydaD	orf, hypothetical protein
b1353	0	0	0	0	0	0	0	0	0	0	0	0	0	0	0	0	0	0	0	0	0	0	0	0	0	0	0	0	0	0	0	0	0	0	0	0	0	0	0	0	0	0	0	sieB	phage superinfection exclusion protein
b1353a	0	1	1	1	0	1	0	1	1	0	1	0	1	0	1	1	1	1	0	1	1	1	1	1	0	0	1	0	1	1	0	1	1	1	1	0	0	1	1	0	0	1	1	ydaF	Uncharacterized conserved protein
b1354	0	1	1	1	1	1	1	1	1	0	1	0	1	0	1	1	1	1	1	1	1	0	1	1	0	1	1	1	1	1	0	1	1	1	1	0	1	1	1	1	0	1	1	b1354	orf, hypothetical protein
b1355	0	0	0	0	0	0	0	0	0	0	0	0	0	0	0	0	0	0	0	0	0	0	0	0	0	0	0	0	0	0	0	0	0	0	0	0	0	0	0	0	0	0	0	b1355	orf, hypothetical protein
b1356	0	0	0	0	0	0	0	0	0	0	0	0	0	0	0	0	0	0	0	0	0	0	0	0	0	0	0	0	0	0	0	0	0	0	0	0	0	0	0	0	0	0	0	ydaR	orf, hypothetical protein
b1357	0	0	0	0	0	0	0	0	0	0	0	0	0	0	0	0	0	0	0	0	0	0	0	0	0	0	0	0	0	0	0	1	0	0	0	0	0	0	0	0	0	0	0	ydaS	orf, hypothetical protein
b1358	0	0	1	1	0	1	1	1	1	0	1	0	1	0	1	1	1	1	1	1	1	1	1	1	0	1	1	0	1	1	1	1	1	1	1	1	1	1	1	1	0	1	1	ydaT	orf, hypothetical protein
b1359	0	0	1	0	0	0	0	0	0	0	0	0	0	0	0	0	0	0	0	0	0	0	0	0	0	0	1	0	1	1	1	1	1	1	1	1	1	1	1	1	1	1	0	ydaU	orf, hypothetical protein
b1360	0	0	1	1	1	1	1	1	1	1	1	1	1	0	1	1	1	1	1	1	1	1	1	1	1	1	1	1	1	1	1	0	1	1	1	1	1	1	1	1	1	1	1	b1360	putative DNA replication factor
b1361	0	1	1	1	0	0	0	0	0	0	0	0	0	0	0	0	1	0	0	1	0	1	1	1	0	0	0	0	1	0	0	1	0	0	0	0	0	0	0	0	0	1	1	ydaW	orf, hypothetical protein
b1362	0	1	1	1	1	1	1	1	1	1	1	1	0	1	0	0	1	1	1	1	1	1	1	1	1	1	1	1	1	1	1	1	0	0	0	0	0	0	0	0	1	0	0	b1362	putative Rac prophage endopeptidase
b1363	0	0	0	1	0	0	0	0	0	0	0	0	0	0	0	0	0	0	0	0	0	0	0	0	1	0	1	1	1	1	1	1	0	0	0	0	0	0	0	0	0	0	0	trkG	trk system potassium uptake; part of Rac prophage
b1364	0	1	0	1	1	1	1	1	1	1	1	1	1	0	1	1	1	1	1	1	1	1	1	1	1	1	1	1	1	1	1	1	1	0	0	1	1	1	1	1	1	1	1	b1364	orf, hypothetical protein
b1365	0	0	0	1	1	1	1	1	1	0	1	1	0	0	1	1	0	1	1	1	1	1	1	1	0	1	1	1	1	1	1	1	0	0	0	0	0	0	0	0	0	0	0	b1365	orf, hypothetical protein
b1366	0	0	0	0	0	0	0	0	0	0	0	0	0	0	0	0	0	0	0	0	0	0	0	0	0	0	0	0	0	0	0	0	0	0	0	0	0	0	0	0	0	0	0	ydaY	orf, hypothetical protein
b1367	0	0	0	0	0	0	0	0	0	0	0	0	0	0	0	0	0	0	0	0	0	0	0	1	0	0	0	0	0	0	0	1	0	0	0	0	0	0	0	0	0	0	0	b1367	orf, hypothetical protein
b1368	0	0	0	0	0	0	0	0	0	0	0	0	0	0	0	0	0	0	0	0	0	0	0	0	0	0	0	0	0	0	0	0	0	0	0	0	0	0	0	0	0	0	0	b1368	putative alpha helix protein
b1369	0	1	1	1	1	0	0	1	1	1	1	1	1	1	1	1	1	1	1	1	1	1	1	1	1	1	0	0	1	0	1	1	0	1	1	1	1	1	1	1	0	0	0	b1369	orf, hypothetical protein
b1371	0	0	0	0	0	0	0	0	0	0	0	0	0	0	0	0	0	0	0	0	0	0	0	0	0	0	0	0	0	0	0	0	0	0	0	0	0	0	0	0	0	0	0	b1371	orf, hypothetical protein
b1372	0	1	1	1	1	0	0	1	1	1	1	1	1	0	0	1	1	1	1	1	1	1	1	1	0	0	1	1	1	1	1	1	1	1	0	0	0	0	0	0	0	1	1	b1372	putative membrane protein
b1373	0	0	1	1	1	1	1	1	1	1	1	1	1	0	0	0	1	1	1	1	1	1	1	1	0	1	1	1	1	0	1	1	0	0	0	0	0	0	0	0	0	1	1	ynaC	orf, hypothetical protein
b1374	0	0	0	0	0	0	0	0	0	0	0	0	0	0	0	0	0	0	0	0	0	0	0	0	0	0	0	0	1	0	0	0	0	0	0	0	0	0	0	0	0	0	0	b1374	putative transposon resolvase
b1375	0	0	0	0	0	0	0	0	0	0	0	0	0	0	0	0	0	0	0	0	0	0	0	0	0	0	1	1	1	0	0	0	0	0	0	0	0	0	0	0	0	0	0	ynaE	orf, hypothetical protein
b1376	1	1	1	1	1	1	1	1	1	1	1	1	1	1	1	1	1	1	1	1	1	0	1	1	1	1	1	1	1	1	1	1	1	1	1	1	1	0	1	1	1	1	1	ynaF	putative filament protein
b1377	1	1	1	1	1	1	1	1	1	1	1	1	1	1	1	1	1	1	1	1	1	0	1	1	1	1	0	1	1	1	1	1	1	1	1	1	1	0	1	1	1	1	1	b1377	putative outer membrane protein
b1378	1	1	1	1	1	1	1	1	1	1	1	1	1	1	1	1	1	1	1	1	1	0	1	1	1	1	1	1	1	1	1	1	1	1	1	1	1	0	1	1	1	1	1	ydbK	putative oxidoreductase, Fe-S subunit
b1379	1	1	1	1	1	1	1	1	1	1	1	1	1	1	1	1	1	1	1	1	1	0	1	1	1	1	1	1	1	1	1	1	1	1	1	1	1	0	1	1	1	1	1	hslJ	heat shock protein hslJ
b1380	1	1	1	1	1	1	1	1	1	1	1	1	1	1	1	1	1	1	1	1	1	0	1	1	1	1	1	1	1	1	1	1	1	1	1	1	1	0	1	1	1	1	1	ldhA	fermentative D-lactate dehydrogenase, NAD-dependent
b1381	1	1	1	1	1	1	1	1	1	1	1	1	1	1	1	1	1	1	1	1	1	0	1	1	1	1	1	1	1	1	1	1	1	1	1	1	1	0	1	1	1	1	1	ydbH	orf, hypothetical protein
b1382	1	1	1	1	1	1	1	1	1	1	1	1	1	1	1	1	1	1	1	1	1	0	1	1	1	1	1	1	1	1	1	1	1	1	1	1	1	0	1	1	1	1	1	ynbE	orf, hypothetical protein
b1383	1	1	1	1	1	1	1	0	0	1	1	1	0	1	1	0	1	1	1	1	1	0	0	1	1	1	1	1	1	1	1	1	1	1	1	1	1	0	1	1	1	1	1	ydbL	orf, hypothetical protein
b1384	0	0	1	0	1	0	0	0	0	0	0	0	0	0	0	0	0	0	0	0	0	0	0	0	0	1	0	1	1	1	1	1	0	0	0	0	0	0	0	0	0	0	0	feaR	regulatory protein for 2-phenylethylamine catabolism
b1385	0	0	0	0	1	0	0	0	0	1	0	0	0	0	0	0	1	0	0	0	1	0	1	1	0	1	0	1	1	1	1	1	0	0	0	0	1	0	0	0	0	0	0	feaB	phenylacetaldehyde dehydrogenase
b1386	0	0	0	0	1	0	0	0	0	0	0	0	0	0	0	0	0	0	0	0	0	0	0	0	0	1	0	1	1	1	1	1	0	0	0	0	0	0	0	0	0	0	0	tynA	copper amine oxidase (tyramine oxidase)
b1387	0	0	0	0	1	0	0	0	0	0	0	0	0	0	0	0	0	0	0	0	0	0	0	0	0	1	0	1	1	1	1	1	0	0	0	0	0	0	0	0	0	0	0	maoC	putative aldehyde dehydrogenase
b1388	0	0	0	0	1	0	0	0	0	0	0	0	0	0	0	0	0	0	0	0	0	0	0	1	0	1	0	1	1	1	1	1	0	0	0	0	0	0	0	0	0	0	0	ydbO	orf, hypothetical protein
b1389	0	0	0	0	1	0	0	0	0	0	0	0	0	0	0	0	0	0	0	0	1	0	0	0	0	1	0	1	1	1	1	1	0	0	0	0	0	0	0	0	0	0	0	ynbF	orf, hypothetical protein
b1390	0	0	0	0	1	0	0	0	0	0	0	0	0	0	0	0	0	0	0	0	0	0	0	0	0	1	0	1	1	1	1	1	0	0	0	0	0	0	0	0	0	0	0	ydbP	orf, hypothetical protein
b1391	0	0	0	1	1	1	1	1	1	0	1	1	0	0	1	1	0	0	1	1	1	1	1	1	0	1	1	1	1	1	1	1	0	0	0	0	0	0	0	0	0	0	0	b1391	orf, hypothetical protein
b1392	0	0	0	0	1	0	0	0	0	0	0	0	0	0	0	0	0	0	0	0	0	0	0	0	1	1	1	1	1	1	1	1	0	0	0	0	0	0	0	0	0	0	0	b1392	putative oxidoreductase
b1393	0	1	0	0	1	1	0	1	1	1	1	1	1	0	0	1	1	0	0	1	1	1	1	1	1	1	0	1	1	1	1	1	0	0	0	0	0	0	0	0	0	0	0	ydbS	putative enzyme
b1394	0	0	0	0	1	0	0	0	0	0	0	0	0	0	0	0	0	0	0	0	0	0	0	0	1	1	0	1	1	1	1	1	0	0	0	0	0	0	0	0	0	0	0	b1394	putative enzyme
b1395	0	0	0	0	1	0	0	0	0	0	0	0	0	0	0	0	0	0	0	0	0	0	0	0	1	0	0	1	1	1	1	1	0	0	0	0	0	0	0	0	0	0	0	ydbU	putative enzyme
b1396	0	0	0	0	1	0	0	0	0	0	0	0	0	0	0	0	0	0	0	0	1	0	0	0	1	0	0	1	1	1	1	1	0	0	0	0	0	0	0	0	0	0	0	b1396	orf, hypothetical protein
b1397	0	0	0	0	1	0	0	0	0	0	0	0	0	0	0	0	0	0	0	0	0	0	0	0	1	0	0	1	1	1	1	1	0	0	0	0	0	0	0	0	0	0	0	b1397	putative acyltransferase
b1398	0	0	0	0	1	0	0	0	0	0	0	0	0	0	0	0	0	0	0	0	1	1	0	1	1	1	0	1	1	1	1	1	0	0	0	0	0	0	0	0	0	0	0	b1398	orf, hypothetical protein
b1399	0	0	0	0	1	0	0	0	0	0	0	0	0	0	0	0	0	0	0	0	0	0	0	0	1	1	0	1	1	1	0	1	0	0	0	0	0	0	0	0	0	0	0	b1399	orf, hypothetical protein
b1400	0	0	0	0	1	0	0	0	0	0	0	0	0	0	0	0	0	0	0	0	0	0	0	0	1	1	0	1	1	1	1	1	0	0	0	0	0	0	0	0	0	0	0	b1400	putative transferase
b1401	0	0	0	0	0	0	0	0	0	0	0	0	0	0	0	0	0	0	0	0	0	0	0	0	0	0	0	0	0	0	0	0	0	0	0	0	0	0	0	0	0	0	0	ydbA_1	split orf
b1405	0	0	0	0	0	0	0	0	0	0	0	0	0	0	0	0	0	0	0	0	0	0	0	0	0	0	0	0	0	0	0	0	0	0	0	0	0	0	0	0	0	0	0	ydbA_2	split orf
b1406	0	1	0	0	1	0	0	0	0	0	0	0	0	0	0	0	0	0	0	0	0	0	0	0	1	1	1	1	1	1	1	1	0	0	0	0	0	0	0	0	0	0	0	ydbC	putative dehydrogenase
b1407	0	1	0	0	0	0	0	0	0	0	0	0	0	0	0	0	0	0	0	0	0	0	0	0	1	1	1	1	1	1	1	1	1	1	1	1	1	0	1	1	0	0	0	ydbD	orf, hypothetical protein
b1408	1	0	0	0	1	1	1	1	1	1	1	1	1	1	1	1	1	1	1	1	1	0	0	1	1	1	1	1	1	1	1	1	1	1	1	1	0	0	1	1	0	1	1	b1408	probable enzyme
b1409	1	0	1	0	1	0	0	0	1	1	0	1	0	0	0	0	1	1	1	1	0	0	0	1	0	0	0	0	0	0	0	0	1	1	1	1	1	0	1	1	0	1	1	b1409	putative phosphatidate cytidiltransferase
b1410	1	0	1	1	1	1	1	1	1	1	1	1	1	1	1	1	1	1	1	1	1	0	1	1	1	1	1	1	1	1	1	0	1	1	1	1	1	0	1	1	0	1	1	b1410	orf, hypothetical protein
b1411	1	0	1	1	1	1	1	1	1	1	1	1	1	1	1	1	1	1	1	1	1	0	1	1	1	1	1	1	1	1	1	1	1	1	1	1	1	0	1	1	0	1	1	ynbD	putative enzymes
b1412	1	1	1	1	1	1	1	1	1	1	1	1	1	1	1	1	1	1	1	1	1	0	1	1	1	1	1	1	1	1	1	1	1	1	1	1	0	0	1	1	1	1	1	acpD	acyl carrier protein phosphodiesterase
b1413	1	1	1	1	1	0	1	1	1	1	1	1	1	1	1	1	1	1	1	1	1	0	1	1	1	1	1	1	1	1	1	1	1	1	1	1	1	0	1	1	1	1	1	hrpA	helicase, ATP-dependent
b1414	0	1	1	1	1	1	1	1	1	1	1	1	1	1	1	1	1	1	1	1	1	0	1	1	1	1	1	1	1	1	1	1	0	1	1	1	1	0	1	1	1	1	1	ydcF	orf, hypothetical protein
b1415	1	0	1	0	0	1	1	1	1	1	1	1	1	1	1	1	0	1	1	1	1	0	1	1	1	1	1	1	0	1	1	0	0	1	1	1	1	0	1	1	1	1	1	aldA	aldehyde dehydrogenase, NAD-linked
b1416.m	1	1	1	0	1	1	1	1	1	1	1	1	1	1	1	1	0	1	1	1	0	0	1	1	1	1	1	1	1	1	1	0	0	1	1	1	1	0	1	1	1	1	1	gapC	Glyceraldehyde-3-phosphate dehydrogenase
b1418	1	1	1	0	1	1	1	1	1	1	1	1	1	1	1	1	0	1	1	1	1	1	1	1	1	1	1	1	1	1	1	0	0	1	1	1	1	0	1	1	1	1	1	cybB	cytochrome b(561)
b1419	1	1	1	0	1	1	1	1	1	1	1	1	1	0	1	1	0	1	1	1	1	1	1	1	1	1	1	1	1	1	1	1	1	1	1	1	1	0	1	1	1	1	1	ydcA	orf, hypothetical protein
b1420	1	0	1	0	1	1	1	1	1	1	1	1	1	1	1	1	0	1	1	1	1	1	1	1	1	1	1	1	1	1	1	0	0	1	1	1	1	0	1	1	1	1	1	-	orf, hypothetical protein
b1421	1	0	1	1	1	1	1	1	0	1	1	1	1	1	1	1	0	1	1	1	1	1	1	1	1	1	1	1	1	1	1	1	1	1	1	1	1	0	1	1	1	1	1	trg	methyl-accepting chemotaxis protein III, ribose sensor receptor
b1422	1	1	1	1	1	1	1	1	1	1	1	1	1	1	1	1	0	1	1	1	1	1	1	1	1	1	1	1	1	1	1	1	1	1	1	1	1	0	1	1	1	1	1	-	putative transcriptional regulator LYSR-type
b1423	1	1	1	1	1	1	1	1	1	1	1	1	1	1	1	1	0	1	1	1	1	1	1	1	1	1	1	1	1	1	1	1	1	1	1	1	1	0	1	1	1	1	1	-	orf, hypothetical protein
b1424	1	1	1	1	1	1	1	1	1	1	1	1	1	1	1	1	0	1	1	1	1	1	1	1	1	1	1	1	1	1	1	1	1	1	1	1	1	0	1	1	1	1	1	ydcG	putative glycoprotein
b1425	1	1	1	1	1	1	1	1	1	1	1	1	1	1	1	1	0	1	1	1	1	1	1	1	1	1	1	1	1	1	1	1	1	1	1	1	1	0	1	1	1	1	1	b1425	orf, hypothetical protein
b1426	1	1	1	1	1	1	1	1	1	1	1	1	1	1	1	1	0	1	1	1	1	1	1	1	1	1	1	1	1	1	1	1	1	1	1	1	1	0	1	1	1	1	1	ydcH	orf, hypothetical protein
b1427	1	1	1	1	1	1	1	1	1	1	1	1	1	1	1	1	0	1	1	1	1	1	1	1	1	1	1	1	1	1	1	1	1	1	1	1	1	0	1	1	1	1	1	rimL	acetylation of N-terminal serine of 30S ribosomal subunit protein L7; acetyl transferase
b1428	1	1	1	1	1	1	1	1	1	1	1	1	1	1	1	1	0	1	1	1	1	1	1	1	1	1	1	1	1	1	1	1	1	1	1	1	1	0	1	1	1	1	1	-	orf, hypothetical protein
b1429	1	1	1	1	1	1	1	1	1	1	1	1	1	1	1	1	0	1	1	1	1	1	1	1	1	1	1	1	1	1	1	1	1	1	1	1	1	0	1	1	1	1	1	tehA	tellurite resistance
b1430	1	1	1	1	1	1	1	1	1	1	1	1	1	1	1	1	0	1	1	1	1	1	1	1	1	1	1	1	1	1	1	1	1	1	1	1	1	0	1	1	1	1	1	tehB	tellurite resistance
b1431	1	1	1	1	1	1	1	1	1	1	1	1	1	1	1	1	0	1	1	1	1	1	1	1	1	1	1	1	1	1	1	1	1	1	1	1	1	0	1	1	1	1	1	b1431	orf, hypothetical protein
b1432	1	0	1	1	1	1	1	1	1	1	1	1	1	1	1	1	1	1	1	1	1	1	1	1	0	0	0	0	0	0	0	0	1	1	1	1	1	1	1	1	1	1	1	b1432	putative virulence protein
b1433	1	0	1	1	1	1	0	0	0	0	0	1	0	0	1	0	0	0	0	0	0	0	0	0	1	1	1	1	1	1	1	1	0	0	0	0	0	0	0	0	0	0	0	b1433	putative membrane transport protein
b1434	1	0	1	1	1	0	0	0	0	0	0	0	0	0	1	0	0	0	0	0	0	0	0	0	1	1	1	1	1	1	1	1	0	0	0	0	0	0	0	0	0	0	0	ydcN	orf, hypothetical protein
b1435	1	1	1	1	1	0	0	0	0	0	0	0	0	0	1	0	0	0	0	0	0	0	0	0	1	1	1	1	1	1	1	1	0	0	0	0	0	0	0	0	0	0	0	ydcP	putative collagenase

b1436	1	1	1	1	1	1	0	1	0	0	0	0	0	0	1	0	0	0	0	0	0	0	0	0	1	1	1	1	1	1	1	1	0	0	0	0	0	0	0	0	0	0	0	-	orf, hypothetical protein
b1437	1	1	1	1	1	0	1	1	1	0	1	1	1	0	1	1	1	0	0	1	1	1	1	1	1	1	1	1	1	1	1	1	1	0	0	0	1	0	1	1	1	1	1	-	orf, hypothetical protein
b1438	1	1	1	1	1	0	0	0	0	0	0	0	0	0	1	0	0	0	0	0	0	0	0	0	1	1	1	1	1	1	1	1	0	0	0	0	0	0	0	0	0	0	0	-	orf, hypothetical protein
b1439	1	1	1	1	1	0	0	0	0	0	0	0	0	0	0	0	0	0	0	0	0	0	0	0	1	1	1	1	1	1	1	1	0	0	0	0	0	0	0	0	0	0	0	-	putative transcriptional regulator; also putative ATP-binding component of a transport system
b1440	1	1	1	1	1	0	0	0	0	0	0	0	0	0	0	0	0	0	0	0	0	0	0	0	1	1	1	1	1	1	1	1	0	0	0	0	0	0	0	0	0	0	0	b1440	putative transport protein
b1441	1	1	1	1	1	0	0	0	0	0	0	0	0	0	0	0	0	0	0	0	0	0	0	0	1	1	1	1	1	1	1	1	0	0	0	0	0	0	0	0	0	0	0	b1441	putative ATP-binding component of a transport system
b1442	1	1	1	1	1	0	0	0	0	0	0	0	0	0	0	0	0	0	0	0	0	0	0	0	1	1	1	1	1	1	1	1	0	0	0	0	0	0	0	0	0	0	0	b1442	putative transport system permease protein
b1443	1	1	1	1	1	0	0	0	0	0	0	0	0	0	0	0	0	0	0	0	0	0	0	0	1	1	1	1	1	1	0	1	1	1	1	1	1	0	1	1	1	1	1	b1443	putative transport system permease protein
b1444	1	1	1	1	1	1	1	1	1	1	1	1	0	0	1	1	0	0	0	1	1	1	1	1	1	1	1	1	1	1	1	1	1	1	1	1	1	0	1	1	1	1	1	b1444	putative aldehyde dehydrogenase
b1445	1	1	1	1	1	0	0	1	0	1	1	1	0	1	1	1	1	1	1	1	1	0	1	1	1	1	1	1	1	1	1	1	0	1	1	1	1	1	1	1	1	1	1	b1445	orf, hypothetical protein
b1446	1	1	1	1	1	0	0	0	0	1	1	1	0	1	1	1	1	1	1	1	1	0	1	1	1	1	1	1	1	1	1	1	0	1	1	1	1	0	1	1	1	1	1	b1446	orf, hypothetical protein
b1447	1	1	1	1	1	0	1	1	1	1	1	1	0	1	1	1	1	1	1	1	1	1	1	1	1	1	1	1	1	1	1	1	1	1	1	1	1	0	1	1	1	1	1	b1447	orf, hypothetical protein
b1448	1	1	1	1	1	0	1	1	1	1	1	1	1	1	1	1	1	1	1	1	1	1	1	1	1	1	1	1	1	1	1	1	1	1	1	1	1	0	1	1	1	1	1	b1448	putative resistance protein
b1449	1	1	1	1	1	0	1	1	1	1	1	1	1	1	1	1	1	1	1	1	1	1	1	1	1	1	1	1	0	0	1	1	1	1	1	1	1	0	1	1	1	1	1	yncB	putative oxidoreductase
b1450	1	0	1	1	1	0	1	1	1	1	1	1	0	0	0	0	1	1	1	1	1	1	1	1	1	1	1	1	1	1	1	1	1	1	1	1	1	0	1	1	1	1	1	b1450	orf, hypothetical protein
b1451	1	0	1	1	1	0	1	1	1	1	1	1	1	1	1	1	1	0	0	0	0	0	0	0	1	1	1	1	1	1	1	1	0	0	0	0	0	0	0	0	0	0	0	b1451	putative outer membrane receptor for iron transport
b1452	1	1	1	1	1	0	1	1	1	1	1	1	1	1	1	1	1	1	1	1	1	1	1	1	1	1	1	1	1	1	1	1	0	0	0	0	0	0	0	0	0	0	0	b1452	putative receptor
b1453	1	1	1	1	1	0	1	1	1	1	1	1	1	1	1	1	1	1	1	1	1	1	1	1	1	1	1	1	1	1	1	1	0	0	0	0	0	0	0	0	0	0	0	ansP	L-asparagine permease
b1455	1	0	1	0	0	0	0	0	0	0	0	0	1	0	0	0	1	0	0	0	0	0	0	0	1	1	1	1	1	1	0	0	0	0	0	0	0	0	0	0	0	0	0	b1455	orf, hypothetical protein
b1456	1	1	1	1	1	0	1	0	0	1	1	1	1	1	1	1	1	1	1	1	1	0	1	1	1	1	1	1	1	0	1	1	0	0	0	0	0	0	0	0	0	1	0	rhsE	rhsE protein in rhs element
b1457	0	0	0	0	0	0	0	0	0	0	0	0	0	0	0	0	0	0	0	0	0	0	0	0	1	1	1	1	0	0	0	0	0	0	0	0	0	0	0	0	0	0	0	ydcD	orf, hypothetical protein
b1458	0	0	0	0	0	0	0	0	0	0	0	0	0	0	0	0	0	0	0	0	1	0	0	0	1	1	1	1	0	0	0	0	0	0	0	0	0	0	0	0	0	0	0	b1458	orf, hypothetical protein
b1459	0	0	0	0	0	0	0	0	0	0	0	0	0	0	0	0	0	0	0	0	1	0	0	0	1	1	1	1	0	0	0	0	0	0	0	0	0	0	0	0	0	0	0	b1459	orf, hypothetical protein
b1460	1	1	1	1	1	0	1	0	0	0	0	0	0	0	0	0	0	0	0	0	1	0	0	0	1	1	1	1	1	1	1	1	0	0	0	0	0	0	0	0	0	0	0	ydcC	putative receptor
b1461	0	1	1	1	1	0	0	1	0	1	1	1	0	0	0	0	1	0	0	0	1	0	0	1	1	1	1	1	1	0	1	1	1	0	0	0	0	0	0	0	0	1	0	ydcE	orf, hypothetical protein
b1462	0	0	1	0	1	0	0	0	0	0	0	0	0	0	0	0	1	0	0	0	0	0	0	0	0	1	0	1	1	0	0	0	0	0	0	0	0	0	0	0	0	0	0	b1462	orf, hypothetical protein
b1463	1	1	1	0	1	1	0	0	0	0	0	1	1	0	0	0	1	0	0	0	1	0	0	0	1	1	1	1	1	0	1	1	0	0	0	0	0	0	0	0	0	0	0	b1463	putative N-hydroxyarylamine O-acetyltransferase
b1464	1	0	1	0	1	0	0	0	0	0	0	0	0	0	0	0	1	0	0	0	0	0	0	0	0	1	0	1	1	0	0	0	0	0	0	0	0	0	0	0	0	0	0	yddE	orf, hypothetical protein
b1465	1	1	1	0	1	0	0	0	0	0	0	0	0	0	0	0	1	0	0	0	0	0	0	0	0	1	0	1	1	0	0	0	0	0	0	0	0	0	0	0	0	0	0	narV	cryptic nitrate reductase 2, gamma subunit
b1466	1	0	1	0	1	0	0	0	0	0	0	0	0	0	0	0	1	0	0	0	0	0	0	0	0	1	0	1	1	0	0	0	0	0	0	0	0	0	0	0	0	0	0	narW	cryptic nitrate reductase 2, delta subunit, assembly function
b1467	1	0	1	0	1	0	0	0	0	0	0	0	0	0	0	0	1	0	0	0	0	0	0	0	0	1	0	1	1	0	0	0	0	0	0	0	0	0	0	0	0	0	0	narY	cryptic nitrate reductase 2, beta subunit
b1468	1	1	1	0	1	1	0	1	1	0	1	1	0	1	0	1	1	0	0	0	0	0	0	0	0	1	1	1	1	0	1	0	1	1	1	1	1	1	1	1	1	1	1	narZ	cryptic nitrate reductase 2, alpha subunit
b1469	1	1	1	0	0	0	0	0	0	0	0	0	0	0	0	0	1	0	0	0	0	0	0	0	0	1	1	1	1	0	0	0	1	1	1	1	1	1	1	1	1	1	1	narU	nitrite extrusion protein 2
b1470	1	1	1	0	1	0	0	0	0	0	0	0	0	0	0	0	0	0	0	0	1	1	1	0	1	1	1	1	1	0	1	1	1	1	1	1	1	1	1	1	1	1	1	b1470	orf, hypothetical protein
b1471	0	0	1	0	1	0	0	0	0	1	0	0	1	0	0	0	0	1	1	1	1	1	1	1	1	1	1	1	1	1	0	1	1	1	1	1	1	1	1	1	1	1	1	b1471	putative glycoportein
b1472	1	1	1	1	1	0	0	0	0	0	0	0	1	1	1	1	0	1	1	1	1	1	1	1	1	1	1	1	1	1	1	1	1	1	1	1	1	1	1	1	1	1	1	b1472	putative outer membrane porin protein
b1473	1	1	1	1	1	0	1	1	1	1	1	1	1	1	1	1	0	1	1	1	1	1	1	1	1	1	1	1	1	1	1	1	1	1	1	1	1	1	1	1	1	1	1	yddG	orf, hypothetical protein
b1474	1	1	1	1	1	0	1	1	1	1	1	1	1	1	1	1	1	1	1	1	1	1	1	1	0	1	1	1	1	1	1	1	1	1	1	1	1	1	1	1	1	1	1	fdnG	formate dehydrogenase-N, nitrate-inducible, alpha subunit
b1475	1	1	1	1	1	0	1	1	1	1	1	1	1	1	1	1	1	1	1	1	1	1	1	1	0	1	1	1	1	1	1	1	1	1	1	1	1	1	1	1	1	1	1	fdnH	formate dehydrogenase-N, nitrate-inducible, iron-sulfur beta subunit
b1476	1	1	1	1	1	0	1	1	1	1	1	1	1	1	1	0	1	1	1	1	1	1	1	1	0	1	1	1	1	1	1	1	1	1	1	1	1	1	1	1	1	1	1	fdnI	formate dehydrogenase-N, nitrate-inducible, cytochrome B556(Fdn) gamma subunit
b1477	1	1	1	0	1	0	0	1	1	1	1	1	0	1	1	1	0	1	1	1	1	1	1	1	0	1	1	1	1	0	1	1	1	1	1	1	1	1	1	1	1	1	1	yddM	orf, hypothetical protein
b1478	1	1	1	1	1	0	1	1	1	1	1	1	1	1	1	1	0	1	1	1	1	1	1	1	0	1	1	1	1	1	1	1	1	1	1	1	1	1	1	1	1	1	1	adhP	alcohol dehydrogenase
b1479	1	1	1	1	1	0	1	1	1	1	1	1	1	1	1	1	0	1	1	1	1	1	1	1	0	1	1	1	1	1	1	1	1	1	1	1	1	1	1	1	1	1	1	sfcA	NAD-linked malate dehydrogenase (malic enzyme)
b1481	0	0	1	1	1	0	1	1	1	1	1	1	1	1	1	1	0	1	1	1	1	1	1	1	0	1	1	1	1	1	1	1	0	0	0	0	0	0	0	0	0	1	1	b1481	orf, hypothetical protein
b1482	1	1	1	1	1	0	1	1	1	1	1	1	1	1	1	1	0	1	1	1	1	1	1	1	0	1	1	1	1	1	1	1	1	1	1	1	1	1	1	1	1	1	1	osmC	osmotically inducible protein
b1483	1	0	1	1	1	0	1	1	1	1	1	1	1	1	1	1	0	1	1	1	1	1	1	1	0	1	1	1	1	1	1	1	1	1	1	1	1	1	1	1	1	1	1	b1483	putative ATP-binding component of a transport system
b1484	1	1	1	1	1	0	1	1	1	1	1	1	1	1	1	1	1	1	1	1	1	1	1	1	0	1	1	1	0	1	1	1	1	1	1	1	1	1	1	1	1	1	1	b1484	putative ATP-binding component of a transport system
b1485	0	0	1	1	1	0	1	1	1	1	1	1	1	1	1	1	0	1	1	1	1	1	1	1	0	1	1	1	1	1	1	0	1	1	1	1	1	1	1	1	1	1	1	b1485	putative transport protein
b1486	0	0	1	1	1	0	0	0	0	1	1	0	1	1	1	1	0	1	1	1	1	1	1	0	0	1	1	1	1	1	1	1	0	0	0	0	0	0	0	0	0	0	0	b1486	putative transport system permease protein
b1487	0	0	1	1	1	0	1	1	1	1	1	1	1	1	1	1	0	1	1	1	1	1	1	1	0	1	1	1	1	1	1	1	0	0	0	0	0	0	0	0	0	0	0	b1487	putative hemin-binding lipoprotein
b1488	0	0	1	1	1	0	1	1	1	1	1	1	1	1	1	1	0	1	1	1	1	1	1	1	0	1	1	1	1	1	1	1	1	0	0	1	1	1	1	1	1	1	1	b1488	orf, hypothetical protein
b1489	0	1	1	1	1	0	1	1	1	1	1	1	1	1	1	1	0	1	1	1	1	1	1	1	0	1	1	1	1	1	1	1	1	1	1	1	1	1	1	1	1	1	1	b1489	putative enzyme
b1490	0	1	1	1	1	0	1	1	1	1	1	1	1	1	1	1	1	1	1	1	1	1	1	1	0	1	1	1	1	1	1	1	1	1	1	1	1	1	1	1	1	1	1	b1490	orf, hypothetical protein
b1491	1	1	1	1	1	0	1	1	1	1	1	1	1	1	1	1	1	1	1	1	1	1	1	1	1	1	1	1	1	1	1	1	1	1	1	1	1	1	1	1	1	1	1	b1491	orf, hypothetical protein
b1492	1	1	1	1	1	0	1	1	1	1	1	1	1	1	1	1	1	1	1	1	1	1	1	1	0	1	1	1	1	1	1	1	1	1	1	1	1	1	1	1	1	1	1	xasA	acid sensitivity protein, putative transporter
b1493	1	1	1	1	1	0	1	1	1	1	1	1	1	1	1	1	1	1	1	1	1	1	1	1	0	1	1	1	1	1	1	1	1	1	1	1	1	1	1	1	1	1	1	gadB	glutamate decarboxylase isozyme
b1494	0	0	1	1	1	0	1	1	1	1	1	1	1	1	1	1	1	1	1	1	1	1	1	1	0	1	1	1	1	1	1	1	1	1	0	1	1	1	1	1	1	1	1	pqqL	putative peptidase
b1495	0	0	1	1	1	1	1	1	1	1	1	1	1	1	1	1	1	1	1	1	0	1	1	0	0	1	1	1	0	1	1	1	1	1	1	1	1	1	1	1	1	0	0	yddB	orf, hypothetical protein
b1496	0	0	1	1	0	1	1	1	1	1	1	1	1	1	1	1	1	1	1	1	0	1	1	0	0	1	1	1	1	1	1	1	1	1	1	1	1	1	1	1	1	0	0	yddA	putative ATP-binding component of a transport system
b1497	0	1	1	1	0	0	0	0	0	1	0	0	1	1	1	1	1	1	1	1	0	1	1	0	0	1	1	1	0	1	1	1	1	1	1	1	1	1	1	1	1	0	0	b1497	putative enzyme
b1498	0	1	1	1	1	0	0	0	0	0	0	0	1	1	1	1	1	1	1	1	1	1	1	1	0	1	1	1	1	1	1	1	1	1	1	1	1	1	1	1	1	1	1	b1498	putative sulfatase
b1499	1	0	1	1	1	1	1	1	1	1	1	1	1	1	1	1	1	1	1	1	1	1	1	1	1	1	1	1	1	1	1	1	1	1	1	1	1	1	1	1	1	1	1	b1499	putative ARAC-type regulatory protein
b1500	1	0	1	1	1	1	1	1	1	1	1	1	1	1	1	1	1	1	1	1	1	1	1	1	1	1	1	1	1	1	1	1	1	1	1	1	1	1	1	1	1	1	1	b1500	orf, hypothetical protein
b1501	1	0	1	1	1	1	1	1	1	1	1	1	1	1	1	1	1	1	1	1	1	1	1	1	1	1	1	1	1	1	1	1	1	1	1	1	1	1	1	1	1	1	1	b1501	putative oxidoreductase, major subunit
b1502	1	0	1	1	1	0	0	0	0	0	0	0	0	0	0	0	0	0	0	0	0	0	0	0	1	1	1	1	0	1	1	1	1	1	1	1	1	1	0	1	1	1	1	b1502	putative adhesin; similar to FimH protein
b1503	0	0	0	1	0	0	0	0	0	0	0	0	0	0	0	0	0	0	0	0	0	0	0	0	1	1	1	1	0	1	1	1	0	0	0	0	0	0	0	0	1	1	0	b1503	putative fimbrial-like protein
b1504	0	0	0	1	0	0	0	0	0	0	0	0	0	0	0	0	0	0	0	0	0	0	0	0	1	0	1	1	0	1	1	1	0	0	0	0	0	0	0	0	1	1	1	b1504	putative fimbrial-like protein
b1505	0	0	0	1	0	0	0	0	0	0	0	0	0	0	0	0	0	0	0	0	0	0	0	0	0	0	0	0	1	0	0	0	0	0	0	0	0	0	0	0	0	0	0	b1505	putative outer membrane protein
b1506	0	0	0	0	1	1	0	1	1	1	1	1	0	0	0	1	1	0	0	1	1	1	1	1	1	1	1	0	1	0	1	1	0	0	0	0	0	0	0	0	0	1	0	b1506	orf, hypothetical protein
b1507	0	1	1	1	1	0	0	0	0	0	0	0	0	0	1	0	1	0	1	1	0	1	1	0	0	1	1	1	0	1	1	1	0	0	0	1	1	1	1	1	1	0	0	hipA	persistence to inhibition of murein or DNA biosynthesis, DNA-binding regulator
b1508	1	0	1	0	1	0	0	0	0	0	0	0	0	0	0	0	0	0	0	0	0	0	1	0	0	0	0	0	0	0	0	0	0	0	0	0	0	0	0	0	0	0	0	hipB	persistence to inhibition of murein or DNA biosynthesis; regulatory protein
b1509	0	0	1	0	1	0	0	0	0	0	0	0	0	0	0	0	0	0	0	0	0	0	0	0	0	0	0	0	0	0	0	0	0	0	0	0	0	0	0	0	0	0	0	b1509	putative ATP-binding component of a transport system and adhesin protein
b1510	0	0	1	0	1	0	0	0	0	0	0	0	0	0	0	0	0	0	0	0	0	0	0	0	0	0	0	0	0	0	0	0	0	0	0	0	0	0	0	0	0	0	0	ydeK	orf, hypothetical protein
b1511	0	0	0	0	1	0	0	0	0	0	0	0	0	0	0	0	0	0	0	0	0	0	0	0	0	1	1	1	0	1	1	1	1	0	1	1	1	1	1	1	1	0	0	ydeV	putative kinase
b1512	0	0	0	0	1	0	0	0	0	0	0	0	0	0	0	0	0	0	0	0	0	0	0	0	0	0	0	0	0	1	1	1	1	1	1	1	1	1	1	1	1	0	0	ydeW	putative transcriptional regulator, sorC family
b1513	0	0	1	0	1	0	0	0	0	0	0	0	0	0	0	0	0	0	0	0	0	0	0	0	0	0	0	0	0	1	1	1	1	1	1	1	1	1	1	1	1	0	0	b1513	putative ATP-binding component of a transport system
b1514	0	0	0	0	1	0	0	0	0	1	0	1	0	0	0	0	1	0	0	1	0	0	1	0	0	0	0	0	0	1	1	1	1	1	1	1	1	1	1	1	1	1	1	ydeY	putative transport system permease protein
b1515	0	0	0	0	1	0	0	0	0	0	0	0	0	0	0	0	0	0	0	0	0	0	0	0	0	0	1	0	0	1	1	1	1	1	1	1	1	1	1	1	1	0	0	ydeZ	putative transport system permease protein
b1516	0	0	0	0	1	0	0	0	0	0	0	0	0	0	0	0	0	0	0	0	0	0	0	0	1	0	1	0	0	1	1	1	1	1	1	1	1	1	1	1	1	1	1	b1516	putative LACI-type transcriptional regulator
b1517	1	0	0	0	1	0	0	0	0	0	0	0	0	0	0	0	0	0	0	0	0	0	0	0	0	0	1	1	1	1	1	1	1	1	1	1	1	1	1	1	1	1	1	yneB	orf, hypothetical protein
b1518	1	0	0	1	1	0	0	0	0	0	0	0	0	0	0	0	0	0	0	0	0	0	1	0	0	0	1	1	1	1	1	1	1	1	1	1	1	1	1	1	1	1	1	b1518	orf, hypothetical protein
b1519	1	1	0	1	1	0	1	1	1	1	1	1	0	0	0	0	1	1	1	1	1	1	1	1	1	0	1	1	1	1	1	1	1	1	1	1	1	1	1	1	1	1	1	b1519	putative enzyme
b1520	1	1	0	1	1	0	1	1	1	1	1	1	0	0	0	0	1	1	1	1	1	1	1	1	1	0	1	1	1	1	1	1	1	1	1	1	1	1	1	1	1	1	1	b1520	orf, hypothetical protein
b1521	1	1	1	1	1	0	1	1	1	1	1	1	0	0	0	0	1	1	1	1	1	1	1	1	1	1	1	1	1	1	1	1	1	1	1	1	1	1	1	1	1	1	1	uxaB	altronate oxidoreductase
b1522	1	1	1	1	1	0	1	1	0	1	1	1	1	1	0	1	1	1	1	1	1	1	1	1	1	1	1	1	1	1	1	1	0	1	1	1	1	1	1	1	1	1	1	b1522	orf, hypothetical protein
b1523	1	1	1	1	1	0	1	1	1	1	1	1	1	1	0	1	1	1	1	1	1	1	1	1	1	1	1	1	1	1	1	1	1	1	1	1	1	1	1	1	1	1	1	b1523	orf, hypothetical protein
b1524	1	1	1	1	1	0	1	1	1	1	1	1	1	1	0	1	1	1	1	1	1	1	1	1	1	1	1	1	1	1	1	1	1	1	1	1	1	1	1	1	1	1	1	yneH	putative glutaminase
b1525	1	1	1	1	1	0	1	1	1	1	1	1	1	1	0	1	1	1	1	1	1	1	1	1	1	1	1	1	1	1	1	1	1	1	1	1	1	1	1	1	1	1	1	b1525	putative aldehyde dehydrogenase
b1526	1	1	1	1	1	0	1	1	1	1	1	1	1	1	0	1	1	1	1	1	1	1	1	1	1	1	1	1	1	1	0	1	0	0	0	0	0	0	0	0	0	0	0	yneJ	putative transcriptional regulator LYSR-type
b1527	1	1	1	1	1	0	1	1	1	1	1	1	1	1	0	1	1	1	1	1	1	1	1	1	1	1	1	1	1	1	1	1	1	1	1	1	1	1	1	1	1	1	1	b1527	orf, hypothetical protein
b1528	1	1	1	0	1	0	1	1	1	1	1	1	1	1	0	1	1	1	1	1	1	1	1	1	1	1	1	1	1	1	1	1	1	1	1	1	1	1	1	1	1	1	1	ydeA	putative resistance / regulatory protein
b1529	1	1	1	0	1	0	1	1	1	1	1	1	1	1	0	0	1	1	1	1	1	1	1	1	1	1	1	1	1	1	1	1	1	1	1	1	1	1	1	1	1	1	1	ydeB	orf, hypothetical protein
b1531	1	1	1	0	1	0	1	1	1	1	1	1	1	1	0	1	1	1	1	1	1	1	1	1	1	1	1	1	0	1	1	1	1	1	1	1	1	1	1	1	1	1	1	marA	multiple antibiotic resistance; transcriptional activator of defense systems
b1532	1	1	1	0	1	0	1	1	1	1	1	1	1	1	0	1	1	1	1	1	1	1	1	1	1	1	1	1	0	1	1	1	1	1	1	1	1	1	1	1	1	1	1	marB	multiple antibiotic resistance protein
b1533	1	0	0	1	1	0	0	0	0	0	0	0	0	0	0	0	0	0	0	0	0	0	0	0	1	0	0	1	1	1	1	0	1	1	1	1	1	1	1	1	1	1	1	ydeD	orf, hypothetical protein
b1534	1	0	0	1	1	0	0	0	0	0	0	0	0	0	0	0	0	0	0	0	0	0	0	0	0	1	1	1	1	1	1	1	1	1	1	1	1	1	1	1	1	1	1	ydeF	putative transport protein
b1535	1	0	1	1	1	0	1	1	1	1	1	1	1	1	1	0	0	0	1	1	1	1	1	1	0	1	1	1	1	1	1	1	0	0	0	0	0	0	0	0	0	0	0	ydeH	orf, hypothetical protein
b1535a	1	0	1	1	1	0	1	1	1	1	1	1	1	1	0	1	0	1	1	1	1	1	1	1	0	1	1	1	1	1	1	1	0	0	0	1	0	0	0	0	0	0	0	ydeG	Uncharacterized protein
b1536	1	0	1	1	1	0	1	1	1	1	1	1	0	0	0	0	1	0	0	0	0	0	0	0	0	1	0	0	1	1	1	1	1	1	1	1	1	1	1	1	0	0	0	ydeI	orf, hypothetical protein
b1537	1	0	1	0	1	0	1	1	1	1	1	1	1	1	1	1	1	0	0	0	0	0	0	0	0	1	0	0	1	1	1	1	1	1	1	1	1	1	1	1	0	1	1	ydeJ	orf, hypothetical protein
b1538	1	1	1	1	1	0	1	1	1	1	1	1	1	1	1	1	1	1	1	1	1	1	1	1	0	1	1	1	1	1	1	1	1	1	1	1	1	1	1	1	1	1	1	dcp	dipeptidyl carboxypeptidase II
b1539	1	1	1	1	1	0	1	1	1	1	1	1	1	1	1	1	1	1	1	1	1	1	1	1	0	1	1	1	1	1	1	1	1	1	1	1	1	1	1	1	1	1	1	ydfG	putative oxidoreductase
b1540	1	1	1	1	1	0	1	1	1	1	1	1	1	1	1	1	1	1	1	1	1	1	1	1	0	1	1	1	1	1	1	1	1	1	1	1	1	1	1	1	1	1	1	ydfH	orf, hypothetical protein
b1541	1	1	1	1	1	0	1	1	1	1	1	1	1	1	1	1	1	1	1	1	1	1	1	1	0	1	1	1	1	1	1	1	1	1	1	1	1	1	1	1	1	1	1	b1541	orf, hypothetical protein
b1542	1	1	1	1	1	0	1	1	1	1	1	1	1	1	1	1	1	1	1	1	1	1	1	1	0	1	1	1	1	1	1	1	1	1	1	1	1	1	1	1	1	1	1	ydfI	putative oxidoreductase
b1543	1	0	0	1	1	0	0	0	0	0	0	0	0	0	0	0	0	0	0	0	0	0	0	0	0	1	1	1	1	1	1	0	1	1	1	1	1	1	1	1	0	0	0	b1543	putative transport protein

b1544	0	0	0	1	0	0	0	1	1	1	1	0	0	0	1	1	1	0	0	1	0	1	0	0	0	0	1	1	1	0	1	0	0	0	0	0	0	1	1	1	1	1	0	ydfK	orf, hypothetical protein
b1545	0	0	0	0	0	0	0	0	0	0	0	0	0	0	0	0	0	0	0	0	0	0	0	0	0	0	0	0	1	0	0	0	0	0	0	0	0	0	0	0	0	0	0	b1545	orf, hypothetical protein
b1546	0	0	1	1	1	1	1	1	1	1	1	1	1	0	0	0	1	1	1	1	1	1	1	1	0	1	1	1	1	0	1	1	0	0	0	0	0	0	0	0	0	1	1	ydfM	orf, hypothetical protein
b1547	0	0	1	1	1	0	1	1	1	1	1	1	1	0	0	0	1	1	1	1	1	1	1	1	0	1	1	0	1	0	0	1	0	0	0	0	0	0	0	0	0	1	1	b1547	orf, hypothetical protein
b1548	0	0	1	0	0	0	0	0	0	0	0	0	0	0	0	0	0	0	0	0	0	0	0	0	0	0	0	1	1	0	0	0	0	0	0	0	0	0	0	0	0	0	0	nohA	homolog of Qin prophage packaging protein NU1
b1549	0	0	0	0	0	0	0	0	0	0	0	0	0	0	0	0	0	0	0	0	0	0	0	0	0	0	0	0	0	0	0	0	0	0	0	0	0	0	0	0	0	0	0	ydfO	orf, hypothetical protein
b1550	0	0	0	0	0	0	0	0	0	0	0	0	0	0	0	0	0	0	0	0	0	0	0	0	0	0	0	1	1	0	0	0	0	0	0	0	0	0	0	0	0	0	0	b1550	orf, hypothetical protein
b1551	0	0	0	0	0	0	0	0	0	0	0	0	0	0	0	0	0	0	0	0	0	0	0	0	0	0	0	1	1	0	0	0	0	0	0	0	0	0	0	0	0	0	0	b1551	orf, hypothetical protein
b1552	0	0	1	0	0	0	0	0	0	0	0	0	0	1	0	0	0	0	0	0	0	0	0	0	0	0	0	1	1	0	0	1	0	1	1	0	0	0	0	0	0	0	0	cspI	cold shock-like protein
b1553	0	0	0	0	0	0	0	0	0	0	0	0	0	0	0	0	0	0	0	0	0	0	0	0	0	1	0	1	1	0	0	1	0	0	0	0	0	0	0	0	0	0	0	b1553	orf, hypothetical protein
b1554	1	1	0	1	0	0	1	1	1	1	1	1	1	1	1	0	1	1	1	1	1	1	1	1	0	1	0	1	1	0	1	1	1	1	1	1	1	1	1	1	0	1	0	b1554	putative lysozyme
b1555	1	1	1	0	0	1	1	1	1	1	1	1	0	0	0	0	0	1	1	1	1	1	1	1	0	1	0	1	1	1	1	1	0	0	0	0	0	0	0	0	0	1	1	b1555	orf, hypothetical protein
b1556	1	1	1	1	1	0	0	0	0	1	1	1	0	0	0	0	0	0	0	0	1	1	1	1	0	1	0	1	1	0	1	1	1	1	1	0	0	1	1	0	1	1	1	b1556	orf, hypothetical protein
b1557	0	0	0	0	0	0	0	0	0	0	0	0	0	0	0	0	0	0	0	0	0	0	0	0	0	0	0	1	1	0	0	0	0	0	0	0	0	0	0	0	0	0	0	cspB	cold shock protein; may affect transcription
b1558	0	0	0	0	0	0	0	0	0	0	0	0	0	0	0	0	0	0	0	0	0	0	0	0	0	0	0	0	0	0	0	0	0	0	0	0	0	0	0	0	0	0	0	cspF	cold shock protein
b1559	0	0	0	0	0	0	0	1	1	1	1	0	0	0	0	0	0	1	1	1	1	1	1	1	0	0	0	1	0	0	0	0	0	0	0	0	0	0	0	0	0	1	1	b1559	orf, hypothetical protein
b1560	1	1	1	1	0	0	1	1	1	1	1	1	1	1	0	1	0	1	1	1	1	1	1	1	0	1	1	1	1	0	1	1	0	1	1	1	1	1	1	1	1	0	0	b1560	orf, hypothetical protein
b1561	1	0	0	1	1	1	0	1	0	0	1	1	1	0	0	1	0	1	1	1	1	1	1	1	0	1	1	1	1	0	1	1	1	1	1	1	1	1	1	1	1	1	1	rem	orf, hypothetical protein
b1562	1	1	1	1	1	1	1	1	1	1	1	1	1	1	1	1	1	1	1	1	1	1	1	1	0	1	1	1	1	1	1	1	1	1	1	1	1	1	1	1	1	1	1	relF	polypeptide destructive to membrane potential
b1563	0	0	0	0	0	0	0	0	0	0	0	0	0	0	0	0	0	0	0	0	0	0	0	0	0	1	1	1	1	0	0	0	1	1	1	1	1	1	1	1	1	1	1	relE	orf, hypothetical protein
b1564	0	0	0	0	0	0	0	0	0	0	0	0	0	0	0	0	0	0	0	0	0	0	0	0	0	1	1	1	1	0	0	0	1	1	1	1	1	1	1	1	1	1	1	relB	negative regulator of translation
b1565	0	0	0	1	0	0	0	1	0	0	0	0	0	0	0	0	0	0	0	0	1	0	0	0	0	1	1	1	1	0	0	0	1	1	1	1	1	1	1	1	1	1	1	b1565	orf, hypothetical protein
b1566	0	0	0	0	0	0	0	0	0	0	0	0	0	0	0	0	0	0	0	0	0	0	0	0	0	0	0	0	0	0	0	0	0	0	0	0	0	0	0	0	0	0	0	flxA	orf, hypothetical protein
b1568	0	0	0	0	0	0	0	0	0	0	0	0	1	0	0	0	0	0	1	1	0	1	1	1	0	0	0	0	0	0	0	0	0	0	0	0	0	0	0	0	0	0	0	b1568	orf, hypothetical protein
b1569	0	0	1	0	0	0	0	0	0	0	0	0	1	0	0	0	0	1	1	1	1	1	1	1	0	0	0	0	0	0	1	1	0	1	1	0	0	0	0	0	0	0	0	dicC	regulator of dicB
b1570	0	0	0	0	0	0	0	0	0	0	0	0	0	0	0	0	0	0	0	0	0	0	0	0	0	0	0	0	0	0	0	0	0	0	0	0	0	0	0	0	0	0	0	dicA	regulator of dicB
b1571	0	1	1	1	0	1	1	1	1	0	1	0	1	0	1	0	1	1	1	1	1	1	1	1	0	1	1	0	1	1	1	1	1	1	1	1	1	1	1	1	0	1	1	ydfA	orf, hypothetical protein
b1572	0	1	0	1	0	0	0	0	0	0	0	0	1	0	0	0	1	0	0	1	0	1	1	1	0	0	1	0	1	0	1	0	0	0	0	0	0	0	0	0	0	0	0	ydfB	orf, hypothetical protein
b1573	0	1	0	1	0	0	0	0	0	0	0	0	1	0	0	0	1	0	0	1	1	1	1	1	0	0	1	0	1	0	1	0	0	0	0	0	0	0	0	0	0	0	0	ydfC	orf, hypothetical protein
b1575	0	1	0	1	0	0	0	0	0	0	0	0	1	1	1	1	1	0	0	1	0	1	1	1	0	1	1	1	1	0	1	0	0	0	0	0	0	0	0	0	0	0	0	dicB	inhibition of cell division
b1576	0	1	0	1	0	0	0	0	0	0	0	0	1	0	0	0	1	1	1	1	1	1	1	1	0	1	1	0	1	0	1	0	0	0	0	0	0	0	0	0	0	0	0	ydfD	orf, hypothetical protein
b1577	0	1	0	0	0	0	0	0	0	0	0	0	1	0	0	0	1	1	1	1	0	1	1	1	0	0	0	0	0	0	0	0	0	0	0	0	0	0	0	0	0	0	0	ydfE	orf, hypothetical protein
b1578	1	0	1	1	0	1	1	1	1	1	1	1	0	1	1	1	1	1	1	1	1	1	1	1	1	1	1	1	1	1	0	1	1	1	1	1	1	1	1	1	1	1	1	b1578	orf, hypothetical protein
b1579	0	0	0	0	0	0	0	0	0	0	0	0	0	0	0	0	0	1	0	0	1	0	0	0	0	0	0	0	1	0	0	1	0	0	0	0	0	0	0	0	0	0	0	b1579	putative transposase
b1580	1	0	0	0	1	0	0	0	0	0	0	0	0	0	0	0	0	0	0	0	0	0	0	0	1	1	1	1	1	1	1	0	0	0	0	0	0	0	0	0	0	0	0	rspB	starvation sensing protein
b1581	1	1	0	0	1	0	0	0	0	0	0	0	0	0	0	0	0	0	0	0	0	0	0	0	1	1	1	1	1	1	1	0	1	1	1	1	1	1	1	1	1	1	1	rspA	starvation sensing protein
b1582	1	1	0	0	1	0	0	1	0	0	0	1	0	0	0	0	0	0	0	0	0	0	0	0	1	1	1	1	1	1	1	1	1	1	1	1	1	1	1	1	0	1	1	b1582	orf, hypothetical protein
b1583	1	1	0	1	1	0	0	0	0	0	0	0	0	0	0	0	0	0	0	0	0	0	0	0	1	1	1	1	1	1	1	1	1	1	1	1	1	1	1	1	1	1	1	b1583	orf, hypothetical protein
b1584	1	1	0	1	1	1	1	1	1	1	1	1	0	1	1	0	0	1	1	1	1	0	1	1	1	1	1	1	1	1	1	1	1	1	1	1	1	1	1	1	0	1	1	speG	spermidine N1-acetyltransferase
b1585	1	1	0	0	1	1	1	1	1	1	1	1	1	1	1	0	0	1	1	1	1	1	1	1	1	1	1	1	1	1	1	1	1	1	1	1	1	1	1	1	1	1	1	ynfC	orf, hypothetical protein
b1586	1	1	0	1	1	1	1	1	1	1	1	1	1	1	1	1	0	1	1	1	1	1	1	1	1	1	1	1	1	1	1	1	1	1	1	1	1	1	0	1	1	1	1	b1586	orf, hypothetical protein
b1587	1	1	0	1	1	0	1	1	1	0	1	1	1	1	1	1	0	0	0	1	1	1	1	1	1	1	1	1	1	1	1	1	1	1	1	1	1	1	0	1	1	1	1	b1587	putative oxidoreductase, major subunit
b1588	1	1	0	0	1	1	1	1	1	1	1	1	0	1	1	1	0	1	1	1	1	1	0	0	1	1	1	1	0	1	1	1	1	1	1	1	1	1	1	1	0	0	0	b1588	putative oxidoreductase, major subunit
b1589	1	1	1	1	1	1	1	1	1	1	1	1	1	1	1	1	0	1	1	1	1	1	0	0	1	1	1	1	0	1	1	1	1	1	1	1	1	1	1	1	1	0	0	b1589	putative oxidoreductase, Fe-S subunit
b1590	1	0	1	1	1	1	1	1	1	1	1	1	0	1	1	1	0	1	1	1	1	1	0	0	1	1	1	1	1	1	1	1	1	1	1	1	1	1	1	1	1	1	1	b1590	putative DMSO reductase anchor subunit
b1591	1	1	1	1	1	1	1	1	1	1	1	1	0	1	1	1	0	1	1	1	1	1	1	1	1	1	1	1	1	1	1	1	1	1	1	1	1	1	1	1	1	1	1	b1591	putative oxidoreductase component
b1592	1	1	1	1	1	1	1	1	1	1	1	1	0	1	1	1	0	1	1	1	1	1	1	1	1	1	1	1	1	1	1	1	1	1	1	1	1	1	1	1	1	1	1	b1592	putative chloride channel
b1593	1	1	1	1	1	1	1	1	1	1	1	1	1	1	1	1	0	1	1	1	1	1	1	1	1	1	1	1	1	1	1	1	1	1	1	1	1	1	1	1	1	1	1	b1593	orf, hypothetical protein
b1596	1	1	1	1	1	0	1	1	1	1	1	1	1	1	1	1	1	1	1	1	1	1	1	1	1	1	1	1	1	1	1	1	1	1	1	1	1	1	1	1	1	1	1	ynfM	putative transport protein
b1603	1	1	1	1	1	0	1	1	1	1	1	1	1	1	1	1	1	1	1	1	1	1	1	1	1	1	1	1	1	1	1	1	1	1	1	1	1	1	1	1	1	1	1	pntA	pyridine nucleotide transhydrogenase, alpha subunit
b1607	1	1	1	1	1	1	1	1	1	1	1	1	0	1	1	1	0	1	1	1	1	1	1	1	1	1	1	1	1	1	1	1	1	1	1	1	1	1	1	1	1	1	1	ydgC	orf, hypothetical protein
b1611	1	1	1	1	1	0	0	1	1	1	1	1	0	1	1	1	1	1	1	1	1	1	1	1	1	1	1	1	1	1	1	1	1	1	1	1	1	1	1	1	1	1	1	fumC	fumarase C= fumarate hydratase Class II; isozyme
b1612	1	1	1	1	1	0	1	0	1	1	1	1	1	1	1	1	0	1	1	1	1	1	1	1	1	1	1	1	1	1	1	1	1	1	1	1	1	1	1	1	1	1	1	fumA	fumarase A = fumarate hydratase Class I; aerobic isozyme
b1614	1	1	1	1	1	0	0	1	1	1	1	1	1	1	1	0	1	1	1	1	1	1	1	1	1	1	1	1	1	1	1	1	1	1	1	1	1	1	1	1	1	1	1	ydgA	orf, hypothetical protein
b1615	1	1	1	1	0	1	0	1	1	1	0	0	0	1	0	0	1	0	0	0	1	1	1	1	0	1	1	1	1	0	0	1	1	1	1	0	0	0	0	0	0	0	0	uidC	membrane-associated protein
b1616	1	0	1	1	1	1	1	1	1	1	1	1	1	1	1	1	1	1	1	1	1	1	1	1	1	1	1	1	1	1	1	1	1	1	1	1	1	1	1	1	1	1	1	uidB	glucuronide permease
b1617	1	1	1	1	0	0	0	0	0	0	0	0	0	1	0	0	0	0	0	0	0	0	0	0	0	0	1	0	1	1	1	1	1	1	1	1	1	1	1	1	1	0	0	uidA	beta-D-glucuronidase
b1618	1	0	1	1	1	1	1	1	1	1	1	1	1	1	1	1	1	1	1	1	1	1	1	1	1	1	1	1	1	1	1	0	1	1	1	1	1	1	1	1	1	1	1	uidR	repressor for uid operon
b1619	1	1	1	1	1	1	1	1	1	1	1	1	1	1	1	1	1	1	1	1	1	1	1	1	1	1	1	1	1	1	1	0	1	1	1	1	1	1	1	1	1	1	1	hdhA	NAD-dependent 7alpha-hydroxysteroid dehydrogenase, dehydroxylation of bile acids
b1620	1	1	1	1	1	1	1	1	1	1	1	1	1	1	1	1	1	1	1	1	1	1	1	1	1	1	1	1	1	1	1	0	1	1	1	1	1	1	1	1	1	1	1	malI	repressor of malX and Y genes
b1629	1	1	1	1	1	0	0	0	1	1	1	1	1	1	1	0	0	1	1	1	1	1	1	1	0	1	1	1	1	1	1	1	0	1	1	1	1	1	1	1	1	1	1	b1629	putative membrane protein
b1633	1	1	1	1	1	1	1	1	1	1	1	1	1	1	1	1	1	1	1	1	1	1	1	1	1	1	1	1	1	1	1	1	1	1	1	1	0	1	1	1	1	1	1	nth	endonuclease III; specific for apurinic and/or apyrimidinic sites
b1642	1	1	1	1	1	0	1	1	1	1	1	1	1	1	1	1	1	1	1	1	1	1	1	1	1	1	1	1	1	1	1	1	1	1	1	1	1	1	1	1	1	1	1	slyA	transcriptional regulator for cryptic hemolysin
b1643	1	1	1	1	1	0	1	1	1	1	1	1	1	1	1	1	1	1	1	1	1	1	1	1	1	1	1	1	1	1	1	1	1	1	1	1	1	1	1	1	1	1	1	b1643	orf, hypothetical protein
b1644	1	1	1	1	1	0	1	1	1	1	1	1	1	1	1	1	1	1	1	1	1	1	1	1	1	1	1	1	1	1	1	1	1	1	1	1	1	1	1	1	1	1	1	b1644	putative membrane protein
b1646	1	0	1	1	1	1	1	1	1	1	1	1	1	1	1	1	1	1	1	1	1	1	1	1	1	1	1	1	1	1	1	1	1	1	1	1	1	1	1	1	1	1	1	sodC	superoxide dismutase precursor (Cu-Zn)
b1647	1	1	1	1	1	1	1	1	1	1	1	1	1	1	1	1	1	1	1	1	1	1	1	1	0	1	1	1	1	1	1	1	1	1	1	1	1	1	1	1	0	1	1	b1647	orf, hypothetical protein
b1648	1	1	1	1	1	1	1	1	1	1	1	1	1	1	1	1	1	1	1	1	1	1	1	1	1	1	1	1	0	1	1	1	1	1	1	1	1	1	1	1	1	1	1	b1648	orf, hypothetical protein
b1649	1	1	1	1	1	0	1	1	1	1	1	1	1	1	1	1	1	1	1	1	1	1	1	1	1	1	1	1	1	1	1	1	1	1	1	1	1	1	1	1	0	1	1	b1649	orf, hypothetical protein
b1657	1	0	1	1	1	1	1	1	1	1	1	1	1	1	1	1	1	1	1	1	1	1	1	1	1	1	1	1	1	1	1	1	1	1	1	1	1	1	1	1	1	1	1	b1657	putative transport protein
b1658	1	1	1	1	1	0	1	1	1	1	1	1	1	1	1	1	1	1	1	1	1	1	1	1	1	1	1	1	1	1	1	1	1	1	1	1	1	1	1	1	1	1	1	purR	transcriptional repressor for pur regulon, glyA, glnB, prsA, speA
b1667	0	1	1	1	1	0	1	1	1	1	1	1	1	1	1	1	1	1	1	1	1	1	1	1	1	1	1	1	1	1	1	1	1	1	1	1	1	1	1	1	1	0	0	b1667	orf, hypothetical protein
b1668	1	0	1	1	1	1	1	1	1	1	1	1	1	1	1	1	0	1	1	1	1	1	1	0	1	1	1	1	1	1	1	1	1	1	1	1	1	1	1	1	1	0	0	b1668	orf, hypothetical protein
b1669	1	1	1	1	1	1	1	1	1	1	1	1	1	1	1	1	0	1	1	1	1	1	1	0	1	1	1	1	1	1	1	1	1	1	1	1	1	1	1	1	1	0	0	b1669	orf, hypothetical protein
b1671	1	1	1	1	0	1	1	1	1	1	1	1	1	1	1	1	0	1	1	1	1	1	1	0	1	1	1	1	1	1	1	1	1	1	1	1	1	1	1	1	1	0	0	b1671	putative oxidoreductase, Fe-S subunit
b1672	1	1	1	1	0	1	1	1	1	1	1	1	1	1	1	1	0	1	1	1	1	1	1	0	1	1	1	1	1	1	1	1	1	1	1	1	1	1	1	1	1	0	0	b1672	orf, hypothetical protein
b1673	0	1	1	1	1	1	1	1	1	1	1	1	1	1	1	1	1	1	1	1	1	1	1	0	1	1	1	1	1	1	1	1	1	1	1	1	1	1	1	1	1	1	1	b1673	orf, hypothetical protein
b1674	0	1	1	1	1	0	0	0	0	0	0	0	1	1	1	1	1	0	0	1	0	1	0	1	1	1	1	1	1	1	1	1	1	1	1	1	1	1	1	1	0	1	1	b1674	putative oxidoreductase, Fe-S subunit
b1675	1	1	1	1	1	1	1	1	1	1	1	1	1	1	1	1	1	1	1	1	1	1	1	1	1	1	1	1	1	1	1	1	1	1	1	1	1	1	1	1	0	1	1	b1675	orf, hypothetical protein
b1680	1	1	1	1	1	0	1	1	1	1	1	1	1	1	1	1	1	1	1	1	1	1	1	1	1	1	1	1	1	1	1	1	1	1	1	1	1	1	1	0	1	1	1	b1680	orf, hypothetical protein
b1683	1	1	1	1	1	1	1	1	1	1	1	1	1	1	1	1	0	1	1	1	1	1	1	1	1	1	1	1	1	1	1	1	1	1	1	1	1	1	1	1	1	1	1	ynhE	orf, hypothetical protein
b1684	0	1	1	1	1	1	1	1	1	1	1	1	1	1	1	1	1	1	1	1	1	1	1	1	1	1	1	1	1	1	1	1	1	1	1	1	1	1	1	1	1	1	1	ydiC	orf, hypothetical protein
b1689	1	1	1	1	1	1	1	1	1	1	1	1	1	1	1	1	1	1	1	1	1	1	1	0	1	1	1	1	1	1	1	1	1	1	1	1	1	1	1	1	1	1	1	b1689	orf, hypothetical protein
b1690	1	1	1	1	1	0	0	0	0	0	0	0	0	0	0	0	0	0	0	0	0	0	0	0	1	1	1	1	1	1	1	1	1	1	1	1	1	1	1	1	1	1	1	b1690	putative transport system permease protein
b1691	0	1	1	1	1	0	1	1	1	1	1	1	1	1	1	1	0	1	1	1	1	0	1	0	1	1	1	1	1	1	1	1	1	1	1	1	1	1	1	1	1	1	1	b1691	putative amino acid/amine transport protein
b1692	1	1	1	1	1	0	1	1	1	1	1	1	0	1	1	1	1	1	1	1	1	1	1	1	1	1	1	1	1	1	1	1	1	1	1	1	1	1	1	1	1	1	1	ydiB	putative oxidoreductase
b1694	0	1	0	1	0	1	1	1	1	1	1	1	1	1	1	1	0	1	1	1	1	1	1	1	1	1	1	1	1	0	0	0	0	0	0	0	0	0	0	0	0	1	1	ydiF	putative enzyme
b1695	1	1	0	1	1	1	1	1	1	1	1	1	1	1	1	1	0	1	1	1	1	1	1	0	1	1	1	1	1	0	0	0	0	0	0	0	0	0	0	0	0	1	1	b1695	putative oxidoreductase
b1696	1	1	0	1	1	1	1	1	1	1	1	1	1	1	1	1	0	1	1	1	1	1	1	0	1	0	1	1	1	0	0	0	1	1	1	1	1	1	1	1	0	1	1	b1696	putative ARAC-type regulatory protein
b1697	1	1	0	1	1	1	1	1	1	1	1	1	1	1	1	1	1	1	1	1	1	1	1	1	1	1	1	1	1	1	1	1	1	1	1	1	1	1	1	1	1	1	1	ydiQ	putative transport protein
b1698	1	1	1	1	1	1	1	1	1	1	1	1	1	1	1	1	1	1	1	1	1	1	1	1	1	0	1	1	1	0	1	1	1	1	1	1	1	1	1	1	1	1	1	ydiR	putative flavoprotein
b1699	1	1	0	1	1	0	1	1	1	1	1	1	1	1	1	1	1	1	1	1	1	1	1	1	1	0	1	1	0	0	0	0	1	1	1	1	1	1	1	1	1	1	1	ydiS	flavoprotein; probably electron transport
b1700	1	1	0	1	1	1	1	1	1	1	1	1	1	1	1	1	1	1	1	1	1	1	1	1	1	0	1	1	1	1	1	1	1	1	1	1	1	1	1	1	1	1	1	ydiT	orf, hypothetical protein
b1701	1	1	0	1	1	1	1	1	1	1	1	1	1	1	1	1	1	1	1	1	1	1	1	1	1	1	1	1	1	1	1	1	1	1	1	1	1	1	1	1	1	1	1	ydiD	putative ligase/synthetase
b1705	1	1	1	1	1	1	1	1	1	1	1	1	1	1	1	1	1	1	1	1	1	0	1	1	0	0	0	1	0	0	1	1	0	1	1	1	1	1	1	1	1	1	1	ydiE	orf, hypothetical protein
b1707	1	0	1	1	1	1	1	1	1	1	1	1	1	1	1	1	1	1	1	1	1	1	1	1	1	1	1	1	0	1	1	1	1	1	1	1	1	1	1	1	1	1	1	b1707	orf, hypothetical protein
b1721m	0	0	1	1	1	1	1	1	1	1	1	1	1	1	1	1	1	0	0	0	0	0	1	0	1	1	1	1	1	1	1	1	0	1	1	1	1	1	1	1	0	0	0	-	Ankyrin repeat protein
b1722	0	1	1	1	1	1	1	1	1	1	1	1	1	1	1	1	1	1	1	1	1	1	1	0	1	1	1	1	1	1	1	1	1	1	1	1	1	1	1	1	1	1	1	b1722	orf, hypothetical protein
b1725	1	0	1	1	1	1	1	1	1	1	1	1	1	1	1	1	1	1	1	1	1	1	1	1	1	1	1	1	1	1	1	1	1	1	1	1	0	1	1	1	1	1	1	b1725	orf, hypothetical protein
b1730	1	0	1	1	1	0	0	0	0	0	0	0	0	0	0	0	0	0	0	0	0	0	0	0	1	1	1	1	1	1	1	1	1	1	1	1	1	1	1	1	1	1	1	b1730	orf, hypothetical protein
b1731	0	1	1	1	1	0	0	0	0	0	0	0	0	0	0	0	0	0	0	0	0	0	0	0	1	1	1	1	1	1	1	1	1	1	1	1	1	1	1	1	1	1	1	b1731	orf, hypothetical protein
b1732	0	0	1	1	1	0	0	0	0	0	0	0	0	0	0	0	0	0	0	0	0	0	0	0	1	1	1	1	1	1	1	1	1	1	1	1	1	1	1	1	1	1	1	katE	catalase; hydroperoxidase HPII(III)
b1733	0	1	1	1	1	0	0	0	0	0	0	0	0	0	0	0	0	0	0	0	0	0	0	0	0	1	1	1	1	1	1	1	0	1	1	1	1	1	1	1	1	1	1	ydjC	orf, hypothetical protein
b1734	0	0	1	1	1	0	0	0	0	0	0	0	0	0	0	0	0	0	0	0	0	0	0	0	1	1	1	1	1	1	1	1	1	1	1	1	1	1	1	1	1	1	1	celF	phospho-beta-glucosidase; cryptic
b1735	0	0	1	1	1	0	0	0	0	0	0	0	0	0	0	0	0	0	0	0	1	1	1	0	1	1	1	1	1	1	1	1	1	1	1	1	1	1	1	1	1	0	0	celD	negative transcriptional regulator of cel operon
b1736	0	1	1	1	1	1	1	1	1	1	1	1	1	1	1	1	1	1	1	1	1	1	1	1	1	1	1	1	1	1	1	1	1	1	1	1	1	1	1	1	1	1	1	celC	PEP-dependent phosphotransferase enzyme III for cellobiose, arbutin, and salicin

b1737	0	0	1	1	1	1	1	1	1	1	1	1	1	1	1	1	1	1	1	1	1	1	1	1	1	1	1	1	1	1	1	1	1	1	1	1	1	1	1	1	1	1	1	celB	PEP-dependent phosphotransferase enzyme II for cellobiose, arbutin, and salicin
b1738	0	1	1	1	1	1	1	1	1	1	1	1	1	1	1	1	1	1	1	1	1	1	1	1	1	1	1	1	1	1	1	1	1	1	1	1	1	1	1	1	1	1	1	celA	PEP-dependent phosphotransferase enzyme IV for cellobiose, arbutin, and salicin
b1741	1	0	1	1	1	1	1	1	1	1	1	1	1	1	1	1	1	1	1	1	1	1	1	1	1	1	1	1	1	1	1	1	1	1	1	1	1	1	1	1	1	1	1	b1741	putative excinuclease subunit
b1742	1	1	1	1	1	1	1	1	1	1	1	1	1	1	1	1	1	1	1	1	1	1	1	1	0	1	1	1	1	1	1	1	1	1	1	1	1	1	1	1	1	1	1	b1742	orf, hypothetical protein
b1745	1	1	1	1	1	0	1	1	1	1	1	1	1	1	1	1	1	1	1	1	1	1	1	1	1	1	1	1	1	1	1	1	1	1	1	1	1	1	1	1	1	1	1	b1745	orf, hypothetical protein
b1746	1	1	1	1	1	1	1	1	1	1	1	1	1	1	1	1	1	1	1	1	1	1	1	1	1	1	1	1	1	1	1	1	1	1	1	1	1	1	1	1	0	1	1	b1746	putative aldehyde dehydrogenase
b1749	1	1	1	1	1	1	1	1	1	1	1	1	1	1	1	1	1	1	1	1	1	1	1	1	0	0	0	1	0	0	0	1	1	1	1	1	1	1	1	1	1	1	1	xthA	exonuclease III
b1756	1	1	1	1	1	0	1	1	1	1	1	1	1	1	1	0	0	1	1	1	1	0	1	1	1	1	1	1	1	1	1	1	1	1	1	1	1	1	1	1	0	1	1	b1756	putative ATP-binding component of a transport system
b1757	1	0	1	1	1	1	1	1	1	1	1	1	1	1	1	1	1	1	1	1	1	1	1	1	1	1	1	1	1	1	1	1	1	1	1	1	1	1	1	1	1	1	1	b1757	putative thiosulfate sulfur transferase
b1758	1	0	1	1	1	1	1	1	1	1	1	1	1	1	1	1	0	1	1	1	1	1	1	1	1	1	1	1	1	1	1	1	1	1	1	1	1	1	1	1	1	1	1	b1758	putative cytochrome oxidase
b1759	1	1	1	1	1	1	1	1	1	1	1	1	1	1	1	1	0	1	1	1	1	1	1	1	1	1	1	1	1	1	1	1	1	1	1	1	0	1	1	1	1	1	1	b1759	orf, hypothetical protein
b1760	1	0	1	1	1	1	1	1	1	1	1	1	1	1	1	1	1	1	1	1	1	1	1	1	1	1	1	1	1	1	1	1	1	1	1	1	1	1	1	1	1	1	1	b1760	orf, hypothetical protein
b1762	1	0	1	0	1	1	1	1	1	1	1	1	1	0	0	1	1	1	1	1	1	1	1	1	1	1	1	1	1	1	1	1	1	1	1	1	1	1	1	1	1	1	1	b1762	orf, hypothetical protein
b1767	1	1	1	1	1	0	1	1	1	1	1	1	1	1	1	1	0	1	1	1	1	1	1	1	0	1	1	1	1	1	1	1	1	1	1	1	1	1	1	1	1	1	1	ansA	cytoplasmic L-asparaginase I
b1769	1	1	0	1	1	0	0	0	0	0	0	0	0	0	0	0	0	0	0	0	0	0	0	0	1	1	1	1	1	1	1	1	1	1	1	1	1	1	1	1	1	1	1	ydjE	putative transport protein
b1770	1	1	0	1	1	0	0	0	0	0	0	0	0	0	0	0	0	0	0	0	0	0	0	0	1	1	1	1	1	1	1	1	1	1	1	1	1	1	1	1	1	1	1	b1770	putative DEOR-type transcriptional regulator
b1771	1	1	0	1	1	0	0	0	0	0	0	0	0	0	0	0	0	0	0	0	0	0	0	0	1	1	1	1	1	1	1	1	1	1	1	1	1	1	1	1	1	1	1	b1771	orf, hypothetical protein
b1772	1	1	0	1	1	0	0	0	0	0	0	0	0	0	0	0	0	0	0	0	0	0	0	0	1	1	1	1	1	1	1	1	1	1	1	1	1	1	1	1	1	1	1	b1772	putative kinase
b1773	0	1	0	0	1	0	0	0	0	0	0	0	0	0	0	0	0	0	0	0	0	0	0	0	1	1	1	1	1	1	1	1	1	1	1	1	1	1	1	1	1	1	1	b1773	putative aldolase
b1774	1	0	0	0	0	0	0	0	0	0	0	0	0	0	0	0	0	0	0	0	0	0	0	0	1	1	1	1	1	1	1	1	1	1	1	1	1	1	1	1	1	1	1	ydjJ	putative oxidoreductase
b1775	1	1	0	1	0	0	0	0	0	0	0	0	0	0	0	0	0	0	0	0	0	0	0	0	1	1	1	1	1	1	1	1	1	1	1	1	1	1	1	1	1	1	1	b1775	putative transport protein
b1776	1	1	0	1	1	0	0	0	0	0	0	0	0	0	0	0	0	0	0	0	0	0	0	0	1	1	1	1	1	1	1	1	1	1	1	1	1	1	1	1	1	1	1	b1776	putative oxidoreductase
b1779	1	1	1	1	1	0	1	0	1	1	1	1	1	1	1	1	1	1	1	1	1	1	1	1	1	1	1	1	1	1	1	1	1	1	1	1	1	1	1	1	1	1	1	gapA	glyceraldehyde-3-phosphate dehydrogenase A
b1781	1	0	1	1	1	1	1	1	1	1	1	1	1	1	1	1	1	1	1	1	1	1	1	1	1	1	1	1	1	1	1	1	1	1	1	1	1	1	1	1	1	1	1	b1781	putative an aldehyde reductase
b1783	0	1	1	1	1	1	1	1	1	1	1	1	1	1	1	1	0	1	1	1	1	1	1	1	1	0	0	1	1	1	1	1	1	1	0	1	1	1	1	1	1	1	1	yeaG	orf, hypothetical protein
b1784	0	1	1	1	1	1	1	1	1	0	1	1	1	1	1	1	0	1	1	1	1	1	1	1	1	1	1	1	1	1	1	1	0	0	0	1	0	0	1	0	1	1	1	yeaH	orf, hypothetical protein
b1785	0	1	1	1	1	1	1	1	1	1	1	1	1	1	1	1	0	1	1	1	1	1	1	1	1	1	1	1	1	1	1	1	0	0	0	1	0	0	1	0	1	1	1	yeaI	orf, hypothetical protein
b1786	0	1	1	1	1	1	1	1	1	1	1	1	1	1	1	1	0	1	1	1	1	1	1	1	1	1	1	1	1	1	1	1	0	0	1	1	1	0	1	1	1	1	1	yeaJ	orf, hypothetical protein
b1787	1	1	1	1	1	1	1	1	1	1	1	1	1	1	1	1	1	1	1	1	1	1	1	1	1	1	1	1	1	1	1	1	0	0	1	1	1	0	1	1	1	1	1	yeaK	orf, hypothetical protein
b1788	1	1	1	1	1	1	1	1	1	1	1	1	1	1	1	1	1	1	1	1	1	1	1	1	1	1	1	1	1	1	1	1	0	0	1	1	1	0	1	1	1	1	1	b1788	orf, hypothetical protein
b1789	1	1	1	1	1	1	1	1	1	1	1	1	1	1	1	1	1	1	1	1	1	1	1	1	1	1	1	1	1	1	1	1	0	0	1	1	1	0	1	1	1	1	1	yeaL	orf, hypothetical protein
b1790	1	1	1	1	1	1	1	1	1	1	1	1	0	1	1	1	0	1	1	1	1	0	1	1	1	1	1	1	1	1	1	1	1	0	1	1	1	1	1	1	1	1	1	yeaM	putative ARAC-type regulatory protein
b1791	1	0	1	1	1	1	1	1	1	1	1	1	1	1	1	1	1	1	1	1	1	1	1	1	1	1	1	1	1	1	1	1	0	0	1	1	1	0	1	1	1	1	1	yeaN	putative amino acid/amine transport protein
b1792	1	1	1	1	1	1	1	1	1	1	1	1	1	1	1	1	1	0	1	1	1	1	1	1	1	1	1	0	0	1	0	1	0	0	1	1	1	0	1	1	1	1	1	yeaO	orf, hypothetical protein
b1793	1	1	1	1	1	1	1	1	1	1	1	1	1	1	1	1	1	1	1	1	1	1	1	1	1	1	1	1	1	1	1	1	0	0	1	1	1	0	1	1	1	1	1	yoaF	orf, hypothetical protein
b1794	1	1	1	1	1	0	0	0	0	0	0	0	0	0	0	0	0	0	0	0	0	0	0	0	1	1	1	1	1	1	1	1	0	0	1	1	1	0	1	1	1	1	1	yeaP	orf, hypothetical protein
b1795	1	1	1	1	1	1	1	1	1	1	1	1	1	1	1	1	1	1	1	1	1	1	1	1	1	1	1	1	1	1	1	1	0	0	0	0	0	0	0	0	0	1	0	yeaQ	orf, hypothetical protein
b1796	1	1	1	1	1	1	1	1	1	1	1	1	1	1	1	1	1	1	1	1	0	1	1	1	1	1	1	1	1	1	1	1	0	0	0	0	0	0	0	0	0	0	0	yoaG	orf, hypothetical protein
b1797	1	1	1	1	1	1	1	1	1	1	1	1	1	1	1	1	1	1	1	1	0	1	1	1	1	1	1	1	1	1	1	1	0	0	0	0	0	0	0	0	0	0	0	yeaR	orf, hypothetical protein
b1798	1	1	1	1	1	1	1	1	1	1	1	1	1	1	1	1	1	0	0	0	0	0	0	0	1	1	1	1	1	1	1	1	0	0	0	0	0	0	0	0	0	0	0	yeaS	orf, hypothetical protein
b1799	1	1	1	1	0	1	1	1	1	1	1	1	1	1	1	1	1	0	0	0	0	0	0	0	1	1	1	1	1	1	1	1	0	0	0	0	0	0	0	0	0	0	0	yeaT	putative transcriptional regulator LYSR-type
b1800	1	1	1	1	1	1	1	1	1	1	1	1	1	1	1	1	1	0	0	0	0	0	0	0	1	1	1	1	1	1	1	1	0	0	0	0	0	0	0	0	0	0	0	yeaU	putative tartrate dehydrogenase
b1801	1	1	1	1	0	1	1	1	1	1	1	1	1	1	1	1	1	0	0	0	0	0	0	0	1	1	1	1	1	1	1	1	0	0	0	0	0	0	0	0	0	0	0	yeaV	putative transport protein
b1802	1	1	1	1	1	1	1	1	1	1	1	1	1	1	1	1	1	0	0	0	0	0	0	0	1	1	1	1	1	1	1	1	1	1	1	1	1	1	1	1	1	1	1	yeaW	orf, hypothetical protein
b1803	1	1	1	1	1	1	1	1	1	1	1	1	1	1	1	1	1	0	0	0	0	0	0	0	1	1	1	1	1	1	1	1	1	1	1	1	1	1	1	1	1	1	1	yeaX	putative diogenase beta subunit
b1806	1	1	1	1	1	1	1	1	1	1	1	1	1	1	1	1	1	1	1	1	1	1	1	1	1	1	1	1	1	1	1	1	1	1	1	1	1	1	1	1	0	1	1	b1806	putative outer membrane protein
b1807	1	1	1	1	1	0	1	1	1	1	1	1	1	1	1	1	1	1	1	1	1	1	1	1	1	1	1	1	1	1	1	1	1	1	1	1	1	1	1	1	1	1	1	yeaZ	orf, hypothetical protein
b1808	1	1	1	1	1	1	1	1	1	1	1	1	1	1	1	1	1	1	1	1	1	1	1	1	1	1	1	1	1	1	1	1	0	1	1	1	1	1	1	1	1	1	1	b1808	putative enzyme
b1810	1	1	1	1	1	1	0	1	1	1	1	1	1	1	1	1	1	1	1	1	1	1	1	0	1	1	1	1	1	1	1	1	1	1	1	1	1	1	1	1	1	1	1	b1810	orf, hypothetical protein
b1811	1	1	1	1	1	1	1	1	1	1	1	1	1	1	1	1	1	1	1	1	1	1	1	1	1	1	1	1	1	1	1	1	0	1	1	1	1	1	1	1	1	1	1	b1811	orf, hypothetical protein
b1821	1	1	1	1	1	1	1	1	1	1	1	1	1	1	1	1	0	1	1	1	1	1	1	1	1	1	1	1	1	1	1	1	1	1	1	1	1	1	1	1	1	1	1	b1821	orf, hypothetical protein
b1822	1	1	1	1	1	1	1	1	1	1	1	1	1	1	1	1	0	1	1	1	1	1	1	1	1	1	1	1	1	1	1	1	1	1	1	1	1	1	1	1	1	1	1	yebH	putative enzyme
b1823	1	1	1	1	0	1	1	1	1	1	1	1	1	1	1	1	0	1	1	1	1	1	1	1	1	1	1	1	1	1	1	1	1	1	1	1	1	1	1	1	1	1	1	cspC	cold shock protein
b1839	1	1	1	0	1	1	1	1	1	1	1	1	1	1	1	1	1	1	1	1	0	1	1	1	1	1	1	1	1	1	1	1	1	1	1	1	1	1	1	1	1	1	1	-	orf, hypothetical protein
b1840	1	1	1	0	1	1	1	1	1	1	1	1	1	1	1	1	1	1	1	1	0	1	1	1	1	1	1	1	1	1	1	1	1	1	1	1	1	1	1	1	1	1	1	-	putative resistance protein
b1841	1	1	1	0	1	1	1	1	1	1	1	1	1	1	1	1	1	1	1	1	0	1	1	1	1	1	1	1	1	1	1	1	1	1	1	1	1	1	1	1	1	1	1	-	orf, hypothetical protein
b1842	1	1	1	1	1	1	1	1	1	1	1	1	1	1	1	1	1	1	1	1	0	1	1	1	1	1	1	1	1	1	1	1	1	1	1	1	1	1	1	1	1	1	1	holE	DNA polymerase III, theta subunit
b1843	1	1	1	1	1	1	1	1	1	1	1	1	1	1	1	1	1	1	1	1	0	1	1	1	1	1	1	1	1	1	1	1	1	1	1	1	1	1	1	1	1	1	1	-	orf, hypothetical protein
b1844	1	1	1	1	1	1	1	1	1	1	1	1	1	1	1	1	1	1	1	1	0	1	1	1	1	1	1	1	1	1	1	1	1	1	1	1	1	1	1	1	1	1	1	-	orf, hypothetical protein
b1845	1	1	1	1	1	1	1	1	1	1	1	1	1	1	1	1	1	1	1	1	0	1	1	1	1	1	1	1	1	1	1	1	1	1	1	1	1	1	1	1	1	1	1	ptrB	protease II
b1846	1	0	1	1	1	1	1	1	1	1	1	1	1	1	1	1	1	1	1	1	0	1	1	1	1	1	1	1	1	1	1	1	1	1	1	1	1	1	1	1	1	1	1	yebE	orf, hypothetical protein
b1847	1	1	1	1	1	1	1	1	1	1	1	1	1	1	1	1	1	1	1	1	0	1	1	1	1	1	1	1	1	1	1	1	1	1	1	1	1	1	1	1	1	1	1	yebF	orf, hypothetical protein
b1848	1	1	1	1	1	1	1	1	1	1	1	1	1	1	1	1	1	1	1	1	0	1	1	1	1	1	1	1	1	1	1	1	1	1	1	1	1	1	1	1	1	1	1	yebG	orf, hypothetical protein
b1849	1	1	1	1	1	1	1	1	1	1	1	1	1	1	1	1	1	1	1	1	0	1	1	1	1	1	1	1	1	1	1	1	1	1	1	1	1	1	1	1	1	1	1	purT	phosphoribosylglycinamide formyltransferase 2
b1850	1	1	1	1	1	1	1	1	1	1	1	1	1	1	1	1	1	1	1	1	0	1	1	1	1	1	1	1	1	1	1	1	1	1	1	1	1	1	1	1	1	1	1	eda	2-keto-3-deoxygluconate 6-phosphate aldolase and 2-keto-4-hydroxyglutarate aldolase
b1851	1	1	1	1	1	1	1	1	1	1	1	1	1	1	1	1	1	1	1	1	0	1	1	1	1	1	1	1	1	1	1	1	1	1	1	1	1	1	1	1	1	1	1	edd	6-phosphogluconate dehydratase
b1852	1	1	1	1	1	1	1	1	1	1	1	1	1	1	1	1	1	1	1	1	0	1	1	1	1	1	1	1	1	1	1	1	1	1	1	1	1	1	1	1	1	1	1	zwf	glucose-6-phosphate dehydrogenase
b1853	1	1	1	1	1	1	1	1	1	1	1	1	1	1	1	1	1	1	1	1	0	1	1	1	1	1	1	1	1	1	1	1	1	1	1	1	1	1	1	1	1	1	1	yebK	orf, hypothetical protein
b1854	1	1	1	1	1	1	1	1	1	1	1	1	1	1	1	1	1	1	1	1	0	1	1	1	1	1	1	1	1	1	1	1	1	1	1	1	1	1	1	1	1	1	1	pykA	pyruvate kinase II, glucose stimulated
b1861	1	1	1	1	1	1	1	1	1	1	1	1	0	1	1	1	1	1	1	1	1	1	1	1	1	1	1	1	1	1	1	1	1	1	1	1	1	1	1	1	1	1	1	ruvA	Holliday junction helicase subunit B; branch migration; repair
b1862	1	1	1	1	1	0	1	1	1	1	1	1	0	0	1	1	1	1	1	1	1	1	1	1	1	1	1	1	1	1	1	1	1	1	1	1	1	1	1	1	1	1	1	yebB	orf, hypothetical protein
b1864	1	1	1	1	1	1	1	1	1	1	1	1	1	1	1	1	1	1	1	1	1	1	1	1	1	1	1	1	1	1	1	1	1	1	1	1	1	1	1	0	1	1	1	yebC	orf, hypothetical protein
b1867	0	1	1	1	1	1	1	1	1	1	1	1	1	1	1	1	1	1	1	1	1	1	1	0	1	1	1	1	1	1	1	1	1	1	1	1	1	1	1	1	1	1	1	yecD	orf, hypothetical protein
b1868	0	1	0	1	1	1	0	1	0	1	1	1	0	0	0	1	1	0	0	1	1	1	1	1	1	1	1	1	1	1	1	1	1	0	0	0	1	1	1	1	1	1	1	yecE	orf, hypothetical protein
b1872	1	1	1	1	1	0	0	0	0	1	1	1	1	1	1	0	0	1	1	1	1	1	1	1	1	1	1	1	1	1	1	1	0	1	1	1	1	1	1	1	1	1	1	bisZ	biotin sulfoxide reductase 2
b1873	1	0	1	1	1	1	1	1	1	1	1	1	1	1	1	1	1	1	1	1	1	1	1	1	1	1	1	1	1	1	1	1	1	1	1	1	1	1	1	1	1	1	1	yecK	putative cytochrome C-type protein
b1877	1	0	1	0	1	1	1	1	1	1	1	1	1	1	1	1	1	1	1	1	1	1	1	1	1	0	0	1	1	1	1	1	1	1	1	0	1	1	1	1	1	1	1	yecT	orf, hypothetical protein
b1878	1	1	0	1	1	1	1	1	1	1	1	1	1	1	1	1	1	1	1	1	1	1	1	1	1	1	1	1	1	1	1	1	1	1	1	1	1	1	1	1	0	1	1	flhE	flagellar protein
b1879	1	1	0	1	1	1	1	1	1	1	1	1	1	1	1	1	1	1	1	1	1	1	1	1	1	0	1	1	1	1	1	1	1	1	1	1	1	1	1	1	0	1	1	flhA	flagellar biosynthesis; possible export of flagellar proteins
b1880	0	1	1	1	1	1	1	1	1	1	1	1	1	1	1	1	1	1	1	1	1	1	1	1	1	0	1	1	1	1	1	1	1	1	1	1	1	1	1	1	0	1	1	flhB	putative part of export apparatus for flagellar proteins
b1881	0	0	1	1	1	1	1	1	1	1	1	1	1	1	1	1	1	1	1	1	1	1	1	1	1	0	1	1	1	1	1	1	1	1	1	1	1	1	1	1	0	1	1	cheZ	chemotactic response; CheY protein phophatase; antagonist of CheY as switch regulator
b1882	0	0	1	1	1	1	1	1	1	1	1	1	1	1	1	1	1	1	1	1	1	1	1	1	1	0	0	1	1	1	1	1	1	1	1	1	1	1	1	1	0	1	1	cheY	chemotaxis regulator transmits chemoreceptor signals to flagelllar motor components
b1883	0	0	1	1	1	1	1	1	1	1	1	1	1	1	1	1	1	1	1	1	1	1	1	1	1	1	0	1	1	1	1	1	1	1	1	1	1	1	1	1	0	1	1	cheB	response regulator for chemotaxis (cheA sensor); protein methylesterase
b1884	0	0	1	1	1	1	1	1	1	1	1	1	1	1	1	1	1	1	1	1	1	1	1	1	1	0	0	1	1	1	1	1	1	1	1	1	1	1	1	1	0	1	1	cheR	response regulator for chemotaxis; protein glutamate methyltransferase
b1885	1	0	1	1	1	1	1	1	1	1	1	1	0	1	0	0	1	1	1	1	1	1	1	1	1	1	0	1	1	1	1	1	1	1	1	1	1	1	1	1	0	1	1	tap	methyl-accepting chemotaxis protein IV, peptide sensor receptor
b1886	1	0	1	1	1	1	1	1	1	1	1	1	0	1	0	0	1	1	1	1	1	1	1	1	1	1	1	1	1	1	1	1	1	1	1	1	1	1	1	1	0	1	1	tar	methyl-accepting chemotaxis protein II, aspartate sensor receptor
b1887	0	0	1	1	1	1	1	1	1	1	1	1	0	0	0	0	1	1	1	1	1	1	1	1	1	1	1	1	1	1	1	1	1	1	1	1	1	1	1	1	0	1	1	cheW	positive regulator of CheA protein activity
b1888	0	0	1	0	1	1	1	1	1	1	1	1	0	0	0	0	1	1	1	1	1	1	1	1	1	1	1	1	1	1	1	1	1	1	1	1	1	1	1	1	0	1	1	cheA	sensory transducer kinase between chemo- signal receptors and CheB and CheY
b1889	1		1	0	1	0	1	1	0	1	1	1	0	0	0	0	1	1	1	1	1	1	1	1	1	1	1	1	1	1	1	1	1	1	1	1	1	1	0	1	0	1	1	motB	enables flagellar motor rotation, linking torque machinery to cell wall
b1890	1	1	1	0	1	0	0	0	0	0	0	0	0	0	0	0	1	1	1	1	1	1	1	1	1	1	1	1	1	1	1	1	1	1	1	1	1	1	1	1	0	1	1	motA	proton conductor component of motor; no effect on switching
b1891	1	1	1	0	1	0	0	0	0	0	0	0	1	1	1	1	1	1	1	1	1	1	1	1	1	1	1	1	1	1	1	1	1	1	1	1	1	1	1	1	0	1	1	flhC	regulator of flagellar biosynthesis acting on class 2 operons; transcription initiation factor
b1892	1	1	1	0	1	0	0	0	0	0	0	0	1	1	1	1	1	1	1	1	1	1	1	1	1	1	1	1	1	1	1	1	1	1	1	1	1	1	1	1	0	1	1	flhD	regulator of flagellar biosynthesis, acting on class 2 operons; transcriptional initiation factor
b1895	1	1	1	0	1	0	0	0	0	0	0	0	1	1	1	1	1	1	1	1	1	1	1	1	1	1	1	0	1	1	1	1	1	1	0	0	1	1	1	1	0	1	1	yecG	putative regulator
b1896	1	1	1	0	1	1	1	0	0	1	1	1	1	1	1	1	1	1	1	1	1	1	1	1	1	1	1	1	1	1	1	1	1	1	0	0	1	1	1	1	0	1	1	otsA	trehalose-6-phosphate synthase
b1897	1	1	1	0	1	1	1	1	1	1	1	1	1	1	1	1	1	1	1	1	1	1	1	1	1	1	1	1	1	1	1	1	1	1	1	1	1	1	1	1	0	1	1	otsB	trehalose-6-phosphate phophatase, biosynthetic
b1898m	1	1	1	1	1	1	1	1	1	1	1	1	1	1	1	1	1	1	1	1	1	1	1	1	1	1	1	0	1	1	1	1	1	1	1	1	1	1	1	1	0	1	1	araH	ABC-type arabinose transport system, permease component
b1900	1	1	1	1	1	1	1	1	1	1	1	1	1	1	1	1	1	1	1	1	1	1	1	1	1	1	1	0	1	1	1	1	1	1	1	1	1	1	1	1	0	1	1	araG	ATP-binding component of high-affinity L-arabinose transport system
b1918	1	1	1	1	1	1	1	1	1	1	1	1	1	1	1	1	0	0	1	1	1	1	1	1	1	1	1	1	1	1	1	1	1	1	1	1	1	1	1	1	1	1	1	yecS	putative transport system permease protein (former yecC)
b1923	1	1	0	1	1	1	0	1	1	1	1	1	1	1	1	1	1	0	0	1	1	1	0	1	1	1	1	1	1	1	1	1	1	1	1	0	1	1	1	1	1	1	1	fliC	flagellar biosynthesis; flagellin, filament structural protein
b1924	1	1	0	1	1	1	1	1	1	1	1	1	1	1	1	1	1	1	1	1	1	1	1	1	1	1	1	1	1	1	1	0	1	1	1	1	1	1	1	1	1	1	1	fliD	flagellar biosynthesis; filament capping protein; enables filament assembly
b1925	1	1	1	1	1	1	1	1	1	1	1	1	1	1	1	1	1	1	1	1	1	1	1	1	1	1	1	1	1	1	1	0	1	1	1	1	1	1	1	1	1	1	1	fliS	flagellar biosynthesis; repressor of class 3a and 3b operons (RflA activity)
b1926	1	1	1	1	1	1	1	1	1	1	1	1	1	1	1	1	1	1	1	1	1	1	1	1	1	1	1	1	1	1	1	0	1	1	1	1	1	1	1	1	1	1	1	fliT	flagellar biosynthesis; repressor of class 3a and 3b operons (RflA activity)
b1927	1	1	1	1	1	1	1	1	1	1	1	1	1	1	1	1	1	1	1	1	1	1	1	1	1	1	1	1	1	1	1	0	1	1	1	1	1	1	1	1	1	1	1	amyA	cytoplasmic alpha-amylase
b1928	1	1	1	1	1	1	1	1	1	1	1	1	1	1	1	1	1	1	1	1	1	1	1	1	1	1	1	1	1	1	1	0	1	1	1	1	1	1	1	1	1	1	1	yedD	orf, hypothetical protein
b1929	1	1	1	1	1	1	1	1	1	1	1	1	1	1	1	1	1	1	1	1	1	1	1	1	1	1	1	1	1	1	1	0	1	1	1	1	1	1	1	1	1	1	1	yedE	putative transport system permease protein
b1931	1	0	1	1	1	1	1	1	1	1	1	1	1	1	1	1	1	1	1	1	1	1	1	1	1	1	1	1	1	1	1	0	0	1	1	1	1	1	1	1	1	1	1	yedK	orf, hypothetical protein

b1932	0	0	0	0	0	0	0	0	0	0	0	0	0	0	0	0	0	0	0	0	0	0	0	0	0	0	0	0	0	0	0	0	0	0	0	0	0	0	0	0	0	0	0	yedL	orf, hypothetical protein
b1933	0	0	0	0	0	0	0	0	0	0	0	0	0	0	0	0	0	0	0	0	0	0	0	0	1	0	0	0	0	1	0	0	0	0	0	0	0	0	0	0	0	0	0	b1933	orf, hypothetical protein
b1934	0	0	0	0	0	0	0	0	0	0	0	0	0	0	0	0	0	0	0	0	0	0	0	0	1	0	0	0	0	1	0	0	0	0	0	0	0	0	0	0	0	0	0	yedN	orf, hypothetical protein
b1935	1	0	0	0	0	0	0	0	0	0	0	0	0	0	0	0	0	0	0	0	0	0	0	0	1	0	0	0	0	1	0	0	0	0	0	0	0	0	0	0	0	0	0	yedM	orf, hypothetical protein
b1936	0	1	1	0	1	0	0	0	0	0	0	0	1	1	0	1	1	0	0	0	1	0	1	1	1	1	1	1	1	1	1	0	1	1	1	0	0	1	1	1	1	0	0	b1936	orf, hypothetical protein
b1937	0	1	1	1	1	0	0	0	0	0	0	0	1	1	1	1	1	1	1	1	1	0	1	1	1	1	1	1	1	1	1	1	1	1	1	1	1	1	1	1	1	0	0	fliE	flagellar biosynthesis; basal-body component, possibly at (MS-ring)-rod junction
b1938	0	1	1	1	1	0	0	0	0	0	0	0	1	1	1	1	1	1	1	1	1	0	1	1	1	1	1	1	1	1	1	1	1	1	1	1	0	1	1	0	1	0	0	fliF	flagellar biosynthesis; basal-body MS(membrane and supramembrane)-ring and collar protein
b1939	0	1	1	1	1	0	0	0	0	0	0	0	1	1	1	1	1	1	1	1	1	0	1	1	1	1	1	1	1	1	1	1	1	1	1	1	1	1	1	1	1	0	0	fliG	flagellar biosynthesis, component of motor switching and energizing, enabling rotation and determining its direction
b1940	0	1	1	1	1	0	0	0	0	0	0	0	1	1	1	1	1	1	1	1	1	0	1	1	1	1	1	1	1	1	1	1	1	1	1	1	1	1	1	1	1	0	0	fliH	flagellar biosynthesis; export of flagellar proteins?
b1941	0	1	1	1	1	0	0	0	0	0	0	1	0	1	1	0	1	1	1	1	1	0	1	1	0	0	1	0	0	1	1	1	1	1	1	1	1	1	1	1	1	1	1	fliI	flagellum-specific ATP synthase
b1942	0	1	1	1	1	0	0	0	0	0	0	0	0	1	1	1	1	1	1	1	1	0	1	1	1	1	1	1	1	1	1	1	1	1	1	1	1	1	1	1	1	0	0	fliJ	flagellar fliJ protein
b1943	0	1	1	1	1	0	0	0	0	0	0	0	0	1	1	1	1	1	1	1	1	0	1	1	1	1	1	1	1	1	1	1	1	1	1	1	1	1	0	1	1	0	0	fliK	flagellar hook-length control protein
b1944	0	1	1	1	1	0	0	0	0	0	0	0	0	1	1	1	1	1	1	1	1	0	1	1	1	1	1	1	1	1	0	1	1	1	1	1	1	1	1	1	1	0	0	fliL	flagellar biosynthesis
b1945	0	1	1	1	1	1	0	1	1	1	1	1	0	1	1	1	1	1	1	1	1	0	1	1	1	1	1	1	1	1	1	1	1	1	1	1	1	1	1	1	1	0	0	fliM	flagellar biosynthesis, component of motor switch and energizing, enabling rotation and determining its direction
b1946	0	1	1	1	1	1	1	1	1	1	1	1	0	1	1	1	1	1	1	1	1	0	1	1	1	1	1	1	1	1	1	1	1	1	1	1	1	1	1	1	1	0	0	fliN	flagellar biosynthesis, component of motor switch and energizing, enabling rotation and determining its direction
b1947	0	1	1	1	1	1	1	1	1	1	1	1	0	1	1	1	1	1	1	1	1	0	1	1	1	1	1	1	1	1	1	1	1	1	1	1	1	1	1	1	1	0	0	fliO	flagellar biosynthesis
b1948	0	1	1	1	1	1	1	1	1	1	1	1	0	1	1	1	1	1	1	1	1	0	1	1	1	1	1	1	1	1	1	1	1	1	1	1	1	1	1	1	1	0	0	fliP	flagellar biosynthesis
b1949	0	1	1	1	1	1	1	1	1	1	1	1	0	1	1	1	1	1	1	1	1	0	1	1	1	1	1	1	1	1	1	1	1	1	1	1	1	1	1	1	1	0	0	fliQ	flagellar biosynthesis
b1950	0	1	1	1	1	1	1	1	1	1	1	1	0	1	1	1	1	1	1	1	1	0	1	1	1	1	1	1	1	1	1	1	1	1	1	1	1	1	1	1	1	0	0	fliR	flagellar biosynthesis
b1951	1	0	1	1	1	1	1	1	1	0	1	1	0	1	1	1	1	1	1	1	1	0	1	1	1	1	0	1	1	1	1	1	1	1	1	1	1	1	1	1	1	0	0	rcsA	positive regulator for ctr capsule biosynthesis, positive transcription factor
b1952	1	1	1	1	1	1	1	1	1	1	1	1	0	1	1	1	1	1	1	1	1	1	1	1	1	1	1	1	1	1	1	1	1	1	1	1	1	1	1	1	1	1	1	dsrB	orf, hypothetical protein
b1953	1	1	1	1	1	1	1	1	1	1	1	1	0	1	1	1	1	1	1	1	1	1	1	1	1	1	1	1	1	1	1	1	1	1	1	1	1	1	1	1	1	1	1	b1953	orf, hypothetical protein
b1956	1	1	1	1	1	1	1	1	1	1	1	1	0	1	1	1	0	1	1	1	1	1	1	1	1	1	1	1	1	1	1	1	1	1	1	1	1	1	1	1	1	1	1	b1956	orf, hypothetical protein
b1957	1	1	1	1	1	1	1	1	1	1	1	1	0	1	1	1	0	1	1	1	1	1	1	1	1	1	1	1	1	1	1	1	1	1	1	1	1	1	1	1	1	1	1	b1957	orf, hypothetical protein
b1958	1	1	1	1	1	1	1	1	1	1	1	1	0	1	1	1	0	1	1	1	1	1	1	1	1	1	0	0	0	1	1	1	1	1	1	1	1	1	1	1	1	1	1	yedI	orf, hypothetical protein
b1959	1	1	1	1	1	1	1	1	1	1	1	1	0	1	1	1	0	1	1	1	1	1	1	1	1	1	1	1	1	1	1	1	1	1	1	1	1	1	1	1	1	1	1	yedA	putative transmembrane subunit
b1960	1	1	1	1	1	1	1	1	1	1	1	1	0	1	1	1	0	1	1	1	1	1	1	1	1	1	0	1	1	1	1	1	0	1	1	1	1	1	1	1	1	1	1	vsr	DNA mismatch endonuclease, patch repair protein
b1961	1	1	1	1	1	1	1	1	1	1	1	1	0	1	1	1	0	1	1	1	1	1	1	1	1	1	1	1	1	1	1	1	1	1	1	1	0	1	1	1	1	1	1	dcm	DNA cytosine methylase
b1962	1	1	1	1	1	1	1	1	1	1	1	1	0	1	1	1	0	1	1	1	1	1	1	1	1	1	1	1	1	1	1	1	0	1	1	1	1	1	1	1	1	1	1	yedJ	orf, hypothetical protein
b1963	1	1	1	1	1	1	1	1	1	1	1	1	0	1	1	1	0	1	1	1	1	1	1	1	1	1	1	1	1	1	1	1	0	1	1	1	1	1	1	1	1	1	1	b1963	orf, hypothetical protein
b1964	0	1	1	1	1	1	1	1	1	1	1	1	0	0	1	1	1	0	0	1	1	1	1	1	1	0	1	1	1	1	1	1	1	0	1	1	1	1	1	1	1	1	1	b1964	putative outer membrane protein
b1966	1	1	1	1	1	1	0	1	1	1	1	1	0	1	0	1	1	0	0	0	1	0	1	1	0	0	1	1	1	1	1	1	1	1	1	0	1	1	1	1	1	1	1	b1966	putative outer membrane protein
b1967	0	1	1	1	1	1	1	1	1	1	1	1	1	0	0	1	1	1	1	1	1	1	1	1	1	1	1	1	1	1	1	1	1	1	1	1	0	0	0	1	1	1	1	yedU	orf, hypothetical protein
b1968	0	1	1	1	0	1	1	1	1	1	1	1	0	1	1	1	0	1	1	1	1	1	1	1	1	1	1	1	1	1	1	1	1	1	1	1	1	1	1	1	1	1	1	yedV	putative 2-component sensor protein
b1969	1	1	1	1	0	1	1	1	1	1	1	1	0	1	1	1	0	1	1	1	1	1	1	1	1	1	1	1	1	1	1	1	1	1	1	1	1	1	1	1	1	1	1	yedW	putative 2-component transcriptional regulator
b1970	1	1	1	1	1	1	1	1	1	1	1	1	0	1	1	1	0	1	1	1	0	1	1	1	1	1	1	1	1	1	1	1	1	1	1	1	1	1	1	1	1	1	1	b1970	orf, hypothetical protein
b1971	1	1	1	1	1	1	1	1	1	1	1	1	1	1	1	1	1	1	1	1	1	1	1	1	1	1	1	1	1	1	1	1	1	1	1	1	0	1	1	1	1	1	1	b1971	putative reductase
b1972	1	1	1	1	1	1	1	1	1	1	1	1	0	1	1	0	0	1	1	1	0	1	1	1	1	1	1	1	1	1	1	1	1	1	1	1	1	1	1	1	1	1	1	b1972	orf, hypothetical protein
b1973	1	1	1	1	1	1	1	1	1	1	1	1	0	1	1	1	0	1	1	1	0	1	1	1	1	1	1	1	1	1	1	1	1	1	1	1	1	1	1	1	1	1	1	b1973	orf, hypothetical protein
b1974	0	0	1	1	1	0	0	0	0	0	0	0	0	1	1	1	0	1	1	1	1	1	1	1	1	1	1	1	1	1	1	1	0	0	0	0	0	0	0	0	0	0	0	yodB	putative cytochrome
b1976	1	1	1	1	1	1	1	1	1	1	1	1	0	1	1	1	0	1	1	1	1	1	1	1	1	1	1	0	1	1	1	1	1	1	1	1	1	1	1	1	1	1	1	b1976	orf, hypothetical protein
b1978	0	0	1	1	0	1	1	1	0	1	1	1	0	0	1	0	0	1	1	1	1	1	1	1	1	0	1	0	1	1	1	1	0	0	0	0	0	0	0	0	0	0	0	b1978	putative factor
b1979	0	0	1	0	1	0	0	0	1	1	1	0	0	0	0	0	1	1	1	1	0	1	1	1	1	1	1	1	1	1	1	0	0	0	0	0	0	0	0	0	0	0	0	b1979	orf, hypothetical protein
b1980	0	0	1	0	1	0	0	0	1	1	1	0	0	0	0	0	1	1	1	1	1	1	1	0	1	1	1	1	1	1	1	0	0	0	0	0	0	0	0	0	0	0	0	b1980	orf, hypothetical protein
b1981	1	1	1	0	1	0	0	1	1	1	1	0	0	0	0	0	1	1	1	1	0	1	1	1	1	1	1	1	1	1	1	0	0	0	0	0	0	0	0	0	0	0	0	shiA	putative transport protein, shikimate
b1982	1	1	1	1	1	1	1	1	1	1	1	1	0	0	0	0	1	1	1	1	0	1	1	1	1	1	1	1	1	1	1	0	1	1	1	1	1	1	1	1	1	1	1	amn	AMP nucleosidase
b1983	0	0	1	1	1	1	1	1	1	1	1	1	1	1	1	1	1	1	1	1	1	1	1	1	1	1	1	1	1	1	1	1	1	1	1	1	1	1	1	1	1	1	1	b1983	orf, hypothetical protein
b1987	1	0	1	1	1	1	1	1	1	1	1	1	0	0	1	1	1	1	1	1	1	1	1	1	1	1	1	1	1	1	1	1	1	1	1	1	0	1	1	1	1	1	1	cbl	transcriptional regulator cys regulon; accessory regulatory circuit affecting cysM
b1988	1	0	1	1	1	1	1	1	1	1	1	1	1	0	0	0	1	1	1	1	1	1	1	1	1	1	1	1	1	1	1	1	1	1	1	1	1	1	1	1	1	1	1	nac	nitrogen assimilation control protein
b1991	1	0	1	1	1	1	1	1	1	1	1	1	1	1	1	1	1	1	1	1	1	1	1	1	1	1	1	1	1	1	1	1	1	1	1	1	1	1	1	1	1	1	1	cobT	nicotinate-nucleotide dimethylbenzimidazole-P phophoribosyl transferase
b1993	1	1	1	1	1	1	1	1	1	1	1	1	1	1	1	1	0	1	1	1	1	1	1	1	1	1	1	1	1	1	1	1	1	1	1	1	1	1	1	1	1	1	1	cobU	cobinamide kinase/cobinamide phosphate guanylyltransferase
b1995	0	0	0	1	0	1	1	1	1	1	1	1	1	0	1	1	1	1	1	1	1	1	1	1	1	1	0	1	0	1	0	1	0	0	0	1	1	1	1	1	1	1	1	b1995	orf, hypothetical protein
b1998	0	0	0	0	0	0	0	0	0	0	0	1	0	0	0	0	0	0	0	1	0	0	1	1	1	0	0	0	0	1	0	1	0	0	0	0	0	0	0	0	0	1	0	b1998	orf, hypothetical protein
b1999	0	0	0	0	1	1	0	1	1	0	1	1	0	0	0	1	0	0	0	1	1	1	1	1	1	0	0	0	1	1	1	1	0	0	1	1	0	0	0	0	0	1	0	yeeP	putative histone
b2000	0	0	0	0	0	0	0	0	0	0	0	0	0	0	0	0	0	0	0	0	0	0	0	0	0	0	0	0	0	0	0	0	0	0	1	1	0	0	0	0	0	0	0	flu	outer membrane fluffing protein, similar to adhesin
b2001	0	0	0	0	0	0	0	0	0	0	0	0	0	0	0	0	0	0	0	0	0	0	0	0	0	0	0	0	0	0	0	0	0	0	1	1	0	0	0	0	0	0	0	b2001	orf, hypothetical protein
b2002	1	1	0	0	1	0	1	1	1	1	1	1	0	0	0	0	0	1	1	1	1	0	0	1	1	0	0	0	0	1	1	1	1	0	1	1	0	0	0	0	0	0	0	yeeS	putative DNA repair protein, RADC family
b2003	1	1	0	0	1	1	1	1	1	1	1	1	0	0	0	0	0	1	1	1	1	0	0	1	1	0	0	0	0	1	1	1	1	0	1	1	0	0	0	0	0	0	0	yeeT	orf, hypothetical protein
b2004	1	1	0	0	1	1	1	1	1	1	1	1	0	0	0	0	0	1	1	1	1	0	0	1	1	0	0	0	0	1	1	0	1	0	1	1	0	0	0	0	0	0	0	yeeU	putative structural protein
b2005	1	1	0	0	1	1	1	1	1	1	1	1	0	0	0	0	0	1	1	1	1	0	0	1	1	0	1	0	0	1	1	1	1	0	1	1	0	0	0	0	0	0	0	yeeV	orf, hypothetical protein
b2006	1	1	0	0	1	1	1	1	1	1	1	1	0	0	0	0	0	1	1	1	1	0	0	1	1	0	1	0	0	1	1	1	1	0	1	1	0	0	0	0	0	0	0	yeeW	orf, hypothetical protein
b2007	1	1	1	1	1	0	1	1	1	1	1	1	0	1	1	1	1	1	1	1	1	1	1	1	1	1	1	1	1	1	1	1	1	0	1	1	1	1	1	1	1	1	1	yeeX	putative alpha helix protein
b2008	1	1	1	1	1	0	1	1	1	1	1	1	0	1	1	0	1	1	1	1	1	1	1	1	1	1	1	1	1	1	1	1	0	0	0	0	1	0	0	0	0	1	1	yeeA	orf, hypothetical protein
b2009	1	0	1	1	1	0	1	1	1	1	1	1	0	0	0	0	1	1	1	1	1	1	1	1	1	1	1	1	1	1	1	1	0	0	0	0	0	0	0	0	0	0	0	sbmC	SbmC protein
b2010	1	0	1	1	1	1	1	1	1	1	1	1	0	0	0	0	1	1	1	1	1	1	1	1	1	1	1	1	1	1	1	1	0	0	0	0	0	0	0	0	0	0	0	dacD	penicillin binding protein 6b
b2012	1	0	1	1	0	1	1	1	1	1	1	1	1	1	1	1	1	1	1	1	1	1	1	1	1	1	1	1	1	1	1	1	1	1	1	1	1	1	1	1	1	1	1	yeeD	orf, hypothetical protein
b2013	1	0	1	1	0	1	1	1	1	1	1	1	1	1	1	1	1	1	1	1	1	1	1	1	1	1	1	1	1	1	1	1	1	1	1	1	1	1	1	1	1	1	1	yeeE	putative transport system permease protein
b2015	1	1	1	1	1	1	1	1	1	1	1	1	1	1	1	1	1	1	1	1	1	1	1	1	1	1	0	1	1	1	1	1	1	1	1	1	1	1	1	1	1	1	1	yeeY	putative transcriptional regulator LYSR-type
b2016	1	1	1	1	1	1	1	1	1	1	1	1	1	1	1	1	1	1	1	1	1	0	1	1	1	1	1	1	1	1	1	1	1	1	1	1	1	1	1	1	1	1	1	b2016	putative enzyme of sugar metabolism
b2017	0	0	1	1	1	1	1	1	1	1	1	1	1	1	1	1	1	1	1	1	1	1	1	1	1	0	0	0	0	0	0	1	1	1	1	1	1	1	1	1	1	1	1	yefM	orf, hypothetical protein
b2020	1	1	1	1	1	0	1	1	1	1	1	1	1	1	1	1	1	1	1	0	1	1	1	1	1	1	1	1	1	0	0	1	1	1	1	1	1	1	1	1	1	1	1	hisD	L-histidinal:NAD+ oxidoreductase; L-histidinol:NAD+ oxidoreductase
b2022	1	1	1	1	1	1	1	1	1	1	1	1	1	1	1	1	1	1	1	1	1	1	1	1	1	1	1	1	1	1	1	1	0	1	1	1	1	1	1	1	1	1	1	hisB	imidazoleglycerolphosphate dehydratase and histidinol-phosphate phosphatase
b2024	1	1	1	1	1	0	0	0	1	1	1	1	0	1	1	1	1	1	1	1	1	1	1	1	1	1	1	1	1	1	1	1	0	1	1	1	1	1	1	1	0	1	1	hisA	N-(5'-phospho-L-ribosyl-formimino)-5-amino-1-(5'- phosphoribosyl)-4-imidazolecarboxamide isomerase
b2025	1	1	1	1	1	0	1	1	1	1	1	1	1	1	1	1	1	1	1	1	1	1	1	1	1	1	1	1	1	1	1	1	1	1	1	1	1	1	1	1	1	1	1	hisF	imidazole glycerol phosphate synthase subunit in heterodimer with HisH
b2026	1	1	1	1	1	1	1	1	1	1	1	1	1	1	1	1	1	1	1	1	1	1	1	1	1	0	0	1	1	1	1	1	0	1	1	1	1	1	1	1	1	1	1	hisI	phosphoribosyl-amp cyclohydrolase; phosphoribosyl-ATP pyrophosphatase
b2027	1	1	1	1	1	1	1	1	1	1	1	1	1	1	0	1	1	1	1	0	1	1	1	1	1	1	1	1	1	1	1	1	1	1	1	1	1	1	1	1	1	1	1	wzzB	regulator of length of O-antigen component of lipopolysaccharide chains
b2028	1	1	1	1	1	0	1	1	1	1	1	1	1	1	1	1	1	1	1	0	1	1	1	1	1	1	1	1	1	1	1	1	1	1	1	1	1	1	1	1	1	1	1	ugd	UDP-glucose 6-dehydrogenase
b2029	1	1	1	1	1	1	0	1	1	1	1	1	0	1	1	1	1	1	1	1	1	1	1	1	1	1	0	1	0	0	1	1	0	1	1	1	1	1	1	1	1	1	1	gnd	gluconate-6-phosphate dehydrogenase, decarboxylating
b2029a	0	0	0	0	0	0	0	0	0	0	0	0	0	0	0	0	0	0	0	0	0	0	0	0	0	0	0	0	0	0	0	0	0	0	0	0	0	0	0	0	0	0	0	yefJ	Predicted glycosyltransferase
b2032	0	0	0	0	0	0	0	0	0	0	0	0	0	0	0	0	0	0	0	0	0	0	0	0	0	0	0	0	0	0	0	0	0	0	0	0	0	0	0	0	0	0	0	wbbK	putative glucose transferase
b2033	0	0	0	0	0	0	0	0	0	0	0	0	0	0	0	0	0	0	0	0	0	0	0	0	0	0	0	0	0	0	0	0	0	0	0	0	0	0	0	0	0	0	0	wbbJ	putative O-acetyl transferase
b2034	0	0	0	0	0	0	0	0	0	0	0	0	0	0	0	0	0	0	0	0	0	0	0	0	0	0	0	0	0	0	0	0	0	0	0	0	0	0	0	0	0	0	0	wbbI	putative Galf transferase
b2035	0	0	0	0	0	0	0	0	0	0	0	0	0	0	0	0	0	0	0	0	0	0	0	0	0	0	0	0	0	0	0	0	0	0	0	0	0	0	0	0	0	0	0	wbbH	O-antigen polymerase
b2036	0	0	0	0	0	0	0	0	0	0	0	0	0	0	0	0	0	0	0	0	0	0	0	0	0	0	0	0	0	0	0	0	0	0	0	0	0	0	0	0	0	0	0	glf	UDP-galactopyranose mutase
b2037	0	0	0	0	0	0	0	0	0	0	0	0	0	0	0	0	0	0	0	0	0	0	0	0	0	0	0	0	0	0	0	0	0	0	0	0	0	0	0	0	0	0	0	rfbX	putative O-antigen transporter
b2038	0	0	0	0	0	0	0	0	0	0	0	0	0	0	0	0	0	0	0	0	0	0	0	0	0	0	0	0	0	0	0	0	0	0	0	0	0	0	0	0	0	0	0	rfbC	dTDP-6-deoxy-D-glucose-3,5 epimerase
b2039	1	0	0	0	0	0	0	0	0	1	0	1	0	1	1	0	0	1	1	0	0	0	0	0	0	1	0	1	0	0	0	0	1	1	1	0	1	1	1	1	1	1	1	rfbA	glucose-1-phosphate thymidylyltransferase
b2040	1	0	0	1	0	0	1	0	0	1	0	1	0	1	1	1	0	0	1	0	0	0	0	0	0	1	0	0	0	0	1	0	1	1	1	0	0	1	1	1	1	1	1	rfbD	dTDP-6-deoxy-L-mannose-dehydrogenase
b2041	1	0	0	1	0	0	0	0	0	1	0	1	0	1	1	1	0	1	1	0	0	0	0	0	0	1	0	1	0	0	1	0	1	1	1	1	1	1	1	1	0	1	1	rfbB	dTDP-glucose 4,6 dehydratase
b2042	1	1	0	1	1	1	1	0	1	1	1	1	1	1	1	1	1	1	1	0	1	1	1	1	0	1	1	1	1	1	1	1	1	1	1	1	1	1	1	1	1	1	1	galF	homolog of Salmonella UTP--glucose-1-P uridyltransferase, probably a UDP-gal transferase
b2043	0	0	0	1	1	1	1	1	1	1	1	1	1	1	1	1	1	1	1	0	1	1	1	1	1	1	1	1	0	1	1	1	1	1	1	1	1	1	1	1	1	1	1	wcaM	orf, hypothetical protein
b2044	0	0	0	1	1	0	1	0	1	1	1	1	1	1	1	1	1	1	1	1	1	1	1	1	1	1	0	1	0	1	1	1	1	1	1	1	1	1	1	1	1	1	1	wcaL	putative colanic acid biosynthesis glycosyl transferase
b2045	0	0	0	1	1	1	1	1	1	1	1	1	1	1	1	1	1	0	1	0	1	1	1	1	0	1	1	1	0	1	1	1	1	1	1	1	1	1	1	1	0	1	1	wcaK	putative galactokinase (EC 2.7.1.6).
b2046	0	0	0	1	1	0	0	1	0	1	1	1	1	1	1	1	1	1	1	1	1	1	1	1	0	0	1	1	0	0	1	1	0	1	1	1	1	1	1	1	1	1	1	wzxC	probable export protein
b2047	0	0	0	1	1	1	1	1	1	1	1	1	1	1	0	1	1	1	1	1	1	1	1	1	1	1	1	1	0	1	1	1	1	1	1	1	1	1	1	1	1	1	1	wcaJ	putative colanic acid biosynthsis UDP-glucose lipid carrier transferase
b2048	0	1	1	1	1	1	1	1	1	1	1	1	0	1	1	1	1	1	1	1	1	1	1	1	1	1	1	1	1	1	1	1	1	1	1	1	1	1	1	1	1	1	1	cpsG	phosphomannomutase
b2049	0	1	1	1	1	1	1	1	1	1	1	1	1	1	1	1	1	1	1	1	1	1	1	1	1	1	0	0	1	1	1	1	1	1	1	1	1	1	1	1	1	1	1	cpsB	mannose-1-phosphate guanyltransferase
b2050	0	0	1	1	1	0	1	1	1	1	1	1	1	1	1	1	1	1	1	1	1	1	1	1	1	1	1	1	0	1	1	1	1	1	1	1	0	1	1	1	1	1	1	wcaI	putative colanic biosynthesis glycosyl transferase
b2051	0	0	1	1	1	0	1	1	1	1	1	1	1	1	1	1	1	1	1	1	1	1	1	1	1	1	1	1	0	1	1	1	1	1	1	1	0	1	1	1	1	1	1	wcaH	GDP-mannose mannosyl hydrolase
b2052	0	0	1	1	1	0	1	1	1	1	1	1	1	1	1	1	1	1	1	1	1	1	1	1	1	1	1	1	0	1	1	1	1	1	1	1	1	1	1	1	1	1	1	wcaG	putative nucleotide di-P-sugar epimerase or dehydratase
b2053	0	1	1	1	1	1	1	1	1	1	1	1	1	1	1	1	1	1	1	1	1	1	1	1	1	1	1	1	1	1	1	1	1	1	1	1	1	1	1	1	1	1	1	gmd	GDP-D-mannose dehydratase
b2054	0	0	1	1	1	0	1	0	1	1	1	0	1	1	1	1	1	1	1	1	1	1	1	1	1	1	1	1	0	1	0	1	1	1	1	1	1	1	1	1	1	1	1	wcaF	putative transferase
b2055	0	0	1	1	1	0	1	1	1	1	1	0	1	1	1	1	1	1	1	1	1	1	1	1	1	1	1	1	0	1	1	1	1	1	1	0	0	0	0	0	0	1	1	wcaE	putative colanic acid biosynthesis glycosyl transferase
b2056	0	0	1	1	1	1	1	1	1	1	1	1	1	1	1	1	1	1	1	1	1	1	1	1	1	1	1	1	0	0	1	1	1	1	1	1	1	1	1	1	1	1	1	wcaD	putative colanic acid polymerase
b2057	0	0	1	1	1	1	1	1	1	1	1	1	1	1	1	1	1	1	1	1	1	1	1	1	1	1	1	1	0	0	1	1	1	1	1	1	1	1	1	1	1	1	1	wcaC	putative glycosyl transferase
b2058	0	1	1	1	1	1	1	1	1	1	1	1	1	1	1	1	1	1	1	1	1	1	1	1	1	1	1	1	1	1	1	1	1	1	1	1	1	1	1	1	1	1	1	wcaB	putative transferase

b2059	0	0	1	1	1	1	1	1	1	1	1	1	1	1	1	1	1	1	1	1	1	1	1	1	1	1	1	1	0	0	1	1	1	1	1	1	1	1	1	1	1	1	1	wcaA	putative regulator
b2060	0	0	1	1	1	0	1	1	1	1	1	1	1	1	1	1	1	1	1	1	1	1	1	1	1	1	0	1	0	0	1	1	1	1	1	1	1	1	1	1	1	1	1	b2060	orf, hypothetical protein
b2061	0	0	1	1	1	1	1	1	1	1	1	1	1	1	1	1	1	1	1	1	1	1	1	1	1	1	1	1	0	0	1	0	1	1	1	1	1	1	1	1	1	1	1	wzb	probable protein-tyrosine-phosphatase
b2062	0	0	1	1	1	1	1	1	1	1	1	1	1	1	1	1	1	1	1	1	1	1	1	1	1	1	1	1	0	0	1	1	1	1	1	1	1	1	1	0	1	1	1	wza	putative polysaccharide export protein
b2066	1	1	1	1	1	1	1	1	1	1	1	1	1	1	1	1	1	1	1	1	1	1	1	1	1	1	1	1	0	1	1	1	1	1	1	1	1	1	1	1	1	1	1	udk	uridine/cytidine kinase
b2067	1	1	1	1	0	1	1	1	1	1	1	1	1	1	1	1	1	1	1	1	1	1	1	1	1	1	1	1	1	1	1	1	1	1	1	1	1	1	1	1	1	1	1	yegE	putative sensor-type protein
b2068	1	1	1	1	0	0	1	1	1	1	1	1	1	1	1	1	1	1	1	1	1	1	1	1	1	1	1	1	1	1	1	1	1	1	1	1	1	1	1	1	1	1	1	alkA	3-methyl-adenine DNA glycosylase II, inducible
b2069	1	1	1	1	0	1	1	1	1	1	1	1	1	1	1	1	1	1	1	1	1	1	1	1	1	1	1	1	1	1	1	1	1	1	1	1	1	1	1	1	1	1	1	yegD	putative heat shock protein
b2070	1	0	1	1	0	0	1	1	1	1	1	1	1	1	1	0	1	1	1	1	1	1	1	1	1	1	1	1	0	1	1	1	1	1	1	1	1	1	1	1	1	1	1	b2070	putative chaperonin
b2071	0	0	0	0	0	0	0	0	0	0	0	0	0	0	0	0	1	1	1	1	1	1	1	1	0	0	0	0	1	1	1	1	0	0	0	0	0	0	0	0	0	0	0	b2071	orf, hypothetical protein
b2072	1	0	1	1	1	0	0	0	0	1	1	1	1	1	1	1	1	1	1	1	1	1	1	1	0	0	0	0	1	0	0	1	1	1	1	1	1	1	1	1	1	1	1	b2072	orf, hypothetical protein
b2073	1	0	1	1	1	1	1	1	1	1	1	1	1	1	1	1	1	1	1	1	1	1	1	1	0	0	0	0	1	1	1	1	1	1	1	1	1	1	1	1	1	1	1	b2073	orf, hypothetical protein
b2074	0	1	1	1	1	1	1	1	1	1	1	1	1	1	1	1	1	1	1	1	1	1	1	1	1	1	1	1	1	1	1	1	1	1	1	1	1	1	1	1	0	1	1	b2074	putative membrane protein
b2075	0	1	1	1	1	1	1	1	1	1	1	1	1	1	1	1	1	1	1	1	1	1	1	1	1	1	1	1	1	1	1	1	1	1	1	1	0	1	1	1	1	1	1	yegN	orf, hypothetical protein
b2076	0	1	1	1	1	1	1	1	1	1	1	1	1	1	1	1	1	1	1	1	1	1	1	1	1	1	1	1	1	1	1	1	1	1	1	1	1	1	1	1	1	1	1	yegO	orf, hypothetical protein
b2082	0	0	0	1	1	1	0	1	1	1	1	1	1	0	0	1	0	0	1	1	1	1	1	1	1	0	1	0	0	0	1	1	0	0	0	0	0	0	0	0	0	1	0	ogrK	prophage P2 ogr protein
b2084	1	0	1	1	1	1	0	1	1	1	1	1	0	1	1	1	0	1	1	1	1	1	1	1	1	1	1	1	1	1	0	1	1	1	1	1	1	1	1	1	1	1	1	b2084	orf, hypothetical protein
b2085	1	0	1	1	1	1	1	1	1	1	1	1	0	1	1	1	1	1	1	1	1	1	1	1	1	1	1	1	1	0	1	1	0	1	1	1	1	1	1	1	1	1	1	b2085	orf, hypothetical protein
b2086	1	0	1	1	1	1	1	1	1	1	1	1	1	1	1	1	1	1	1	1	1	1	1	1	1	1	1	1	1	1	1	1	1	1	1	1	1	1	1	1	1	1	1	b2086	orf, hypothetical protein
b2087m	1	0	1	1	1	1	1	1	1	1	1	1	1	1	1	1	1	1	1	1	1	1	1	1	1	1	1	1	1	1	1	1	1	1	1	1	1	1	1	1	1	1	1	gatR	Galactitol utilization operon repressor
b2091	1	0	1	1	1	1	1	1	1	1	1	1	1	1	1	1	1	1	1	1	1	1	1	1	1	1	1	1	1	1	1	1	1	1	1	1	1	1	1	1	1	1	1	gatD	galactitol-1-phosphate dehydrogenase
b2093	1	0	1	1	1	1	1	1	1	1	1	1	1	1	1	1	1	1	1	1	1	1	1	1	1	1	1	1	1	1	1	1	1	1	1	1	1	1	1	1	1	1	1	gatB	galactitol-specific enzyme IIB of phosphotransferase system
b2094	1	0	1	1	1	1	1	1	1	1	1	1	1	1	1	1	1	1	1	1	1	1	1	1	1	1	1	1	1	1	1	1	1	1	1	1	1	1	1	1	1	1	1	gatA	galactitol-specific enzyme IIA of phosphotransferase system
b2095	1	0	1	1	1	1	1	1	1	1	1	1	1	1	1	1	1	1	1	1	1	1	1	1	1	1	1	1	1	1	1	1	1	1	1	1	1	1	1	1	1	1	1	gatZ	putative tagatose 6-phosphate kinase 1
b2096	1	0	1	1	1	1	1	1	1	1	1	1	1	1	1	1	1	1	1	1	1	1	1	1	1	1	1	1	1	1	1	1	1	1	1	1	1	1	1	1	1	1	1	gatY	tagatose-bisphosphate aldolase 1
b2097	1	0	1	1	1	1	1	1	1	1	1	1	1	1	1	1	1	1	1	1	1	1	1	1	1	1	1	1	1	1	1	1	1	1	1	1	1	1	1	1	1	1	1	b2097	orf, hypothetical protein
b2098	1	0	1	1	1	1	1	1	1	1	1	1	1	1	1	1	1	1	1	1	1	1	1	1	1	1	1	1	1	1	1	1	1	1	0	0	1	1	1	1	0	1	1	yegT	putative nucleoside permease protein
b2099	1	0	1	1	1	0	0	1	1	1	1	1	1	1	1	1	1	1	1	1	1	1	1	1	1	1	1	1	1	1	1	1	1	1	1	1	1	1	1	1	1	1	1	b2099	orf, hypothetical protein
b2100	1	0	1	1	1	1	1	1	1	1	1	1	1	1	1	1	1	1	1	1	1	1	1	1	1	1	1	1	1	1	1	1	1	1	1	1	1	1	1	1	1	1	1	b2100	putative kinase
b2101	1	0	1	1	1	1	1	1	1	1	1	1	1	1	1	1	1	1	1	1	1	1	1	1	1	1	1	1	1	1	1	1	1	1	1	1	1	1	1	1	1	1	1	yegW	putative transcriptional regulator
b2105	1	1	1	1	1	1	1	1	1	1	1	1	0	0	1	1	1	1	1	1	1	1	1	1	1	1	1	1	1	1	1	1	1	1	1	1	1	1	1	1	1	1	1	yohL	orf, hypothetical protein
b2106	1	1	1	1	1	1	1	1	1	1	1	1	0	0	0	1	1	1	1	1	1	1	1	1	1	1	1	1	1	1	1	1	1	1	1	1	1	1	1	1	1	1	1	yohM	orf, hypothetical protein
b2107	1	1	1	1	1	1	1	1	1	1	1	1	0	0	0	1	1	1	1	1	1	1	1	1	1	1	1	1	1	1	1	1	1	1	1	1	1	1	1	1	1	1	1	b2107	orf, hypothetical protein
b2108	0	1	1	1	1	0	0	0	0	0	0	0	0	0	0	1	0	0	0	0	0	0	0	0	1	1	1	1	1	1	1	1	0	1	1	1	1	1	1	1	1	1	1	yehA	putative type-1 fimbrial protein
b2109	1	1	1	1	1	0	0	0	0	0	0	0	0	0	0	1	0	1	1	1	1	1	1	1	1	1	1	1	1	1	1	1	0	1	1	1	1	1	1	1	1	1	1	yehB	putative outer membrane protein
b2110	0	1	1	1	0	1	1	1	0	1	1	1	0	0	0	1	0	0	1	1	1	1	1	1	1	1	1	1	1	1	1	1	0	1	1	1	1	1	1	1	1	1	1	yehC	putative chaperone
b2111	0	1	1	1	0	0	0	0	0	0	0	1	0	0	0	1	1	0	0	0	1	0	0	0	1	1	1	1	1	1	1	1	0	1	1	1	1	1	1	1	1	1	1	yehD	putative fimbrial-like protein
b2112	1	1	1	1	1	1	0	1	1	1	1	1	0	0	0	1	1	1	0	1	1	1	1	1	1	1	1	1	1	1	1	1	1	1	1	1	1	1	1	1	1	1	1	yehE	orf, hypothetical protein
b2113	1	1	1	1	1	1	1	1	1	1	1	1	1	1	1	1	1	1	1	1	1	1	1	1	0	1	1	1	1	1	1	1	1	1	1	1	1	1	1	1	1	1	1	mrp	putative ATPase
b2115	0	1	1	1	1	1	1	1	1	1	1	1	0	0	0	1	0	0	1	1	1	1	1	1	1	1	1	1	1	1	1	1	1	1	1	1	1	1	1	1	1	1	1	molR_1	molybdate metabolism regulator, first fragment
b2116	0	0	0	0	0	0	0	0	0	0	0	0	0	1	0	1	0	0	0	0	0	0	0	0	1	1	1	1	1	1	1	1	1	1	1	1	1	1	1	1	1	1	1	molR_2	molybdate metabolism regulator, second fragment 2
b2117	1	1	0	1	0	0	0	0	0	0	0	0	1	1	1	1	0	0	0	0	0	0	0	0	1	1	1	1	1	1	1	1	1	1	1	1	1	1	1	1	0	1	1	molR_3	molybdate metabolism regulator, third fragment
b2118	1	1	0	1	0	0	0	0	0	0	0	0	1	1	1	1	0	0	0	0	0	0	0	0	1	1	1	1	1	1	1	1	1	1	1	1	1	1	1	1	1	1	1	yehI	putative regulator
b2118a	0	1	0	1	0	0	0	0	0	0	0	0	1	1	1	1	0	0	0	0	0	0	0	0	1	1	1	1	1	1	1	1	1	0	1	1	1	1	1	1	1	1	1	yehK	Uncharacterized conserved protein
b2119	0	1	0	1	1	1	1	1	1	1	1	1	1	1	1	1	1	1	1	1	1	1	1	1	1	1	1	1	1	1	1	1	1	0	1	1	1	1	1	1	1	1	1	yehL	orf, hypothetical protein
b2120	0	1	1	1	1	0	0	0	0	0	0	0	1	1	1	1	1	0	0	0	0	0	0	0	1	1	1	1	1	1	1	0	1	0	1	1	1	1	1	1	1	1	1	yehM	orf, hypothetical protein
b2121	1	1	0	1	1	0	0	0	0	0	0	0	1	1	1	1	1	0	0	0	0	0	0	0	1	1	1	1	1	1	1	0	1	0	1	1	1	1	1	1	1	1	1	yehP	orf, hypothetical protein
b2122	1	1	1	1	1	0	0	0	0	0	0	0	0	1	1	1	1	0	0	0	0	0	0	0	0	1	0	1	1	1	1	0	0	0	1	1	1	1	1	1	1	1	1	yehQ	orf, hypothetical protein
b2123	1	1	1	1	1	1	1	1	1	1	1	1	1	1	1	1	1	1	1	1	1	1	1	1	1	1	1	1	1	1	1	1	1	1	1	0	1	1	1	1	1	1	1	yehR	orf, hypothetical protein
b2127	1	1	1	0	1	0	1	1	0	1	1	1	1	1	1	1	1	1	1	1	1	1	0	1	1	1	1	1	1	1	1	1	1	1	1	1	1	1	1	1	1	0	0	yehV	putative transcriptional regulator
b2128	1	1	1	0	1	1	1	1	1	1	1	1	1	1	1	1	1	1	1	1	1	1	0	1	1	1	1	1	1	1	1	1	1	1	1	1	1	1	1	1	1	1	1	yehW	putative transport system permease protein
b2129	1	1	1	0	1	1	1	1	1	1	1	1	1	1	1	1	1	1	1	1	1	1	0	1	1	1	1	1	1	1	1	1	1	1	1	1	1	1	1	1	1	1	1	yehX	putative ATP-binding component of a transport system
b2130	1	1	1	0	1	1	1	1	1	1	1	1	1	1	1	1	1	1	1	1	1	1	0	1	1	1	1	1	1	1	1	1	1	1	1	1	1	1	1	1	1	1	1	yehY	putative transport system permease protein
b2131	1	1	1	0	1	1	1	1	1	1	1	1	1	1	1	1	1	1	1	1	1	1	0	1	1	0	0	1	1	1	1	1	1	1	1	1	1	1	1	1	1	1	1	yehZ	putative transport system permease protein
b2132	1	1	1	1	1	1	1	1	1	1	1	1	1	1	1	1	1	1	1	1	1	1	0	1	1	1	1	1	1	1	1	1	1	1	1	1	1	1	1	1	1	1	1	bglX	beta-D-glucoside glucohydrolase, periplasmic
b2133	1	1	1	1	1	0	0	1	0	1	1	1	0	1	1	1	1	1	1	1	1	1	1	1	1	1	1	1	1	1	1	1	0	1	1	1	1	1	1	1	1	1	1	dld	D-lactate dehydrogenase, FAD protein, NADH independent
b2134	1	1	1	1	1	1	1	1	1	1	1	1	1	1	1	1	1	1	1	1	1	1	0	1	1	1	1	1	1	1	1	1	1	1	1	1	1	1	1	1	1	1	1	pbpG	penicillin-binding protein 7
b2135	1	1	1	1	1	1	1	1	1	1	1	1	1	1	1	1	1	1	1	1	1	1	0	1	1	1	1	1	1	1	1	1	1	1	1	1	1	1	1	1	1	1	1	yohC	orf, hypothetical protein
b2136	1	1	1	1	1	1	1	1	1	1	1	1	1	1	1	1	1	1	1	1	1	1	0	1	1	1	1	1	1	1	1	1	1	1	1	1	1	1	1	1	1	1	1	yohD	orf, hypothetical protein
b2137	1	0	1	1	1	1	1	1	1	1	1	1	1	1	1	1	1	1	1	1	1	1	0	1	1	1	1	1	1	1	1	1	1	1	1	1	1	1	1	1	1	1	1	yohF	putative oxidoreductase
b2137a	1	0	1	1	1	1	1	1	1	1	1	1	1	1	1	1	1	1	1	1	1	1	0	1	1	1	1	1	1	1	1	0	1	1	1	1	1	1	1	1	1	1	1	yohE	Predicted dehydrogenase
b2138	1	1	1	1	1	1	1	1	1	1	1	1	1	1	1	1	1	1	1	1	1	1	0	1	1	1	1	1	1	1	1	0	1	1	1	1	1	1	1	1	1	1	1	yohG	putative channel/filament proteins
b2139	1	1	1	1	1	1	1	1	1	1	1	1	0	1	1	1	0	1	1	1	1	1	0	1	1	1	1	1	1	1	1	0	1	1	1	1	1	1	1	1	1	1	1	yohH	orf, hypothetical protein
b2140	1	1	1	1	1	1	1	1	1	1	1	1	1	1	1	1	0	1	1	1	1	1	0	1	1	1	1	1	1	1	1	1	1	1	1	1	1	1	1	1	1	1	1	yohI	putative regulator protein
b2141	1	1	1	1	1	1	1	1	1	1	1	1	1	1	1	1	0	1	1	1	1	1	0	1	1	1	1	1	1	1	1	0	1	1	1	1	1	1	1	1	1	1	1	yohJ	orf, hypothetical protein
b2142	1	1	1	1	1	1	1	1	1	1	1	1	1	1	1	1	0	1	1	1	1	1	0	1	1	1	1	1	1	1	1	1	1	1	1	1	1	1	1	1	1	1	1	yohK	putative seritonin transporter
b2143	1	1	1	1	1	1	1	1	1	1	1	1	1	1	1	1	0	1	1	1	1	1	0	1	1	1	1	1	1	1	1	1	1	1	1	1	1	1	1	1	1	1	1	cdd	cytidine/deoxycytidine deaminase
b2144	1	1	1	1	1	1	1	1	1	1	1	1	1	1	1	1	1	1	1	1	1	1	0	1	1	1	1	1	1	1	1	1	1	1	1	1	1	1	1	1	1	1	1	sanA	vancomycin sensitivity
b2145	1	1	1	1	1	1	1	1	1	1	1	1	1	1	1	1	1	1	1	1	1	1	0	1	1	1	1	1	1	1	1	1	1	1	1	1	1	1	1	1	1	1	1	b2145	orf, hypothetical protein
b2146	1	1	1	1	1	1	1	1	1	1	1	1	1	1	1	1	1	1	1	1	1	1	0	1	1	1	1	1	1	1	1	1	1	1	1	1	1	1	1	1	1	1	1	b2146	putative oxidoreductase
b2151	1	1	1	1	1	1	1	1	1	1	1	1	1	1	1	1	1	1	1	1	1	1	1	1	1	1	1	1	0	1	1	1	1	1	1	1	1	1	1	1	1	1	1	galS	mgl repressor, galactose operon inducer
b2154	1	1	1	1	1	1	1	1	1	1	1	1	1	1	1	1	0	1	1	1	1	1	1	1	1	1	0	1	0	1	1	1	1	1	1	1	1	1	1	1	1	1	1	yeiG	putative esterase (EC 3.1.1.-).
b2160	1	1	1	1	0	1	1	1	1	1	1	1	1	1	1	1	1	1	1	1	1	1	1	1	1	1	1	1	1	1	1	1	1	1	1	1	1	1	1	1	1	1	1	yeiI	putative kinase
b2162	1	0	1	1	1	1	1	1	1	1	1	1	1	1	1	1	1	1	1	1	1	1	1	1	1	1	1	1	1	1	1	1	1	1	1	1	1	1	1	1	1	1	1	yeiK	orf, hypothetical protein
b2163	1	0	1	1	1	0	0	0	0	0	0	0	0	0	0	0	0	0	0	0	0	0	0	0	1	1	1	1	1	1	1	1	1	1	1	1	1	1	1	1	1	1	1	yeiL	putative transcriptional regulator
b2165	0	1	1	1	1	1	1	1	1	1	1	1	1	1	1	1	1	1	1	1	1	1	1	1	1	1	1	1	1	1	1	1	1	1	1	1	1	1	1	1	1	1	1	yeiN	orf, hypothetical protein
b2166	0	1	1	1	1	0	1	1	1	1	1	1	1	1	1	1	1	0	0	0	0	0	0	0	1	1	1	1	1	1	1	1	1	1	1	1	1	1	1	1	1	1	1	yeiC	putative kinase
b2169	1	1	1	1	1	1	1	1	1	1	1	1	1	1	1	1	1	1	1	1	1	1	1	1	1	1	1	1	1	1	1	1	1	1	1	1	0	1	1	1	1	1	1	fruB	PTS system, fructose-specific IIA/fpr component
b2170	1	1	1	1	1	1	1	1	1	1	1	1	1	1	1	1	1	1	1	1	1	1	1	1	1	1	1	1	1	1	1	0	1	1	1	1	1	1	1	1	1	1	1	yeiO	putative transport
b2172	1	1	1	1	1	0	1	1	1	1	1	1	0	1	1	1	1	1	1	1	1	1	1	1	1	1	1	1	1	1	1	1	1	1	1	1	1	1	1	1	1	1	1	yeiQ	putative oxidoreductase
b2178	1	1	1	1	1	1	1	1	1	1	1	1	1	1	1	1	1	1	1	1	1	1	1	1	1	1	1	1	1	1	1	1	1	1	1	1	0	1	1	1	1	1	1	yejB	putative transport system permease protein
b2180	1	1	1	1	1	1	1	1	1	1	1	1	1	1	1	1	1	1	1	1	1	1	1	1	1	1	1	1	1	0	1	1	1	1	1	1	1	1	1	1	1	1	1	yejF	putative ATP-binding component of a transport system
b2184	1	1	1	1	1	1	1	1	1	1	1	1	1	1	1	1	1	1	1	1	1	1	1	1	1	1	1	1	1	1	1	1	1	1	1	1	0	1	1	1	1	1	1	yejH	putative ATP-dependent helicase
b2190	1	1	1	1	1	0	1	0	0	1	1	1	1	1	1	1	0	1	1	1	1	1	1	1	1	1	1	1	1	1	1	1	1	1	1	1	1	1	1	1	1	1	1	yejO	putative ATP-binding component of a transport system
b2215	1	1	1	1	1	1	0	0	0	1	1	1	0	1	1	0	1	1	1	1	1	1	1	1	1	1	1	1	1	1	1	1	1	1	1	1	1	1	1	1	1	1	1	ompC	outer membrane protein 1b (Ib;c)
b2219	0	1	1	1	0	0	0	0	0	0	0	0	0	0	0	0	0	0	0	0	0	0	0	0	1	1	1	1	1	1	1	1	0	0	0	0	0	0	0	0	0	0	0	atoS	sensor protein AtoS for response regulator AtoC
b2220	0	1	1	1	0	0	0	0	0	0	0	0	0	0	0	0	0	0	0	0	0	0	0	0	1	0	1	1	1	1	1	1	0	0	0	0	0	0	0	0	0	0	0	atoC	response regulator of ato, ornithine decarboxylase antizyme (sensor ATOS)
b2221	0	1	1	1	0	0	0	0	0	0	0	0	0	0	0	0	0	0	0	0	0	0	0	0	1	0	1	1	1	1	1	1	0	0	0	0	0	0	0	0	0	0	0	atoD	acetyl-CoA:acetoacetyl-CoA transferase alpha subunit
b2222	0	1	1	1	0	0	0	0	0	0	0	0	0	0	0	0	0	0	0	0	0	0	0	0	1	0	1	1	1	1	1	1	0	0	0	0	0	0	0	0	0	0	0	atoA	acetyl-CoA:acetoacetyl-CoA transferase beta subunit
b2223	0	1	1	1	0	0	0	0	0	0	0	0	0	0	0	0	0	0	0	0	0	0	0	0	1	0	1	1	1	1	1	1	0	0	0	0	0	0	0	0	0	0	0	atoE	short chain fatty acid transporter
b2224	0	1	1	1	0	0	0	0	0	0	0	0	0	0	0	0	0	0	0	0	0	0	0	0	1	0	1	1	1	1	1	1	0	0	0	0	0	0	0	0	0	0	0	atoB	acetyl-CoA acetyltransferase
b2225	1	1	1	1	1	0	0	0	1	0	1	0	1	1	1	1	1	1	1	1	1	1	1	1	1	0	1	1	1	1	1	1	1	1	1	1	1	1	1	1	1	1	1	b2225	orf, hypothetical protein
b2226	1	1	1	1	1	0	0	0	1	0	1	0	1	1	1	1	1	1	1	1	1	1	1	1	1	0	1	1	1	1	1	1	1	1	1	1	1	1	1	1	1	1	1	b2226	orf, hypothetical protein
b2228m	1	1	0	1	1	1	1	1	1	0	1	1	1	1	1	1	1	1	1	1	1	1	1	1	1	0	1	1	1	1	1	1	1	1	1	1	1	1	1	1	1	1	1	yfaSR	Large extracellular alpha-helical protein
b2229	1	1	0	0	1	0	0	0	0	0	0	0	0	0	0	0	0	0	0	0	0	0	0	0	1	1	1	1	1	1	1	1	1	1	1	1	1	1	1	1	1	1	1	b2229	orf, hypothetical protein
b2230	0	1	1	1	1	1	0	0	0	0	1	1	1	1	0	0	1	0	0	0	1	0	1	0	1	1	1	1	1	1	1	1	1	1	1	1	0	1	1	1	1	1	1	yfaA	orf, hypothetical protein
b2232	1	1	1	1	1	1	1	1	1	1	1	1	1	1	1	1	1	1	1	1	1	1	1	1	1	1	1	1	1	1	1	1	1	1	1	1	0	1	1	1	1	1	1	ubiG	3-demethylubiquinone-9 3-methyltransferase and 2-octaprenyl-6-hydroxy phenol methylase
b2233	1	1	1	1	1	1	1	1	1	1	1	1	0	1	1	0	1	1	1	1	1	1	1	1	0	0	1	1	0	1	0	1	0	1	1	1	1	1	1	1	1	1	1	yfaL	putative ATP-binding component of a transport system
b2237	1	0	1	1	1	1	1	1	1	1	1	1	1	1	1	1	1	1	1	1	1	1	1	1	1	1	1	1	1	1	1	1	1	1	1	1	1	1	1	1	1	1	1	inaA	pH-inducible protein involved in stress response
b2238	0	0	1	1	1	0	1	1	1	1	1	1	1	1	1	1	1	1	1	1	1	1	1	1	1	1	1	1	1	0	0	1	1	1	1	1	1	1	1	1	1	1	1	yfaH	orf, hypothetical protein
b2239	1	1	1	1	1	0	1	1	1	1	1	1	1	1	1	1	1	1	1	1	1	1	1	1	1	1	1	1	1	1	1	1	1	1	1	1	1	1	1	1	1	1	1	glpQ	glycerophosphodiester phosphodiesterase, periplasmic
b2240	1	1	1	1	1	1	1	1	1	1	1	1	1	1	1	1	1	1	1	1	1	1	1	1	1	1	1	1	1	1	1	1	1	1	1	1	0	1	1	1	1	1	1	glpT	sn-glycerol-3-phosphate permease
b2241	1	1	1	1	1	0	1	1	1	1	1	1	1	1	1	1	1	1	1	1	1	1	1	1	1	1	1	1	1	1	1	1	1	1	1	1	1	1	1	1	1	1	1	glpA	sn-glycerol-3-phosphate dehydrogenase (anaerobic), large subunit
b2242	1	1	1	1	1	0	1	1	1	1	1	1	1	1	1	1	1	1	1	1	1	1	1	1	1	1	1	1	1	1	1	1	1	1	1	1	1	1	1	1	1	1	1	glpB	sn-glycerol-3-phosphate dehydrogenase (anaerobic), membrane anchor subunit
b2244	0	1	1	1	1	0	0	1	1	1	1	1	1	1	1	1	1	1	1	1	1	1	1	1	1	1	0	1	1	1	1	1	0	0	0	1	1	1	1	1	1	1	1	yfaD	orf, hypothetical protein
b2245	1	1	1	1	1	0	1	1	1	1	1	1	1	1	1	1	1	1	1	1	1	1	1	1	1	1	1	1	1	1	1	1	1	1	1	1	1	1	1	1	1	1	1	b2245	orf, hypothetical protein

b2246	1	1	1	1	0	0	0	0	1	0	1	0	1	1	1	1	1	1	1	1	1	1	1	1	1	1	1	1	1	1	1	1	0	0	0	0	0	0	0	0	0	0	0	b2246	putative transport protein
b2247	1	1	1	1	0	0	0	0	1	0	1	0	1	1	1	1	1	1	1	1	1	1	1	1	1	1	1	1	1	1	1	1	0	0	0	0	0	0	0	0	0	0	0	b2247	putative racemase
b2248	1	1	1	1	0	0	0	0	1	0	1	0	1	1	1	1	1	1	1	1	1	1	1	1	1	1	1	1	1	1	1	1	0	0	0	0	0	0	0	0	0	0	0	b2248	putative regulator
b2249	0	1	1	1	1	1	0	1	1	0	1	1	1	0	1	1	1	1	1	1	1	1	1	1	1	1	1	1	1	1	1	1	1	1	1	1	0	1	1	1	1	1	1	b2249	orf, hypothetical protein
b2250	0	1	1	1	1	0	0	1	1	1	1	1	1	0	1	1	1	1	1	1	1	1	1	1	1	1	1	1	1	1	1	1	1	1	1	1	1	1	1	1	1	1	1	b2250	orf, hypothetical protein
b2251	0	1	1	1	1	0	0	0	1	0	1	0	1	0	1	1	1	1	1	1	1	1	1	1	1	1	1	1	1	1	1	1	1	1	1	1	1	1	1	1	1	1	1	yfaO	orf, hypothetical protein
b2252	0	1	1	1	1	0	0	0	0	0	0	0	1	1	1	1	1	1	1	1	1	1	1	1	1	1	1	1	1	1	1	1	1	1	1	1	0	1	1	1	1	1	1	ais	protein induced by aluminum
b2253	1	1	1	1	1	0	0	0	0	0	0	0	1	1	1	1	1	1	1	1	1	1	1	1	1	1	0	1	1	1	1	1	1	1	1	1	1	1	1	1	1	1	1	b2253	putative enzyme
b2254	1	1	1	1	1	0	0	0	0	0	0	0	1	1	1	1	1	1	1	1	1	1	1	1	1	1	0	1	1	1	1	1	1	1	1	1	1	1	1	1	1	1	1	b2254	putative sugar transferase
b2255	1	1	1	1	1	0	1	1	1	1	1	1	1	1	1	1	1	1	1	1	1	1	1	1	1	1	0	1	1	1	1	1	1	1	1	1	0	1	1	1	1	1	1	b2255	putative transformylase
b2256	1	1	1	1	0	0	1	1	1	1	1	1	1	1	1	1	1	1	1	1	1	1	1	1	1	1	1	1	1	1	1	1	1	1	1	0	0	0	1	1	1	1	1	b2256	orf, hypothetical protein
b2257	1	1	1	1	1	0	1	1	1	1	1	1	1	1	1	1	1	1	1	1	1	1	1	1	1	1	1	1	1	1	1	1	1	1	1	1	1	1	1	1	1	1	1	b2257	orf, hypothetical protein
b2258	1	1	1	1	1	0	1	1	1	1	1	1	1	1	1	1	1	1	1	1	1	1	1	1	1	1	1	1	1	1	1	1	1	1	1	1	0	1	1	1	1	1	1	b2258	putative transport/receptor protein
b2259	1	1	1	1	1	0	0	0	0	1	0	0	1	1	1	1	1	1	1	1	1	1	1	1	1	1	1	1	1	1	1	1	1	1	1	1	1	1	1	1	1	1	1	pmrD	polymyxin resistance protein B
b2261	1	1	1	1	1	0	0	0	0	1	1	1	0	1	1	1	1	1	1	1	1	1	1	1	1	1	1	1	1	1	1	1	1	1	1	1	1	1	1	1	1	1	1	menC	o-succinylbenzoyl-CoA synthase; conversion of chorismate to 2-o-succinylbenzoyl-CoA
b2262	1	1	1	1	1	0	1	1	1	1	1	1	1	1	1	1	1	1	1	1	1	1	1	1	1	1	1	1	1	1	1	1	1	1	1	1	0	1	1	1	1	1	1	menB	dihydroxynaphtoic acid synthetase
b2264	1	1	1	1	1	0	1	1	1	1	1	1	1	1	1	1	1	1	1	1	1	1	1	1	1	1	1	1	1	1	0	1	1	1	1	1	0	1	1	1	1	1	1	menD	2-oxoglutarate decarboxylase; SHCHC synthase
b2266	1	1	1	1	1	0	0	0	0	1	1	1	0	1	1	0	1	1	1	1	1	1	1	1	1	1	1	1	1	1	1	1	1	1	1	1	1	1	1	1	1	1	1	elaB	orf, hypothetical protein
b2268	1	0	1	0	0	0	0	0	0	0	0	0	0	1	0	0	0	0	0	0	0	0	0	0	0	1	1	1	1	1	1	1	0	1	1	0	0	0	0	0	0	0	0	elaC	orf, hypothetical protein
b2269	1	0	1	1	1	1	1	1	1	1	1	1	0	0	0	0	1	1	1	1	1	1	1	1	1	1	1	1	1	0	1	1	1	1	1	0	0	0	0	0	1	1	1	elaD	putative sulfatase / phosphatase
b2270	0	1	0	0	0	0	0	0	0	0	0	0	0	0	0	0	0	0	0	0	0	0	0	0	1	1	1	1	1	1	1	1	0	0	0	0	0	0	0	0	0	0	0	yfbK	orf, hypothetical protein
b2271	1	1	0	1	1	0	1	1	1	1	1	1	0	1	1	1	1	1	1	1	1	1	1	1	1	1	1	1	1	1	1	1	1	0	1	1	1	1	1	1	1	0	0	yfbL	putative aminopeptidase
b2272	1	1	1	1	1	1	1	1	1	1	1	1	0	1	1	1	1	1	1	1	1	1	1	1	1	1	1	1	1	1	1	1	1	1	1	1	1	1	1	1	1	1	1	yfbM	orf, hypothetical protein
b2273	0	0	0	0	0	0	0	0	0	0	0	0	0	0	0	0	0	0	0	0	0	0	0	0	0	0	0	0	0	0	0	0	0	0	0	0	0	0	0	0	0	0	0	yfbN	orf, hypothetical protein
b2274	0	0	0	0	0	0	0	0	0	0	0	0	0	0	0	0	0	0	0	0	0	0	0	0	0	0	0	0	0	0	0	0	0	0	0	0	0	0	0	0	0	0	0	b2274	orf, hypothetical protein
b2275	0	0	0	0	0	0	0	0	0	0	0	0	0	0	0	0	0	0	0	0	0	0	0	0	0	0	0	0	0	0	0	0	0	0	0	0	0	0	0	0	0	0	0	b2275	orf, hypothetical protein
b2276	1	1	1	1	1	1	1	1	1	1	1	1	1	1	1	1	1	1	1	1	1	1	1	1	1	1	1	1	1	1	1	1	1	1	1	1	0	1	1	1	1	1	1	nuoN	NADH dehydrogenase I chain N
b2284	1	1	1	1	1	0	1	1	1	1	1	1	1	1	1	1	1	1	1	1	1	1	1	1	1	1	1	1	1	1	1	1	1	1	1	1	0	1	1	1	1	1	1	nuoF	NADH dehydrogenase I chain F
b2291	1	1	1	1	1	1	1	1	1	1	1	1	1	1	1	1	1	1	1	1	1	1	1	1	1	1	1	1	0	1	1	1	1	1	1	1	1	1	1	1	1	1	1	b2291	putative alpha helix protein
b2292	1	1	1	1	1	1	1	1	1	1	1	1	1	1	1	1	1	1	1	1	1	1	1	1	1	1	1	1	0	1	1	1	1	1	1	1	1	1	1	1	1	1	1	yfbS	putative transport protein
b2293	1	1	1	1	1	1	1	1	1	1	1	1	1	1	1	1	1	1	1	1	1	1	1	1	1	1	1	1	0	1	1	1	1	1	1	1	1	1	1	1	1	1	1	yfbT	putative phosphatase
b2294	1	1	1	1	1	1	1	1	1	1	1	1	1	1	1	1	1	1	1	1	1	1	1	1	1	1	1	1	0	1	1	1	1	1	1	1	1	1	1	1	1	1	1	b2294	orf, hypothetical protein
b2295	1	1	1	1	1	1	1	1	1	1	1	1	1	1	1	1	1	1	1	1	1	1	1	1	1	1	1	1	0	1	1	1	1	1	1	0	1	1	1	1	1	1	1	b2295	orf, hypothetical protein
b2296	1	1	1	1	1	1	1	1	1	1	1	1	1	1	1	1	1	1	1	1	1	1	1	1	1	1	1	1	0	1	1	1	1	1	1	1	1	1	1	1	1	1	1	ackA	acetate kinase
b2297	1	1	1	1	1	1	1	1	1	1	1	1	0	1	1	1	1	1	1	1	1	1	1	1	1	1	1	1	1	1	1	0	1	1	1	1	1	1	1	1	1	1	1	pta	phosphotransacetylase
b2298	1	1	1	1	1	1	1	1	1	1	1	1	1	1	1	1	1	1	1	1	1	1	1	1	1	1	1	1	1	1	1	1	1	1	1	0	1	1	1	1	1	1	1	yfcC	putative S-transferase
b2299	1	1	1	1	1	0	1	1	1	1	1	1	1	1	1	1	1	1	1	1	1	1	1	1	1	1	1	1	1	1	1	1	1	1	1	0	1	1	1	1	1	1	1	b2299	putative regulator
b2300	1	1	1	1	1	1	1	1	1	1	1	1	1	1	1	1	1	1	1	1	1	1	1	1	1	1	1	1	1	1	1	1	1	1	1	0	1	1	1	1	1	1	1	yfcE	orf, hypothetical protein
b2301	1	0	1	1	1	1	1	1	1	1	1	1	1	1	1	1	1	1	1	1	1	1	1	1	1	1	1	1	1	1	1	1	1	1	1	1	1	1	1	1	1	1	1	yfcF	orf, hypothetical protein
b2305	1	1	1	1	0	1	1	1	1	1	1	1	1	1	1	1	1	1	1	1	1	1	1	1	1	1	1	1	1	1	1	1	1	1	1	1	1	1	1	1	1	1	1	yfcI	orf, hypothetical protein
b2308	1	1	1	1	1	1	1	1	1	1	1	1	1	1	1	1	1	1	1	1	1	1	1	1	1	1	1	1	1	1	1	1	1	1	1	1	0	1	1	1	1	1	1	hisQ	histidine transport system permease protein
b2309	1	1	1	1	1	1	1	1	1	1	1	1	1	1	1	1	1	1	1	1	1	1	1	1	1	1	1	1	1	1	1	1	1	1	1	0	1	1	1	1	1	1	1	hisJ	histidine-binding periplasmic protein of high-affinity histidine transport system
b2311	1	1	1	1	1	1	1	1	1	1	1	1	1	1	1	1	1	1	1	1	1	1	1	1	1	1	1	1	1	1	1	1	1	1	1	1	0	1	1	1	1	1	1	ubiX	3-octaprenyl-4-hydroxybenzoate carboxy-lyase
b2315	1	1	1	1	1	1	1	1	1	1	1	1	1	1	1	1	1	1	1	1	1	1	1	1	1	1	1	1	1	1	1	1	1	1	1	1	0	1	1	1	1	1	1	folC	dihydrofolate:folylpolyglutamate synthetase; dihydrofolate synthetase
b2322	0	1	1	1	1	1	1	1	1	1	1	1	1	1	1	1	1	1	1	1	1	1	1	1	1	1	1	1	1	1	1	1	1	1	1	0	1	1	1	1	1	1	1	b2322	putative transport protein
b2324	1	1	1	1	1	1	1	1	1	1	1	1	1	1	1	1	1	1	1	1	1	1	1	1	1	1	1	1	1	0	0	1	1	1	1	0	0	1	1	1	1	1	1	b2324	putative peptidase
b2326	1	1	1	1	1	1	1	1	1	1	1	1	1	1	1	1	1	1	1	1	1	1	1	1	1	1	1	1	1	1	1	1	1	1	1	0	1	1	1	1	1	1	1	b2326	putative transporting ATPase
b2328	1	1	1	1	1	0	1	1	1	1	1	1	1	1	1	1	1	1	1	1	1	1	1	1	1	1	1	1	1	1	1	1	1	1	1	1	1	1	1	1	1	1	1	mepA	murein DD-endopeptidase, penicillin-insensitive
b2331	1	1	1	1	1	1	1	1	1	1	1	1	1	1	1	1	1	1	1	1	1	1	1	1	1	1	1	1	1	1	1	1	1	1	1	1	1	1	1	0	1	1	1	b2331	orf, hypothetical protein
b2332	0	0	0	0	0	0	0	0	0	0	0	0	0	0	0	0	0	0	0	0	0	0	0	0	0	0	0	0	0	0	0	0	0	0	0	0	0	0	0	0	0	0	0	b2332	orf, hypothetical protein
b2333	0	0	0	0	0	0	0	0	0	0	0	0	0	0	0	0	0	0	0	0	1	1	1	1	0	0	0	0	0	0	0	0	0	0	0	0	0	0	0	0	0	0	0	b2333	putative fimbrial-like protein
b2334	0	0	0	0	0	0	0	0	0	0	0	0	0	0	0	0	0	0	0	0	0	0	0	0	0	0	0	0	0	0	0	0	0	0	0	0	0	0	0	0	0	0	0	b2334	orf, hypothetical protein
b2335	0	0	0	0	0	0	0	0	0	0	0	0	0	1	0	0	0	0	0	0	1	0	0	0	0	0	0	0	0	0	0	0	0	0	0	0	0	0	0	0	0	0	0	b2335	putative fimbrial protein
b2336	0	1	1	1	1	1	1	1	1	1	1	1	1	1	1	0	0	1	1	1	1	1	1	1	1	1	0	1	1	1	1	1	1	1	1	0	0	1	1	1	1	1	1	yfcS	putative chaperone
b2338m	0	1	1	1	1	1	1	1	1	1	1	1	1	1	1	1	1	1	1	1	1	1	1	1	1	1	1	1	1	1	1	1	1	1	1	1	1	1	1	1	1	1	1	yfcU	PapC-like porin protein involved in fimbrial biogenesis
b2339	1	0	1	0	1	1	1	1	1	1	1	1	1	1	1	1	0	1	1	1	0	1	0	1	1	1	1	1	1	1	1	1	1	1	1	0	1	1	1	1	1	1	1	b2339	putative fimbrial-like protein
b2340	1	1	1	1	1	0	1	1	1	1	0	0	1	1	1	1	1	1	1	1	1	1	1	1	1	1	1	1	1	1	1	1	1	1	1	1	1	1	1	1	1	1	1	b2340	orf, hypothetical protein
b2342	1	1	1	1	1	1	1	1	1	1	1	1	1	1	1	1	1	1	1	1	1	1	1	1	1	1	1	1	1	1	1	1	1	1	1	1	0	1	1	1	1	1	1	b2342	putative acyltransferase
b2344	1	1	1	0	1	1	1	1	1	1	1	1	1	1	1	1	1	1	1	1	1	1	1	1	1	1	1	1	1	1	1	1	1	1	1	0	1	1	1	1	1	1	1	fadL	transport of long-chain fatty acids; sensitivity to phage T2
b2345	1	0	1	1	1	1	1	1	1	1	1	1	1	1	1	1	0	1	1	1	1	0	1	1	1	1	1	1	1	1	1	1	1	1	1	0	1	1	1	1	1	1	1	b2345	orf, hypothetical protein
b2347	1	0	1	1	1	1	1	1	1	1	1	1	1	1	1	1	1	1	1	1	1	1	1	1	1	1	1	1	1	1	1	1	1	1	1	1	1	1	1	1	1	1	1	yfdC	putative transport
b2349	0	0	1	0	1	0	0	0	0	0	0	0	0	0	0	0	0	0	0	0	0	0	0	0	1	1	1	1	1	1	0	1	1	0	0	0	0	0	0	0	0	0	0	intC	putative prophage Sf6-like integrase
b2350	0	0	0	0	0	0	0	0	0	0	0	0	0	0	0	0	0	0	0	0	0	0	0	0	0	0	0	0	0	1	0	1	0	1	1	1	1	1	1	1	1	1	1	b2350	orf, hypothetical protein
b2351	0	0	0	0	0	0	0	0	0	0	0	0	0	0	0	0	0	0	0	0	0	0	0	0	0	0	0	0	0	0	0	0	0	0	0	0	0	0	0	0	0	0	0	b2351	putative glycan biosynthesis enzyme
b2352	0	0	0	0	0	0	0	0	0	0	0	0	0	0	0	0	0	0	0	0	0	0	0	0	0	0	0	0	0	0	0	0	0	0	0	0	0	0	0	0	0	0	0	b2352	putative ligase
b2353	0	0	0	0	0	0	0	0	0	0	0	0	0	1	0	1	0	0	0	0	0	0	0	0	0	0	0	0	0	0	0	0	0	0	0	0	0	0	0	0	0	0	0	b2353	orf, hypothetical protein
b2354	0	0	0	0	0	0	0	0	0	0	0	0	0	1	1	1	1	0	0	0	0	0	0	0	0	0	0	0	0	0	0	0	0	0	0	0	0	0	0	0	0	0	0	b2354	orf, hypothetical protein
b2355	0	0	0	0	0	0	0	0	0	0	0	0	0	0	0	0	0	0	0	0	0	0	0	0	0	0	0	0	0	0	0	0	0	1	1	0	0	0	0	0	0	0	0	yfdL	putative RNA polymerase beta
b2356	0	0	0	0	1	1	1	1	1	1	1	1	1	0	0	0	0	1	1	1	1	1	1	1	0	0	0	0	0	0	0	0	0	0	0	0	0	0	0	0	0	1	1	yfdM	orf, hypothetical protein
b2357	0	0	0	0	0	1	1	1	1	1	1	1	1	0	0	0	0	1	1	1	1	1	1	1	0	0	0	1	0	0	0	0	0	0	0	0	0	0	0	0	0	1	1	yfdN	orf, hypothetical protein
b2358	0	0	0	0	1	1	0	1	1	1	1	1	1	0	0	0	0	1	1	1	1	1	1	1	0	0	0	1	0	0	0	0	0	0	0	0	0	0	0	0	0	1	0	yfdO	orf, hypothetical protein
b2359	0	0	0	0	1	0	0	1	1	1	1	0	0	0	0	0	0	0	0	0	0	1	0	0	0	0	0	1	0	0	0	0	0	0	0	0	0	0	0	0	0	0	0	b2359	orf, hypothetical protein
b2360	0	0	0	0	1	0	0	1	1	1	1	0	0	0	0	0	0	0	0	0	0	1	0	0	0	0	0	1	0	0	0	0	0	0	0	0	0	0	0	0	0	0	0	b2360	orf, hypothetical protein
b2361	0	0	0	0	1	0	0	1	1	1	1	0	0	0	0	0	0	0	0	0	1	1	0	0	0	0	0	1	0	0	0	0	0	0	0	0	0	0	0	0	0	0	1	b2361	orf, hypothetical protein
b2362	0	0	0	0	0	0	0	1	1	1	1	0	0	0	0	0	0	0	0	0	0	1	0	0	0	0	0	1	0	0	0	0	0	0	1	1	0	1	0	0	0	1	1	b2362	orf, hypothetical protein
b2363	0	0	0	0	0	0	0	0	0	0	0	0	0	1	0	0	0	0	0	0	0	0	0	0	0	0	0	0	0	0	0	0	0	0	1	1	0	1	0	0	0	1	1	b2363	orf, hypothetical protein
b2364	0	0	0	1	1	0	0	0	1	1	1	0	0	1	1	1	1	1	1	1	1	1	1	1	1	1	1	1	1	0	1	1	0	0	0	0	0	0	0	0	0	1	0	dsdC	D-serine dehydratase (deaminase) transcriptional activator
b2365	0	1	0	0	1	0	0	0	0	0	0	0	0	0	0	0	0	0	0	0	0	0	0	0	1	1	1	1	1	0	1	1	0	0	0	0	0	0	0	0	0	0	0	dsdX	transport system permease (serine?)
b2366	1	1	1	1	1	1	1	1	1	1	1	1	1	1	1	1	1	1	1	1	1	1	1	1	1	1	1	1	1	1	1	1	1	1	1	1	1	1	1	1	1	0	0	dsdA	D-serine dehydratase (deaminase)
b2367	1	0	1	1	1	1	1	1	1	1	1	1	1	1	1	1	1	1	1	1	1	1	1	1	1	1	1	1	1	1	1	1	1	1	1	1	0	1	1	1	1	1	1	emrY	multidrug resistance protein Y
b2368	1	0	1	1	1	1	1	1	1	1	1	1	1	1	1	1	1	1	1	1	1	1	1	1	1	1	1	1	1	1	1	1	1	1	1	1	1	1	1	1	1	1	1	emrK	multidrug resistance protein K
b2369	1	0	1	1	1	1	1	1	1	1	1	1	1	1	1	1	1	1	1	1	1	0	1	1	1	1	1	1	1	1	1	1	1	1	1	1	1	1	1	1	1	1	1	evgA	putative positive transcription regulator (sensor EvgS)
b2370	1	0	1	1	1	1	1	1	1	1	1	1	1	1	1	1	0	1	1	1	1	1	1	1	1	1	1	1	1	1	1	1	1	1	1	1	1	1	1	1	1	1	1	evgS	putative sensor for regulator EvgA
b2371	1	0	1	1	1	1	1	1	1	1	1	1	1	1	1	1	1	1	1	1	1	1	1	1	1	1	1	1	1	1	1	1	1	1	1	1	0	1	1	1	1	1	1	yfdE	putative enzyme
b2372	1	0	1	1	1	1	1	1	1	1	1	1	1	1	1	1	1	1	1	1	1	1	1	1	1	1	1	1	1	1	1	1	1	1	1	1	1	1	1	1	1	1	1	b2372	putative receptor protein
b2373	1	0	1	1	1	1	1	1	1	1	1	1	1	1	1	1	1	1	1	1	1	1	1	1	1	1	1	1	1	1	1	1	1	1	1	1	1	1	1	1	1	1	1	b2373	putative enzyme
b2374	1	0	1	1	1	1	1	1	1	1	1	1	1	1	1	1	1	1	1	1	1	1	1	1	1	1	1	1	1	1	1	1	1	1	1	1	1	1	1	1	1	1	1	b2374	putative enzyme
b2375	1	0	1	1	1	1	1	1	1	1	1	1	1	1	1	1	1	1	1	1	1	1	1	1	1	1	1	1	1	1	1	1	1	1	1	1	1	1	1	1	1	1	1	b2375	orf, hypothetical protein
b2376	1	0	1	1	1	1	1	1	1	1	1	1	1	1	1	1	1	1	1	1	1	1	1	1	1	1	1	1	1	1	1	1	1	1	1	1	1	1	1	1	1	1	1	b2376	orf, hypothetical protein
b2378	1	1	1	1	1	1	1	1	1	1	1	1	1	1	1	1	1	1	1	1	1	1	1	1	1	1	1	0	1	1	0	1	1	1	1	1	1	1	1	1	1	1	1	ddg	putative heat shock protein
b2379	1	1	1	1	1	1	1	1	1	1	1	1	1	1	1	1	1	1	1	1	1	1	1	1	1	1	1	1	1	1	1	1	1	1	1	0	1	1	1	1	1	1	1	b2379	putative aminotransferase
b2381	1	1	1	1	1	1	1	1	1	1	1	1	1	1	1	1	1	1	1	1	1	1	1	1	1	1	1	1	1	1	1	1	1	1	1	1	0	1	1	1	1	1	1	b2381	putative 2-component transcriptional regulator
b2382	1	1	1	1	1	0	1	1	1	1	1	1	1	1	1	1	1	1	1	1	1	1	1	1	1	1	1	1	1	1	1	1	1	1	1	1	1	1	1	1	1	1	1	b2382	putative ARAC-type regulatory protein
b2384	1	1	1	1	1	1	1	1	1	1	1	1	1	1	1	1	1	1	1	1	1	1	1	1	1	1	1	1	0	1	1	1	1	1	1	1	1	1	1	1	1	1	1	b2384	orf, hypothetical protein
b2385	1	1	1	1	1	0	1	1	1	1	1	1	1	1	1	1	1	1	1	1	1	1	1	1	0	0	0	1	0	1	1	1	1	1	1	1	1	1	1	1	1	1	1	b2385	putative peptidase
b2386	1	1	1	1	1	1	1	1	1	1	1	1	1	1	1	1	1	1	1	1	1	1	1	1	1	1	1	1	1	1	1	1	1	1	1	1	0	1	1	1	1	1	1	b2386	putative transport protein
b2387	1	1	1	1	0	1	1	1	1	1	1	1	1	1	1	1	1	1	1	1	1	1	1	1	1	1	1	1	1	1	1	1	1	1	1	1	1	1	1	1	1	1	1	b2387	putative PTS system enzyme IIB component
b2389	0	1	1	1	1	1	1	1	1	1	1	1	1	1	1	1	1	1	1	1	1	1	1	1	1	1	1	1	1	1	1	1	1	1	1	0	0	1	1	1	1	1	1	b2389	orf, hypothetical protein
b2390	0	1	1	1	1	1	1	1	1	1	1	1	1	1	1	1	1	1	1	1	1	1	1	1	1	1	1	1	1	1	1	1	1	1	1	1	1	1	1	1	1	1	1	b2390	orf, hypothetical protein
b2391	0	1	1	1	1	1	1	1	1	1	1	1	1	1	1	1	1	1	1	1	1	1	1	1	1	1	1	1	1	1	1	1	1	1	1	1	1	1	1	1	1	1	1	b2391	orf, hypothetical protein
b2392	1	1	1	1	1	1	1	1	1	1	1	1	1	1	1	1	1	1	1	1	1	1	1	1	1	1	1	1	1	1	1	1	1	1	1	0	1	1	1	1	1	1	1	b2392	putative transport system permease
b2405	0	1	0	1	0	0	0	0	0	0	0	0	0	0	0	0	0	0	0	0	0	0	0	0	0	0	0	0	0	0	0	0	0	0	0	0	0	0	0	0	0	0	0	xapR	regulator for xapA
b2406	1	1	1	1	1	1	0	1	1	0	1	1	0	1	0	1	1	0	0	1	1	1	1	1	0	0	0	0	0	0	0	1	0	1	1	0	0	0	0	0	0	0	0	xapB	xanthosine permease
b2407	0	1	0	1	0	0	0	0	0	0	0	0	0	0	0	0	0	0	0	0	0	0	0	0	0	0	0	0	0	0	0	0	0	0	0	0	0	0	0	0	0	0	0	xapA	xanthosine phosphorylase
b2408	0	0	0	1	0	0	0	0	0	0	0	0	0	0	0	0	0	0	0	0	0	0	0	0	0	0	0	0	0	0	0	0	0	0	0	0	0	0	0	0	0	0	0	yfeN	putative sugar hydrolase
b2409	1	0	1	1	1	1	1	1	1	1	1	1	1	1	1	1	1	1	1	1	1	1	1	1	1	1	1	1	1	1	1	1	1	1	1	1	1	1	1	1	1	1	1	yfeR	putative transcriptional regulator LYSR-type

b2414	1	1	1	1	0	0	0	0	0	0	0	0	1	1	0	0	1	0	0	0	1	0	0	0	1	1	1	1	1	1	1	1	0	1	1	0	0	0	0	0	1	0	0	cysK	cysteine synthase A, O-acetylserine sulfhydrolase A
b2415	1	1	1	1	1	1	1	0	1	1	1	1	1	1	1	1	1	1	1	1	1	1	1	1	1	1	1	0	0	1	0	1	1	1	1	1	1	1	1	1	1	1	1	ptsH	PTS system protein HPr
b2416	1	1	1	1	1	1	1	1	1	1	1	1	1	1	1	1	1	1	1	1	1	1	1	1	1	1	1	1	1	1	1	1	1	1	1	1	0	1	1	1	1	1	1	ptsI	PEP-protein phosphotransferase system enzyme I
b2419	1	1	1	1	1	1	1	1	1	1	1	1	1	1	1	1	1	1	1	1	1	1	1	1	1	0	0	1	0	0	0	1	1	1	1	0	1	1	1	1	1	1	1	yfeK	orf, hypothetical protein
b2420	0	0	0	0	0	0	0	0	0	0	0	0	0	0	0	0	0	0	0	0	0	0	0	0	0	0	0	0	0	0	0	0	1	1	1	1	1	1	1	1	1	1	1	b2420	orf, hypothetical protein
b2422	1	1	1	1	1	1	1	1	1	1	1	1	1	1	1	1	1	1	1	1	1	1	1	1	1	1	1	1	1	1	1	1	1	1	1	0	1	1	1	1	1	1	1	cysA	ATP-binding component of sulfate permease A protein; chromate resistance
b2427	1	0	1	1	1	1	1	1	1	1	1	1	1	1	1	1	1	1	1	1	1	1	1	1	1	1	1	1	1	1	1	1	1	1	1	1	1	1	1	1	1	1	1	yfeT	orf, hypothetical protein
b2428	1	1	1	1	1	1	1	1	1	1	1	1	1	1	1	1	1	1	1	1	1	1	1	1	1	1	1	1	1	1	1	1	1	1	1	0	0	1	1	1	1	1	1	yfeU	putative regulator
b2430	1	0	1	1	1	1	1	1	1	1	1	1	1	1	1	1	1	1	1	1	1	1	1	1	1	1	1	1	1	1	1	1	0	0	0	0	0	0	0	0	0	0	0	b2430	putative beta-lactamase
b2432	1	1	1	1	1	1	1	1	1	1	1	1	1	1	1	1	1	1	1	1	1	1	1	1	1	1	1	1	1	1	1	1	1	1	1	0	0	1	1	1	1	1	1	b2432	orf, hypothetical protein
b2433	1	1	1	1	1	1	1	1	1	1	1	1	1	1	1	1	1	1	1	1	1	1	1	1	1	1	1	1	1	1	1	1	1	1	1	0	1	1	1	1	1	1	1	b2433	orf, hypothetical protein
b2435	1	1	1	1	1	1	1	1	1	1	1	1	1	1	1	1	1	1	1	1	1	1	1	1	1	1	1	1	1	1	1	1	1	1	1	0	0	1	1	1	1	1	1	amiA	N-acetylmuramoyl-l-alanine amidase I
b2438	1	1	1	1	1	1	1	1	1	1	1	1	1	1	1	1	1	1	1	1	1	1	1	1	1	1	1	1	1	1	1	1	1	1	1	1	0	1	1	1	1	1	1	b2438	orf, hypothetical protein
b2439	1	1	1	1	1	0	0	0	0	1	1	1	0	1	1	1	0	0	1	1	1	0	1	1	0	1	1	1	1	1	0	0	1	1	1	1	1	1	1	1	1	1	1	b2439	orf, hypothetical protein
b2440	1	1	1	1	1	1	1	1	1	1	1	1	1	1	1	1	1	1	1	1	1	1	1	1	1	1	1	1	1	1	1	1	0	0	0	0	0	0	0	0	1	1	1	eutC	ethanolamine ammonia-lyase, light chain
b2441	1	1	1	1	1	1	1	1	1	1	1	1	1	1	1	1	1	1	1	1	0	1	1	1	1	1	0	1	1	1	1	1	0	0	0	0	0	0	0	0	1	1	1	eutB	ethanolamine ammonia-lyase, heavy chain
b2442	0	0	0	0	1	0	0	0	0	0	0	0	0	0	0	0	0	0	0	0	0	0	0	0	0	1	0	0	0	0	0	0	0	0	0	0	0	0	0	0	1	1	1	b2442	putative prophage integrase
b2443	0	0	0	0	0	0	0	0	0	0	0	0	0	0	0	0	0	0	0	0	0	0	0	0	0	0	0	0	0	0	0	0	0	0	0	0	0	0	0	0	0	1	1	b2443	orf, hypothetical protein
b2444	0	0	0	0	0	0	0	0	0	0	0	0	0	0	0	0	0	0	0	0	0	0	0	0	0	0	0	0	0	0	0	0	0	0	0	0	0	0	0	0	0	0	0	b2444	orf, hypothetical protein
b2445	0	0	0	0	0	0	0	0	0	0	0	0	0	0	0	0	0	0	0	0	0	0	0	0	0	0	0	0	0	0	0	0	0	0	0	0	0	0	0	0	0	0	0	b2445	orf, hypothetical protein
b2446	0	0	0	0	0	0	0	0	0	0	0	0	0	0	0	0	0	0	0	0	0	0	0	0	0	0	0	0	0	0	0	0	0	0	0	0	0	0	0	0	0	0	0	b2446	orf, hypothetical protein
b2447	0	0	0	0	0	0	0	0	0	0	0	0	0	0	0	0	0	0	0	0	0	0	0	0	0	0	0	0	0	0	0	0	0	0	0	0	0	0	0	0	0	0	0	b2447	orf, hypothetical protein
b2448	0	0	0	0	0	0	0	0	0	0	0	0	0	0	0	0	0	0	0	0	0	0	0	0	0	0	0	0	0	0	0	0	0	0	0	0	0	0	0	0	0	0	0	b2448	orf, hypothetical protein
b2449	0	0	0	0	0	0	0	0	0	0	0	0	0	0	0	0	0	0	0	0	0	0	0	0	0	0	0	0	0	0	0	0	0	0	0	0	0	0	0	0	0	0	0	b2449	orf, hypothetical protein
b2450	0	0	0	0	0	0	0	0	0	0	0	0	0	0	0	0	0	0	0	0	0	0	0	0	0	0	0	0	0	0	0	0	0	0	0	0	0	0	0	0	0	0	0	b2450	orf, hypothetical protein
b2451	0	1	1	1	1	1	1	1	1	1	1	1	0	0	0	1	1	1	1	1	1	1	1	1	1	1	1	1	1	1	1	1	1	1	1	0	1	1	1	1	1	1	1	b2451	orf, hypothetical protein
b2452	0	1	1	1	1	1	1	1	1	1	1	1	0	0	0	0	1	1	1	1	1	1	1	1	1	1	1	1	1	1	1	1	0	0	0	0	0	0	0	0	0	1	1	eutH	ethanolamine utilization; homolog of Salmonella putative transport protein
b2453	0	1	1	1	1	1	1	1	1	1	1	1	1	0	1	1	1	1	1	1	1	1	1	1	1	1	1	1	1	1	1	1	1	0	0	0	1	1	1	1	1	1	1	eutG	ethanolamine utilization; homolog of Salmonella enzyme, similar to iron-containing alcohol dehydrogenase
b2454	0	1	1	1	1	1	1	1	1	1	1	1	1	1	1	1	1	1	1	1	1	1	1	1	1	1	1	1	1	1	1	1	0	0	0	0	0	0	0	0	0	1	1	eutJ	ethanolamine utilization; homolog of Salmonella gene
b2455	0	1	1	1	1	1	1	1	1	1	1	1	1	1	1	1	1	1	1	1	1	1	1	1	1	1	1	1	1	1	1	1	0	0	0	0	0	0	0	0	0	1	1	eutE	ethanolamine utilization; similar to acetaldehyde dehydrogenase
b2456	0	1	1	1	1	1	1	1	1	1	1	1	0	1	1	1	1	1	1	1	1	1	1	1	1	1	1	1	1	0	1	1	0	0	0	0	0	0	0	0	0	1	1	cchB	detox protein
b2457	0	1	1	1	1	1	1	1	1	1	1	1	0	1	1	1	1	1	1	1	1	1	1	1	1	1	1	1	1	0	1	1	0	0	0	0	0	0	0	0	0	1	1	cchA	detox protein
b2458	0	1	1	1	1	1	1	1	1	1	1	1	0	1	1	1	1	1	1	1	1	1	1	1	1	0	0	1	1	1	1	1	0	0	0	0	0	0	0	0	0	1	1	eutI	ethanolamine utilization; homolog of Salmonella acetyl/butyryl P transferase
b2459	0	1	1	1	1	1	1	1	1	1	1	1	0	1	1	1	1	1	1	1	1	1	1	1	1	1	0	1	1	1	1	1	0	0	0	0	0	0	0	0	0	0	0	b2459	orf, hypothetical protein
b2460	0	1	1	1	1	1	1	1	1	1	1	1	0	1	1	1	1	1	1	1	1	1	1	1	1	1	1	1	1	1	1	1	1	1	1	1	1	1	1	1	1	1	1	b2460	orf, hypothetical protein
b2461	0	1	1	1	1	1	1	1	1	1	1	1	0	1	1	1	1	1	1	1	1	1	1	1	1	1	1	1	1	1	1	1	1	1	1	1	0	1	1	1	1	1	1	b2461	orf, hypothetical protein
b2462	1	1	1	1	1	1	1	1	1	1	1	1	1	1	1	1	0	1	1	1	1	1	1	1	1	1	1	1	1	1	1	1	1	1	1	1	1	1	0	1	1	1	1	b2462	orf, hypothetical protein
b2463	1	1	1	1	1	1	1	1	1	1	1	1	0	1	1	1	0	1	1	1	1	1	1	1	1	1	1	1	1	1	1	1	0	1	1	1	1	1	1	1	1	1	1	b2463	putative multimodular enzyme
b2464	1	1	1	1	1	1	1	1	1	1	1	1	1	1	1	1	0	1	1	1	1	1	1	1	1	1	1	1	1	1	1	1	1	1	1	0	1	1	1	1	1	1	1	talA	transaldolase A
b2465	1	1	1	1	1	1	1	1	1	1	1	1	1	1	1	1	1	1	1	1	1	1	1	1	1	1	1	1	1	1	1	1	0	1	1	1	1	1	1	1	1	1	1	tktB	transketolase 2 isozyme
b2466	1	0	1	1	1	1	1	1	1	1	1	1	1	1	1	1	0	1	1	1	1	1	1	1	1	1	1	1	1	1	1	1	1	1	1	1	1	1	1	1	1	1	1	b2466	orf, hypothetical protein
b2467	1	1	1	1	1	1	1	1	1	1	1	1	1	1	1	1	0	1	1	1	1	1	1	1	1	1	1	1	1	1	1	0	1	1	1	1	1	1	1	1	1	1	1	yffH	orf, hypothetical protein
b2468	1	1	1	1	1	1	1	1	1	1	1	1	1	1	1	1	0	1	1	1	1	1	1	1	1	1	1	1	1	1	1	1	1	1	1	1	1	1	1	1	1	1	1	yffG	putative oxidoreductase, Fe-S subunit
b2469	1	1	1	1	1	1	1	1	1	1	1	1	1	1	1	1	0	1	1	1	1	1	1	1	1	1	1	1	1	1	1	1	1	1	1	0	1	1	1	1	1	1	1	narQ	sensor for nitrate reductase system, protein histidine kinase (acts on NarP and narL)
b2470	1	1	1	1	1	1	1	1	1	1	1	1	1	1	1	1	0	1	1	1	1	1	1	1	1	1	1	1	1	1	1	1	1	1	1	0	0	1	1	1	1	1	1	acrD	sensitivity to acriflavine, integral membrane protein, possible efflux pump
b2472	1	1	1	1	1	1	1	1	1	1	1	1	1	1	1	1	1	1	1	1	1	1	1	1	1	1	1	1	1	1	1	1	1	1	1	1	0	1	1	1	1	1	1	dapE	N-succinyl-diaminopimelate deacylase
b2473	1	1	1	1	1	1	1	1	1	1	1	1	1	1	1	1	1	1	1	1	1	1	1	1	1	1	1	1	1	1	1	1	1	1	1	1	0	1	1	1	1	1	1	ypfH	orf, hypothetical protein
b2476	1	1	1	1	1	1	1	1	1	1	1	1	1	1	1	1	1	1	1	1	1	1	1	1	1	1	1	1	1	1	1	1	1	1	1	0	1	1	1	1	1	1	1	purC	phosphoribosylaminoimidazole-succinocarboxamide synthetase = SAICAR synthetase
b2477	1	1	1	1	1	1	1	1	1	1	1	1	1	1	1	1	1	1	1	1	1	1	1	1	1	1	1	1	1	1	1	1	1	1	1	1	0	1	1	1	1	1	1	nlpB	lipoprotein-34
b2481	1	0	1	1	1	1	1	1	1	1	1	1	1	1	1	1	1	1	1	1	1	1	1	1	1	1	1	1	1	1	1	1	1	1	1	1	1	1	1	1	1	1	1	hyfA	hydrogenase 4 Fe-S subunit
b2482	1	0	1	1	1	1	1	1	1	1	1	1	1	1	1	1	1	1	1	1	1	1	1	1	1	1	1	1	1	1	1	1	1	1	1	1	1	1	1	1	1	1	1	hyfB	hydrogenase 4 membrane subunit
b2483	1	0	1	1	1	1	1	1	1	1	1	1	1	1	1	1	1	1	1	1	1	1	1	1	1	1	1	1	1	1	1	1	1	1	1	1	1	1	1	1	1	1	1	hyfC	hydrogenase 4 membrane subunit
b2484	1	0	1	1	1	1	1	1	1	1	1	1	1	1	1	1	1	1	1	1	1	1	1	1	1	1	1	1	1	1	1	1	1	1	1	0	1	1	1	1	1	1	1	hyfD	hydrogenase 4 membrane subunit
b2485	1	0	1	1	1	1	1	1	1	1	1	1	1	1	1	1	1	1	1	1	1	1	1	1	1	1	1	1	1	1	1	1	1	1	1	1	1	1	1	1	1	1	1	hyfE	hydrogenase 4 membrane subunit
b2486	1	0	1	1	1	1	1	1	1	1	1	1	1	1	1	1	1	1	1	1	1	1	1	1	1	1	1	1	1	1	1	1	1	1	1	1	1	1	1	1	1	1	1	hyfF	hydrogenase 4 membrane subunit
b2487	1	0	1	0	1	1	1	1	1	1	1	1	1	1	1	1	1	1	1	1	1	1	1	1	1	1	1	1	1	1	1	1	1	1	1	0	1	1	1	1	1	1	1	hyfG	hydrogenase 4 subunit
b2488	1	1	1	1	1	1	1	1	1	1	1	1	1	1	1	1	1	1	1	1	1	1	1	1	1	1	1	1	1	1	1	1	1	1	1	0	1	1	1	1	1	1	1	hyfH	hydrogenase 4 Fe-S subunit
b2490	1	0	1	0	1	1	1	1	1	1	1	1	1	1	1	1	1	1	1	1	1	1	1	1	1	1	1	1	1	1	1	1	1	1	1	0	1	1	1	1	1	1	1	b2490	putative protein processing element
b2491	1	0	1	1	1	1	1	1	1	1	1	1	1	1	1	1	1	1	1	1	1	1	1	1	1	1	1	1	1	1	1	1	1	1	1	1	1	1	1	1	1	1	1	hyfR	putative 2-component regulator, interaction with sigma 54
b2492	1	0	1	1	1	1	1	1	1	1	1	1	1	1	1	1	1	1	1	1	1	1	1	1	1	1	1	1	1	1	1	1	1	1	1	1	1	1	1	1	1	1	1	focB	probable formate transporter (formate channel 2)
b2497	1	1	1	1	1	1	1	1	1	1	1	1	1	1	1	1	1	1	1	1	1	1	1	1	1	1	1	1	1	1	1	1	1	1	1	0	1	1	1	1	1	1	1	uraA	uracil transport
b2498	1	1	1	1	1	1	1	1	1	1	1	1	1	1	1	1	1	1	1	1	1	1	1	1	1	1	1	1	1	1	1	1	1	1	1	0	1	1	1	1	1	1	1	upp	uracil phosphoribosyltransferase
b2500	1	1	1	1	1	1	1	1	1	1	1	1	1	1	1	1	1	1	1	1	1	1	1	1	1	1	1	1	1	1	1	1	1	1	1	0	1	1	1	1	1	1	1	purN	phosphoribosylglycinamide formyltransferase 1
b2504	1	1	1	1	1	0	0	0	0	0	0	0	0	0	0	0	0	0	0	0	0	0	0	0	1	1	1	1	1	1	1	1	1	1	1	1	1	1	1	1	1	1	1	b2504	orf, hypothetical protein
b2505	1	1	1	1	1	0	1	1	1	1	1	1	0	0	0	0	1	1	1	1	1	1	1	1	1	1	1	1	1	1	1	1	1	1	1	0	1	1	1	1	1	1	1	b2505	putative outer membrane lipoprotein
b2509	1	1	1	1	1	1	1	1	1	1	1	1	1	1	1	1	1	1	1	1	1	1	1	1	1	0	1	1	1	1	1	1	1	1	1	1	1	1	1	1	1	1	1	xseA	exonuclease VII, large subunit
b2510	1	0	1	0	1	1	1	1	1	1	1	1	1	1	1	1	1	1	1	1	1	1	1	1	1	1	1	1	1	1	1	1	1	1	1	1	1	1	1	1	1	1	1	b2510	orf, hypothetical protein
b2512	1	1	1	1	1	0	0	0	0	1	1	1	0	1	1	0	1	1	1	1	1	1	1	1	0	1	1	0	0	0	0	1	1	1	1	1	1	1	1	1	1	1	1	b2512	putative dehydrogenase
b2516	1	1	1	0	0	1	1	1	1	1	1	1	1	1	1	1	1	1	1	0	1	1	1	1	1	1	1	0	0	1	1	1	1	1	1	1	1	1	1	1	1	1	1	yfgA	putative membrane protein
b2520	1	1	1	0	1	1	1	1	1	1	1	1	1	1	1	1	1	1	1	1	1	1	1	1	1	1	1	1	1	1	1	1	0	0	0	0	0	0	0	0	0	0	0	b2520	orf, hypothetical protein
b2521	1	1	1	0	1	1	1	1	1	1	1	1	1	1	1	1	1	1	1	1	1	1	1	1	1	0	1	1	1	1	1	1	1	1	1	1	1	1	1	1	1	1	1	sseA	putative thiosulfate sulfurtransferase
b2527	1	1	1	1	1	1	1	1	1	1	1	1	1	1	1	1	1	1	1	1	1	1	1	1	1	1	1	1	1	1	1	1	1	1	1	1	1	1	1	0	1	1	1	yfhE	orf, hypothetical protein
b2537	0	1	1	1	1	1	1	1	1	1	1	1	1	1	1	1	1	1	1	1	1	1	1	1	1	1	1	1	1	1	1	1	1	1	1	1	1	1	1	1	1	1	1	hcaR	transcriptional activator of hca cluster
b2538	0	0	1	1	0	0	0	0	0	0	0	0	1	1	1	1	1	1	1	1	1	1	1	1	1	1	1	1	1	1	1	1	1	1	1	1	1	1	1	1	1	1	1	hcaA1	large terminal subunit of phenylpropionate dioxygenase
b2539	0	0	1	1	0	0	0	0	0	0	0	0	1	1	1	1	1	1	1	1	1	1	1	1	1	1	1	1	1	1	1	1	1	1	1	1	1	1	1	0	1	1	1	hcaA2	small terminal subunit of phenylpropionate dioxygenase
b2540	0	0	1	1	0	0	0	0	0	0	0	0	1	1	1	1	1	1	1	1	1	1	1	1	1	1	1	1	1	1	1	1	1	1	1	1	1	1	1	1	1	1	1	hcaC	ferredoxin subunit of phenylpropionate dioxygenase
b2541	0	0	1	1	0	0	0	0	0	0	0	0	1	1	1	1	1	1	1	1	1	1	1	1	1	1	1	1	1	1	1	1	1	1	1	1	1	1	1	1	1	1	1	hcaB	2,3-dihydroxy-2,3-dihydrophenylpropionate dehydrogenase
b2542	0	0	1	1	0	0	0	0	0	0	0	0	1	1	1	1	1	1	1	1	1	1	1	1	1	1	1	1	1	1	0	1	1	1	1	1	1	1	1	1	1	1	1	hcaD	ferredoxin reductase subunit of phenylpropionate dioxygenase
b2543	0	1	1	1	0	0	0	0	1	1	0	0	1	1	1	1	1	0	0	1	1	0	1	1	1	1	1	1	1	1	1	1	1	1	1	1	1	1	1	1	1	1	1	yphA	orf, hypothetical protein
b2544	0	1	1	0	0	0	0	0	0	0	0	0	1	1	1	1	1	0	0	0	0	0	0	0	1	1	1	1	1	1	1	1	1	1	1	1	1	1	1	1	1	1	1	yphB	orf, hypothetical protein
b2545	0	1	1	1	1	0	0	0	0	0	0	0	1	1	1	1	1	0	0	0	1	0	0	0	1	1	1	1	1	1	1	1	1	1	1	1	1	1	1	1	1	1	1	yphC	putative oxidoreductase
b2546	0	1	1	1	1	1	0	1	0	1	1	1	1	1	1	1	1	0	1	1	1	1	1	1	1	1	1	1	1	1	1	1	1	1	1	1	1	1	1	1	1	1	1	yphD	putative transport system permease protein
b2547	0	1	1	0	0	0	0	0	0	0	0	0	1	1	1	1	1	0	0	0	0	0	0	0	1	1	1	1	1	1	1	1	1	1	1	1	1	1	1	1	1	1	1	yphE	putative ATP-binding component of a transport system
b2548	1	1	1	0	0	0	0	0	0	0	0	0	1	1	1	1	1	0	0	0	0	0	0	0	1	1	1	1	1	1	1	1	1	1	1	1	1	1	1	1	1	1	1	yphF	putative LACI-type transcriptional regulator
b2549	1	1	1	1	1	0	0	0	0	0	0	0	1	1	1	1	1	1	1	1	1	1	1	1	1	1	1	1	1	1	1	1	1	1	1	1	1	1	1	1	1	1	1	yphG	orf, hypothetical protein
b2550	1	1	1	1	1	0	0	0	0	1	1	1	1	1	1	1	1	1	1	1	1	1	1	1	1	1	1	1	1	1	1	1	1	1	1	1	1	1	1	1	1	1	1	yphH	putative NAGC-like transcriptional regulator
b2556	1	1	1	1	1	1	1	1	1	1	1	1	1	1	1	1	1	1	1	1	1	1	1	1	1	1	0	1	1	0	1	1	1	1	1	1	1	1	1	1	1	1	1	yfhK	putative 2-component sensor protein
b2565	1	1	1	1	1	1	1	1	1	1	1	1	1	1	1	1	1	1	1	1	1	1	1	1	1	1	0	1	1	1	1	1	1	1	1	1	1	1	1	1	1	1	1	recO	protein interacts with RecR and possibly RecF proteins
b2575	1	1	1	1	1	0	1	1	1	1	1	1	1	1	1	1	1	1	1	1	1	1	1	1	1	1	1	1	1	1	1	1	1	1	1	1	1	1	1	1	1	1	1	yfiC	putative enzyme
b2583	1	1	1	0	1	1	1	1	1	1	1	1	1	1	1	1	1	1	1	1	1	1	1	1	1	1	1	1	1	1	1	1	1	1	1	1	1	1	1	1	1	1	1	yfiP	orf, hypothetical protein
b2593	1	1	1	1	1	1	1	1	1	1	1	1	0	1	1	1	1	1	1	1	1	1	1	1	1	1	1	1	1	1	1	1	1	1	1	1	1	1	1	1	1	1	1	yfiH	orf, hypothetical protein
b2594	1	1	1	1	1	0	1	1	1	1	1	1	1	1	1	1	1	1	1	1	1	1	1	1	1	1	1	1	1	1	1	1	1	1	1	1	1	1	1	1	1	1	1	sfhB	suppressor of ftsH mutation
b2599	1	0	1	1	1	1	1	1	1	1	1	1	1	1	0	1	1	1	1	1	1	1	1	1	1	1	1	1	1	1	1	1	1	1	1	1	1	1	1	1	1	1	1	pheA	chorismate mutase-P and prephenate dehydratase
b2600	1	0	1	1	1	1	1	1	1	1	1	1	1	1	1	1	1	1	1	1	1	1	1	1	1	1	1	1	1	1	1	1	1	1	1	1	1	1	1	1	1	1	1	tyrA	chorismate mutase-T and prephenate dehydrogenase
b2601	1	0	1	1	1	1	1	1	1	1	1	1	1	1	1	1	1	1	1	1	1	1	1	1	1	1	1	1	1	1	1	1	1	1	1	1	1	1	1	1	1	1	1	aroF	3-deoxy-D-arabinoheptulosonate-7-phosphate synthase (DAHP synthetase, tyrosine repressible)
b2602	1	0	1	1	1	1	1	1	1	1	1	1	1	1	1	1	1	1	1	1	1	1	1	1	1	1	1	1	1	1	1	1	1	1	1	1	1	1	1	1	1	1	1	yfiL	orf, hypothetical protein
b2603	1	0	1	1	1	1	1	1	1	1	1	1	1	1	1	1	1	1	1	1	1	1	1	1	1	1	1	1	1	1	1	1	1	1	1	1	1	1	1	1	1	1	1	b2603	orf, hypothetical protein
b2604	1	0	1	1	1	1	1	1	1	1	1	1	1	1	1	1	1	1	1	1	1	1	1	1	1	1	1	1	1	1	1	1	1	1	1	1	1	1	1	1	1	1	1	yfiN	orf, hypothetical protein
b2605	1	1	1	1	1	1	1	0	1	1	1	1	1	1	1	1	1	1	1	1	1	1	1	1	1	1	1	1	1	1	1	1	1	1	1	1	1	1	1	1	1	1	1	yfiB	putative outer membrane protein
b2617	1	1	1	1	1	1	1	1	1	1	1	1	1	1	0	1	1	1	1	1	1	1	1	1	1	1	1	1	1	1	1	1	1	1	1	1	1	1	1	1	1	1	1	smpA	small membrane protein A
b2622	0	0	0	0	0	0	0	0	0	0	0	1	0	0	0	0	0	1	0	0	1	0	1	1	0	0	0	0	0	0	0	1	1	1	1	0	0	1	1	1	1	1	1	intA	prophage CP4-57 integrase
b2623	0	0	0	0	1	0	0	1	1	1	1	1	0	0	1	0	0	0	0	1	1	0	1	1	0	0	0	1	1	0	1	0	0	0	0	1	1	1	1	1	1	1	1	yfjH	putative histone
b2624	0	0	0	0	0	0	0	0	0	0	0	0	0	0	0	0	0	0	0	0	0	0	0	0	0	0	0	0	0	0	0	0	0	0	0	0	0	0	0	0	0	0	0	alpA	prophage CP4-57 regulatory protein alpA
b2625	0	0	0	0	0	0	0	0	0	0	0	0	0	0	0	0	0	0	0	0	0	0	0	0	0	0	0	0	0	0	0	0	0	0	0	0	0	0	0	0	0	0	0	yfjI	orf, hypothetical protein
b2626	0	0	0	0	0	0	0	0	0	0	0	0	0	0	0	0	0	0	0	0	0	0	0	0	0	0	0	0	0	0	0	0	0	0	0	0	0	0	0	0	0	0	0	yfjJ	orf, hypothetical protein
b2627	0	0	0	0	0	0	0	0	0	0	0	0	0	0	0	0	1	0	0	0	1	1	1	1	0	0	0	0	0	0	0	0	0	0	0	0	0	0	0	0	0	0	0	yfjK	orf, hypothetical protein

b2628	0	0	0	0	0	0	0	0	0	0	0	0	0	0	0	0	0	0	0	0	0	0	0	0	0	0	0	0	0	0	0	0	0	0	0	0	0	0	0	0	0	0	0	yfjL	orf, hypothetical protein
b2629	0	0	0	0	0	0	0	0	0	0	0	0	0	0	0	0	0	0	0	0	0	0	0	0	0	0	0	0	0	0	0	0	0	0	0	0	0	0	0	0	0	0	0	yfjM	orf, hypothetical protein
b2630	0	0	0	0	0	0	0	0	0	0	0	0	0	0	0	0	0	0	0	0	0	0	0	0	0	0	0	0	0	0	0	0	0	0	0	0	0	0	0	0	0	0	0	yfjN	putative cell division protein
b2631	0	0	0	0	0	0	0	0	0	0	0	0	0	0	0	0	0	0	0	0	0	0	0	0	0	0	0	0	0	0	0	0	0	0	0	0	0	0	0	0	0	0	0	yfjO	orf, hypothetical protein
b2632	0	0	0	0	0	0	0	0	0	0	0	0	0	0	0	0	0	0	0	0	0	0	0	0	0	0	0	0	0	0	0	0	0	0	0	0	0	0	0	0	0	0	0	yfjP	putative GTP-binding protein
b2633	0	0	0	0	0	0	0	0	0	0	0	0	0	0	0	0	0	0	0	0	0	0	0	0	0	0	0	0	0	0	0	0	0	0	0	0	0	0	0	0	0	0	0	yfjQ	orf, hypothetical protein
b2634	0	0	0	0	0	0	0	0	0	0	0	0	0	0	0	0	0	0	0	0	0	0	0	0	0	0	0	0	0	0	0	0	0	0	0	0	0	0	0	0	0	0	0	yfjR	orf, hypothetical protein
b2636	0	0	0	0	0	0	0	0	0	0	0	0	0	0	0	0	0	0	0	0	0	0	0	0	0	0	0	0	0	0	0	0	0	0	0	0	0	0	0	0	0	0	0	b2636	orf, hypothetical protein
b2637	0	0	0	0	0	0	0	0	0	0	0	0	0	0	0	0	0	0	0	0	0	0	0	0	0	0	0	0	0	0	0	0	0	0	0	0	0	0	0	0	0	0	0	yfjT	orf, hypothetical protein
b2638	0	0	0	0	0	0	0	0	0	0	0	0	0	0	0	0	0	0	0	0	0	0	0	0	0	0	0	0	0	0	0	0	0	0	0	0	0	0	0	0	0	0	0	b2638	orf, hypothetical protein
b2639m	1	0	1	0	0	0	0	0	0	0	0	0	0	1	0	0	0	0	0	0	0	0	0	0	0	0	0	0	0	0	0	0	0	1	1	0	0	0	0	0	0	0	0	-	Arsenite permease
b2642	0	0	0	0	0	0	0	0	0	0	0	0	0	0	0	0	0	0	0	0	0	0	0	0	0	0	0	0	0	0	0	0	0	0	0	0	0	0	0	0	0	0	0	yfjW	orf, hypothetical protein
b2643	0	0	0	0	0	0	0	0	0	0	0	0	0	0	0	0	0	0	0	0	0	0	0	0	0	0	0	0	0	0	0	0	0	0	0	0	0	0	0	0	0	0	0	yfjX	orf, hypothetical protein
b2644	1	0	0	0	0	0	0	0	0	0	0	0	0	0	0	0	0	0	0	0	0	0	0	0	0	0	0	0	0	0	0	0	0	0	1	0	0	0	0	0	0	0	0	yfjY	putative DNA repair protein
b2645	0	0	0	0	0	0	0	0	0	0	0	0	0	0	0	0	0	0	0	0	0	0	0	0	0	0	0	0	0	0	0	0	0	0	0	0	0	0	0	0	0	0	0	yfjZ	orf, hypothetical protein
b2646	0	0	0	0	0	0	0	0	0	0	0	0	0	0	0	0	0	0	0	0	0	0	0	0	0	0	0	0	0	0	0	0	0	0	0	0	0	0	0	0	0	0	0	ypjF	orf, hypothetical protein
b2647	1	0	1	1	0	0	0	0	0	0	0	0	0	0	0	0	1	0	0	0	0	0	0	0	0	1	1	1	1	1	1	1	0	0	0	0	0	0	0	0	0	0	0	ypjA	putative ATP-binding component of a transport system
b2649	0	0	1	1	0	0	0	0	0	0	0	0	0	0	0	0	0	0	0	0	0	0	0	0	0	1	1	1	1	1	1	0	0	0	0	0	0	0	0	0	0	0	0	b2649	orf, hypothetical protein
b2650	0	0	1	1	0	0	0	0	0	0	0	0	0	0	0	0	0	0	0	0	0	0	0	0	0	1	1	1	1	0	1	1	0	0	0	0	0	0	0	0	0	0	0	b2650	orf, hypothetical protein
b2653	0	0	1	1	0	1	0	1	1	1	1	1	0	0	0	0	0	0	0	0	0	0	0	0	0	1	1	1	1	0	1	0	1	1	1	1	1	1	1	1	0	0	0	b2653	orf, hypothetical protein
b2654	0	0	0	1	0	1	1	1	1	0	1	1	0	0	0	0	0	0	0	0	0	0	0	0	0	1	1	1	1	0	1	0	1	1	1	1	1	1	1	1	0	0	0	b2654	orf, hypothetical protein
b2655	0	0	1	1	0	1	1	1	1	1	1	1	1	1	0	1	0	0	0	0	0	0	0	0	0	1	1	1	1	0	1	0	1	1	1	1	1	1	1	1	0	0	0	b2655	orf, hypothetical protein
b2656	0	0	1	1	0	1	1	1	1	1	1	1	1	1	1	1	0	0	0	0	1	0	0	0	0	1	1	1	1	0	1	0	1	1	1	1	1	1	1	1	0	0	0	b2656	orf, hypothetical protein
b2657	0	0	1	1	0	1	1	1	1	1	1	1	1	1	1	1	0	0	0	0	0	0	0	0	0	1	1	1	1	0	1	0	1	1	1	1	1	1	1	1	0	0	0	b2657	putative enzyme
b2658	0	0	1	1	0	1	1	1	1	1	1	1	1	1	1	1	0	0	0	0	0	0	0	0	0	1	1	1	1	1	1	1	1	1	1	1	1	1	1	1	0	0	0	b2658	orf, hypothetical protein
b2659	0	1	1	1	0	1	1	1	1	1	1	1	1	1	0	1	1	0	0	0	0	0	0	0	0	1	1	1	1	1	1	1	1	1	1	1	1	1	1	1	0	0	0	b2659	orf, hypothetical protein
b2660	0	1	1	1	0	0	0	1	0	1	1	1	0	1	1	1	1	0	0	0	0	0	0	0	0	1	0	1	0	0	0	1	0	1	1	1	1	1	1	1	0	0	0	ygaF	orf, hypothetical protein
b2661	0	1	1	1	1	1	1	1	1	1	1	1	1	1	1	1	1	0	0	1	1	1	1	1	0	1	1	1	1	1	1	1	1	1	1	1	1	1	1	1	0	1	1	gabD	succinate-semialdehyde dehydrogenase, NADP-dependent activity
b2662	1	1	1	1	0	1	1	1	1	1	1	1	1	1	1	1	1	0	0	0	0	0	0	0	1	1	1	1	1	1	1	1	1	1	1	1	1	1	1	1	0	1	1	gabT	4-aminobutyrate aminotransferase activity
b2663	1	1	1	1	0	1	1	1	1	1	1	1	1	1	1	1	1	0	0	0	0	0	0	0	1	1	1	1	1	1	1	1	1	1	1	1	1	1	1	1	0	1	1	gabP	transport permease protein of gamma-aminobutyrate
b2664	1	1	1	1	1	1	1	1	1	1	1	1	1	1	0	1	1	0	0	0	0	0	0	0	1	1	1	1	1	1	1	1	0	1	1	1	1	1	1	1	1	1	1	ygaE	putative transcriptional regulator
b2665	1	0	1	1	1	1	1	1	1	1	1	1	1	1	1	1	1	0	0	0	0	0	0	0	1	1	1	1	1	1	1	1	1	1	1	1	1	1	1	1	1	1	1	ygaU	orf, hypothetical protein
b2666	1	1	1	1	1	1	1	1	1	1	1	1	1	1	1	1	1	0	0	0	0	0	0	0	1	1	1	1	1	1	1	1	1	1	1	1	1	1	1	1	1	1	1	b2666	orf, hypothetical protein
b2667	1	1	1	1	1	1	1	1	1	1	1	1	1	1	1	1	1	0	0	0	0	0	0	0	1	1	1	1	1	1	1	1	1	1	1	1	1	1	1	1	1	1	1	b2667	orf, hypothetical protein
b2668	1	1	1	1	1	1	1	1	1	1	1	1	1	1	1	1	1	0	0	0	0	0	0	0	1	1	1	1	1	1	1	1	1	1	1	1	1	1	1	1	1	1	1	ygaP	orf, hypothetical protein
b2669	1	1	1	1	1	1	1	1	1	1	1	1	1	1	0	1	1	0	0	0	0	0	0	0	1	1	1	1	1	1	1	1	1	1	1	1	1	1	1	1	1	1	1	stpA	DNA-binding protein; H-NS-like protein; chaperone activity; RNA splicing?
b2670	1	1	1	1	1	1	1	1	1	1	1	1	1	1	1	1	1	0	0	0	0	0	0	0	1	1	1	1	1	1	1	1	1	1	1	1	1	1	1	1	1	1	1	b2670	orf, hypothetical protein
b2671	1	1	1	1	1	0	1	1	1	1	1	1	1	1	1	0	1	0	0	0	1	1	1	1	0	1	1	1	1	1	1	1	1	1	1	1	1	1	1	1	1	1	1	ygaC	orf, hypothetical protein
b2675	1	1	1	1	1	1	1	1	1	1	1	1	1	1	0	1	1	1	1	1	1	1	1	1	1	1	1	1	1	1	1	1	1	1	1	1	1	1	1	1	1	1	1	nrdE	ribonucleoside-diphosphate reductase 2, alpha subunit
b2701	1	1	1	1	1	0	1	1	0	1	1	1	1	1	1	1	1	1	1	1	1	1	1	1	1	1	1	1	1	1	1	1	1	1	1	1	1	1	1	1	1	1	1	mltB	membrane-bound lytic murein transglycosylase B
b2705	1	1	1	1	1	1	1	1	1	1	1	1	1	1	0	1	1	1	1	1	1	1	1	1	1	1	1	1	1	1	1	1	1	1	1	1	1	1	1	1	1	1	1	srlD	glucitol (sorbitol)-6-phosphate dehydrogenase
b2708	1	1	1	1	1	0	1	1	1	1	1	1	1	1	1	1	1	1	1	1	1	1	1	1	1	1	1	1	1	1	1	1	1	1	1	1	1	1	1	1	1	1	1	gutQ	orf, hypothetical protein
b2710	1	1	1	1	1	0	1	1	1	1	1	1	1	1	1	1	1	1	1	1	1	1	1	1	1	1	1	1	1	1	1	1	1	1	1	1	1	1	1	1	1	1	1	b2710	putative flavodoxin
b2711	1	1	1	1	1	1	1	1	1	1	1	1	1	1	1	1	1	1	1	1	1	1	1	1	1	1	0	1	1	1	1	1	1	1	1	1	1	1	1	1	1	1	1	ygbD	putative oxidoreductase
b2712	1	1	1	1	1	1	1	1	1	1	1	1	1	1	0	1	1	1	1	1	1	1	1	1	1	1	1	1	1	1	1	1	1	1	1	1	1	1	1	1	1	1	1	hypF	transcriptional regulatory protein
b2714	1	1	1	1	1	0	0	0	0	1	1	1	0	1	1	0	1	1	1	1	1	1	1	1	0	0	0	0	1	0	1	1	0	1	1	1	1	1	1	1	1	1	1	ascG	ascBF operon repressor
b2715	0	0	1	1	1	1	1	1	1	1	1	1	1	1	1	1	1	1	1	1	1	1	1	1	1	1	1	1	1	1	1	1	1	1	0	0	0	0	0	0	1	1	1	ascF	PTS system enzyme II ABC (asc), cryptic, transports specific beta-glucosides
b2716	1	0	1	1	1	1	1	1	1	1	1	1	1	1	1	1	1	1	1	1	1	1	1	1	1	1	1	1	1	0	1	0	1	1	0	0	0	0	0	0	1	1	1	ascB	6-phospho-beta-glucosidase; cryptic
b2717	1	1	1	1	1	1	1	1	1	1	1	1	1	1	1	1	1	1	1	1	1	1	1	1	1	1	1	1	1	1	1	1	1	1	0	0	0	0	0	0	1	1	1	hycI	protease involved in processing C-terminal end of the large subunit of hydrogenase 3
b2718	1	1	1	1	1	1	1	1	1	1	1	1	1	1	1	1	1	1	1	1	1	1	1	1	1	1	1	1	1	1	1	1	1	1	0	0	0	0	0	0	1	1	1	hycH	processing of large subunit (HycE) of hydrogenase 3 (part of the FHL complex)
b2719	1	1	1	1	1	1	1	1	1	1	1	1	1	1	1	1	1	1	1	1	1	1	1	1	1	1	1	1	1	1	1	1	1	1	0	0	0	0	0	0	1	1	1	hycG	hydrogenase activity
b2720	1	1	1	1	1	1	1	1	1	1	1	1	1	1	1	1	1	1	1	1	1	1	1	1	1	0	0	0	1	1	1	1	1	1	0	0	0	0	0	0	1	1	1	hycF	probable iron-sulfur protein of hydrogenase 3 (part of FHL complex)
b2721	1	1	1	1	1	1	1	1	1	1	1	1	1	1	1	1	1	1	1	1	1	1	1	1	0	0	1	1	1	1	1	1	0	1	1	1	1	1	1	1	1	1	1	hycE	large subunit of hydrogenase 3 (part of FHL complex)
b2724	1	1	1	1	1	1	1	1	1	1	1	1	1	0	1	0	1	1	1	1	1	1	1	1	1	1	1	1	1	1	1	1	1	1	1	1	1	1	1	1	1	1	1	hycB	probable small subunit of hydrogenase-3, iron-sulfur protein (part of formate hydrogenlyase (FHL) complex)
b2728	1	1	1	1	1	1	1	1	1	1	1	1	1	1	1	1	1	1	1	1	1	0	1	1	1	1	1	1	1	1	1	1	1	1	1	1	1	1	1	1	1	1	1	hypC	pleiotrophic effects on 3 hydrogenase isozymes
b2729	1	1	1	1	1	1	1	1	1	1	1	1	1	1	1	1	1	1	1	1	1	0	1	1	1	1	1	1	1	1	1	1	1	1	1	1	1	1	1	1	1	1	1	hypD	pleiotrophic effects on 3 hydrogenase isozymes
b2730	1	0	1	1	1	1	1	1	1	1	1	1	1	1	1	1	1	1	1	1	1	1	1	1	1	1	1	1	1	1	1	1	1	1	1	1	1	1	1	1	1	1	1	hypE	plays structural role in maturation of all 3 hydrogenases
b2732	1	1	1	1	1	1	1	1	1	1	1	1	1	1	1	1	1	1	1	1	1	0	1	1	1	1	1	1	1	1	1	1	1	1	1	1	1	1	1	1	1	1	1	ygbA	orf, hypothetical protein
b2733	1	1	1	1	1	1	1	1	1	1	1	1	1	1	1	1	1	1	1	1	1	0	1	1	1	1	1	1	1	1	1	1	1	1	1	1	1	1	1	1	1	1	1	mutS	methyl-directed mismatch repair
b2734	0	1	1	1	1	1	1	1	1	1	1	1	1	1	0	1	1	1	1	1	1	0	1	1	1	1	1	1	1	1	1	1	1	1	1	1	1	1	1	1	1	1	1	pphB	protein phosphatase 2
b2735	0	1	1	1	1	1	1	1	1	1	1	1	1	1	1	1	1	1	1	1	1	0	1	1	1	1	1	1	1	1	1	1	1	1	1	1	1	1	1	1	1	1	1	ygbI	putative DEOR-type transcriptional regulator
b2736	0	1	1	1	1	1	1	1	1	1	1	1	1	1	1	1	1	1	1	1	1	0	1	1	1	1	1	1	1	1	1	1	1	1	1	1	1	1	1	1	1	1	1	b2736	putative dehydrogenase
b2737	0	1	1	1	1	1	1	1	1	1	1	1	1	1	1	1	1	1	1	1	1	0	1	1	1	1	1	1	0	1	1	1	0	0	0	0	0	0	0	0	1	1	1	b2737	orf, hypothetical protein
b2738	0	1	1	1	1	1	1	1	1	1	1	1	1	1	1	1	1	1	1	1	1	0	1	1	1	1	1	1	0	1	1	1	0	0	0	0	0	0	0	0	1	1	1	ygbL	putative epimerase/aldolase
b2739	0	1	1	1	1	1	1	1	1	1	1	1	1	1	1	1	1	1	1	1	1	0	1	1	1	1	1	1	1	1	1	1	0	0	0	0	0	0	0	0	0	1	1	ygbM	orf, hypothetical protein
b2740	0	1	1	1	1	1	1	1	1	1	1	1	1	1	1	1	1	1	1	1	1	0	1	1	1	1	1	1	1	1	1	1	0	0	0	0	0	0	0	0	0	1	1	b2740	putative transport protein
b2741	1	1	1	1	1	1	1	1	1	1	1	1	1	1	1	1	1	1	1	1	1	0	1	1	1	1	1	1	1	1	1	1	1	1	1	1	1	1	1	1	1	1	1	rpoS	RNA polymerase, sigma S (sigma38) factor; synthesis of many growth phase related proteins
b2742	1	1	1	1	1	1	1	1	1	1	1	1	1	1	1	1	1	1	1	1	1	0	1	1	1	1	1	1	1	1	1	1	1	1	1	1	1	1	1	1	1	1	1	nlpD	lipoprotein
b2745	1	1	1	1	1	1	1	1	1	1	1	1	1	1	0	1	1	1	1	1	1	1	1	1	1	1	1	1	1	1	1	1	0	1	1	1	1	1	1	1	1	1	1	ygbO	putative hydrogenase subunit
b2754	1	0	0	0	0	0	0	0	0	0	0	0	0	0	0	0	0	0	0	0	0	0	0	0	0	0	0	0	0	0	0	0	0	0	0	0	0	0	0	0	0	0	0	ygbF	orf, hypothetical protein
b2755	1	0	0	0	0	0	0	0	0	0	0	0	0	0	0	0	0	0	0	0	0	0	0	0	0	0	0	0	0	0	0	0	0	0	0	0	0	0	0	0	0	0	0	b2755	orf, hypothetical protein
b2756	1	0	0	0	0	0	0	0	0	0	0	0	0	0	0	0	0	0	0	0	0	0	0	0	0	0	0	0	0	0	0	0	0	0	0	0	0	0	0	0	0	0	0	b2756	orf, hypothetical protein
b2757	1	0	0	0	0	0	0	0	0	0	0	0	0	0	0	0	0	0	0	0	0	0	1	0	0	0	0	0	0	0	0	0	0	0	0	0	0	0	0	0	0	0	0	b2757	orf, hypothetical protein
b2758	0	0	0	0	0	0	0	0	0	0	0	0	0	0	0	0	0	0	0	0	0	0	0	0	0	0	0	0	0	0	0	0	0	0	0	0	0	0	0	0	0	0	0	b2758	orf, hypothetical protein
b2759	0	0	0	0	0	0	0	0	0	0	0	0	0	0	0	0	0	0	0	0	0	0	0	0	0	0	0	0	0	0	0	0	0	0	0	0	0	0	0	0	0	0	0	ygcK	orf, hypothetical protein
b2760	0	0	0	0	0	0	0	0	0	0	0	0	0	0	0	0	0	0	0	0	0	0	0	0	0	0	0	0	0	0	0	0	0	0	0	0	0	0	0	0	0	0	0	b2760	orf, hypothetical protein
b2761	0	0	0	0	0	0	0	0	0	0	0	0	0	0	0	0	0	0	0	0	0	0	0	0	0	0	0	0	0	0	0	0	0	0	0	0	0	0	0	0	0	0	0	ygcB	orf, hypothetical protein
b2764	1	1	1	1	1	0	1	1	1	1	1	1	1	1	1	1	1	1	1	1	1	1	1	1	0	1	1	1	1	1	1	1	1	1	1	1	1	1	1	1	1	1	1	cysJ	sulfite reductase (NADPH), flavoprotein beta subunit
b2765	1	1	1	1	1	1	1	1	1	1	1	1	1	1	0	1	1	1	1	1	1	1	1	1	1	1	1	1	1	1	1	1	1	1	1	1	1	1	1	1	1	1	1	ygcM	putative 6-pyruvoyl tetrahydrobiopterin synthase
b2766	1	1	1	1	1	1	1	1	1	1	1	1	1	1	1	0	1	1	1	1	1	1	1	1	1	1	1	1	1	1	1	1	1	1	1	1	1	1	1	1	1	1	1	ygcN	orf, hypothetical protein
b2768	1	1	1	1	1	0	0	0	0	1	1	1	0	1	1	0	1	0	1	1	1	1	1	1	0	1	1	0	1	0	0	1	1	1	1	1	1	1	1	1	1	1	0	ygcP	putative anti-terminator regulatory protein
b2769	1	1	1	1	1	1	1	1	1	1	1	1	1	1	1	1	1	1	1	1	1	1	1	1	1	1	1	1	1	1	1	1	1	1	1	1	1	1	1	1	1	0	0	ygcQ	putative flavoprotein
b2770	1	1	1	1	1	1	1	1	1	1	1	1	1	1	1	1	1	1	1	1	1	1	1	1	1	1	1	1	1	1	1	1	1	1	1	1	1	1	1	1	1	0	0	ygcR	putative transport protein
b2771	1	1	1	1	1	1	1	1	1	1	1	1	0	0	0	0	1	1	1	1	1	1	1	1	1	1	1	1	1	1	1	1	1	1	1	0	0	0	1	1	1	0	0	ygcS	putative transport protein
b2772	1	1	1	1	1	1	1	1	1	1	1	1	1	0	1	1	1	1	1	1	1	1	1	1	1	1	1	1	1	1	1	1	1	1	1	1	1	1	1	1	1	1	1	b2772	orf, hypothetical protein
b2773	1	0	1	1	1	1	1	1	1	1	1	1	1	0	0	1	1	1	1	1	1	0	1	1	1	1	1	0	1	1	1	1	1	0	0	1	1	1	1	1	1	1	1	ygcU	putative oxidoreductase subunit
b2774	1	1	1	1	0	0	0	0	0	0	0	0	0	0	0	0	0	0	0	0	0	0	0	0	1	1	1	0	1	1	1	1	0	0	0	0	0	0	0	0	1	0	0	ygcW	putative oxidoreductase
b2775	1	1	1	1	0	0	0	0	0	0	0	0	1	0	0	0	1	0	0	0	0	0	0	0	1	1	1	1	1	1	1	1	0	0	0	0	0	0	0	0	1	0	0	yqcE	putative transport protein
b2776	1	1	1	1	0	0	0	0	0	0	0	0	1	0	0	0	1	0	0	0	0	0	0	0	1	1	1	0	1	1	1	1	0	0	0	0	0	0	0	0	1	0	0	ygcE	putative kinase
b2778	0	0	1	1	1	1	1	1	1	1	1	1	1	1	1	1	1	0	1	1	1	1	1	1	1	1	1	1	1	1	1	1	1	1	1	1	1	1	1	1	1	1	1	ygcG	orf, hypothetical protein
b2781	1	1	1	1	1	1	1	1	1	1	1	1	1	1	1	1	1	1	1	1	1	1	1	1	1	1	0	1	1	1	1	1	1	1	1	1	1	1	1	1	1	1	1	mazG	orf, hypothetical protein
b2782	1	0	1	1	1	1	1	1	1	1	1	1	1	1	1	1	1	1	1	1	1	1	1	1	1	1	1	1	1	1	1	1	0	0	0	0	0	0	0	0	0	0	0	chpA	probable growth inhibitor, PemK-like, autoregulated
b2783	1	0	1	1	1	1	1	1	1	1	1	1	1	1	1	1	1	1	1	1	1	1	1	1	1	1	1	1	1	1	1	1	0	0	0	0	0	0	0	0	0	0	0	chpR	suppressor of inhibitory function of ChpA, PemI-like, autoregulated
b2787	1	0	1	1	1	1	1	1	1	1	1	1	0	1	1	1	1	1	1	1	1	1	1	1	1	1	1	1	1	0	1	0	1	1	1	1	1	1	1	1	1	1	1	ygcX	putative glucarate dehydratase
b2788	1	0	1	1	1	1	1	1	1	1	1	1	0	1	0	1	1	1	1	1	1	1	1	1	1	1	1	1	1	1	1	1	1	1	1	1	1	1	1	1	1	1	1	ygcY	putative glucarate dehydratase
b2789	1	0	1	1	1	1	1	1	1	1	1	1	0	1	1	1	1	1	1	1	1	1	1	1	1	1	1	1	1	1	1	1	1	1	1	1	1	1	1	1	1	1	1	b2789	putative transport protein
b2790	1	1	1	1	1	1	1	1	1	1	1	1	0	1	1	1	1	1	1	1	1	1	1	1	1	1	1	1	1	1	1	1	1	1	1	1	1	1	1	1	1	1	1	b2790	orf, hypothetical protein
b2791	1	1	1	1	1	1	1	1	1	1	1	1	0	1	1	1	1	1	1	1	1	1	1	1	1	1	1	1	1	1	1	1	1	1	1	1	1	1	1	1	1	1	1	yqcB	orf, hypothetical protein
b2792	1	1	1	1	1	1	1	1	1	1	1	1	0	1	1	1	1	1	1	1	1	1	1	1	1	1	1	1	1	1	1	1	1	1	1	1	1	1	1	1	1	1	1	b2792	orf, hypothetical protein
b2793	1	1	1	1	1	1	1	1	1	1	1	1	0	1	1	1	1	1	1	1	1	1	1	1	1	1	1	1	1	1	1	1	1	1	1	1	1	1	1	1	1	1	1	syd	interacts with secY
b2794	1	1	1	1	1	1	1	1	1	1	1	1	0	1	1	1	1	1	1	1	1	1	1	1	1	1	1	1	1	1	1	1	1	1	1	1	1	1	1	1	1	1	1	yqcD	orf, hypothetical protein
b2795	1	1	1	1	1	1	1	1	1	1	1	1	0	1	1	1	1	1	1	1	1	1	1	1	1	1	1	1	1	1	1	1	1	1	1	1	1	1	1	1	1	1	1	ygdH	orf, hypothetical protein
b2796	1	1	1	1	1	1	1	1	1	1	1	1	0	1	1	1	1	1	1	1	1	1	1	1	1	1	1	1	1	1	1	1	1	1	1	1	1	1	1	1	1	1	1	sdaC	probable serine transporter
b2797	1	1	1	1	1	1	1	1	1	1	1	1	0	1	1	1	1	1	1	1	1	1	1	1	1	1	1	1	1	1	1	1	1	1	1	1	1	1	1	1	1	1	1	sdaB	L-serine dehydratase (deaminase), L-SD2
b2798	1	1	1	1	1	1	1	1	1	1	1	1	0	1	1	1	1	1	1	1	1	1	1	1	1	1	1	1	1	1	1	1	1	1	1	1	1	1	1	1	1	1	1	exo	5'-3' exonuclease
b2799	1	1	1	1	1	1	1	1	1	1	1	1	0	1	1	1	0	1	1	1	1	1	1	1	1	1	1	1	1	1	1	1	1	1	1	1	1	1	1	1	1	1	1	fucO	L-1,2-propanediol oxidoreductase

b2800	1	1	1	1	1	1	1	1	1	1	1	1	0	1	1	1	0	1	1	1	1	1	1	1	1	1	1	1	1	1	1	1	1	1	1	1	1	1	1	1	1	1	1	fucA	L-fuculose-1-phosphate aldolase
b2801	1	1	1	1	1	0	0	0	0	0	0	0	1	1	1	1	1	1	1	1	1	1	1	1	1	1	1	1	1	1	1	1	1	1	1	1	1	1	1	1	1	1	1	fucP	fucose permease
b2802	1	1	1	1	1	0	1	1	1	1	1	1	1	1	1	1	1	1	1	1	1	1	1	1	1	1	1	1	1	1	1	1	1	1	1	1	1	1	1	1	1	1	1	fucI	L-fucose isomerase
b2803	1	1	1	1	1	1	1	1	1	1	1	1	1	1	0	1	1	1	1	1	1	1	1	1	1	1	1	1	1	1	1	1	1	1	1	1	1	1	1	1	1	1	1	fucK	L-fuculokinase
b2807	1	1	1	1	1	1	1	1	1	1	1	1	0	1	1	1	1	1	1	1	1	1	1	1	1	1	1	1	1	1	1	1	1	1	1	1	1	1	1	1	1	1	1	ygdD	orf, hypothetical protein
b2809	1	1	1	1	1	1	1	1	1	1	1	1	1	1	0	1	1	1	1	1	1	1	1	1	1	1	1	1	1	1	1	1	1	1	1	1	1	1	1	1	1	1	1	b2809	orf, hypothetical protein
b2811	1	1	1	1	1	0	1	1	1	1	1	1	0	1	0	1	1	1	1	1	1	1	1	1	0	0	0	1	0	0	1	1	1	1	1	1	1	1	1	1	1	1	1	ygdK	orf, hypothetical protein
b2813	1	1	1	1	1	1	1	1	1	1	1	1	1	1	0	1	1	1	1	1	1	1	1	1	1	1	1	1	1	1	1	1	1	1	1	1	1	1	1	1	1	1	1	mltA	membrane-bound lytic murein transglycosylase A
b2820	1	1	1	1	1	1	0	1	1	1	1	1	0	1	1	1	1	0	1	1	1	0	1	1	0	0	1	1	0	0	1	1	1	1	1	1	1	1	1	1	1	1	1	recB	DNA helicase, ATP-dependent dsDNA/ssDNA exonuclease V subunit, ssDNA endonuclease
b2823	1	1	1	1	1	1	1	1	1	1	1	1	1	1	1	1	1	1	1	1	1	1	1	1	1	1	1	1	1	1	1	1	0	1	1	1	1	1	1	1	1	1	1	ppdC	prepilin peptidase dependent protein C
b2825	1	1	1	1	1	1	1	1	1	1	1	1	1	1	1	1	1	1	1	1	1	1	1	1	1	1	1	1	1	1	1	1	1	1	0	1	1	1	1	1	1	1	1	ppdB	prepilin peptidase dependent protein B
b2839	1	1	1	1	1	1	0	0	0	1	1	1	1	1	1	1	1	1	1	1	1	1	1	1	1	1	1	1	1	1	1	1	1	1	1	1	1	1	1	1	1	1	1	lysR	positive regulator for lys
b2841	1	0	1	1	1	1	1	1	1	1	1	1	1	1	1	1	1	1	1	1	1	0	1	1	1	1	1	1	1	1	1	1	1	1	1	1	1	1	1	1	1	1	1	araE	low-affinity L-arabinose transport system proton symport protein
b2842	0	0	1	1	1	1	1	1	1	1	1	1	1	1	1	1	1	1	1	1	1	0	1	1	1	1	1	1	1	1	1	1	1	1	1	1	1	1	1	1	1	1	1	kduD	2-deoxy-D-gluconate 3-dehydrogenase
b2843	0	0	1	1	1	1	1	1	1	1	1	1	1	1	0	1	1	1	1	1	1	0	1	1	1	1	1	0	1	1	1	1	1	1	1	1	1	1	1	1	1	1	1	kduI	homolog of pectin degrading enzyme 5-keto 4-deoxyuronate isomerase
b2844	0	0	1	1	1	1	1	1	1	1	1	1	1	1	1	1	1	1	1	1	1	0	1	1	1	1	1	0	1	1	1	1	1	1	1	1	1	1	1	1	1	1	1	yqeF	putative acyltransferase
b2845	0	0	1	1	1	1	1	1	1	1	1	1	1	1	1	1	1	1	1	1	1	0	1	1	1	1	1	0	1	1	1	1	1	1	1	1	1	1	1	1	1	1	1	b2845	putative transporter protein
b2846	0	0	1	0	1	0	0	0	0	0	0	0	0	0	0	0	0	0	0	0	0	0	0	0	1	1	1	0	1	1	1	0	0	0	0	0	0	0	0	0	0	0	0	yqeH	orf, hypothetical protein
b2847	0	0	1	0	0	0	0	0	0	0	0	0	0	0	0	0	0	0	0	0	0	0	0	0	0	0	0	0	0	0	0	0	0	0	0	0	0	0	0	0	0	0	0	yqeI	putative sensory transducer
b2848	0	1	1	1	1	1	0	0	1	1	1	1	0	0	1	1	1	0	0	1	1	1	1	1	1	1	0	1	1	0	1	1	0	0	0	1	1	1	1	1	1	1	1	yqeJ	orf, hypothetical protein
b2849	0	0	1	0	0	0	0	0	0	0	0	0	0	0	0	0	0	0	0	0	0	0	0	0	0	0	0	0	0	0	0	0	0	0	0	0	0	0	0	0	0	0	0	yqeK	orf, hypothetical protein
b2850	0	0	1	0	0	0	0	0	0	0	0	0	0	0	0	0	0	0	0	0	0	0	0	0	0	0	0	0	0	0	0	0	0	0	0	0	0	0	0	0	0	0	0	ygeF	orf, hypothetical protein
b2851	0	0	1	0	0	0	0	0	0	0	0	0	0	0	0	0	0	0	0	0	0	0	0	0	0	0	0	0	0	0	0	0	0	0	0	0	0	0	0	0	0	0	0	ygeG	orf, hypothetical protein
b2852	0	0	1	0	0	0	0	0	0	0	0	0	0	0	0	0	0	0	0	0	0	0	0	0	0	0	0	0	0	0	0	0	0	0	0	0	0	0	0	0	0	0	0	ygeH	putative invasion protein
b2853	0	0	0	0	0	0	0	0	0	0	0	0	0	0	0	0	0	0	0	0	0	0	0	0	0	0	0	0	0	0	0	0	0	0	0	0	0	0	0	0	0	0	0	b2853	orf, hypothetical protein
b2854	0	0	0	0	0	0	0	0	0	0	0	0	0	0	0	0	0	0	0	0	0	0	0	0	0	0	0	0	0	0	0	0	0	0	0	0	0	0	0	0	0	0	0	b2854	orf, hypothetical protein
b2855	0	0	0	0	0	0	0	0	0	0	0	0	0	0	0	0	0	0	0	0	0	0	0	0	0	0	0	0	0	0	0	0	0	0	0	0	0	0	0	0	0	0	0	ygeK	putative 2-component transcriptional regulator
b2856	0	0	0	0	0	0	0	0	0	0	0	0	0	0	0	0	0	0	0	0	0	0	0	0	0	0	0	0	0	0	0	0	0	0	0	0	0	0	0	0	0	0	0	b2856	orf, hypothetical protein
b2857	0	0	0	0	0	0	0	0	0	0	0	0	0	0	0	0	0	0	0	0	0	0	0	0	0	0	0	0	0	0	0	0	0	0	0	0	0	0	0	0	0	0	0	b2857	orf, hypothetical protein
b2858m	0	0	0	0	0	0	0	0	0	0	0	0	0	0	0	0	0	0	0	0	0	0	0	0	0	0	0	0	0	0	0	0	0	0	0	0	0	0	0	0	0	0	0	ygeN	Uncharacterized protein
b2859	0	0	0	0	0	0	0	0	0	0	0	1	0	0	0	0	0	0	0	0	0	0	0	0	0	0	0	0	0	0	0	1	0	0	0	0	0	0	0	0	0	0	0	b2859	orf, hypothetical protein
b2862	0	0	1	1	0	0	0	0	0	0	0	0	0	0	0	0	0	0	0	0	0	0	0	0	0	0	0	0	0	0	0	1	0	0	0	0	0	0	0	0	0	0	0	b2862	orf, hypothetical protein
b2863	0	0	1	1	1	0	0	0	0	0	0	0	0	0	0	0	0	0	0	0	0	0	0	0	1	1	1	1	1	1	1	1	1	1	1	1	1	1	1	1	1	0	0	b2863	orf, hypothetical protein
b2865	0	1	1	0	1	1	1	1	1	1	1	1	1	1	1	1	1	1	1	1	1	1	1	1	1	1	1	1	1	1	1	0	1	1	1	0	1	1	1	1	1	0	0	b2865	putative lipoprotein
b2866	0	0	1	0	1	0	0	0	0	1	0	0	1	1	1	1	1	1	1	1	1	0	1	1	1	1	1	1	1	1	1	1	1	1	1	1	1	1	1	1	1	0	0	b2866	orf, hypothetical protein
b2867	0	0	1	0	1	0	0	0	0	0	0	0	1	1	1	1	1	1	1	1	1	0	1	1	1	1	1	1	1	1	1	1	1	1	1	1	1	1	1	1	1	0	0	ygeT	putative dehydrogenase
b2868	0	0	1	0	1	0	0	0	0	0	0	0	1	1	1	1	0	1	1	1	1	0	1	1	1	1	1	1	1	1	1	1	1	1	1	1	1	1	1	1	0	0	0	b2868	putative dehydrogenase
b2869	0	0	1	0	1	0	0	0	0	0	0	0	1	1	1	1	0	1	1	1	1	0	1	1	1	1	1	1	1	1	1	1	0	0	0	0	0	0	0	0	0	0	0	ygeV	putative transcriptional regulator
b2870	0	0	1	0	1	0	0	0	0	0	0	0	1	1	0	1	0	0	0	0	0	0	1	0	1	1	1	1	1	1	1	1	0	0	0	0	0	0	0	0	0	0	0	ygeW	putative carbamoyl transferase
b2871	0	1	1	1	1	1	0	1	1	1	1	1	1	1	1	1	1	1	1	1	1	1	1	1	1	1	1	1	1	1	1	1	1	0	0	1	1	1	1	1	1	1	1	ygeX	putative dehydratase
b2872	0	0	1	0	0	0	0	0	0	0	0	0	1	1	1	1	0	0	0	0	0	0	1	0	1	1	1	1	1	1	1	1	0	0	0	0	0	0	0	0	0	0	0	ygeY	putative deacetylase
b2873	0	0	1	0	0	0	0	0	0	0	0	0	1	1	1	1	1	0	0	0	1	1	1	1	1	1	1	1	1	1	1	1	0	0	0	0	0	0	0	0	0	0	0	b2873	orf, hypothetical protein
b2874	0	0	1	0	0	0	0	0	0	0	0	0	1	1	1	1	0	0	0	0	0	0	1	0	1	1	1	1	1	1	1	1	0	0	0	0	0	0	0	0	0	0	0	yqeA	putative kinase
b2875	0	0	1	1	0	0	0	0	0	0	0	0	1	1	1	1	0	0	0	0	0	0	1	0	0	1	1	1	1	1	1	1	0	0	0	0	0	0	0	0	0	0	0	b2875	putative synthases
b2876	0	1	1	1	1	0	0	0	0	1	1	1	0	1	1	0	1	1	1	1	1	1	1	1	1	1	1	1	1	0	1	1	0	0	0	1	1	1	1	1	1	1	1	b2876	orf, hypothetical protein
b2877	0	0	1	1	0	0	0	0	0	0	0	0	1	1	1	1	0	0	0	0	0	0	0	0	1	1	1	1	1	1	1	0	1	0	0	0	0	0	0	0	0	0	0	ygfJ	orf, hypothetical protein
b2878	0	0	1	1	0	0	0	0	0	0	0	0	1	0	0	0	0	0	0	0	0	0	1	1	1	1	1	1	1	1	1	0	1	0	0	0	0	0	0	0	0	0	0	b2878	putative oxidoreductase, Fe-S subunit
b2879	0	0	1	1	0	0	0	0	0	0	0	0	1	0	0	0	0	0	0	0	0	0	1	1	1	1	1	1	1	1	1	0	1	0	0	0	0	0	0	0	0	0	0	b2879	putative proteoglycan
b2880	0	0	1	1	0	0	0	0	0	0	0	0	1	0	0	0	0	0	0	0	1	0	1	1	1	1	1	1	1	1	1	1	1	0	0	0	0	0	0	0	0	0	0	b2880	orf, hypothetical protein
b2881	0	0	1	1	0	0	0	0	0	0	0	0	1	0	0	0	0	0	0	0	0	0	1	1	1	1	1	1	1	1	1	1	1	0	0	0	0	0	0	0	0	0	0	b2881	putative dehydrogenase
b2883	0	0	0	1	0	0	0	0	0	0	0	0	1	0	0	0	0	1	1	1	0	0	1	1	1	1	1	1	1	1	1	0	1	0	0	0	0	0	0	0	0	1	1	ygfP	orf, hypothetical protein
b2884	1	0	1	1	0	0	0	0	0	0	0	0	1	0	0	1	0	1	1	1	0	0	1	1	1	1	1	1	1	1	1	0	1	1	1	0	0	0	0	0	0	1	1	ygfQ	orf, hypothetical protein
b2885	1	0	1	1	0	0	0	0	0	0	0	0	1	0	0	0	0	1	1	1	0	0	1	1	1	1	1	1	1	1	1	0	1	0	0	0	0	0	0	0	0	1	1	ygfR	putative oxidoreductase
b2886	0	0	1	1	0	0	0	0	0	0	0	0	1	0	0	0	0	1	1	1	0	0	1	1	1	1	1	1	1	1	1	0	1	0	0	0	0	0	0	0	0	1	1	ygfS	putative oxidoreductase, Fe-S subunit
b2887	0	0	1	1	0	0	0	0	0	0	0	0	1	0	0	0	0	1	1	1	0	0	1	1	1	1	1	1	1	1	1	0	1	0	0	0	0	0	0	0	0	1	1	ygfT	putative oxidoreductase, Fe-S subunit
b2888	0	1	1	1	1	0	0	0	0	0	0	0	1	1	1	1	1	1	1	1	1	1	1	1	1	1	1	1	1	1	1	1	1	1	1	1	1	1	1	1	1	1	1	ygfU	putative permease
b2890	1	1	1	1	1	1	1	1	1	1	1	1	1	1	1	1	1	1	1	1	1	1	1	1	1	1	1	1	1	1	1	1	0	1	1	1	1	1	1	1	1	1	1	lysS	lysine tRNA synthetase, constitutive; suppressor of ColE1 mutation in primer RNA
b2893	1	1	1	1	1	1	0	1	0	1	1	1	1	1	1	1	1	1	1	1	1	1	1	1	1	1	1	1	1	1	1	1	1	1	1	1	1	1	1	1	1	1	1	dsbC	protein disulfide isomerase II
b2902	1	0	1	0	1	1	1	1	1	1	1	1	1	1	0	1	1	1	1	1	1	1	1	1	1	1	1	1	1	1	1	1	1	1	1	1	1	1	1	1	1	1	1	ygfF	putative oxidoreductase
b2906	1	1	1	1	1	1	1	1	1	1	1	1	1	1	0	1	1	1	1	1	1	1	1	1	1	1	1	1	1	1	1	1	0	1	1	1	1	1	1	1	1	1	1	visC	orf, hypothetical protein
b2907	1	1	1	1	1	0	0	0	1	1	1	1	1	1	0	1	1	0	1	1	1	1	1	1	1	1	1	1	1	1	1	1	1	1	1	1	1	1	1	1	0	1	1	ubiH	2-octaprenyl-6-methoxyphenol--> 2-octaprenyl-6-methoxy-1, 4-benzoquinone
b2908	1	1	1	1	1	1	1	1	1	1	1	1	1	1	1	1	1	1	1	1	1	1	1	1	1	0	0	1	1	1	1	1	0	1	1	1	1	1	1	1	1	1	1	pepP	proline aminopeptidase P II
b2910	1	1	1	1	1	1	1	1	1	1	1	1	1	1	0	1	1	1	1	1	1	1	1	1	1	1	1	1	1	1	1	1	1	1	1	1	1	1	1	1	1	1	1	ygfE	orf, hypothetical protein
b2912	1	1	1	1	1	1	1	1	1	1	1	1	1	1	0	1	1	1	1	1	1	1	1	1	1	1	1	1	1	1	1	1	1	1	1	1	1	1	1	1	1	1	1	ygfA	putative ligase
b2915	1	1	1	1	1	1	0	0	0	1	1	1	1	1	0	1	1	1	1	1	1	1	1	1	0	0	1	1	1	0	1	1	0	1	1	1	1	1	1	1	1	1	1	yqfE	orf, hypothetical protein
b2917	1	0	1	1	1	1	1	1	1	1	1	1	1	1	1	1	1	1	1	1	1	1	1	1	1	1	1	1	1	1	1	1	1	1	1	1	1	1	1	1	1	1	1	sbm	methylmalonyl-CoA mutase (MCM)
b2918	1	0	1	1	1	1	1	1	1	1	1	1	1	1	1	1	1	1	1	1	1	1	1	1	1	1	1	1	1	1	1	1	1	1	1	1	1	1	1	1	1	1	1	ygfD	putative nucleotide-binding protein
b2919	0	0	1	1	1	1	1	1	1	1	1	1	1	1	1	1	1	1	1	1	0	1	1	1	1	1	1	1	1	1	1	1	1	1	1	1	1	1	1	1	1	1	1	ygfG	putative enzyme
b2920	0	0	1	1	1	1	1	1	1	1	1	1	1	1	1	1	1	1	1	1	0	1	1	1	1	1	1	1	1	1	1	1	1	1	1	1	1	1	1	1	1	1	1	ygfH	putative coenzyme A transferase
b2921	0	1	1	1	1	1	1	1	1	1	1	1	1	1	1	1	1	1	1	1	0	1	1	1	1	1	1	1	1	1	1	1	1	1	1	1	1	1	1	1	1	1	1	ygfI	putative transcriptional regulator LYSR-type
b2924	1	1	1	1	1	1	1	1	1	1	1	1	1	1	1	1	1	1	1	1	1	1	1	1	1	1	0	1	1	1	1	1	1	1	1	1	1	1	1	1	1	1	1	yggB	putative transport protein
b2927	1	1	1	1	1	1	1	1	1	1	1	1	1	1	0	1	1	1	1	1	1	1	1	1	1	1	1	1	1	1	1	1	1	1	1	1	1	1	1	1	1	1	1	epd	D-erythrose 4-phosphate dehydrogenase
b2928	0	1	0	1	1	1	1	1	0	1	1	1	1	0	1	1	1	0	0	1	1	1	1	1	1	0	1	0	1	1	1	1	1	0	0	0	0	0	1	0	0	0	0	yggC	putative kinase
b2929	1	1	0	1	1	1	1	1	1	1	1	1	1	1	0	1	1	1	1	1	1	1	1	1	1	1	1	1	1	1	1	1	0	1	1	1	1	1	1	1	1	1	1	yggD	putative transcriptional regulator
b2930	1	1	0	1	1	1	1	1	1	1	1	1	1	1	1	1	1	1	1	1	1	1	1	1	1	0	0	0	0	0	0	1	1	1	1	1	1	1	1	1	1	1	1	yggF	orf, hypothetical protein
b2931	1	1	0	1	1	1	1	1	1	1	1	1	1	1	0	1	1	1	1	1	1	1	1	1	1	1	1	1	1	1	1	1	1	1	1	1	1	1	1	1	1	1	1	b2931	putative oxidoreductase
b2932	1	1	0	1	1	1	1	1	1	1	1	1	1	1	1	1	1	1	1	1	1	1	1	1	1	1	1	1	1	1	1	1	1	1	1	1	1	1	1	1	1	1	1	yggP	orf, hypothetical protein
b2933	1	1	1	1	1	1	1	1	1	1	1	1	1	1	1	1	1	1	1	1	1	1	1	1	1	1	1	1	1	1	1	0	0	0	0	0	0	0	0	0	0	1	1	cmtA	PTS system, mannitol-specific enzyme II component, cryptic
b2934	1	1	1	1	1	1	1	1	1	1	1	1	1	1	1	1	1	1	1	1	1	1	1	1	1	1	1	1	1	1	1	1	0	0	0	0	0	0	0	0	0	1	1	cmtB	PTS system, mannitol-specific enzyme II component, cryptic
b2936	1	1	1	1	1	1	1	1	1	1	1	1	1	1	1	1	1	1	1	1	1	1	1	1	1	1	0	1	1	0	1	1	1	1	1	1	1	1	1	1	1	1	1	yggG	orf, hypothetical protein
b2945	1	1	1	1	1	1	1	1	1	1	1	1	1	1	1	0	1	1	1	1	1	1	1	1	0	1	0	0	1	0	1	1	1	1	1	1	1	1	1	1	1	0	1	endA	DNA-specific endonuclease I
b2954	1	1	1	1	1	0	0	0	0	1	1	1	1	1	1	1	1	1	1	1	1	1	1	1	1	1	1	1	1	1	1	1	1	1	1	1	1	1	1	1	1	1	1	yggV	putative ribosomal protein
b2957	1	1	1	1	1	1	1	0	0	1	1	1	0	1	1	1	1	1	1	1	1	1	1	1	1	1	0	1	1	1	1	1	1	1	1	1	1	1	1	1	0	1	1	ansB	periplasmic L-asparaginase II
b2968	1	1	0	1	1	1	1	1	1	1	1	1	1	1	1	1	1	1	1	1	1	1	1	1	0	0	1	1	1	0	0	0	0	0	0	0	0	0	0	0	0	0	0	yghD	putative secretion pathway protein
b2969	1	1	0	1	1	1	1	1	1	1	1	1	1	1	1	1	1	1	1	1	1	1	1	1	0	0	1	1	1	0	0	0	0	0	0	0	0	0	0	0	0	0	0	yghE	putative general secretion pathway for protein export (GSP)
b2970				1	1	1	1	1	1	0	1	1	1		1	1	1	1	1	1	1	1	1	1	0	0	1	1	1	0	0	0	0	0		0	0	0	0	0	0	0	0	b2970	putative general secretion pathway for protein export (GSP)
b2971	0	1	0	0	0	0	0	0	0	0	0	0	0	0	0	0	0	0	0	0	0	0	0	0	0	0	1	1	1	0	0	0	0	0	0	0	0	0	0	0	0	0	0	b2971	orf, hypothetical protein
b2972	0	1	0	0	0	0	0	0	0	0	0	0	0	0	0	0	0	0	0	0	0	0	0	0	0	0	1	1	1	0	0	0	0	0	0	0	0	0	0	0	0	0	0	b2972	putative peptidase
b2973	0	1	0	0	0	0	0	0	0	0	0	0	0	0	0	0	0	0	0	0	0	0	0	0	0	0	1	1	1	0	0	0	0	0	0	0	0	0	0	0	0	0	0	b2973	orf, hypothetical protein
b2974	0	1	0	0	0	0	0	0	0	0	0	0	0	0	0	0	0	0	0	0	0	0	0	0	0	0	1	1	1	0	0	0	0	0	0	0	0	0	0	0	0	0	0	b2974	putative endoglucanase
b2975	1	1	1	0	1	1	0	1	0	1	0	0	0	1	0	0	0	0	0	0	0	1	1	1	0	1	1	1	1	0	0	1	0	1	1	1	1	1	1	1	1	0	0	yghK	putative permease
b2976	0	1	0	0	0	0	0	0	0	0	0	0	0	0	0	0	0	0	0	0	0	0	0	0	0	1	1	1	1	1	0	1	0	1	1	1	1	1	1	1	1	1	1	glcB	malate synthase G
b2977	0	1	0	1	0	1	1	1	1	1	1	1	1	0	1	1	1	1	1	1	1	1	1	1	1	1	1	1	1	1	1	1	1	1	1	1	1	1	1	1	1	1	1	glcG	orf, hypothetical protein
b2978	0	1	0	0	0	0	0	0	0	0	0	0	0	0	0	0	0	0	0	0	0	0	0	0	0	1	1	1	1	0	0	0	0	1	1	1	1	1	1	1	1	1	1	glcF	glycolate oxidase iron-sulfur subunit
b2979	0	1	0	0	0	0	0	0	0	0	0	0	0	0	0	0	0	0	0	0	0	0	0	0	0	1	1	1	1	1	0	1	0	1	1	1	1	1	1	1	1	1	1	glcD	glycolate oxidase subunit D
b2980	0	1	0	1	1	1	1	1	1	1	1	1	1	0	0	1	1	1	0	1	1	1	1	1	1	1	1	1	1	1	1	1	1	1	1	1	1	1	1	1	1	1	1	glcC	transcriptional activator for glc operon
b2981	0	1	0	0	0	0	0	0	0	0	0	0	0	0	0	0	0	0	0	0	0	0	0	0	1	1	1	0	1	1	0	1	0	1	1	1	1	1	1	1	1	1	1	b2981	orf, hypothetical protein
b2983	0	1	1	1	0	0	0	0	0	0	0	0	0	0	0	0	0	0	0	0	0	0	0	0	1	1	1	1	1	1	0	1	0	0	1	1	1	0	1	0	1	1	1	yghQ	orf, hypothetical protein
b2984	0	1	1	1	0	0	0	0	0	0	0	0	0	0	0	0	0	0	0	0	0	0	0	0	1	1	1	1	1	1	0	1	0	0	1	1	1	0	1	1	1	1	1	yghR	orf, hypothetical protein
b2985	0	1	1	1	0	0	0	0	0	0	0	0	0	0	0	0	0	0	0	0	0	0	0	0	1	1	1	1	1	1	0	1	0	0	1	1	1	0	1	1	1	1	1	yghS	orf, hypothetical protein
b2986	1	1	1	1	1	0	0	0	0	0	0	0	0	0	0	0	0	0	0	0	0	0	0	0	1	1	1	1	1	1	0	1	1	0	1	1	1	1	1	1	1	1	1	yghT	orf, hypothetical protein
b2987	1	1	1	1	1	0	0	0	0	0	0	0	0	1	0	0	0	0	0	0	0	0	0	0	1	1	1	1	1	1	1	1	1	1	1	1	1	1	1	1	1	1	1	pitB	low-affinity phosphate transport
b2991	1	1	1	1	1	0	1	1	1	1	1	1	1	1	1	1	1	1	1	1	1	1	1	1	1	1	1	1	1	1	1	1	1	1	1	1	1	1	1	1	1	1	1	hybF	may modulate levels of hydrogenease-2
b2994	1	1	1	1	1	1	1	1	1	1	1	1	1	1	1	1	1	1	1	1	1	1	1	1	1	1	1	1	1	1	1	1	1	1	1	1	1	1	1	0	1	1	1	hybC	probable large subunit, hydrogenase-2
b2999m	1	0	1	1	1	1	1	1	1	1	1	1	1	1	1	1	1	1	1	1	1	1	1	1	1	1	1	1	1	1	1	1	1	1	1	1	1	1	1	1	1	1	1	yghYX	Predicted hydrolase
b3002	1	0	1	1	1	1	1	1	1	1	1	1	1	1	1	1	1	1	1	1	1	1	1	1	1	1	1	1	1	1	1	1	1	1	1	1	1	1	1	1	1	1	1	yqhA	orf, hypothetical protein

b3003	1	0	1	1	1	1	1	1	1	1	1	1	1	1	1	1	1	1	1	1	1	1	1	1	1	1	1	1	1	1	1	1	1	1	1	1	1	1	1	1	1	1	1	yghA	putative oxidoreductase
b3004	1	1	1	1	1	1	1	1	1	1	1	1	1	1	1	1	1	1	1	1	1	1	1	1	1	1	1	1	1	1	1	1	0	1	1	1	1	1	1	1	1	1	1	b3004	orf, hypothetical protein
b3011	1	1	1	1	1	1	1	0	1	1	1	1	1	1	1	1	1	1	1	1	1	1	1	1	1	1	1	1	1	1	1	1	1	1	1	1	1	1	1	1	1	1	1	yqhD	putative oxidoreductase
b3012	1	1	1	1	1	1	1	0	1	1	1	1	1	1	1	1	1	1	1	1	1	1	1	1	1	1	1	1	1	1	1	1	1	1	1	1	1	1	1	1	1	1	1	yqhE	orf, hypothetical protein
b3013	1	1	1	1	1	1	1	0	1	1	1	1	1	1	1	1	1	1	1	1	1	1	1	1	1	1	1	1	1	1	1	1	1	1	1	1	1	1	1	1	1	1	1	yqhG	orf, hypothetical protein
b3014	1	1	1	1	1	1	1	0	1	1	1	1	1	1	1	1	1	1	1	1	1	1	1	1	1	1	1	1	1	1	1	1	1	1	1	1	1	1	1	1	1	1	1	yqhH	orf, hypothetical protein
b3015m	1	1	1	1	1	1	1	1	1	1	1	1	1	1	1	1	1	1	1	1	1	1	1	1	1	1	0	1	0	0	0	1	0	1	1	1	1	1	1	1	1	1	1	ygiRQ	Fe-S oxidoreductase
b3017	1	0	1	1	1	1	1	1	1	1	1	1	1	1	1	1	1	1	1	1	1	1	1	1	1	1	1	0	1	1	1	1	1	1	1	1	1	1	1	1	1	1	1	sufI	suppressor of ftsI
b3020	1	1	0	1	0	0	0	0	0	0	0	0	0	0	0	0	0	0	0	0	0	0	0	0	1	1	1	1	1	1	1	1	0	0	0	0	0	0	0	0	0	0	0	b3020	putative transport periplasmic protein
b3021	0	0	0	0	0	0	0	0	0	0	0	0	0	0	0	0	0	0	0	0	0	0	0	0	0	0	0	0	0	0	0	0	0	0	0	0	0	0	0	0	0	0	0	b3021	orf, hypothetical protein
b3022	0	0	0	0	0	0	0	0	0	0	0	0	0	0	0	0	0	0	0	0	0	0	0	0	0	0	0	0	0	0	0	0	0	0	0	0	0	0	0	0	0	0	0	b3022	orf, hypothetical protein
b3027	0	0	0	0	0	0	0	0	0	0	0	0	0	0	0	0	1	0	0	0	0	0	0	0	1	1	1	1	1	1	1	1	1	1	1	1	1	1	1	1	1	1	1	b3027	orf, hypothetical protein
b3028	1	1	1	1	1	1	1	0	1	1	1	1	1	1	1	1	1	1	1	1	1	1	1	1	1	1	1	1	1	1	1	1	1	1	1	1	1	1	1	1	1	1	1	mdaB	modulator of drug activity B
b3043	0	0	1	0	0	1	1	1	1	1	1	1	0	1	1	1	0	0	1	1	1	1	1	1	0	1	1	0	1	1	0	1	0	0	0	0	0	0	0	0	1	1	1	ygiL	putative fimbrial-like protein
b3046	0	0	1	0	0	0	0	0	0	0	0	0	0	0	0	0	0	0	0	0	0	0	0	0	1	1	1	1	1	1	1	1	0	0	0	0	0	0	0	0	0	1	1	yqiG	putative membrane protein
b3047	0	0	1	0	0	0	0	0	0	0	0	0	0	0	0	0	1	0	0	0	0	0	0	0	1	1	1	1	1	1	1	1	0	0	0	0	0	0	0	0	0	1	1	yqiH	putative membrane protein
b3048	0	0	1	0	0	0	0	0	0	0	0	0	0	0	0	0	0	0	0	0	0	0	0	0	1	1	1	1	0	1	1	1	0	0	0	0	0	0	0	0	1	1	1	yqiI	orf, hypothetical protein
b3049	1	1	1	0	1	0	0	0	0	0	0	0	0	0	0	0	0	0	0	0	0	0	0	0	1	1	1	1	1	1	1	1	1	0	1	0	1	1	1	1	1	1	1	glgS	glycogen biosynthesis, rpoS dependent
b3050	0	1	1	1	1	0	0	0	0	0	0	0	0	0	0	0	0	0	0	0	1	0	0	0	1	1	1	1	1	1	1	1	0	0	0	0	0	0	0	0	0	1	1	b3050	putative oxidoreductase
b3051	1	1	1	1	1	1	1	1	1	1	1	1	0	1	1	1	1	0	1	1	1	1	1	1	1	1	1	1	1	1	1	1	0	0	0	0	0	0	0	0	0	1	1	b3051	putative membrane protein
b3052	1	1	1	1	1	1	1	0	1	1	1	1	1	1	1	1	1	1	1	1	1	1	1	1	1	1	1	1	1	1	1	1	1	1	1	1	1	1	1	1	1	1	1	b3052	putative kinase
b3054	1	1	1	1	1	1	1	1	1	1	1	1	1	1	1	1	1	1	1	1	1	1	1	1	1	0	0	1	0	0	1	1	1	1	1	1	1	1	1	1	1	1	1	ygiF	orf, hypothetical protein
b3072	1	0	1	1	1	1	0	0	1	1	1	1	0	1	1	1	0	1	1	1	1	1	1	1	1	1	1	1	1	1	1	1	1	1	1	1	1	1	1	1	1	1	1	aer	aerotaxis sensor receptor, flavoprotein
b3074	1	1	1	1	1	0	0	0	0	0	0	0	1	1	1	1	0	1	1	1	1	1	1	1	1	1	1	1	1	1	1	1	1	1	1	1	1	1	1	1	1	1	1	ygjH	putative tRNA synthetase
b3075	1	1	1	1	1	0	0	0	0	0	0	0	1	1	1	1	0	1	1	1	1	1	1	1	1	1	0	1	1	1	1	1	1	1	1	1	1	1	1	1	1	1	1	ebgR	regulator of ebg operon
b3076	1	1	1	1	1	0	0	0	0	0	0	0	1	1	1	1	0	1	1	1	1	1	1	1	1	1	0	1	1	1	1	1	1	1	1	1	1	1	1	1	1	1	1	ebgA	evolved beta-D-galactosidase, alpha subunit; cryptic gene
b3077	0	1	1	1	1	1	0	0	1	1	1	1	1	1	1	1	0	1	1	1	1	1	1	1	1	1	0	1	1	1	1	1	1	1	1	1	1	1	1	1	1	1	1	ebgC	evolved beta-D-galactosidase, beta subunit; cryptic gene
b3078	0	1	1	1	0	1	1	1	1	1	1	1	1	1	1	1	0	0	0	0	0	0	0	0	1	1	0	1	1	1	1	1	1	1	1	1	1	1	1	1	1	1	1	ygjI	putative oxidoreductase
b3079	1	1	1	1	1	0	1	1	1	1	1	1	0	1	1	1	1	1	1	1	1	1	1	1	1	1	1	1	1	1	1	1	1	1	1	1	1	1	1	1	1	1	1	ygjJ	orf, hypothetical protein
b3080	1	1	1	1	1	1	1	1	1	1	1	1	1	1	1	1	1	1	1	1	1	1	1	1	1	1	0	1	0	1	1	1	1	1	1	1	1	1	1	1	1	1	1	ygjK	putative isomerase
b3081	1	1	1	0	1	1	1	1	1	1	1	1	1	1	1	1	1	1	1	1	1	1	1	1	1	1	0	0	0	1	1	1	0	1	1	1	1	1	1	1	1	1	1	ygjL	putative NADPH dehydrogenase
b3082	1	1	1	1	1	1	1	1	1	1	1	1	1	1	1	1	1	1	1	1	1	1	1	1	1	1	1	1	0	1	1	1	1	1	1	1	1	1	1	1	1	1	1	ygjM	orf, hypothetical protein
b3083	0	1	1	1	1	1	1	1	1	1	1	1	1	1	1	1	1	1	1	1	1	1	1	1	1	1	1	1	0	1	1	1	1	1	1	1	1	1	1	1	1	1	1	ygjN	orf, hypothetical protein
b3086	1	1	1	1	1	1	1	1	1	1	1	1	1	1	1	1	1	1	1	1	1	1	1	1	1	1	1	1	0	1	1	1	1	1	1	1	1	1	1	1	1	1	1	ygjQ	orf, hypothetical protein
b3101	1	1	1	1	1	1	0	0	1	1	1	1	1	1	1	1	1	1	1	1	1	1	1	1	1	1	1	1	1	1	1	1	0	1	1	1	1	1	1	1	1	1	1	yqjF	orf, hypothetical protein
b3104	1	1	1	1	1	1	1	1	1	1	1	1	0	0	1	1	1	1	1	1	1	1	1	1	0	0	1	0	0	0	0	0	0	0	0	1	1	0	0	0	0	0	0	yhaI	putative cytochrome
b3108m	1	1	1	1	1	1	1	1	1	1	1	1	1	1	1	1	1	1	1	1	1	1	1	1	1	1	1	1	1	1	0	1	1	1	1	1	1	1	1	1	1	1	1	yhaNM	Uncharacterized conserved protein
b3110	1	1	1	1	1	1	1	1	1	1	1	1	1	1	1	1	1	1	1	1	1	1	1	1	1	1	1	1	1	1	1	0	1	1	1	1	1	1	1	1	1	1	1	yhaO	putative transport system permease protein
b3111	1	1	1	1	1	1	1	1	1	1	1	1	1	1	1	1	1	1	1	1	1	1	1	1	1	1	1	1	1	1	1	0	1	1	1	1	1	1	1	1	1	0	0	yhaP	putative L-serine dehydratase
b3112	1	1	1	1	1	1	1	1	1	1	1	1	1	1	1	1	1	1	1	1	1	1	1	1	1	1	1	1	1	1	1	0	1	1	1	1	1	1	1	1	1	0	0	yhaQ	putative L-serine dehydratase
b3113	1	1	1	1	1	1	1	1	1	1	1	1	1	1	1	1	1	1	1	1	1	1	1	1	1	1	1	1	1	1	1	0	1	1	1	1	1	1	1	1	1	0	0	yhaR	orf, hypothetical protein
b3114	1	1	1	1	1	1	1	1	1	1	1	1	1	1	1	1	1	1	1	1	1	1	1	1	1	1	1	1	1	1	1	0	1	1	1	1	1	1	1	1	1	0	0	tdcE	probable formate acetyltransferase 3
b3115	1	0	1	1	1	1	1	1	1	1	1	1	1	1	1	1	1	1	1	1	1	1	1	1	1	1	1	1	1	1	1	0	1	1	1	1	1	1	1	1	1	0	0	tdcD	putative kinase
b3116	1	1	1	1	1	1	1	0	1	1	1	1	1	1	1	1	1	1	1	1	1	1	1	1	1	1	1	1	0	1	0	1	1	1	1	1	1	1	1	1	1	1	1	tdcC	anaerobically inducible L-threonine, L-serine permease
b3117	1	0	1	1	1	1	1	1	1	1	1	1	1	1	1	1	1	1	1	1	1	1	1	1	1	1	1	1	1	1	1	1	1	1	1	1	1	1	1	1	1	1	1	tdcB	threonine dehydratase, catabolic
b3118	1	0	1	1	1	1	1	1	1	1	1	1	1	1	1	1	1	1	1	1	1	1	1	1	1	1	1	1	1	1	1	1	1	1	1	1	1	1	1	1	1	0	0	tdcA	transcriptional activator of tdc operon
b3119	1	0	1	1	1	1	1	1	1	1	1	1	1	1	1	1	1	1	1	1	1	1	1	1	1	1	1	1	1	1	1	1	1	1	1	1	1	1	1	1	1	0	0	tdcR	threonine dehydratase operon activator protein
b3120	1	0	0	1	1	1	1	1	1	1	1	1	1	1	1	1	1	1	1	1	1	1	1	1	1	1	1	1	1	1	1	1	1	1	1	1	1	1	1	1	1	0	0	yhaB	orf, hypothetical protein
b3121	1	0	1	1	1	1	1	1	1	1	1	1	1	1	1	1	1	1	1	1	1	1	1	1	0	1	1	1	1	0	0	0	1	1	1	1	1	1	1	1	1	0	0	yhaC	orf, hypothetical protein
b3125	1	1	1	1	1	1	1	1	1	1	1	1	1	1	1	1	1	1	1	1	0	1	1	1	1	1	1	1	1	1	1	1	1	1	1	1	1	1	1	1	1	1	1	yhaE	putative dehydrogenase
b3126	1	1	1	1	1	1	1	1	1	1	1	1	1	1	1	1	1	1	1	1	0	1	1	1	1	1	1	1	1	1	1	1	1	1	1	1	1	1	1	1	1	1	1	yhaF	orf, hypothetical protein
b3127	1	1	1	1	1	1	1	1	1	1	1	1	1	1	1	1	1	1	1	1	0	1	1	1	1	1	1	1	1	1	1	1	1	1	1	1	1	1	1	1	1	1	1	yhaU	putative transport protein
b3128	0	1	1	1	1	0	0	0	0	0	0	0	0	0	0	0	0	0	0	0	0	0	0	0	1	1	1	1	1	1	1	1	1	0	1	1	1	1	1	1	1	0	0	yhaG	putative hydrolase
b3129	0	1	1	1	1	1	0	0	0	0	0	0	0	0	0	0	0	0	0	0	0	0	0	0	1	1	1	1	1	1	1	1	0	0	0	0	0	0	0	0	0	0	0	sohA	putative protease; htrA suppressor protein
b3130	0	1	1	1	1	0	0	0	0	0	0	0	0	0	0	0	0	0	0	0	0	0	0	0	1	1	1	1	1	1	1	1	0	0	0	0	0	0	0	0	0	0	0	yhaV	orf, hypothetical protein
b3131	0	1	0	1	1	1	0	0	0	0	0	0	0	0	0	0	1	0	0	0	0	0	0	0	1	1	1	1	1	1	1	1	1	0	1	0	0	0	0	0	0	0	0	agaR	putative DEOR-type transcriptional regulator of aga operon
b3132	0	1	0	1	1	0	0	0	0	0	0	0	0	0	0	0	0	0	0	0	0	0	0	0	1	1	1	1	1	1	1	1	1	0	1	1	1	1	1	1	0	0	0	agaZ	putative tagatose 6-phosphate kinase 2
b3133	0	1	0	1	1	0	0	0	0	0	0	0	0	0	0	0	0	0	0	0	0	0	0	0	1	1	1	1	1	1	1	1	0	0	1	1	1	1	1	1	1	0	0	agaV	PTS system, cytoplasmic, N-acetylgalactosamine-specific IIB component 2 (EIIB-AGA)
b3134	0	1	0	1	1	0	0	0	0	0	0	0	0	0	0	0	0	0	0	0	0	0	0	0	1	1	1	1	1	1	1	1	0	0	1	1	1	1	1	1	1	0	0	agaW	PTS system N-acetylgalactosameine-specific IIC component 2
b3135	0	1	0	1	1	1	0	0	0	0	1	1	0	0	0	0	1	0	0	0	1	0	0	1	1	1	1	1	1	1	1	1	0	0	0	0	0	0	0	0	0	0	0	agaA	putative N-acetylgalactosamine-6-phosphate deacetylase
b3136	0	1	0	1	1	0	0	0	0	0	0	0	0	0	0	0	1	0	0	0	1	1	1	1	1	1	1	1	1	1	1	1	0	0	0	0	0	0	0	0	0	0	0	agaS	putative tagatose-6-phosphate aldose/ketose isomerase
b3137	0	1	0	1	1	0	0	0	0	0	0	0	0	0	0	0	0	0	0	0	0	0	0	0	1	1	1	1	1	1	1	1	0	0	0	0	0	0	0	0	0	0	0	agaY	tagatose-bisphosphate aldolase 2
b3138	0	1	0	1	0	0	0	0	0	0	0	0	0	0	0	0	0	0	0	0	0	0	0	0	1	1	1	1	1	1	1	1	1	0	1	1	1	1	0	1	0	0	0	agaB	PTS system, cytoplasmic, N-acetylgalactosamine-specific IIB component 1 (EIIB-AGA)
b3139	0	1	0	1	0	0	0	0	0	0	0	0	0	0	0	0	0	0	0	0	0	0	0	0	1	1	1	1	1	1	1	1	1	0	1	1	1	1	0	1	0	0	0	agaC	PTS system N-acetylgalactosamine-specific IIC component 1
b3140	0	1	0	1	0	0	0	0	0	0	0	0	0	0	0	0	0	0	0	0	0	0	0	0	1	1	1	1	1	1	1	1	1	0	1	1	1	1	0	1	0	0	0	agaD	PTS system, N-acetylglucosamine enzyme IID component 1
b3141	0	1	0	1	0	0	0	0	0	0	0	0	0	0	0	0	0	0	0	0	0	0	0	0	1	1	1	1	1	1	1	1	1	0	1	1	1	1	0	1	0	0	0	agaI	putative galactosamine-6-phosphate isomerase
b3142	0	0	0	0	0	0	0	0	0	0	0	0	0	0	0	0	0	0	0	0	0	0	0	0	1	1	1	1	1	1	1	0	0	1	1	1	1	1	0	1	0	0	0	yraH	putative fimbrial-like protein
b3143	0	0	0	0	1	1	0	0	0	0	0	0	0	0	0	0	0	0	0	0	1	1	1	1	1	1	1	1	1	1	1	0	1	1	1	1	1	1	0	1	0	1	1	yraI	putative chaperone
b3144	0	0	1	0	1	1	1	1	1	1	1	1	1	1	0	1	0	1	1	1	1	1	1	1	1	1	1	1	1	1	1	1	1	1	1	1	1	1	1	1	0	1	1	yraJ	putative outer membrane protein
b3145	1	0	1	0	1	1	1	1	1	1	1	1	1	1	1	1	1	1	1	1	1	1	1	1	1	1	1	1	1	1	1	1	1	1	1	1	1	1	1	1	0	1	1	yraK	putative fimbrial protein
b3173	1	0	1	1	1	1	1	1	1	1	1	1	1	1	1	1	1	1	1	1	1	1	1	1	0	0	0	0	0	0	0	0	1	1	1	1	1	1	1	1	1	1	1	yhbX	putative alkaline phosphatase I
b3175	1	1	1	1	1	1	0	1	0	1	1	1	0	1	1	1	1	1	1	1	1	1	1	1	1	0	0	0	1	0	0	1	1	1	1	1	1	1	1	1	1	1	1	secG	protein export - membrane protein
b3201	1	1	1	1	1	0	0	0	0	1	1	1	1	1	1	0	1	0	1	1	1	1	1	1	1	0	0	1	1	1	1	1	0	1	1	1	1	1	1	1	1	1	1	yhbG	putative ATP-binding component of a transport system
b3214	0	0	0	0	0	0	0	0	0	0	0	0	0	0	0	0	0	0	0	0	0	0	0	0	0	0	0	0	0	0	0	0	0	0	0	0	0	0	0	0	0	0	0	gltF	regulator of gltBDF operon, induction of Ntr enzymes
b3215	0	0	0	0	0	0	0	0	0	0	0	0	0	0	0	0	0	0	0	0	0	0	0	0	0	0	0	0	0	0	0	0	0	0	0	0	0	0	0	0	0	0	0	yhcA	putative chaperone
b3216	0	0	0	0	0	0	0	0	0	0	0	0	0	0	0	0	0	0	0	0	0	0	0	0	0	0	0	0	0	0	0	0	0	0	0	0	0	0	0	0	0	0	0	yhcD	putative outer membrane protein
b3217	0	0	0	0	0	0	0	0	0	0	0	0	0	0	0	0	0	0	0	0	0	0	0	0	0	0	0	0	0	0	0	0	0	0	0	0	0	0	0	0	0	0	0	yhcE	orf, hypothetical protein
b3219	0	0	0	0	0	0	0	0	0	0	0	0	0	0	0	0	0	0	0	0	0	0	0	0	0	0	0	0	0	0	0	0	0	0	0	0	0	0	0	0	0	0	0	yhcF	putative transcriptional regulator
b3220	1	1	1	1	1	0	1	1	0	1	1	1	0	1	1	0	0	1	1	1	1	0	1	1	1	1	1	1	1	1	1	1	1	1	1	1	1	1	1	1	1	1	1	yhcG	orf, hypothetical protein
b3221	1	1	1	1	1	1	1	1	1	1	1	1	1	1	1	1	1	1	1	1	1	1	1	1	1	1	1	1	1	1	1	1	1	1	1	1	1	1	1	1	0	1	1	yhcH	orf, hypothetical protein
b3227	0	1	1	1	1	1	1	1	1	1	1	1	1	1	1	1	1	1	1	1	1	1	1	1	1	1	1	1	1	1	1	1	1	1	1	1	1	1	1	1	1	1	1	yhcL	putative transport protein
b3232	1	1	1	1	1	1	1	1	1	1	1	1	1	1	1	1	1	1	1	1	1	1	1	1	1	1	1	1	1	1	1	1	1	1	1	0	1	1	1	1	1	1	1	yhcM	orf, hypothetical protein
b3238	1	1	1	1	1	1	1	1	0	1	1	1	1	1	1	0	1	1	1	1	1	1	1	1	1	0	1	1	0	1	0	1	0	1	1	1	1	1	1	1	0	1	1	yhcN	orf, hypothetical protein
b3240	1	1	1	1	1	1	1	1	1	1	1	1	1	1	1	1	0	1	1	1	1	1	1	1	1	1	1	1	1	1	1	1	1	1	1	1	1	1	1	1	1	1	1	yhcP	orf, hypothetical protein
b3250	1	1	1	1	1	1	1	1	1	1	1	1	1	1	1	1	1	1	1	1	1	1	1	1	1	0	1	1	1	1	1	1	1	1	1	1	1	1	1	1	1	1	1	mreC	rod shape-determining protein
b3251	1	1	1	1	1	1	1	1	1	1	1	1	1	1	1	1	1	1	1	1	1	1	1	1	1	1	0	1	1	1	1	1	1	1	1	1	1	1	1	1	1	1	1	mreB	regulator of ftsI, penicillin binding protein 3, septation function
b3263	0	1	1	1	1	1	1	1	1	1	1	1	1	1	1	1	1	1	1	1	1	1	1	1	1	1	1	1	1	1	1	1	0	1	1	1	1	1	1	1	1	1	1	yhdU	orf, hypothetical protein
b3264	0	1	1	1	1	1	1	1	1	1	1	1	1	1	1	1	0	1	1	1	1	1	1	1	1	1	1	1	1	1	1	1	0	0	0	0	0	0	0	0	0	0	0	envR	putative transcriptional regulator
b3265	1	1	1	1	1	1	1	1	1	1	1	1	1	1	1	1	1	1	1	1	1	1	1	1	1	1	1	1	1	1	1	1	0	0	0	0	0	0	0	0	0	1	1	acrE	transmembrane protein affects septum formation and cell membrane permeability
b3266	1	1	1	1	1	1	1	1	1	1	1	1	1	1	1	1	1	1	1	1	1	1	1	1	1	1	1	1	1	1	1	1	0	0	0	0	0	0	0	0	0	1	1	acrF	integral transmembrane protein; acridine resistance
b3267	0	1	0	1	1	1	1	1	1	1	1	1	1	1	1	1	1	1	1	1	1	1	1	1	1	1	1	1	1	1	1	1	0	0	0	0	0	0	0	0	0	1	1	yhdV	orf, hypothetical protein
b3268	1	0	1	1	1	1	1	1	1	1	1	1	1	1	1	1	1	1	1	1	1	1	1	1	1	1	1	1	1	1	1	1	1	1	1	1	0	1	1	0	1	1	1	yhdW	putative periplasmic binding transport protein
b3269	1	0	1	1	1	1	1	1	1	1	1	1	1	1	1	1	1	1	1	1	1	1	1	1	1	1	1	1	1	1	1	1	1	1	1	1	0	1	1	0	1	1	1	yhdX	putative transport system permease protein
b3270	1	0	1	1	1	1	1	1	1	1	1	1	1	1	1	1	1	1	1	1	1	1	1	1	1	1	1	1	1	1	1	1	1	1	1	1	1	1	1	1	1	1	1	yhdY	putative transport system permease protein
b3271	1	0	1	1	1	1	1	1	1	1	1	1	1	1	1	1	1	1	1	1	1	1	1	1	1	1	1	1	1	1	1	1	1	1	1	1	1	1	1	1	1	1	1	yhdZ	putative ATP-binding component of a transport system
b3291	1	1	1	1	1	1	0	1	0	1	1	1	0	1	1	1	1	1	1	1	1	1	1	1	0	0	1	1	1	1	1	1	1	0	0	1	0	0	1	0	1	1	1	mscL	mechanosensitive channel
b3291a	1	1	1	1	1	1	1	1	1	1	1	1	1	1	1	1	1	1	1	1	1	1	1	1	1	1	1	1	1	1	1	1	0	0	0	0	0	0	0	0	0	1	1	yhdL	Uncharacterized conserved protein
b3292	1	1	1	1	1	1	1	1	1	1	1	1	1	1	1	1	1	1	1	1	1	1	1	1	1	1	1	1	1	1	1	1	0	0	0	0	0	0	0	0	0	1	1	yhdM	putative transcriptional regulator
b3293	1	1	1	1	1	1	1	1	1	1	1	1	1	1	1	1	1	1	1	1	1	1	1	1	1	1	1	1	1	1	1	1	0	0	0	0	0	0	0	0	0	1	1	yhdN	orf, hypothetical protein
b3322	0	0	1	0	0	0	0	0	0	0	0	0	0	0	0	0	0	0	0	0	0	0	0	0	1	1	1	1	1	1	1	1	0	0	0	0	0	0	0	0	0	0	0	pinO	calcium-binding protein required for initiation of chromosome replication
b3323	0	0	1	0	0	0	0	0	0	0	0	0	0	0	0	0	0	0	0	0	0	0	0	0	1	1	1	1	1	1	1	1	0	0	0	0	0	0	0	0	0	0	0	yheD	putative export protein A for general secretion pathway (GSP)
b3324	0	0	0	0	0	0	0	0	0	0	0	0	0	0	0	0	0	0	0	0	0	0	0	0	1	1	1	1	1	1	1	1	0	0	0	0	0	0	0	0	0	0	0	yheE	putative general secretion pathway for protein export (GSP)
b3325	0	0	0	0	0	0	0	0	0	0	0	0	0	0	0	0	0	0	0	0	0	0	0	0	0	1	1	1	1	1	1	1	0	0	0	0	0	0	0	0	0	0	0	yheF	putative general protein secretion protein
b3326	0	0	0	0	0	0	0	0	0	0	0	0	0	0	0	0	0	0	0	0	0	0	0	0	0	1	1	1	1	0	1	0	0	0	0	0	0	0	0	0	0	0	0	yheG	putative general secretion pathway for protein export (GSP) (TYPE II TRAFFIC WARDEN ATPASE)
b3327	0	0	0	0	0	0	0	0	0	0	0	0	0	0	0	0	0	0	0	0	0	0	0	0	0	1	1	1	1	0	1	0	0	0	0	0	0	0	0	0	0	0	0	hofF	putative general protein secretion protein
b3328	0	0	0	0	0	0	0	0	0	0	0	0	0	1	0	0	0	0	0	0	0	0	0	0	0	1	1	1	1	0	1	0	0	0	0	0	0	0	0	0	0	0	0	hofG	putative general protein secretion protein

b3329	0	0	0	0	0	0	0	0	0	0	0	0	0	0	0	0	0	0	0	0	0	0	0	0	0	1	1	1	1	0	1	0	0	0	0	0	0	0	0	0	0	0	0	hofH	putative general protein secretion protein
b3330	0	0	0	0	0	0	0	0	0	0	0	0	0	0	0	0	0	0	0	0	0	0	0	0	0	1	1	1	1	0	1	0	0	0	0	0	0	0	0	0	0	0	0	yheH	putative export protein for general secretion pathway (GSP)
b3331	0	0	0	0	0	0	0	0	0	0	0	0	0	0	0	0	0	0	0	0	0	0	0	0	0	1	1	1	1	0	1	0	0	0	0	0	0	0	0	0	0	0	0	yheI	putative export protein I for general secretion pathway (GSP)
b3332	0	0	0	0	0	0	0	0	0	0	0	0	0	0	0	0	0	0	0	0	0	0	0	0	0	1	1	1	1	1	1	0	0	0	0	0	0	0	0	0	0	0	0	yheJ	putative export protein J for general secretion pathway (GSP)
b3333	0	0	0	0	0	0	0	0	0	0	0	0	0	0	0	0	0	0	0	0	0	0	0	0	0	1	1	1	1	1	1	1	0	0	0	0	0	0	0	0	0	0	0	yheK	putative general protein secretion protein
b3334	0	0	0	0	0	0	0	0	0	0	0	0	0	0	0	0	0	0	0	0	0	0	0	0	1	0	1	1	1	1	1	1	0	0	0	0	0	0	0	0	0	0	0	pshM	putative general secretion
b3335	0	0	0	0	0	0	0	0	0	0	0	0	0	0	0	0	0	0	0	0	0	0	0	0	1	0	1	1	1	1	1	1	0	0	1	0	0	0	0	0	0	0	0	hofD	leader peptidase, integral membrane protein
b3336	1	1	0	0	0	1	0	0	0	0	1	1	0	1	0	1	0	0	0	0	0	0	0	0	1	0	1	1	1	1	1	1	0	1	1	0	0	0	0	0	0	0	0	bfr	bacterioferrin, an iron storage homoprotein
b3337	0	0	0	0	0	0	0	0	0	0	0	0	0	0	0	0	0	0	0	0	0	0	0	0	1	0	1	1	1	1	1	1	0	0	0	0	0	0	0	0	0	0	0	yheA	orf, hypothetical protein
b3338	0	0	0	0	0	0	0	0	0	0	0	0	0	0	0	0	0	0	0	0	0	0	0	0	1	0	1	1	1	1	1	1	0	0	0	0	0	0	0	0	0	0	0	yheB	putative enzyme
b3345	1	1	1	1	1	1	1	1	1	1	1	1	1	1	1	1	1	1	1	1	1	1	1	1	1	1	0	1	1	1	1	1	1	1	1	1	1	1	1	1	1	1	1	yheN	orf, hypothetical protein
b3353	1	1	1	0	1	0	0	1	0	1	1	1	1	1	1	0	1	0	1	1	1	1	1	1	1	1	1	1	1	1	1	1	0	1	1	1	1	1	1	1	0	1	1	yheT	orf, hypothetical protein
b3358	1	1	1	0	1	0	1	1	0	1	1	1	1	1	1	1	1	0	1	1	1	1	1	1	0	1	1	1	1	1	1	0	1	1	1	1	1	1	1	1	1	1	1	yhfK	orf, hypothetical protein
b3362	1	1	1	1	1	1	1	0	0	1	1	1	1	1	1	1	1	1	1	1	1	1	1	1	1	1	1	1	1	1	1	1	1	1	1	1	1	1	1	1	1	1	1	yhfG	orf, hypothetical protein
b3366	1	1	1	1	1	1	1	1	1	1	1	1	1	1	1	1	1	1	1	1	1	1	1	1	1	1	1	1	1	1	1	1	1	1	1	0	1	1	1	1	1	1	1	nirD	nitrite reductase (NAD(P)H) subunit
b3369	0	1	1	1	1	1	1	1	1	1	1	1	1	1	1	1	1	1	1	1	1	1	1	1	1	1	1	1	1	1	1	1	1	1	1	1	1	1	1	1	1	1	1	yhfL	orf, hypothetical protein
b3370	0	0	1	0	1	1	1	1	1	1	1	1	1	1	1	1	1	1	1	1	1	1	1	1	0	0	0	0	0	0	0	0	1	1	1	1	1	1	1	1	1	1	1	yhfM	putative amino acid/amine transport protein
b3371	0	0	1	0	1	1	1	1	1	1	1	1	1	1	1	1	1	1	1	1	1	1	1	1	0	0	0	0	0	0	0	1	1	1	1	1	1	1	1	1	1	1	1	yhfN	putative transport protein
b3372	0	0	1	0	1	1	1	1	1	1	1	1	1	1	1	1	1	1	1	1	1	1	1	1	0	0	0	0	0	0	0	0	1	1	1	1	1	1	1	1	1	1	1	yhfO	orf, hypothetical protein
b3373	0	0	1	0	1	1	1	1	1	1	1	1	1	1	1	1	1	1	1	1	1	1	1	1	0	0	0	0	0	0	0	0	1	1	1	1	1	1	1	1	1	1	1	yhfP	orf, hypothetical protein
b3374	0	0	1	0	1	1	1	1	1	1	1	1	1	1	1	1	1	1	1	1	1	1	1	1	0	0	0	0	0	0	0	0	1	1	1	1	1	1	1	1	1	1	1	yhfQ	putative kinase
b3375	0	0	1	0	1	1	1	1	1	1	1	1	1	1	1	1	1	1	1	1	1	1	1	1	0	0	0	0	0	0	0	1	1	1	1	1	1	1	1	1	1	1	1	yhfR	putative transcriptional regulator
b3376	0	1	1	1	1	1	1	1	1	1	1	1	1	1	1	1	1	1	1	1	1	1	1	1	0	1	1	1	1	1	0	1	1	1	1	1	1	1	1	1	1	1	1	yhfS	orf, hypothetical protein
b3377	0	1	1	1	1	1	1	1	1	1	1	1	1	1	1	1	1	1	1	1	1	1	1	1	1	1	1	1	1	1	1	1	1	1	1	1	1	1	1	1	1	1	1	yhfT	putative transport system permease protein
b3380	1	1	1	1	1	1	1	1	1	1	1	1	1	1	1	1	1	1	1	1	1	1	1	1	1	0	0	0	1	0	0	1	1	1	1	1	1	1	1	1	1	1	1	yhfW	putative mutase
b3381	1	1	1	0	1	1	0	0	0	1	1	1	0	1	1	1	1	1	1	1	1	0	1	1	1	0	1	0	1	0	0	1	0	1	1	1	1	1	1	1	0	1	1	yhfX	orf, hypothetical protein
b3383	1	1	1	1	1	1	1	1	1	1	1	1	1	1	1	0	0	1	1	1	1	1	1	1	1	1	1	1	1	1	1	1	1	1	1	1	1	1	1	1	0	1	1	yhfZ	orf, hypothetical protein
b3385	1	1	1	1	1	1	1	1	1	1	1	1	1	1	1	1	1	1	1	1	1	1	1	1	1	1	0	1	1	1	1	1	1	1	1	1	1	1	1	1	1	1	1	gph	phosphoglycolate phosphatase
b3402	1	0	1	1	1	1	1	1	1	1	1	1	1	1	1	1	1	1	1	1	1	1	1	1	1	1	1	1	1	1	1	1	1	1	1	1	1	1	1	1	1	1	1	yhgE	putative transport
b3404	1	1	1	1	1	0	0	0	0	1	1	1	0	1	1	1	1	1	1	1	1	1	1	1	1	1	1	1	1	1	1	1	1	1	1	1	1	1	1	1	1	1	1	envZ	protein histidine kinase/phosphatase sensor for OmpR, modulates expression of ompF and ompC
b3409	1	1	1	1	1	1	0	1	0	1	1	1	0	1	1	1	1	1	1	1	1	0	1	1	1	1	1	1	1	1	1	1	0	1	1	1	1	1	1	1	1	1	1	feoB	ferrous iron transport protein B
b3410	1	1	1	1	1	1	1	1	1	1	1	1	1	1	1	1	1	1	1	1	1	1	1	1	1	0	0	1	1	1	1	1	0	1	1	1	1	1	1	1	1	1	1	yhgG	orf, hypothetical protein
b3411	1	0	1	1	1	1	1	1	1	1	1	1	1	1	1	1	1	1	1	1	1	1	1	1	1	1	1	1	1	1	1	1	1	1	1	1	1	1	1	1	1	1	1	yhgA	orf, hypothetical protein
b3418	1	1	1	0	1	1	1	1	1	1	1	1	1	1	1	1	1	1	1	1	1	1	1	1	1	1	1	1	1	0	0	0	1	1	1	1	1	1	1	1	1	1	1	malT	positive regulator of mal regulon
b3419m	1	1	1	0	1	1	1	1	1	1	1	1	1	1	1	1	1	0	1	1	1	1	1	1	1	1	1	1	1	0	0	0	0	1	1	1	1	1	1	1	0	1	1	yhgKJ	RNA phosphate cyclase
b3421	0	1	1	0	1	1	1	1	1	1	1	1	1	1	1	1	1	1	1	1	1	1	1	1	0	0	0	0	1	0	0	1	1	1	1	1	1	1	1	1	0	1	1	rtcB	orf, hypothetical protein
b3422	0	1	1	1	1	0	1	1	1	1	1	1	1	1	1	1	1	1	1	1	1	1	1	1	1	1	1	1	1	1	1	1	1	1	1	1	1	1	1	1	1	1	1	rtcR	putative 2-component regulator
b3423	1	1	1	1	1	1	0	0	0	1	1	1	0	1	1	1	1	0	1	1	1	1	1	1	0	0	0	1	0	1	1	0	1	1	1	1	1	1	1	1	1	1	1	glpR	repressor of the glp operon
b3424	1	1	1	1	1	0	1	1	1	1	1	1	1	1	1	1	1	1	1	1	1	1	1	1	1	1	1	1	1	1	1	1	1	1	1	1	1	1	1	1	1	1	1	glpG	protein of glp regulon
b3425	1	1	1	1	1	0	1	1	1	1	1	1	1	1	1	1	1	1	1	1	1	1	1	1	1	1	1	1	1	1	1	1	1	1	1	1	1	1	1	1	1	1	1	glpE	protein of glp regulon
b3426	1	1	1	1	1	1	0	0	0	1	1	1	0	1	1	1	1	0	1	1	1	1	1	1	0	1	0	0	0	0	0	1	0	1	1	1	1	1	1	1	1	1	1	glpD	sn-glycerol-3-phosphate dehydrogenase (aerobic)
b3427	1	1	1	1	1	1	1	1	1	0	0	1	0	1	1	1	1	1	1	1	1	1	1	1	1	1	1	1	1	1	1	1	1	1	1	1	1	1	1	1	1	1	1	yzgL	orf, hypothetical protein
b3434	1	1	1	1	1	1	1	1	1	1	1	1	1	1	1	1	1	1	1	1	1	1	1	1	1	1	0	1	1	0	1	1	1	1	1	1	1	1	1	1	1	1	1	yhgN	orf, hypothetical protein
b3438	1	1	1	1	1	1	1	1	1	1	1	1	0	1	1	1	1	1	1	1	1	1	1	1	1	1	1	1	1	1	1	1	1	1	1	1	1	1	1	1	1	1	1	gntR	regulator of gluconate (gnt) operon
b3441	1	1	1	1	1	1	1	1	1	1	1	1	1	1	1	1	1	1	1	1	1	1	1	1	1	0	0	1	1	1	0	1	1	1	1	1	1	1	1	1	1	1	1	yhhY	orf, hypothetical protein
b3442	0	1	0	1	1	1	1	1	1	1	1	1	1	1	1	1	1	1	1	1	1	1	1	1	1	1	1	1	0	0	1	1	1	0	0	1	1	1	1	1	1	1	1	yhhZ	orf, hypothetical protein
b3443	0	0	0	1	1	1	1	1	1	1	1	1	1	0	0	0	0	1	1	1	1	1	1	1	0	1	1	1	1	1	1	1	0	0	0	0	0	0	0	0	0	0	0	yrhA	orf, hypothetical protein
b3446	0	0	0	1	1	1	1	1	1	1	1	1	1	1	1	1	1	1	1	1	1	1	1	1	1	0	0	1	0	1	1	1	0	0	0	0	0	0	0	0	0	0	0	yrhB	orf, hypothetical protein
b3447	1	1	1	1	1	1	1	1	0	1	1	1	0	1	1	1	1	1	1	1	1	1	1	1	1	0	0	1	1	1	1	1	1	1	1	1	1	1	1	1	1	1	1	ggt	gamma-glutamyltranspeptidase
b3448	1	1	1	1	1	1	1	1	1	1	1	1	1	1	1	1	1	1	1	1	1	1	1	1	1	1	1	1	1	1	1	1	1	1	1	1	1	1	1	1	0	1	1	yhhA	orf, hypothetical protein
b3449	1	1	1	1	1	1	1	1	1	1	1	1	1	1	1	1	1	1	1	1	1	1	1	1	1	1	0	1	1	0	1	1	1	1	1	1	1	1	1	1	1	1	1	ugpQ	glycerophosphodiester phosphodiesterase, cytosolic
b3450	1	1	1	1	1	1	1	1	1	1	1	1	1	1	1	1	1	1	1	1	1	1	1	1	1	0	1	1	0	1	1	1	1	1	1	1	1	1	1	1	1	1	1	ugpC	ATP-binding component of sn-glycerol 3-phosphate transport system
b3451	1	1	1	1	1	1	1	1	1	1	1	1	1	1	1	1	1	1	1	1	1	1	1	1	1	1	0	1	1	0	1	1	1	1	1	1	1	1	1	1	1	1	1	ugpE	sn-glycerol 3-phosphate transport system, integral membrane protein
b3452	1	1	1	1	1	1	1	1	1	1	1	1	1	1	1	1	1	1	1	1	1	1	1	1	1	1	0	1	1	0	1	1	1	1	1	1	1	1	1	1	1	1	1	ugpA	sn-glycerol 3-phosphate transport system, integral membrane protein
b3453	1	1	1	1	1	1	1	1	1	1	1	1	0	0	0	0	1	1	1	1	1	1	1	1	1	1	1	1	1	0	1	1	1	1	1	1	1	1	1	1	1	1	1	ugpB	sn-glycerol 3-phosphate transport system; periplasmic binding protein
b3454	1	1	1	1	1	1	0	0	1	1	1	1	1	1	1	1	1	1	1	1	1	1	1	1	1	1	1	1	1	0	1	1	0	1	1	1	1	1	1	1	1	1	1	livF	ATP-binding component of leucine transport
b3455	1	1	1	1	1	1	1	1	1	1	1	1	1	1	1	1	1	1	1	1	1	1	1	1	1	1	1	1	1	0	1	1	0	1	1	1	1	1	1	1	1	1	1	livG	ATP-binding component of high-affinity branched-chain amino acid transport system
b3457	1	1	1	1	1	1	0	0	1	1	1	1	0	1	1	1	1	1	1	1	1	0	1	1	1	1	0	1	1	1	1	1	1	1	1	1	1	1	1	1	1	1	1	livH	high-affinity branched-chain amino acid transport system; membrane component
b3473	1	1	1	0	1	1	0	0	1	0	0	1	0	1	0	0	0	0	0	0	0	0	0	0	0	1	0	0	0	0	0	0	0	1	1	0	0	0	0	0	0	0	0	yhhS	putative transport
b3481	1	1	1	1	1	1	1	0	0	1	1	1	0	1	1	1	1	0	1	1	1	1	1	1	0	0	0	0	0	0	0	1	1	1	1	1	1	1	1	1	1	1	1	yhhG	orf, hypothetical protein
b3482	1	1	1	0	1	1	1	1	0	1	1	1	1	1	1	1	1	0	1	1	1	1	1	1	1	1	0	1	1	1	1	1	0	0	0	0	0	0	0	0	0	0	0	rhsB	rhsB protein in rhs element
b3483	0	0	0	0	0	0	0	0	0	0	0	0	0	0	0	0	0	0	0	0	0	0	0	0	0	0	0	0	0	0	0	0	0	0	0	0	0	0	0	0	0	0	0	yhhH	orf, hypothetical protein
b3484	1	1	1	1	1	0	1	0	0	0	0	0	0	0	0	0	0	0	0	0	0	1	0	0	1	1	1	1	1	1	1	1	0	0	0	0	0	0	0	0	0	0	0	yhhI	putative receptor
b3485	0	0	1	1	1	1	1	1	1	1	1	1	1	1	1	1	1	1	1	1	1	1	1	1	1	1	1	1	1	0	1	1	1	1	1	1	1	1	1	1	1	1	1	yhhJ	putative transporter
b3486	0	0	1	1	1	1	1	1	1	1	1	1	1	1	1	1	1	1	1	1	1	1	1	1	1	1	1	1	1	0	1	0	1	1	1	1	1	1	1	1	1	1	1	yhiH	putative ATP-binding component of a transport system, fragment 1
b3487	0	0	1	1	1	1	1	1	1	1	1	1	1	1	1	1	1	1	1	1	1	1	1	1	0	0	0	0	0	0	1	1	0	1	1	1	1	1	1	1	0	1	1	yhiI	putative membrane protein
b3488	0	0	1	1	0	0	0	0	0	0	0	0	1	1	1	1	1	0	0	0	0	0	0	0	1	1	1	1	1	0	1	0	0	1	1	1	1	1	1	1	1	1	1	yhiJ	orf, hypothetical protein
b3489	0	0	1	1	0	0	0	0	0	0	0	0	1	1	1	1	1	0	0	0	0	0	0	0	1	1	1	1	1	0	1	0	1	1	1	1	1	1	1	1	1	1	1	yhiK	orf, hypothetical protein
b3490	0	0	1	1	0	0	0	0	0	0	0	0	1	1	1	1	1	0	0	0	0	0	0	0	0	1	1	1	0	0	0	0	0	1	1	1	1	1	1	1	0	1	1	yhiL	orf, hypothetical protein
b3491	0	1	1	1	1	0	0	0	0	0	0	0	1	1	1	1	1	0	0	0	0	0	0	0	1	1	1	1	1	0	1	0	1	1	1	1	1	1	1	1	1	1	0	yhiM	orf, hypothetical protein
b3492	1	1	1	1	1	1	1	1	1	1	1	1	1	1	1	1	1	1	1	1	1	1	1	1	1	1	1	1	1	0	1	1	1	1	1	1	1	1	1	1	1	1	1	yhiN	orf, hypothetical protein
b3497	1	1	1	1	1	1	1	1	1	1	1	1	1	1	1	1	1	1	1	1	1	1	1	1	1	1	0	0	0	0	1	1	1	1	1	1	1	1	1	1	1	1	1	yhiQ	orf, hypothetical protein
b3499	1	1	1	1	1	0	1	0	1	1	1	1	1	1	1	1	1	1	1	1	1	1	1	1	1	1	1	1	1	1	1	1	0	1	1	1	1	1	1	1	1	1	1	yhiR	orf, hypothetical protein
b3501	1	0	1	1	1	1	1	1	1	1	1	1	1	1	1	1	1	1	1	1	1	1	1	1	1	0	0	1	0	1	1	1	1	1	1	1	1	1	1	1	1	1	1	arsR	transcriptional repressor of chromosomal ars operon
b3502	1	1	1	1	1	1	1	0	0	1	1	1	1	1	1	0	1	1	1	1	1	1	1	1	1	1	1	1	1	1	1	1	1	1	1	1	1	1	1	1	0	1	1	arsB	arsenical pump membrane protein
b3503	1	0	1	1	1	1	1	1	1	1	1	1	1	1	1	1	1	1	1	1	1	1	1	1	1	1	1	1	0	1	1	1	1	1	1	1	1	1	1	1	1	1	1	arsC	arsenate reductase
b3504	0	0	1	1	1	1	1	1	1	1	1	1	1	1	1	1	0	1	1	1	1	1	1	1	1	1	1	1	1	1	1	1	1	1	1	1	1	1	1	1	1	1	1	yhiS	orf, hypothetical protein
b3508	1	0	1	1	1	1	1	1	1	1	1	1	1	1	1	1	1	1	1	1	1	1	1	1	1	1	1	1	1	1	1	1	1	1	1	1	1	1	1	1	1	1	1	yhiD	putative transport ATPase
b3515	1	0	1	1	1	1	1	1	1	1	1	1	1	1	1	1	1	1	1	1	1	1	1	1	1	1	1	1	1	1	1	1	1	1	1	1	1	1	1	1	1	1	1	yhiW	putative ARAC-type regulatory protein
b3516	1	0	1	1	1	1	1	1	1	1	1	1	1	1	1	1	1	1	1	1	1	1	1	1	1	1	1	1	1	1	1	1	1	1	1	0	1	1	1	1	1	1	1	yhiX	putative ARAC-type regulatory protein
b3517	1	1	1	1	1	0	1	1	1	1	1	1	1	1	1	1	1	1	1	1	1	1	1	1	1	1	1	1	1	1	1	1	1	1	1	1	1	1	1	1	1	1	1	gadA	glutamate decarboxylase isozyme
b3518	0	1	1	1	1	1	1	1	1	1	1	1	1	1	1	1	1	1	1	1	1	1	1	1	1	1	1	1	1	1	1	1	1	1	1	1	1	1	1	1	1	1	1	yhjA	putative cytochrome C peroxidase (EC 1.11.1)
b3519	0	1	1	1	1	1	1	1	1	1	0	1	1	1	1	1	1	1	1	1	1	1	1	1	1	1	1	1	1	1	1	1	0	1	1	1	1	1	1	1	1	1	1	treF	cytoplasmic trehalase
b3520	0	1	1	1	1	1	1	1	1	1	0	1	1	1	1	1	1	1	1	1	1	1	1	1	1	1	1	1	1	1	1	1	0	1	1	1	1	1	1	1	1	1	1	yhjB	putative regulator
b3521	1	1	1	1	1	1	1	1	1	1	1	1	1	1	1	1	1	1	1	1	1	1	1	1	1	0	0	0	0	1	0	1	0	1	1	1	1	1	1	1	1	1	1	yhjC	putative transcriptional regulator LYSR-type
b3523	1	1	1	1	1	1	1	1	1	1	1	1	1	1	1	1	1	1	1	1	1	1	1	1	0	1	1	1	1	1	1	1	1	1	1	1	1	1	1	1	1	1	1	yhjE	putative transport protein
b3524	1	0	1	1	1	1	1	1	1	1	1	1	1	1	1	1	1	1	1	1	1	1	1	1	1	1	1	1	1	1	1	1	1	1	1	1	1	1	1	1	1	1	1	yhjG	orf, hypothetical protein
b3526	1	1	1	1	1	1	1	1	1	1	1	1	1	1	1	1	1	1	1	1	1	1	1	1	1	1	1	1	1	1	1	1	0	1	1	1	1	1	1	1	0	1	1	kdgK	ketodeoxygluconokinase
b3529	1	1	1	1	1	1	1	1	1	1	1	1	1	1	1	1	1	1	1	1	1	1	1	1	1	1	1	1	1	1	1	1	1	1	1	1	0	1	1	1	1	1	1	yhjK	orf, hypothetical protein
b3530	1	0	1	1	1	1	0	0	1	1	1	1	1	1	1	1	1	1	1	1	1	1	1	1	1	1	1	1	1	1	1	1	1	1	1	1	1	1	1	1	1	1	1	yhjL	putative oxidoreductase subunit
b3531	1	1	1	1	1	1	1	0	1	1	1	1	0	1	1	1	1	1	1	1	1	1	1	1	1	1	1	1	1	1	1	1	1	1	1	1	1	1	1	1	1	1	1	yhjM	putative endoglucanase
b3532	1	0	1	1	1	1	1	1	1	1	1	1	1	1	1	1	1	1	1	1	1	1	1	1	1	1	1	1	1	0	1	1	1	1	1	1	1	1	1	1	1	1	1	yhjN	orf, hypothetical protein
b3534	1	0	1	1	1	1	1	1	1	1	1	1	1	1	1	1	1	1	1	1	0	1	1	1	1	1	1	1	1	0	1	1	1	1	1	1	1	1	1	1	1	1	1	yhjQ	orf, hypothetical protein
b3536	1	0	1	1	1	1	1	1	1	1	1	1	1	1	1	1	1	1	1	1	0	1	1	1	0	1	1	1	1	1	1	0	1	1	1	1	1	1	1	1	1	1	1	yhjS	putative protease
b3537	1	0	1	1	1	1	1	1	1	1	1	1	1	1	1	1	1	1	1	1	0	1	1	1	1	1	1	1	1	1	1	0	1	1	1	0	1	1	1	1	1	1	1	yhjT	orf, hypothetical protein
b3538	1	0	1	1	1	1	1	1	1	1	1	1	1	1	1	1	1	1	1	1	0	1	1	1	1	1	1	1	1	1	1	1	1	1	1	1	1	1	1	1	1	1	1	yhjU	orf, hypothetical protein
b3539	1	0	1	1	1	1	1	1	1	1	1	1	1	1	1	1	1	1	1	1	1	1	1	1	1	1	1	1	1	1	1	1	1	1	1	0	1	1	1	1	1	1	1	yhjV	putative transporter protein
b3540	1	1	1	1	1	1	1	1	1	1	1	1	1	1	1	1	1	1	1	1	1	1	1	1	0	0	1	1	0	0	0	1	0	1	1	1	1	1	1	1	1	1	1	dppF	putative ATP-binding component of dipeptide transport system
b3541	1	1	1	1	1	1	1	1	1	1	1	1	1	1	1	1	1	1	1	1	1	1	1	1	1	1	1	1	1	1	1	1	1	1	1	0	1	1	1	1	1	1	1	dppD	putative ATP-binding component of dipeptide transport system
b3542	1	1	1	1	1	1	1	1	1	1	1	1	1	1	1	1	1	1	1	1	1	1	1	1	1	1	1	1	1	1	1	1	0	1	1	1	1	1	1	1	1	1	1	dppC	dipeptide transport system permease protein 2
b3546	1	1	1	1	1	1	1	1	1	1	1	1	1	1	1	1	1	1	1	1	1	1	1	1	1	1	1	1	1	1	1	1	1	1	1	0	1	1	1	1	1	1	1	yhjW	orf, hypothetical protein
b3547	1	1	1	1	1	1	1	1	1	1	1	1	1	1	1	1	1	1	1	1	1	1	1	1	1	1	1	1	1	1	1	1	1	1	1	0	1	1	1	1	1	1	1	yhjX	putative resistance protein
b3550	1	1	1	1	1	1	1	1	1	1	1	1	1	1	1	1	1	1	1	1	1	1	1	1	1	1	1	1	1	1	1	1	1	1	1	1	1	1	1	0	1	1	1	yiaC	orf, hypothetical protein
b3551	1	1	1	1	1	1	1	1	1	1	1	1	1	1	1	1	1	1	1	1	1	1	1	1	1	1	1	1	1	1	1	1	1	1	1	0	1	1	1	1	1	1	1	bisC	biotin sulfoxide reductase
b3554	1	1	1	1	1	1	0	0	0	1	1	1	1	1	1	0	1	1	1	1	1	1	1	1	1	1	1	1	1	1	0	1	1	1	1	1	1	1	1	1	1	1	1	yiaF	orf, hypothetical protein
b3557	0	1	0	1	1	1	1	1	1	1	1	1	1	0	1	1	1	0	0	1	1	1	1	1	1	1	1	1	1	1	1	1	1	1	1	1	1	1	1	1	1	1	1	yi5A	IS150 hypothetical protein

b3558	0	1	0	1	0	0	0	0	0	0	0	0	0	0	0	0	0	0	0	0	0	0	0	0	1	1	1	1	1	1	1	1	1	1	1	1	1	1	1	1	1	1	1	t150	IS150 putative transposase
b3561	1	1	1	0	1	0	0	0	0	1	0	0	1	1	1	1	1	1	1	1	1	1	1	1	1	1	1	1	1	1	1	1	1	1	1	0	1	1	1	1	1	1	1	yiaH	orf, hypothetical protein
b3562	0	0	1	0	1	0	0	0	0	0	0	0	1	1	1	1	0	1	1	1	1	1	1	1	1	1	1	1	1	1	1	1	1	1	1	1	1	1	1	1	1	1	1	yiaA	orf, hypothetical protein
b3563	0	0	1	1	1	0	1	0	0	0	0	0	1	1	1	1	0	1	1	1	1	1	1	1	1	1	1	1	1	1	1	1	1	1	1	1	1	1	1	1	1	1	1	yiaB	orf, hypothetical protein
b3564	0	0	1	1	1	1	1	1	1	1	1	1	1	1	1	1	1	1	1	1	1	0	0	1	1	1	1	1	1	1	1	1	1	1	1	1	1	1	1	1	1	1	1	xylB	xylulokinase
b3565	0	0	1	1	1	1	1	1	1	1	1	1	1	1	1	1	1	1	1	1	1	0	0	0	1	1	1	1	1	1	1	1	1	1	1	1	1	1	1	1	1	1	1	xylA	D-xylose isomerase
b3566	1	0	0	1	1	1	1	1	1	1	1	1	1	1	1	1	1	1	1	1	1	0	0	0	1	1	1	1	1	1	1	1	1	1	1	1	1	1	1	1	1	1	1	xylF	xylose binding protein transport system
b3567	0	0	0	1	1	1	1	1	1	1	1	1	1	1	1	1	1	1	1	1	1	1	0	0	1	1	1	1	1	0	1	1	1	1	1	1	1	1	1	1	1	1	1	xylG	putative ATP-binding protein of xylose transport system
b3568	0	0	0	1	1	1	1	1	1	1	1	1	1	1	1	1	1	1	1	1	1	1	0	0	1	1	1	1	1	0	1	1	1	1	1	1	1	1	1	1	1	1	1	xylH	putative xylose transport, membrane component
b3569	1	0	0	1	1	1	1	1	1	1	1	1	1	1	1	1	1	1	1	1	1	1	1	1	1	1	1	1	1	1	1	1	1	1	1	1	1	1	1	1	1	1	1	xylR	putative regulator of xyl operon
b3570	1	1	0	1	1	1	1	1	1	1	1	1	1	1	1	1	1	1	1	1	1	1	1	1	1	1	1	1	1	1	1	1	1	1	1	1	1	1	1	1	1	1	1	bax	putative ATP-binding protein
b3571	1	0	0	1	1	1	1	1	1	1	1	1	1	1	1	1	1	1	1	1	1	1	1	1	1	1	1	1	1	1	1	1	1	1	1	1	1	1	1	1	1	1	1	malS	alpha-amylase
b3572	1	1	0	1	1	1	1	1	1	1	1	1	1	1	1	1	1	1	1	1	1	1	1	1	1	1	1	1	1	1	1	1	1	1	1	1	1	1	1	1	1	1	1	avtA	alanine-alpha-ketoisovalerate (or valine-pyruvate) transaminase, transaminase C
b3573	1	1	0	1	1	1	1	1	1	1	1	1	1	1	1	1	1	1	1	1	1	1	1	1	1	1	1	1	1	1	1	1	1	1	1	0	1	1	1	1	1	1	1	yiaI	orf, hypothetical protein
b3574	0	1	0	1	1	1	1	1	1	1	1	1	1	1	1	1	1	1	1	1	1	1	1	1	1	1	1	1	1	1	1	1	1	1	1	1	1	1	1	1	1	1	1	yiaJ	putative regulator
b3575	0	1	0	1	1	1	1	1	1	1	1	1	1	1	1	1	1	1	1	1	1	1	1	1	1	1	1	1	1	1	1	1	1	1	1	1	0	1	1	1	1	1	1	yiaK	putative dehydrogenase
b3576	0	0	0	1	0	1	1	1	1	1	1	1	1	1	1	1	1	1	1	1	1	1	1	0	1	1	1	1	1	1	1	1	0	0	0	0	0	0	0	0	0	1	1	yiaL	putative lipase
b3577	0	0	0	1	0	1	1	1	1	1	1	1	0	1	1	1	1	1	1	1	1	1	1	0	1	1	1	1	1	1	1	1	1	1	1	0	1	1	1	1	0	0	0	yiaM	orf, hypothetical protein
b3578	0	0	0	1	0	1	1	1	1	1	1	1	0	1	1	1	1	1	1	1	1	1	1	0	1	1	1	1	1	1	1	1	1	1	1	1	1	1	0	1	0	0	0	yiaN	putative membrane protein
b3579	0	0	0	1	0	0	0	0	0	0	0	0	0	0	0	0	0	0	0	0	1	0	0	0	1	1	1	1	1	1	1	0	0	1	0	0	1	1	0	1	0	0	0	yiaO	putative solute-binding transport protein
b3580	0	0	0	1	0	0	0	0	0	0	0	0	0	0	0	0	0	0	0	0	0	0	0	0	1	1	1	1	1	1	1	1	0	1	0	0	1	1	0	1	0	0	0	lyxK	L-xylulose kinase, cryptic
b3581	0	0	0	1	0	0	0	0	0	0	0	0	0	0	0	0	0	0	0	0	0	0	0	0	1	1	1	1	1	1	1	1	0	1	0	0	1	1	0	1	0	0	0	sgbH	probable 3-hexulose 6-phosphate synthase
b3582	0	0	0	1	0	0	0	0	0	0	0	0	0	0	0	0	0	0	0	0	0	0	0	0	1	1	1	1	1	1	1	1	0	1	0	0	1	1	0	1	0	0	0	sgbU	probable 3-hexulose-6-phosphate isomerase
b3583	0	0	0	0	0	0	0	0	0	0	0	0	0	0	0	0	0	0	0	0	0	0	0	0	1	1	1	1	1	1	1	0	0	1	0	0	1	1	0	1	0	0	0	sgbE	putative epimerase/aldolase
b3584	0	0	0	0	0	0	0	0	0	0	0	0	0	0	0	0	0	0	0	0	0	0	0	0	1	1	1	1	1	1	1	1	0	1	0	0	1	1	0	1	0	0	0	yiaT	putative outer membrane protein
b3585	1	0	0	0	1	0	0	0	0	0	0	0	0	0	0	0	0	0	0	0	0	0	0	0	1	1	1	1	1	1	1	1	1	1	0	0	1	1	1	1	1	1	1	yiaU	putative transcriptional regulator LYSR-type
b3586	1	0	1	0	1	0	0	0	0	0	0	0	0	1	0	0	0	0	0	0	0	0	0	0	1	1	1	1	1	1	1	1	1	1	0	0	1	1	1	1	1	1	1	yiaV	putative membrane protein
b3587	0	1	0	0	1	0	0	0	0	0	0	0	0	0	0	0	0	0	0	0	0	0	0	0	1	1	1	1	1	1	1	1	1	1	0	0	1	1	1	1	1	1	1	yiaW	orf, hypothetical protein
b3588	0	0	0	1	1	1	1	1	1	1	1	1	1	1	1	1	1	1	1	1	1	1	1	1	1	1	1	1	1	1	1	1	1	1	1	1	1	1	1	1	1	1	1	aldB	aldehyde dehydrogenase B (lactaldehyde dehydrogenase)
b3589	0	1	0	1	1	1	1	1	1	1	1	1	1	1	1	1	1	1	1	1	1	1	1	1	1	1	1	1	1	1	1	1	1	1	1	1	1	1	1	1	1	1	1	yiaY	putative oxidoreductase
b3591	1	1	1	1	1	1	1	1	1	1	1	1	1	1	1	1	1	1	1	1	1	1	1	1	1	1	1	1	1	1	1	1	1	1	1	1	0	1	1	1	1	1	1	selA	selenocysteine synthase: L-seryl-tRNA (Ser) selenium transferase
b3593	1	1	1	0	1	1	0	1	0	1	1	1	1	1	1	1	1	0	1	1	1	1	1	1	1	1	0	1	1	1	1	1	0	0	0	0	0	0	0	0	0	0	0	rhsA	rhsA protein in rhs element
b3594	1	0	0	0	1	0	0	1	0	1	0	0	0	0	0	0	0	0	0	1	1	1	1	1	0	0	0	0	1	0	0	1	0	0	0	0	0	0	0	0	0	0	0	yibA	orf, hypothetical protein
b3595	1	1	1	0	1	1	1	1	1	1	1	1	1	1	1	0	1	1	1	1	1	1	1	1	1	1	1	1	1	1	1	1	0	0	0	0	0	0	0	0	0	0	0	yibJ	orf, hypothetical protein
b3596	0	0	0	0	0	1	1	1	1	1	1	1	0	1	1	1	1	0	0	0	0	0	0	0	1	1	1	1	1	0	1	0	0	0	0	0	0	0	0	0	0	0	0	yibG	orf, hypothetical protein
b3597	0	1	1	0	1	1	1	1	1	0	1	1	0	1	0	1	1	0	0	0	1	0	1	0	1	1	1	1	1	1	1	1	1	1	1	1	1	1	1	1	1	1	1	yibH	putative membrane protein
b3598	0	1	1	0	0	1	1	1	1	1	1	1	0	1	1	1	1	0	0	0	0	0	0	0	1	1	1	1	1	0	1	0	1	1	1	0	1	1	1	1	1	1	1	yibI	orf, hypothetical protein
b3599	0	1	1	1	1	1	1	1	1	1	1	1	0	1	0	1	1	0	1	1	1	1	1	1	1	1	1	1	1	1	1	1	1	1	1	1	0	1	1	1	1	1	0	mtlA	PTS system, mannitol-specific enzyme IIABC components
b3600	0	1	1	0	0	1	1	1	1	0	1	1	0	1	0	1	0	0	0	1	1	1	1	0	1	1	0	1	0	1	1	1	0	1	1	1	1	1	1	1	1	1	1	mtlD	mannitol-1-phosphate dehydrogenase
b3601	0	1	1	1	1	1	1	1	1	1	1	1	0	1	0	1	1	0	1	1	1	1	1	1	1	1	1	1	1	1	1	1	1	1	1	0	1	1	1	1	1	1	1	mtlR	repressor for mtl
b3602	0	1	1	0	0	1	1	1	1	1	1	1	0	1	0	1	0	0	0	1	1	1	1	0	1	1	1	1	1	1	1	0	1	1	1	1	1	1	1	1	1	1	1	yibL	orf, hypothetical protein
b3603	1	1	1	1	1	1	0	1	1	1	1	1	0	1	1	1	1	1	1	1	1	1	1	1	1	1	0	1	1	0	1	1	1	1	1	1	1	1	1	1	1	1	1	lldP	L-lactate permease
b3604	1	1	1	1	1	1	1	1	1	1	1	1	1	1	1	1	1	1	1	1	1	1	1	1	1	1	1	1	1	1	1	1	1	1	1	0	1	1	1	1	1	1	1	lldR	transcriptional regulator
b3608	1	1	1	1	1	1	1	1	1	1	1	1	1	1	1	1	1	1	1	1	1	1	1	1	1	1	1	1	1	1	1	1	1	1	1	0	1	1	1	1	1	1	1	gpsA	glycerol-3-phosphate dehydrogenase (NAD+)
b3612	1	1	1	1	1	1	1	1	1	1	1	1	1	1	1	1	1	1	1	1	1	1	1	1	1	1	1	1	1	1	1	1	1	1	1	0	1	1	1	1	1	1	1	yibO	putative 2,3-bisphosphoglycerate-independent phosphoglycerate mutase
b3615	1	1	1	1	1	1	1	1	1	1	1	1	1	1	1	1	1	1	1	1	1	1	1	1	1	1	1	1	1	1	1	1	1	1	0	0	0	0	0	0	1	1	1	yibD	putative regulator
b3616	1	1	1	1	1	1	0	0	1	1	1	1	0	1	1	0	1	0	1	1	1	1	1	1	1	0	0	0	0	0	1	1	0	1	1	1	1	1	1	1	1	1	1	tdh	threonine dehydrogenase
b3618	0	0	0	0	0	0	0	0	0	0	0	0	0	0	0	0	0	0	0	0	0	0	0	0	1	1	1	1	1	1	1	1	1	1	1	1	1	1	1	1	1	1	1	htrL	involved in lipopolysaccharide biosynthesis
b3621	1	1	1	1	1	1	1	1	1	1	1	1	1	1	1	1	1	1	1	1	1	1	1	1	1	1	1	1	1	1	1	1	1	1	1	0	0	1	1	1	1	1	1	rfaC	heptosyl transferase I; lipopolysaccharide core biosynthesis
b3622	0	0	0	0	0	0	0	0	0	0	0	0	0	0	0	0	0	0	0	0	0	0	0	0	0	0	0	0	0	0	0	0	0	0	0	0	0	0	0	0	0	0	0	rfaL	O-antigen ligase; lipopolysaccharide core biosynthesis
b3623	0	0	0	0	0	0	0	0	0	0	0	0	0	0	0	0	0	0	0	0	0	0	0	0	0	0	0	0	0	0	0	0	0	0	0	0	0	0	0	0	0	0	0	rfaK	probably hexose transferase; lipopolysaccharide core biosynthesis
b3624	0	1	0	0	0	0	0	0	0	0	0	0	0	0	0	0	0	0	0	0	0	0	0	0	0	0	0	0	0	0	0	0	0	0	0	0	0	0	0	0	0	0	0	rfaZ	lipopolysaccharide core biosynthesis
b3625	0	1	0	0	0	0	0	0	0	0	0	0	0	0	0	0	0	0	0	0	0	0	0	0	0	0	0	0	0	0	0	0	0	0	0	0	0	0	0	0	0	0	0	rfaY	lipopolysaccharide core biosynthesis
b3626	0	1	0	0	0	0	0	0	0	0	0	0	0	0	0	0	0	0	0	0	0	0	0	0	0	0	0	0	0	0	0	0	0	0	0	0	0	0	0	0	0	0	0	rfaJ	UDP-D-glucose:(galactosyl)lipopolysaccharide glucosyltransferase
b3627	0	1	0	0	0	0	0	0	0	0	0	0	0	0	0	0	0	0	0	0	0	0	0	0	0	0	0	0	0	0	0	0	0	0	0	0	0	0	0	0	0	0	0	rfaI	UDP-D-galactose:(glucosyl)lipopolysaccharide- alpha-1,3-D-galactosyltransferase
b3628	0	1	0	0	0	0	0	0	0	0	0	0	0	0	0	0	0	0	0	0	0	0	0	0	0	0	0	0	0	0	0	0	0	0	0	0	0	0	0	0	0	0	0	rfaB	UDP-D-galactose:(glucosyl)lipopolysaccharide-1,6- D-galactosyltransferase
b3629	0	0	0	0	0	0	0	0	0	0	0	0	0	0	0	0	0	0	0	0	0	0	0	0	0	0	0	0	0	0	0	0	0	0	0	0	0	0	0	0	0	0	0	rfaS	lipopolysaccharide core biosynthesis
b3630	1	1	1	0	0	0	0	0	0	0	0	0	0	0	0	0	0	0	0	0	0	0	0	0	0	0	0	0	0	0	0	0	0	1	1	0	0	0	0	0	0	0	0	rfaP	lipopolysaccharide core biosynthesis; phosphorylation of core heptose
b3631	1	1	1	0	0	0	0	0	0	0	0	0	0	1	0	0	0	0	0	0	1	0	0	0	0	0	0	0	0	0	0	1	0	1	1	0	0	0	0	0	0	0	0	rfaG	glucosyltransferase I; lipopolysaccharide core biosynthesis
b3632	1	1	1	0	0	0	0	0	0	0	0	0	0	1	0	0	0	0	0	0	1	0	0	0	0	0	0	0	0	0	0	0	0	1	1	0	0	0	0	0	0	0	0	rfaQ	lipopolysaccharide core biosynthesis
b3635	1	1	1	1	1	0	1	1	0	1	1	1	1	1	1	1	1	0	1	1	1	0	1	1	1	1	1	1	1	1	1	1	0	1	1	1	1	1	1	1	1	1	1	mutM	formamidopyrimidine DNA glycosylase
b3638	1	1	1	1	1	1	1	1	1	1	1	1	1	1	1	1	1	1	1	1	1	1	1	1	1	1	1	1	1	1	1	1	1	1	1	0	0	1	1	1	1	1	1	radC	DNA repair protein
b3641	1	1	1	1	1	1	1	1	1	1	1	1	1	1	1	1	1	1	1	1	1	1	1	1	1	1	1	1	1	1	1	1	1	1	1	0	1	1	1	1	1	1	1	ttk	putative transcriptional regulator
b3644	1	1	1	1	1	1	1	1	1	1	1	1	1	1	1	1	1	1	1	1	1	1	1	1	1	1	1	1	1	1	1	1	1	1	1	0	1	1	1	1	1	1	1	yicC	putative alpha helix protein
b3645	1	1	1	0	0	0	0	0	0	0	0	0	0	1	0	0	0	0	0	0	0	0	0	0	0	0	0	0	0	0	0	0	0	1	1	0	0	0	0	0	0	0	0	dinD	DNA-damage-inducible protein
b3651	1	1	1	1	1	1	1	1	1	1	1	1	1	1	1	1	1	1	1	1	1	1	1	1	1	1	1	1	1	1	1	1	1	1	1	1	0	1	1	1	1	1	1	spoU	putative RNA methylase
b3653	1	1	1	1	1	1	1	1	1	1	1	1	1	1	1	1	1	1	1	1	1	1	1	1	1	1	1	1	1	1	1	1	1	1	1	0	1	1	1	1	1	1	1	gltS	glutamate transport
b3655	1	1	1	1	1	1	1	1	1	1	1	1	1	1	1	1	1	1	1	1	1	1	1	1	1	1	1	1	1	1	1	1	1	1	1	0	0	1	1	1	1	1	1	yicH	orf, hypothetical protein
b3656	1	0	1	1	1	1	1	1	1	1	1	1	1	1	1	1	1	1	1	1	1	1	1	1	1	1	1	1	1	1	1	1	1	1	1	1	1	1	1	1	1	1	1	yicI	orf, hypothetical protein
b3657	1	0	1	1	1	1	1	1	1	1	1	1	1	1	1	1	1	1	1	1	1	1	1	1	1	1	1	1	1	1	1	1	1	1	1	1	1	1	1	1	1	1	1	yicJ	putative permease
b3659	1	1	0	0	1	1	1	1	1	1	1	1	1	1	1	1	0	1	1	1	1	1	1	1	1	1	1	1	1	1	1	1	0	0	0	0	0	0	0	0	1	0	0	yicK	two-module transport protein
b3660	1	1	0	1	1	1	1	1	1	1	1	1	1	1	1	1	1	1	1	1	1	1	1	1	1	1	1	1	1	1	1	1	1	0	0	1	1	1	1	1	1	1	1	yicL	putative permease transporter
b3661	1	0	1	0	1	1	1	1	1	1	1	1	1	1	1	1	0	1	1	1	0	0	1	0	1	1	1	1	1	1	1	1	0	0	0	0	0	0	0	0	1	0	0	nlpA	lipoprotein-28
b3662	1	0	1	1	1	1	1	1	1	0	1	1	1	1	1	1	1	1	1	1	0	0	1	0	1	1	1	1	1	1	1	1	1	1	1	0	1	1	1	1	1	1	1	yicM	putative transport protein
b3663	1	1	1	1	1	1	1	1	1	1	1	1	1	1	1	1	1	1	1	1	0	0	1	0	1	1	1	1	1	1	1	1	1	1	1	1	1	1	1	1	1	1	1	yicN	orf, hypothetical protein
b3664	1	0	1	1	1	1	1	1	1	1	1	1	1	1	1	1	1	1	1	1	1	0	1	0	1	1	1	1	1	1	1	1	1	1	1	1	0	1	1	1	1	1	1	yicO	orf, hypothetical protein
b3665	1	1	1	1	1	1	1	1	1	1	1	1	1	1	1	1	1	1	1	1	1	1	1	1	1	1	1	1	1	1	1	1	1	1	1	0	1	1	1	1	1	1	1	yicP	probable adenine deaminase (synthesis xanthine)
b3667	1	1	1	1	1	1	1	1	1	1	1	1	1	1	1	1	1	1	1	1	1	1	1	1	1	1	1	1	1	1	1	1	1	1	1	0	1	1	1	1	1	1	1	uhpC	regulator of uhpT
b3671	1	1	1	1	1	1	1	1	1	1	1	1	1	1	1	1	1	1	1	1	1	1	1	1	1	1	1	1	1	1	1	1	1	1	1	0	1	1	1	1	1	1	1	ilvB	acetolactate synthase I,valine-sensitive, large subunit
b3674	1	1	1	1	1	1	1	1	1	1	1	1	1	1	1	1	1	1	1	1	1	1	1	1	1	1	1	1	1	1	1	1	1	1	1	0	1	1	1	1	1	1	1	yidF	putative transcriptional regulator
b3675	1	1	1	1	1	1	1	1	1	1	1	1	1	1	1	1	1	1	1	1	1	1	1	1	1	1	1	1	1	1	1	1	1	1	1	0	1	1	1	1	1	1	1	yidG	orf, hypothetical protein
b3677	1	1	1	0	0	1	0	0	0	0	0	0	0	0	0	0	0	0	0	0	0	0	0	0	1	1	1	1	1	1	1	1	1	0	0	1	1	1	1	1	1	1	1	yidI	orf, hypothetical protein
b3679	1	0	0	0	0	1	0	0	0	0	0	0	0	0	0	0	0	0	0	0	0	0	0	0	0	1	0	0	1	1	1	1	1	1	1	0	1	1	1	1	1	1	1	yidK	putative cotransporter
b3680	1	0	0	0	0	0	0	0	0	0	0	0	0	0	0	0	0	0	0	0	0	0	0	0	0	1	0	0	1	1	1	1	1	1	1	1	1	1	1	1	1	1	1	yidL	putative ARAC-type regulatory protein
b3681	1	1	1	1	1	1	1	1	1	1	1	1	1	1	1	1	1	1	1	1	1	1	1	1	1	1	1	1	1	1	1	1	1	1	1	0	1	1	1	1	1	1	1	glvG	probable 6-phospho-beta-glucosidase
b3684	1	1	1	1	1	1	0	1	1	1	1	0	1	1	0	0	1	1	1	1	1	1	1	1	1	1	0	1	1	1	1	1	1	1	1	1	1	1	1	1	1	1	1	yidP	putative transcriptional regulator
b3685	1	1	1	1	1	1	1	1	1	1	1	1	1	1	1	1	1	1	1	1	1	1	1	1	1	1	1	1	1	1	1	1	1	1	1	0	1	1	1	1	1	1	1	yidE	putative transport protein
b3688	1	1	1	1	1	1	1	1	1	1	1	1	1	1	1	1	1	1	1	1	1	1	1	1	1	1	1	1	1	1	1	1	1	1	1	0	0	1	1	1	1	1	1	yidQ	orf, hypothetical protein
b3689	1	1	1	1	1	1	1	1	0	1	1	1	1	1	1	1	1	1	1	1	1	1	1	1	1	1	1	1	1	1	1	1	1	1	1	1	1	1	1	1	1	1	1	yidR	orf, hypothetical protein
b3690	1	1	1	1	1	0	0	0	1	0	0	0	0	0	0	0	0	0	0	0	0	1	1	0	0	0	0	0	1	1	1	1	1	1	1	1	1	1	1	1	0	1	1	yidS	orf, hypothetical protein
b3691	0	1	1	1	1	0	0	0	0	0	0	1	0	0	0	0	1	0	0	0	1	0	0	0	0	0	0	0	1	1	1	1	1	1	1	1	1	1	1	1	1	1	1	dgoT	D-galactonate transport
b3692	0	1	1	1	1	0	0	0	0	0	0	0	0	0	0	0	0	0	0	0	0	0	0	0	1	1	0	0	1	1	1	1	1	1	1	1	1	1	1	1	1	1	1	dgoA	2-oxo-3-deoxygalactonate 6-phosphate aldolase and galactonate dehydratase
b3693	0	1	1	1	1	0	1	1	1	1	1	1	1	0	1	1	1	1	1	1	1	1	1	1	1	1	1	1	1	1	1	1	1	1	1	1	1	1	1	1	1	1	1	dgoK	2-oxo-3-deoxygalactonate kinase
b3694	0	1	1	1	1	0	0	0	0	0	0	0	0	0	0	0	0	0	0	0	0	0	0	0	1	1	1	1	1	1	1	1	1	1	1	0	0	1	1	1	1	1	1	b3694	putative FADA-type transcriptional regulator
b3695	0	1	1	1	1	1	0	1	1	1	1	1	0	0	1	1	1	1	1	1	1	1	1	1	1	1	1	1	1	1	1	1	1	1	1	0	1	1	1	1	1	1	1	yidW	regulator protein for dgo operon
b3696	1	1	1	0	1	0	0	0	0	0	0	0	0	0	0	0	0	0	0	0	0	0	0	0	1	1	1	1	1	1	1	1	1	1	1	1	1	1	1	1	1	1	1	yidX	putative replicase EC 2.7.-
b3697	1	1	1	1	1	0	0	0	0	0	0	0	0	0	0	0	0	0	0	0	0	0	0	0	1	1	1	1	1	1	1	1	1	1	1	0	1	1	1	1	1	1	1	yidA	orf, hypothetical protein
b3708	0	1	0	1	1	0	1	1	1	1	1	0	1	0	0	1	1	1	1	1	1	1	1	1	1	1	1	1	1	1	1	1	1	1	1	0	1	1	1	1	1	1	1	tnaA	tryptophanase
b3709	1	1	0	1	1	0	1	1	1	1	1	1	1	1	1	1	1	1	1	1	1	1	1	1	1	1	1	1	1	1	1	1	1	1	1	0	1	1	1	1	1	1	1	tnaB	low affinity tryptophan permease
b3710	1	1	1	1	1	1	1	0	0	1	1	1	0	1	1	1	1	1	1	1	1	1	1	1	1	1	1	1	1	1	1	1	1	1	1	1	0	1	1	1	1	1	1	yidY	putative transport protein
b3712	1	1	1	1	1	1	1	1	1	1	1	1	1	1	1	1	1	0	0	1	1	1	1	1	1	1	1	1	1	1	1	1	1	1	1	1	1	1	1	1	1	1	1	yieE	orf, hypothetical protein
b3713	1	1	1	1	1	1	1	1	1	1	1	1	1	1	1	1	1	0	0	1	1	1	1	1	1	1	1	1	1	1	1	1	1	1	1	1	1	1	1	1	1	1	1	yieF	orf, hypothetical protein
b3714	1	1	1	1	1	1	1	1	1	1	1	1	1	1	1	1	1	0	0	1	1	1	1	1	1	1	1	1	1	1	1	1	1	1	1	1	1	1	1	1	1	1	1	yieG	putative membrane / transport protein
b3715	1	1	1	1	0	0	0	0	0	0	0	0	0	0	0	0	0	0	0	0	0	0	0	0	1	1	1	1	0	1	1	0	1	1	1	0	1	1	1	1	1	1	1	yieH	putative phosphatase
b3716	0	0	1	0	1	0	0	0	0	0	0	0	0	0	0	0	0	0	0	0	0	0	0	0	0	0	0	0	0	0	0	0	1	1	1	0	1	1	1	1	1	1	1	yieI	orf, hypothetical protein
b3717	0	1	1	0	1	0	0	0	0	0	0	0	0	0	0	0	0	0	0	0	1	0	0	0	0	0	0	0	0	0	0	0	1	1	1	0	1	1	1	1	1	1	1	yieJ	orf, hypothetical protein

b3718	0	1	1	0	1	0	0	0	0	0	0	0	0	0	0	0	1	0	0	0	1	0	0	0	0	0	0	0	0	0	0	1	1	1	1	0	1	1	1	1	1	1	1	yieK	putative isomerase
b3719	0	0	1	0	0	0	0	0	0	0	0	0	0	0	0	0	0	0	0	0	0	0	0	0	0	0	0	0	0	0	0	0	1	1	1	1	1	1	1	1	1	0	0	yieL	putative xylanase
b3720	0	0	1	0	0	0	0	0	0	0	0	0	0	0	0	0	0	0	0	0	0	0	0	0	0	0	0	0	0	0	0	0	1	1	1	0	1	1	1	1	1	0	0	yieC	putative receptor protein
b3721	0	0	1	0	0	0	0	0	0	0	0	0	0	0	0	0	0	0	0	0	0	0	0	0	0	0	0	0	0	0	0	0	1	1	1	1	1	1	1	1	1	1	1	bglB	phospho-beta-glucosidase B; cryptic
b3722	0	0	1	0	0	0	0	0	0	0	0	0	0	0	0	0	0	0	0	0	0	0	0	0	0	0	0	0	0	0	0	0	1	1	1	1	1	1	1	1	1	1	1	bglF	PTS system beta-glucosides, enzyme II, cryptic
b3723	0	0	1	1	1	1	0	1	1	1	1	1	0	0	0	0	1	0	1	1	1	1	1	1	0	0	0	0	0	0	0	0	1	1	1	0	1	1	1	1	1	1	1	bglG	positive regulation of bgl operon
b3724	1	1	1	1	1	0	1	1	1	1	1	1	1	1	1	1	0	1	1	1	1	1	1	1	1	1	1	1	1	1	1	0	1	1	1	1	1	1	1	1	1	1	1	phoU	negative regulator for pho regulon and putative enzyme in phosphate metabolism
b3731	1	1	1	1	1	1	0	0	0	1	1	1	0	1	0	1	1	1	1	0	1	1	0	1	0	0	0	0	1	1	1	1	1	1	1	0	1	1	1	1	1	1	1	atpC	membrane-bound ATP synthase, F1 sector, epsilon-subunit
b3738	1	1	1	1	1	1	1	1	1	1	1	1	1	1	1	1	1	1	1	1	1	1	1	1	1	1	1	1	1	1	1	1	1	1	1	0	1	1	1	1	1	1	1	atpB	membrane-bound ATP synthase, F0 sector, subunit a
b3742	1	1	1	1	1	1	1	1	1	1	1	1	1	1	1	1	1	1	1	1	1	1	1	1	1	1	1	1	1	1	1	1	1	1	1	0	1	1	1	1	1	1	1	mioC	initiation of chromosome replication
b3745	1	1	1	1	1	1	1	1	1	1	1	1	1	1	1	1	1	1	1	1	1	1	1	1	1	1	1	1	1	0	1	0	1	1	1	0	1	1	1	1	1	1	1	yieM	orf, hypothetical protein
b3746	1	1	1	1	1	1	1	1	1	1	1	1	1	1	1	1	1	1	1	1	1	1	1	1	1	1	1	1	0	0	0	0	1	1	1	1	1	1	1	1	1	1	1	yieN	putative 2-component regulator
b3747	1	1	1	1	1	1	1	1	1	1	1	1	1	1	1	1	1	1	1	1	1	1	1	1	1	1	1	1	1	1	0	1	1	1	1	1	1	1	1	1	1	1	1	kup	low affinity potassium transport system
b3748	1	1	1	1	1	1	1	1	1	1	1	1	1	1	1	1	1	1	1	1	1	1	1	1	1	1	1	1	1	0	0	0	1	1	1	1	1	1	1	1	1	1	1	rbsD	D-ribose high-affinity transport system; membrane-associated protein
b3749	1	1	1	1	1	1	1	1	1	1	1	1	1	1	1	1	1	0	0	0	1	0	1	0	1	1	1	1	1	0	0	0	1	1	1	1	1	1	1	1	1	1	1	rbsA	ATP-binding component of D-ribose high-affinity transport system
b3750	1	1	1	1	1	1	1	1	1	1	1	1	1	1	1	1	1	1	1	1	1	1	1	1	1	1	1	1	1	0	0	0	1	1	1	1	1	1	1	1	1	1	1	rbsC	D-ribose high-affinity transport system
b3751	1	1	1	1	1	1	1	1	1	1	1	1	1	1	1	1	1	1	1	1	1	1	1	1	1	1	1	1	1	1	0	1	1	1	1	1	1	1	1	1	1	1	1	rbsB	D-ribose periplasmic binding protein
b3753	1	1	1	1	1	1	1	1	1	1	1	1	1	1	1	1	1	1	1	1	1	1	1	1	1	1	1	1	1	1	1	1	1	1	1	0	1	1	1	1	1	1	1	rbsR	regulator for rbs operon
b3754	1	1	1	1	1	1	1	1	1	1	1	1	1	1	1	1	1	1	1	1	1	1	1	1	1	1	1	1	1	1	1	1	1	1	1	0	1	1	1	1	1	1	1	yieO	putative transport protein
b3755	1	1	1	1	1	1	1	1	1	1	1	1	1	1	1	1	1	1	1	1	1	1	1	1	1	1	0	1	1	1	1	1	1	1	1	1	1	1	1	1	1	1	1	yieP	orf, hypothetical protein
b3764	1	1	1	1	1	1	0	0	1	1	1	1	0	1	1	1	1	1	1	1	1	1	1	1	0	0	0	0	0	0	0	1	1	1	1	0	1	1	1	1	1	1	1	yifE	orf, hypothetical protein
b3765	1	1	1	1	1	1	1	1	1	1	1	1	1	1	1	1	1	1	1	1	1	1	1	1	1	1	1	1	0	1	1	1	1	1	1	0	1	1	1	1	1	1	1	yifB	putative 2-component regulator
b3769	1	1	1	1	1	1	1	1	1	1	1	1	1	1	1	1	1	1	1	1	1	1	1	1	1	1	1	1	1	1	1	1	1	1	1	0	1	1	1	1	1	1	1	ilvM	acetolactate synthase II, valine insensitive, small subunit
b3770	1	1	1	1	1	1	1	1	1	1	1	1	1	1	1	1	1	1	1	1	1	1	1	1	1	1	1	1	1	1	1	1	1	1	1	0	1	1	1	1	1	1	1	ilvE	branched-chain amino-acid aminotransferase
b3771	1	1	1	1	1	1	0	0	0	1	1	1	0	1	1	1	1	1	1	1	1	1	1	1	1	0	0	0	0	0	1	1	0	1	1	1	1	1	1	1	0	1	1	ilvD	dihydroxyacid dehydratase
b3773	1	1	1	1	1	1	0	0	0	1	1	1	0	1	1	1	1	0	1	1	1	0	1	1	0	1	1	1	0	0	0	1	1	1	1	1	1	1	1	1	1	1	1	ilvY	positive regulator for ilvC
b3774	1	1	1	1	1	1	1	1	1	1	1	1	1	1	1	1	1	1	1	1	1	1	1	1	1	1	1	1	1	1	1	1	0	1	1	1	1	1	1	1	1	1	1	ilvC	ketol-acid reductoisomerase
b3777m	1	1	1	0	1	1	1	1	1	1	1	1	1	1	1	1	1	1	1	1	1	1	1	1	1	1	1	1	1	1	1	1	0	0	0	0	0	0	0	0	0	0	0	yifN	Uncharacterized conserved protein
b3780	1	1	1	1	1	1	1	1	1	1	1	1	1	1	1	1	1	1	1	1	1	1	1	1	1	1	1	1	1	1	1	1	1	1	1	0	1	1	1	1	1	1	1	rhlB	putative ATP-dependent RNA helicase
b3786	1	1	1	1	1	1	1	1	1	1	1	1	1	1	1	1	1	1	1	1	1	1	1	1	1	1	1	1	1	1	1	1	1	1	1	0	1	1	1	1	1	1	1	wecB	UDP-N-acetyl glucosamine -2-epimerase; synthesis of enterobacterial common antigen (ECA)
b3787	1	1	1	1	1	1	1	1	1	1	1	1	1	1	1	1	1	1	1	1	1	1	1	1	1	1	1	1	1	1	1	1	1	1	1	0	1	1	1	1	1	1	1	wecC	UDP-N-acetyl-D-mannosaminuronic acid dehydrogenase; synthesis of enterobacterial common antigen (ECA)
b3788	1	1	1	1	1	1	1	1	1	1	1	1	1	1	1	1	1	1	1	1	1	1	1	1	1	1	1	1	1	1	1	1	1	1	1	1	0	1	1	1	1	1	1	rffG	dTDP-glucose 4,6-dehydratase
b3790	1	1	1	1	1	1	1	1	1	1	1	1	1	1	1	1	1	1	1	1	1	1	1	1	1	1	1	1	1	1	1	1	1	1	1	0	1	1	1	1	1	1	1	wecD	orf, hypothetical protein
b3793	1	1	1	1	1	1	1	1	1	1	1	1	1	1	1	1	1	1	1	1	1	1	1	1	1	1	1	1	1	1	1	1	1	1	1	0	1	1	1	1	1	1	1	wecF	TDP-Fuc4NAc:lipidII transferase; synthesis of enterobacterial common antigen (ECA)
b3800	1	1	1	1	1	1	1	1	1	1	1	1	1	1	1	0	0	1	1	1	1	1	1	1	1	0	1	0	0	1	0	0	0	1	1	1	1	1	1	1	1	1	1	aslB	putative arylsulfatase regulator
b3801	1	1	1	1	1	0	1	0	1	1	1	1	0	1	1	1	1	1	1	1	1	1	1	1	1	1	1	1	1	1	0	1	0	0	0	1	1	1	1	1	1	1	1	aslA	arylsulfatase
b3803	1	1	1	1	1	1	1	1	1	1	1	1	1	1	1	1	1	1	1	1	1	1	1	1	1	1	1	1	1	1	1	1	1	1	1	0	1	1	1	1	1	1	1	hemX	uroporphyrinogen III methylase
b3805	1	1	1	1	1	1	1	1	1	1	1	1	1	1	1	1	1	1	1	1	1	1	1	1	1	1	1	1	1	1	1	1	1	1	1	0	1	1	1	1	1	1	1	hemC	porphobilinogen deaminase = hydroxymethylbilane synthase
b3807	1	1	1	1	1	1	1	1	1	1	1	1	1	1	1	1	1	1	1	1	1	1	1	1	1	1	1	1	1	1	1	1	1	1	1	0	1	1	1	1	1	1	1	cyaY	orf, hypothetical protein
b3808	1	1	1	1	1	1	1	1	1	1	1	1	1	1	1	1	1	1	1	1	1	1	1	1	1	1	0	1	1	1	1	1	0	1	1	1	1	1	1	1	1	1	1	b3808	orf, hypothetical protein
b3810	1	1	1	1	1	1	1	1	1	1	1	1	1	1	1	1	1	1	1	1	1	1	1	1	1	1	1	1	1	1	1	1	0	1	1	1	1	1	1	1	1	1	1	yigA	orf, hypothetical protein
b3811	1	1	1	1	1	1	1	1	1	1	1	1	1	1	1	1	1	1	1	1	1	1	1	1	1	1	1	1	1	1	1	1	0	1	1	1	1	1	1	1	1	1	1	xerC	site-specific recombinase, acts on cer sequence of ColE1, effects chromosome segregation at cell division
b3815m	1	1	1	1	1	1	1	1	1	1	1	1	1	1	1	1	1	1	1	1	1	1	1	1	1	1	1	1	1	1	1	1	1	1	1	0	1	1	1	1	1	1	1	yigE	Predicted periplasmic protein
b3817	1	0	0	1	0	0	0	0	0	1	0	0	0	0	0	0	1	0	0	0	1	1	1	1	1	1	1	1	1	1	1	1	1	1	1	1	1	1	1	1	1	1	1	yigF	orf, hypothetical protein
b3818	1	1	0	1	0	0	0	0	0	0	0	0	0	0	0	0	0	0	0	0	1	0	0	0	1	1	1	1	1	1	1	1	1	1	1	1	1	1	1	1	1	1	1	yigG	orf, hypothetical protein
b3820	1	1	1	1	1	1	1	1	1	1	1	1	1	1	1	1	1	1	1	1	1	1	1	1	1	1	1	1	1	1	1	1	1	1	1	0	1	1	1	1	1	1	1	yigI	orf, hypothetical protein
b3822	1	1	1	1	1	1	1	1	1	1	1	1	1	1	1	1	1	1	1	1	1	1	1	1	1	1	1	1	1	1	1	1	1	1	1	0	1	1	1	1	1	1	1	recQ	ATP-dependent DNA helicase
b3826m	1	1	1	1	1	1	1	1	1	1	1	1	1	1	1	1	1	1	1	1	1	1	1	1	1	1	1	1	1	1	1	1	1	1	1	0	1	1	1	1	1	1	1	yigL	Predicted hydrolase (HAD superfamily)
b3827	1	1	1	1	1	1	1	1	1	1	1	1	1	1	1	1	1	1	1	1	1	1	1	1	1	1	1	1	1	1	1	1	1	1	1	0	1	1	1	1	1	1	1	yigM	orf, hypothetical protein
b3828	1	1	1	1	1	1	1	1	1	1	1	1	1	1	1	1	1	1	1	1	1	1	1	1	1	1	0	0	0	1	1	1	0	1	1	1	1	1	1	1	1	1	1	metR	regulator for metE and metH
b3830	1	1	1	1	1	1	1	1	1	1	1	1	1	1	1	1	1	1	1	1	1	1	1	1	1	1	1	1	1	1	1	1	1	1	1	0	1	1	1	1	1	1	1	ysgA	putative enzyme
b3831	1	1	1	1	1	1	1	1	1	1	1	1	1	1	1	1	1	1	1	1	1	1	1	1	1	1	1	1	1	1	1	1	1	1	1	0	1	1	1	1	1	1	1	udp	uridine phosphorylase
b3832	1	1	1	1	1	1	1	1	1	1	1	1	1	1	1	1	1	1	1	1	1	1	1	1	1	1	1	1	1	1	1	1	1	1	1	0	1	1	1	1	1	1	1	yigN	putative alpha helix chain
b3834	1	1	1	1	1	1	1	1	1	1	1	1	1	1	1	1	1	1	1	1	1	1	1	1	1	1	1	1	1	1	1	1	1	1	1	0	1	1	1	1	1	1	1	yigP	orf, hypothetical protein
b3839	1	1	1	1	1	1	1	1	1	1	1	1	1	1	1	1	1	1	1	1	1	1	1	1	1	1	1	1	1	1	1	1	1	1	1	0	1	1	1	1	1	1	1	yigU	orf, hypothetical protein
b3842	1	1	1	1	1	1	1	1	1	1	1	1	1	1	1	1	1	1	1	1	1	1	1	1	1	1	0	1	1	1	1	1	1	1	1	1	1	1	1	1	1	1	1	rfaH	transcriptional activator affecting biosynthesis of lipopolysaccharide core, F pilin, and haemolysin
b3846	1	1	1	1	1	1	1	1	1	1	1	1	1	1	1	1	1	1	1	1	1	1	1	1	1	1	1	1	1	1	0	1	1	1	1	1	1	1	1	1	1	1	1	fadB	4-enzyme protein
b3848	1	1	1	1	1	1	1	1	1	1	1	1	1	1	1	1	1	1	1	1	1	1	1	1	1	1	1	1	1	1	1	1	1	1	1	1	1	1	1	1	1	0	1	yigZ	orf, hypothetical protein
b3856	1	1	1	1	1	1	1	1	1	1	1	1	1	1	1	1	1	1	1	1	1	1	1	1	1	1	1	1	1	0	0	1	1	1	1	1	1	1	1	1	1	1	1	mobB	molybdopterin-guanine dinucleotide biosynthesis protein B
b3861	1	0	1	1	1	1	1	1	1	1	1	1	1	1	1	1	1	1	1	1	1	1	1	1	1	1	1	1	1	1	1	1	1	1	1	1	1	1	1	1	1	1	1	yihF	putative GTP-binding protein
b3862	1	0	1	1	1	1	1	1	1	1	1	1	1	1	1	1	1	1	1	1	1	1	1	1	1	1	1	1	1	1	1	1	1	1	1	1	1	1	1	1	1	1	1	yihG	putative endonuclease
b3872	1	0	0	1	1	0	0	0	0	0	0	0	0	0	0	0	1	0	0	0	0	1	1	0	1	0	1	1	1	1	1	1	1	1	1	1	1	1	1	1	1	1	1	yihL	putative transcriptional regulator
b3873	0	0	0	1	0	0	0	0	0	0	0	0	0	0	0	0	0	0	0	0	0	0	0	0	1	0	1	1	1	1	1	0	1	1	1	1	1	1	1	1	1	0	0	yihM	orf, hypothetical protein
b3874	0	0	0	1	1	0	0	0	0	0	0	0	0	0	0	0	1	0	0	0	1	0	0	0	1	1	1	1	0	1	1	1	1	1	1	1	1	1	1	1	0	0	0	yihN	putative resistance protein (transport)
b3875	0	1	0	1	0	0	0	0	0	0	0	0	0	0	0	0	0	0	0	0	0	0	0	0	0	0	0	0	0	0	0	0	1	1	1	1	1	1	1	1	1	0	0	yshA	orf, hypothetical protein
b3876	0	1	0	1	0	0	0	0	0	0	0	0	0	0	0	0	0	0	0	0	0	0	0	0	0	0	0	0	0	0	0	0	0	1	1	1	1	1	1	1	1	0	0	yihO	putative permease
b3877	0	1	0	1	0	0	0	0	0	0	0	0	0	0	0	0	0	0	0	0	0	0	0	0	0	0	0	0	0	0	0	0	0	1	1	1	1	1	1	1	1	0	0	yihP	putative permease
b3878	0	1	0	1	0	0	0	0	0	0	0	0	0	0	0	0	0	0	0	0	0	0	0	0	0	0	0	0	0	0	0	0	0	1	1	1	1	1	1	1	1	0	0	yihQ	putative glycosidase
b3879	0	1	0	1	1	0	0	0	0	0	0	0	0	0	0	0	0	0	0	0	0	0	0	0	0	0	0	0	0	0	0	0	0	1	1	1	1	1	1	1	1	0	0	yihR	putative aldose-1-epimerase (EC 5.1.3.3)
b3880	0	1	0	1	1	0	0	0	0	0	0	0	0	0	0	0	0	0	0	0	0	0	0	0	0	0	0	0	0	0	0	0	0	1	1	1	1	1	1	1	1	1	1	yihS	orf, hypothetical protein
b3881	0	1	0	1	1	0	0	0	0	0	0	0	0	0	0	0	0	0	0	0	0	0	0	0	0	0	0	0	0	0	0	0	0	1	1	1	1	1	1	1	1	1	1	yihT	putative aldolase
b3882	0	1	0	1	1	1	0	0	0	1	0	1	0	1	0	0	0	0	0	0	1	1	0	1	0	0	0	1	0	0	0	0	0	1	1	1	1	1	1	1	1	1	1	yihU	putative dehydrogenase
b3883	0	1	0	1	1	0	0	0	0	0	0	0	0	0	0	0	0	0	0	0	0	0	0	0	0	0	0	0	0	0	0	0	1	1	1	1	1	1	1	1	1	1	1	yihV	putative kinase
b3884	0	1	0	1	1	0	0	0	0	0	0	0	0	0	0	0	0	0	0	0	0	0	0	0	1	0	1	1	1	1	1	0	1	1	1	1	1	1	1	1	1	1	1	yihW	putative DEOR-type transcriptional regulator
b3889	0	0	0	1	1	1	1	1	1	1	1	1	1	1	1	1	0	1	1	1	1	1	1	1	1	1	1	1	1	1	1	1	0	1	1	1	1	1	1	1	1	1	1	yiiE	orf, hypothetical protein
b3890	0	1	1	1	1	1	1	1	1	1	1	1	1	1	1	1	1	1	1	1	1	1	1	1	1	1	1	1	1	1	1	1	0	1	1	1	1	1	1	1	1	1	1	yiiF	orf, hypothetical protein
b3891	1	1	1	1	1	1	1	1	1	1	1	1	1	1	1	1	1	1	1	1	1	1	1	1	1	1	1	1	1	1	1	1	1	1	1	1	1	1	1	0	1	1	1	fdhE	affects formate dehydrogenase-N
b3896	1	0	1	1	1	1	1	1	1	1	1	1	1	1	1	1	1	1	1	1	1	1	1	1	1	1	1	1	1	1	1	1	0	0	0	0	0	0	0	0	0	0	0	yiiG	orf, hypothetical protein
b3897	0	0	1	0	0	0	0	0	0	0	0	0	0	1	1	1	0	1	1	1	0	1	1	1	1	1	1	1	1	1	1	1	1	1	1	1	1	1	1	1	0	0	0	frvR	putative frv operon regulatory protein
b3898	0	0	1	0	0	0	0	0	0	0	0	0	0	1	1	1	0	1	1	1	0	1	1	1	1	1	1	1	1	1	1	1	1	1	1	1	1	1	1	1	0	0	0	frvX	frv operon protein
b3899	0	0	1	0	0	0	0	0	0	0	0	0	0	1	1	1	0	1	1	1	0	1	1	1	1	1	1	1	1	1	1	1	1	1	1	1	1	1	1	1	0	0	0	frvB	PTS system, fructose-like enzyme IIBC component
b3900	0	0	1	1	0	0	0	0	0	0	0	0	0	1	1	1	1	1	1	1	1	1	1	1	1	1	1	1	1	1	1	1	1	1	1	1	1	1	1	1	0	0	0	frvA	PTS system, fructose-specific IIA component
b3902	1	0	1	1	1	1	1	1	1	1	1	1	0	1	1	1	1	1	1	1	1	1	1	1	1	1	1	1	1	1	1	1	1	1	1	1	1	1	1	1	0	1	1	rhaD	rhamnulose-phosphate aldolase
b3903	1	0	1	1	1	0	0	0	0	0	0	0	1	1	1	1	1	1	1	1	1	1	1	1	1	1	1	1	1	1	1	1	1	1	1	1	1	1	1	1	1	1	1	rhaA	L-rhamnose isomerase
b3904	1	1	1	1	1	1	1	1	1	1	1	1	1	1	1	0	1	1	1	1	1	1	1	1	1	1	1	1	1	1	1	1	1	1	1	1	1	1	1	1	1	1	1	rhaB	rhamnulokinase
b3905	1	0	1	1	1	1	1	1	1	1	1	1	1	1	1	1	1	1	1	1	1	1	1	1	1	1	1	1	1	1	1	1	1	1	1	1	1	1	1	1	1	1	1	rhaS	positive regulator for rhaBAD operon
b3906	1	0	1	1	1	1	1	1	1	1	1	1	1	1	1	1	1	1	1	1	1	1	1	1	1	1	1	1	1	1	1	1	1	1	1	1	1	1	1	1	1	1	1	rhaR	positive regulator for rhaRS operon
b3907	1	0	1	1	1	1	1	1	1	1	1	1	1	1	1	1	1	1	1	1	1	1	1	1	1	1	1	1	1	1	1	1	1	1	1	1	1	1	1	1	1	1	1	rhaT	rhamnose transport
b3909	1	0	1	1	1	1	1	1	1	1	1	1	1	1	1	1	1	1	1	1	1	1	1	1	1	1	1	1	1	1	1	1	1	1	1	1	1	1	1	1	1	1	1	kdgT	2-keto-3-deoxy-D-gluconate transport system
b3913m	1	1	1	1	1	1	1	1	1	1	1	1	1	1	1	1	1	1	1	1	1	1	1	1	1	1	1	1	1	1	1	1	0	1	1	1	1	1	1	1	1	1	1	yiiO	Uncharacterized periplasmic protein
b3915	1	1	1	1	1	1	1	1	1	1	1	1	0	1	1	1	1	1	1	1	1	1	1	1	1	1	1	1	1	1	0	1	1	1	1	1	1	1	1	1	1	1	1	yiiP	putative transport system permease protein
b3916	1	1	1	1	1	1	1	0	1	1	1	1	1	1	1	1	1	1	1	1	1	1	1	1	1	1	1	1	1	1	1	1	0	1	1	1	1	1	1	1	1	1	1	pfkA	6-phosphofructokinase I
b3922	1	1	1	1	1	1	1	1	1	1	1	1	1	1	1	1	1	0	0	0	1	1	1	1	1	1	1	1	1	1	1	1	1	1	1	1	1	1	1	1	1	1	1	yiiS	orf, hypothetical protein
b3926	1	1	1	1	1	1	1	1	1	1	1	1	1	1	1	1	1	1	1	1	1	1	1	1	1	0	0	1	0	1	1	1	1	1	1	1	1	1	1	1	1	1	1	glpK	glycerol kinase
b3940	1	0	1	0	0	0	0	0	0	0	0	0	0	1	0	0	0	0	0	0	0	0	0	0	0	0	0	0	0	0	0	0	0	1	1	0	0	0	0	0	0	0	0	metL	aspartokinase II and homoserine dehydrogenase II
b3944	1	1	1	1	1	1	1	1	1	1	1	1	1	1	1	1	1	1	1	1	1	1	1	1	1	1	1	1	1	1	1	1	1	1	1	0	1	1	1	1	1	1	1	yijF	orf, hypothetical protein
b3945	1	1	1	1	1	1	1	1	1	1	1	1	1	1	1	1	1	1	1	1	1	1	1	1	1	1	1	1	1	1	1	1	1	1	1	0	1	1	1	1	1	1	1	gldA	glycerol dehydrogenase, (NAD)
b3946	1	1	1	1	1	1	1	1	1	1	1	1	1	1	1	1	1	1	1	1	1	1	1	1	1	1	1	1	1	1	1	1	1	1	1	0	1	1	1	1	1	1	1	talC	putative transaldolase
b3964	1	1	1	1	1	1	1	1	1	1	1	1	1	1	1	1	1	1	1	1	1	1	1	1	1	0	0	1	1	0	1	1	1	1	1	1	1	1	1	1	1	1	1	yijD	orf, hypothetical protein
b3973	1	1	1	0	0	0	0	0	0	0	0	0	0	1	0	0	0	0	0	0	0	0	0	0	0	0	1	1	1	0	0	0	0	1	1	0	0	0	0	0	0	0	0	birA	biotin-[acetylCoA carboxylase] holoenzyme synthetase and biotin operon repressor
b3980	1	1	1	1	1	1	1	1	1	1	1	1	1	1	1	1	1	1	1	1	1	1	1	1	1	1	1	1	1	1	0	1	1	1	1	1	1	1	1	1	1	1	1	tufB	protein chain elongation factor EF-Tu (duplicate of tufA)
b3989	1	0	0	1	1	1	1	1	1	1	1	1	1	1	1	1	0	1	1	1	1	1	1	1	1	1	1	1	1	1	1	1	0	0	0	0	0	0	0	0	0	0	0	htrC	heat shock protein htrC
b4003	1	1	1	0	1	1	0	0	0	0	1	1	0	1	0	1	1	0	0	0	1	0	1	1	0	0	0	0	0	0	0	1	0	1	1	0	0	0	0	0	0	1	0	hydH	sensor kinase for HydG, hydrogenase 3 activity
b4004	1	1	1	1	1	1	1	1	1	1	1	1	0	1	1	1	1	1	1	1	1	1	1	1	0	1	0	0	0	1	1	1	1	1	1	1	1	1	1	1	1	1	1	hydG	response regulator of hydrogenase 3 activity (sensor HydH)
b4005	1	1	1	1	1	1	1	1	1	1	1	1	1	1	1	1	1	1	1	1	1	1	1	1	0	0	0	1	0	0	0	1	1	1	1	1	1	1	1	1	1	1	1	purD	phosphoribosylglycinamide synthetase = GAR synthetase
b4011	1	0	0	0	0	0	0	0	0	0	0	0	0	0	0	0	0	0	0	0	0	0	0	0	0	0	0	0	0	0	0	1	0	0	0	0	0	0	0	0	0	0	0	yjaA	orf, hypothetical protein

b4012	1	1	1	1	1	1	1	1	1	1	1	1	1	1	1	1	1	1	1	1	1	1	1	1	1	1	1	1	1	1	1	1	0	1	0	1	1	1	1	1	1	1	1	yjaB	orf, hypothetical protein
b4016	1	1	0	1	1	1	1	1	1	1	1	1	1	1	1	1	1	1	1	1	1	1	1	1	1	1	0	1	1	1	1	1	1	1	1	1	1	1	1	1	1	1	1	aceK	isocitrate dehydrogenase kinase/phosphatase
b4017	1	0	0	1	1	0	0	0	0	0	0	0	0	0	0	0	0	1	1	1	0	1	1	1	1	1	1	1	1	1	1	1	1	1	1	1	1	1	1	1	1	1	1	arp	regulator of acetyl CoA synthetase
b4018	1	1	1	1	1	0	1	1	1	1	1	1	1	1	1	1	1	1	1	1	1	1	1	1	1	1	1	1	1	1	1	1	0	1	1	1	1	1	1	1	1	1	1	iclR	repressor of aceBA operon
b4026	1	1	1	1	1	0	1	1	1	1	1	1	1	1	1	1	0	0	1	1	1	1	1	1	1	1	1	1	1	1	1	1	1	1	1	1	1	1	1	1	1	1	1	yjbE	orf, hypothetical protein
b4027	1	0	1	1	1	1	1	1	1	1	1	1	1	1	1	1	1	1	1	1	1	1	1	1	1	1	1	1	1	1	1	1	1	1	1	1	1	1	1	1	1	1	1	yjbF	orf, hypothetical protein
b4028	1	0	1	1	1	1	1	1	1	1	1	1	1	1	1	1	1	1	1	1	1	1	1	1	1	1	1	1	1	1	1	1	1	1	1	1	1	1	1	1	1	1	1	yjbG	orf, hypothetical protein
b4029	1	0	1	1	1	1	1	1	1	1	1	1	1	1	1	1	1	1	1	1	1	1	1	1	1	1	1	1	1	1	1	1	1	1	1	1	1	1	1	1	1	1	1	yjbH	orf, hypothetical protein
b4031	1	0	1	0	1	0	0	0	0	0	0	0	0	0	0	0	0	0	0	0	0	0	0	0	1	1	1	1	1	1	1	1	1	1	1	1	1	1	1	1	1	1	1	xylE	xylose-proton symport
b4032	1	1	1	1	1	1	0	1	0	1	0	1	0	0	0	0	1	0	0	1	1	0	1	1	1	1	1	1	1	1	1	1	1	1	1	1	1	1	1	1	1	1	1	malG	part of maltose permease, inner membrane
b4033	0	1	1	1	1	0	0	0	0	0	0	0	0	0	0	0	0	0	0	0	1	0	0	0	1	1	1	1	1	1	1	1	1	1	1	1	1	1	1	1	1	1	1	malF	part of maltose permease, periplasmic
b4034	0	1	1	0	1	0	0	0	0	0	0	0	0	0	0	0	0	0	0	0	0	0	0	0	1	1	1	1	1	1	1	1	1	1	1	1	1	1	1	1	1	1	1	malE	periplasmic maltose-binding protein; substrate recognition for transport and chemotaxis
b4035	0	1	1	1	1	1	1	1	1	1	1	1	0	0	1	1	1	1	0	1	1	1	1	1	1	1	1	1	1	1	1	1	1	1	1	1	1	1	1	1	1	1	1	malK	ATP-binding component of transport system for maltose
b4036	0	1	1	1	1	0	0	0	0	0	0	0	0	0	0	0	1	0	0	0	0	0	0	0	1	1	1	1	1	1	1	1	1	1	1	1	1	1	1	1	1	1	1	lamB	phage lambda receptor protein; maltose high-affinity receptor
b4037	0	1	1	1	1	0	1	0	0	0	0	0	1	0	0	0	1	0	0	0	1	1	1	1	1	1	1	1	1	1	1	1	1	1	1	1	1	1	1	1	1	1	1	malM	periplasmic protein of mal regulon
b4038	0	0	1	1	1	1	1	1	1	1	1	1	1	1	1	1	1	0	1	1	1	1	1	1	1	1	1	1	1	1	1	1	0	0	1	1	1	1	1	1	1	0	0	yjbI	orf, hypothetical protein
b4047	1	0	1	0	1	1	1	1	1	1	1	1	1	1	1	1	0	1	1	1	1	1	1	0	1	1	1	1	1	1	0	1	1	1	1	1	1	1	1	1	1	1	1	yjbL	orf, hypothetical protein
b4048	0	0	1	0	1	1	1	1	1	1	1	1	1	1	1	1	0	1	1	1	1	1	1	1	0	0	0	1	0	0	1	1	1	1	1	1	1	1	1	1	1	1	1	yjbM	orf, hypothetical protein
b4058	1	1	1	1	1	1	1	1	1	1	1	1	1	1	1	1	1	1	1	1	1	1	1	1	1	1	1	1	1	1	1	1	0	1	1	1	1	1	1	1	1	1	1	uvrA	excision nuclease subunit A
b4060	1	1	1	1	1	1	1	1	1	1	1	1	1	1	1	1	1	1	1	1	1	1	1	1	1	1	1	1	1	1	1	1	1	1	0	1	1	1	1	1	1	1	1	yjcB	orf, hypothetical protein
b4061	0	1	1	1	1	1	1	1	1	1	1	1	1	0	1	1	1	1	1	1	1	1	1	1	1	1	1	1	1	1	1	1	1	1	1	1	1	1	1	1	1	1	1	yjcC	orf, hypothetical protein
b4062	1	1	1	1	1	1	1	1	1	1	1	1	1	0	1	1	1	1	1	1	1	1	1	1	1	1	1	1	1	1	1	1	1	1	1	1	1	1	1	1	1	1	1	soxS	regulation of superoxide response regulon
b4063	1	1	1	1	1	1	1	1	1	1	1	1	1	0	1	1	1	1	1	1	1	1	1	1	1	1	1	1	1	1	1	1	1	1	1	1	1	1	1	1	1	1	1	soxR	redox-sensing activator of soxS
b4064	1	1	1	1	1	1	1	1	1	1	1	1	0	0	1	1	1	1	1	1	1	1	1	1	1	1	1	1	1	1	1	1	1	1	1	1	1	1	1	1	1	1	1	yjcD	orf, hypothetical protein
b4066	0	0	1	1	1	1	1	1	1	1	1	1	1	1	0	1	1	0	1	1	1	1	1	1	1	1	1	1	1	0	1	1	0	1	1	1	1	1	1	1	0	1	1	yjcF	orf, hypothetical protein
b4067	0	1	1	1	1	1	1	1	1	1	1	1	1	1	1	1	1	1	1	1	1	1	1	1	1	1	1	1	0	0	1	1	1	1	1	1	1	1	1	1	1	1	1	yjcG	putative transport protein
b4068	0	1	1	1	1	1	1	1	1	1	1	1	1	1	1	1	1	1	1	1	1	1	1	1	1	1	1	1	0	1	1	1	1	1	1	1	1	1	1	1	1	1	1	yjcH	orf, hypothetical protein
b4069	0	1	1	1	1	1	1	1	1	1	1	1	1	1	1	1	1	1	1	1	1	1	1	1	1	1	1	1	1	1	1	1	1	1	1	1	1	1	1	1	1	1	1	acs	acetyl-CoA synthetase
b4070	0	1	1	1	1	1	1	1	1	1	1	1	1	1	1	1	1	1	1	1	1	1	1	1	1	1	1	1	0	1	1	1	1	1	1	1	1	1	1	1	1	1	1	nrfA	periplasmic cytochrome c(552): plays a role in nitrite reduction
b4071	0	1	1	1	1	1	1	1	1	1	1	1	1	1	1	1	1	1	1	1	1	1	1	1	1	1	1	1	1	1	1	1	1	1	1	1	1	1	1	1	1	1	1	nrfB	formate-dependent nitrite reductase; a penta-haeme cytochrome c
b4072	0	1	1	1	1	1	1	1	1	1	1	1	1	1	1	1	1	1	1	1	1	1	1	1	1	1	1	1	1	1	1	1	1	1	1	1	1	1	1	0	1	1	1	nrfC	formate-dependent nitrite reductase; Fe-S centers
b4073	0	1	1	1	1	1	1	1	1	1	1	1	1	1	1	1	1	1	1	1	1	1	1	1	1	1	1	1	1	1	1	1	1	1	1	1	1	1	1	1	1	1	1	nrfD	formate-dependent nitrate reductase complex; transmembrane protein
b4074	0	1	1	1	1	0	1	0	0	1	1	1	1	1	0	0	1	1	1	1	1	1	1	1	0	0	1	0	0	1	0	1	0	1	1	0	0	0	0	0	0	0	0	nrfE	formate-dependent nitrite reductase; possible assembly function
b4075	0	1	1	1	1	1	1	1	1	1	1	1	1	1	1	1	1	1	1	1	1	1	1	1	1	1	1	1	1	1	1	1	1	1	1	1	1	1	1	1	1	1	1	nrfF	part of formate-dependent nitrite reductase complex
b4076	0	1	1	1	1	1	1	1	1	1	1	1	1	1	1	1	1	1	1	1	1	1	1	1	1	1	1	1	1	1	1	1	1	1	1	1	1	1	1	1	1	1	1	nrfG	part of formate-dependent nitrite reductase complex
b4077	0	1	1	1	1	1	1	1	1	1	1	1	1	1	1	1	1	1	1	1	1	1	1	1	1	1	1	1	1	1	1	1	1	1	1	1	1	1	1	1	1	1	1	gltP	glutamate-aspartate symport protein
b4078	0	1	1	1	1	0	0	0	0	1	0	0	1	1	1	1	1	1	1	1	1	1	1	1	1	1	1	1	1	1	1	1	1	1	1	1	1	1	1	1	1	1	1	yjcO	orf, hypothetical protein
b4079	0	1	1	1	1	0	0	0	0	0	0	0	1	1	1	1	1	1	1	1	1	1	1	1	1	1	1	1	1	1	1	1	1	1	1	1	1	1	1	1	1	1	1	fdhF	selenopolypeptide subunit of formate dehydrogenase H
b4080	0	1	1	1	1	0	0	1	1	1	1	1	1	1	1	1	1	1	1	1	1	1	1	1	1	1	1	1	1	1	1	1	1	1	1	1	1	1	1	1	1	1	1	yjcP	putative enzyme
b4081	0	1	1	1	1	0	0	0	0	0	0	0	1	1	1	1	1	1	1	1	1	1	1	1	1	1	1	1	1	1	1	1	1	1	1	1	1	1	1	1	1	1	1	yjcQ	putative enzyme
b4082	0	1	1	1	1	0	0	0	0	0	0	0	1	1	1	1	1	1	1	1	1	1	1	1	1	1	0	1	1	1	1	1	1	1	1	1	1	1	1	1	1	1	1	yjcR	putative membrane protein
b4083	0	1	1	1	1	0	0	0	0	0	0	0	0	1	1	1	1	1	1	1	1	1	1	1	1	1	1	1	1	1	1	1	0	0	0	0	0	0	0	0	0	1	1	yjcS	orf, hypothetical protein
b4084	0	0	0	0	1	0	0	0	0	1	0	0	0	0	0	0	1	0	0	0	1	0	1	1	0	0	0	0	0	0	0	1	0	0	0	0	0	0	0	0	0	0	0	yjcT	putative NAGC-like transcriptional regulator
b4085	0	0	0	0	1	0	0	0	0	0	0	0	0	0	0	0	0	0	0	0	0	0	0	0	0	0	0	0	0	0	0	0	0	0	0	0	0	0	0	0	0	0	0	yjcU	putative epimerase
b4086	0	0	0	0	1	0	0	0	0	0	0	0	0	0	0	0	0	0	0	0	0	0	0	0	0	0	0	0	0	0	0	0	0	0	0	0	0	0	0	0	0	0	0	yjcV	putative transport system permease protein
b4087	0	0	0	0	1	0	0	0	0	0	0	0	0	0	0	0	0	0	0	0	0	0	0	0	0	0	0	0	0	0	0	0	0	0	0	0	0	0	0	0	0	0	0	yjcW	putative ATP-binding component of a transport system
b4088	0	0	0	1	1	0	0	0	0	0	0	0	0	0	0	0	0	0	0	0	1	0	0	0	0	0	0	0	0	0	0	1	0	0	0	0	0	0	0	0	0	0	0	yjcX	putative LACI-type transcriptional regulator
b4089	0	0	0	0	1	0	0	0	0	0	0	0	0	0	0	0	0	0	0	0	0	0	0	0	0	0	0	0	0	0	0	0	0	0	0	0	0	0	0	0	0	0	0	rpiR	transcriptional repressor of rpiB expression
b4090	0	0	0	1	1	0	0	0	0	0	0	0	0	0	0	1	1	0	0	0	1	0	1	1	0	0	0	0	0	0	0	0	0	0	0	0	0	0	0	0	0	0	0	rpiB	ribose 5-phosphate isomerase B
b4091	1	1	1	1	1	0	0	0	0	0	0	0	1	1	1	1	1	0	0	0	1	1	1	1	1	1	1	1	1	1	1	1	0	0	0	0	1	0	0	0	1	1	1	phnQ	orf, hypothetical protein
b4092	1	1	1	0	1	1	1	1	1	0	1	1	1	1	1	1	1	1	1	1	1	1	1	1	1	1	1	1	1	1	1	1	0	0	0	0	0	1	1	1	1	1	1	phnP	phosphonate metabolism
b4093	1	0	1	1	1	0	0	0	0	0	0	0	0	1	1	1	1	0	0	0	1	0	1	1	1	1	1	1	1	1	0	0	0	0	0	0	0	0	0	0	1	1	1	phnO	putative regulator, phn operon
b4094	1	0	1	1	1	0	0	0	0	0	0	0	1	1	1	1	1	0	0	0	1	0	1	1	1	0	0	1	1	1	1	1	0	0	0	0	0	0	0	0	1	1	1	phnN	ATP-binding component of phosphonate transport
b4095	1	0	1	1	1	0	0	0	0	0	0	0	0	1	1	1	0	0	0	0	1	0	1	1	1	0	1	1	1	1	1	1	0	0	0	0	0	0	0	0	1	1	1	phnM	phosphonate metabolism
b4096	1	0	1	1	1	0	0	0	0	0	0	0	1	1	1	1	1	0	0	0	1	0	1	1	1	0	1	1	1	1	1	1	0	0	0	0	0	0	0	0	1	0	0	phnL	ATP-binding component of phosphonate transport
b4097	1	0	1	1	1	0	0	0	0	0	0	0	1	1	1	1	1	0	0	0	1	0	1	1	1	0	1	1	1	1	1	1	0	0	0	0	0	0	0	0	1	1	1	phnK	ATP-binding component of phosphonate transport
b4098	1	0	1	1	1	0	0	0	0	0	0	0	1	1	1	1	1	0	0	0	1	0	1	1	1	0	1	1	1	1	1	1	0	0	0	0	0	0	0	0	1	1	1	phnJ	phosphonate metabolism
b4099	1	1	1	1	1	1	1	1	1	0	1	1	1	1	1	1	1	1	1	0	1	1	1	1	1	1	1	1	0	1	1	1	1	0	0	1	1	1	1	1	1	1	1	phnI	phosphonate metabolism
b4100	1	0	1	1	1	1	1	0	1	0	1	1	1	1	1	0	1	0	0	0	1	0	1	1	1	0	1	1	1	1	0	1	0	0	0	0	0	0	0	0	0	1	1	phnH	phosphonate metabolism
b4101	1	1	1	1	1	1	1	1	1	1	1	1	1	1	1	1	1	1	1	1	1	1	1	1	1	0	1	1	0	1	1	1	0	0	0	1	1	1	1	1	1	1	1	phnG	phosphonate metabolism
b4102	1	1	1	1	1	0	0	1	0	1	1	1	0	1	1	1	1	1	1	1	1	1	1	1	1	0	1	1	1	1	1	1	0	0	0	0	0	0	0	1	1	1	1	phnF	putative transcriptional regulator
b4103	1	1	1	1	1	1	1	1	1	1	1	1	1	1	1	1	1	1	1	1	1	1	1	1	1	1	1	1	1	1	1	1	0	0	0	0	0	0	0	0	0	1	1	b4103	orf, hypothetical protein
b4104	1	0	1	1	1	1	1	1	1	1	1	1	1	1	1	1	1	1	1	1	1	1	1	1	1	1	1	1	1	1	1	1	0	0	0	0	0	0	0	0	1	1	1	phnE	membrane channel protein component of Pn transporter
b4105	1	0	1	1	1	1	1	1	1	1	1	1	1	1	1	1	1	1	1	1	1	1	1	1	1	1	1	1	1	1	1	1	1	1	1	1	1	1	1	1	1	1	1	phnD	periplasmic binding protein component of Pn transporter
b4106	1	0	1	1	1	1	1	1	1	1	1	1	1	1	1	1	1	1	1	1	1	1	1	1	1	1	1	1	1	1	1	1	1	1	1	1	1	1	1	1	1	1	1	phnC	ATP-binding component of phosphonate transport
b4109	1	0	1	1	1	0	0	0	0	0	0	0	1	1	1	1	1	1	1	1	1	1	1	1	1	1	1	1	1	0	1	1	1	1	1	1	1	1	1	1	1	0	0	yjdA	putative vimentin
b4111	1	1	1	1	1	0	0	0	0	0	0	0	1	1	1	1	1	1	1	1	1	1	1	1	1	1	1	1	1	1	1	1	1	1	1	1	1	1	1	1	1	1	1	proP	low-affinity transport system; proline permease II
b4112	1	1	1	1	1	0	0	0	0	0	0	0	0	1	1	1	1	1	0	1	1	1	1	1	1	1	1	1	1	1	1	1	1	1	1	1	1	1	1	1	1	1	1	basS	sensor protein for basR
b4113	1	1	1	1	1	0	0	0	0	0	0	0	1	1	1	1	1	1	1	1	1	1	1	1	1	1	1	1	1	1	0	0	1	1	1	1	1	1	1	1	1	1	1	basR	transcriptional regulatory protein, member of 2-component regulatory system,
b4114	1	1	1	1	1	0	0	0	0	0	0	0	1	1	1	1	1	1	1	1	1	1	1	1	1	1	1	1	1	1	1	1	1	1	1	1	1	1	1	1	1	1	1	yjdB	orf, hypothetical protein
b4118	1	0	1	1	1	1	1	1	1	1	1	1	1	1	1	1	1	0	1	1	1	1	1	1	1	0	1	1	1	1	1	1	1	1	1	1	1	1	1	1	1	1	1	melR	regulator of melibiose operon
b4119	1	0	1	1	1	1	1	1	1	1	1	1	1	1	1	1	1	0	0	0	0	0	0	0	1	0	1	1	1	1	1	1	1	1	1	1	1	1	1	1	1	1	1	melA	alpha-galactosidase
b4120	1	0	1	1	1	0	0	0	0	0	0	0	0	0	0	0	0	0	0	0	0	0	0	0	1	1	1	1	1	1	1	1	1	1	1	1	1	1	1	1	1	1	1	melB	melibiose permease II
b4121	1	1	1	1	1	0	0	0	0	0	0	0	1	1	1	1	0	0	0	0	0	0	0	0	1	1	1	1	1	1	1	1	1	1	1	1	1	1	1	1	1	1	1	yjdF	orf, hypothetical protein
b4122	1	1	1	1	1	0	0	0	0	0	0	0	1	1	1	1	1	0	0	1	1	1	1	1	1	1	1	1	1	1	1	1	1	1	1	1	1	1	1	1	1	1	1	fumB	fumarase B= fumarate hydratase Class I; anaerobic isozyme
b4123	1	1	1	1	1	0	0	0	0	0	0	0	1	1	1	1	0	0	0	0	0	0	0	0	1	1	1	1	1	1	1	1	1	1	1	1	1	1	1	1	1	1	1	dcuB	anaerobic dicarboxylate transport
b4124	1	0	1	1	1	1	0	1	1	0	1	1	1	1	1	1	0	1	1	1	1	0	0	0	1	1	1	1	1	1	1	1	1	1	1	1	1	1	1	1	1	1	1	yjdG	putative 2-component transcriptional regulator
b4125	1	1	1	1	1	0	0	0	0	0	0	0	1	1	1	1	0	0	0	0	0	0	0	0	1	1	1	1	1	1	1	1	1	1	1	1	1	1	1	1	1	1	1	yjdH	putative 2-component sensor protein
b4126	1	1	1	1	1	0	0	0	0	0	0	0	1	1	1	1	0	0	0	0	0	0	0	0	0	1	1	1	0	1	1	1	1	1	1	1	1	1	1	1	1	1	1	yjdI	orf, hypothetical protein
b4127	1	1	1	1	1	0	0	0	0	0	0	0	1	1	1	1	0	0	0	0	0	0	0	0	0	1	1	1	0	1	1	1	0	0	0	0	0	0	0	0	1	1	1	yjdJ	orf, hypothetical protein
b4128	1	1	1	1	1	1	1	1	1	1	1	1	1	0	1	1	0	0	0	0	0	0	1	0	0	1	1	1	0	1	1	1	0	0	0	0	0	0	0	0	1	0	0	yjdK	orf, hypothetical protein
b4129	1	1	1	1	1	1	1	1	1	1	1	1	1	1	0	1	1	0	0	0	1	1	1	1	1	1	1	1	1	1	1	1	1	1	1	0	1	1	1	1	1	1	1	lysU	lysine tRNA synthetase, inducible; heat shock protein
b4131	1	1	1	0	1	0	0	0	0	0	0	0	0	0	0	0	0	0	0	0	0	0	0	0	0	1	0	0	0	0	0	0	0	1	0	0	0	0	0	0	0	0	0	cadA	lysine decarboxylase 1
b4132	1	1	1	0	1	1	0	1	1	0	1	1	1	0	0	1	1	0	0	0	1	0	1	0	1	1	1	1	1	1	1	1	1	1	0	1	1	1	1	1	0	1	0	cadB	transport of lysine/cadaverine
b4133	0	0	1	0	1	0	0	0	0	0	0	0	0	0	0	0	0	0	0	0	0	0	0	0	0	1	0	0	0	0	0	0	0	0	0	0	0	0	0	0	0	0	0	cadC	transcriptional activator of cad operon
b4135	1	1	1	1	1	1	1	0	0	1	1	1	1	1	1	0	1	1	1	1	1	1	0	1	1	1	0	0	0	0	0	1	0	1	1	1	1	1	1	1	0	1	1	yjdC	orf, hypothetical protein
b4150	1	1	1	1	1	1	1	1	1	1	1	1	1	1	1	1	1	1	1	1	1	1	1	1	1	1	0	1	1	1	1	1	1	1	1	1	1	1	1	1	1	1	1	ampC	beta-lactamase; penicillin resistance
b4152	1	1	1	1	1	1	1	1	1	1	1	1	1	1	1	1	1	1	1	1	1	1	0	1	1	1	1	1	1	1	1	1	1	1	1	1	1	1	1	1	1	1	1	frdC	fumarate reductase, anaerobic, membrane anchor polypeptide
b4153	1	1	1	1	1	1	1	1	1	1	1	1	1	1	1	1	1	1	1	1	1	1	0	1	1	1	1	1	1	1	1	1	1	1	1	1	1	1	1	1	1	1	1	frdB	fumarate reductase, anaerobic, iron-sulfur protein subunit
b4156	1	1	1	1	1	1	0	1	1	1	1	1	1	1	1	1	1	1	1	1	1	1	0	1	1	1	1	1	1	1	1	1	1	1	1	1	1	1	1	1	1	1	1	yjeM	putative transport
b4157	0	1	0	0	1	0	0	0	0	0	0	0	0	0	0	0	1	0	0	0	1	0	1	1	0	0	0	0	0	0	0	1	0	0	0	0	0	0	0	0	0	0	0	yjeN	orf, hypothetical protein
b4158	0	1	0	0	1	0	0	0	0	0	0	0	0	0	0	0	0	0	0	0	0	0	0	0	0	0	0	0	0	0	0	0	0	0	0	0	0	0	0	0	0	0	0	yjeO	orf, hypothetical protein
b4173	1	1	1	1	1	1	1	1	1	1	1	1	1	1	1	1	1	1	1	1	1	1	0	1	1	1	1	1	1	1	1	1	1	1	1	1	1	1	1	1	1	1	1	hflX	GTP - binding subunit of protease specific for phage lambda cII repressor
b4178	1	1	1	1	1	1	1	1	1	1	1	1	1	1	1	1	1	1	1	1	1	1	0	1	1	1	1	1	1	1	1	1	1	1	1	1	1	1	1	1	1	1	1	yjeB	orf, hypothetical protein
b4179	1	1	1	1	1	1	1	1	1	1	1	1	1	1	1	1	1	1	1	1	1	1	0	1	1	1	1	1	1	1	1	1	1	1	1	1	1	1	1	1	1	1	1	vacB	putative enzyme
b4180	1	1	1	1	1	1	1	1	1	1	1	1	1	1	1	1	1	1	1	1	1	1	0	1	1	1	1	1	1	1	1	1	1	1	1	1	1	1	1	1	1	1	1	yjfH	orf, hypothetical protein
b4181	0	0	0	1	1	0	0	0	0	0	0	0	1	1	1	1	0	1	1	1	1	1	1	1	1	1	1	1	1	1	1	1	0	0	0	0	0	0	0	0	0	0	0	yjfI	orf, hypothetical protein
b4182	0	0	0	1	1	0	0	0	0	0	0	0	1	1	1	1	1	1	1	1	1	1	1	1	1	1	1	1	1	1	1	1	0	0	0	0	0	0	0	0	0	0	0	yjfJ	putative alpha helical protein
b4183	0	0	0	1	1	0	0	0	0	0	0	0	1	1	1	1	0	1	1	1	1	1	1	1	1	1	1	1	1	1	1	1	0	0	0	0	0	0	0	0	0	0	0	yjfK	orf, hypothetical protein
b4184	0	1	0	1	1	1	0	0	0	1	1	1	0	1	1	1	1	1	1	1	1	1	1	1	0	0	1	0	1	1	1	1	1	1	1	1	1	1	1	1	1	1	1	yjfL	orf, hypothetical protein
b4186	1	1	1	1	1	1	1	1	1	1	1	1	1	1	1	1	1	1	1	1	1	1	0	1	1	1	1	1	1	1	1	1	1	1	1	1	1	1	1	1	1	1	1	yjfC	putative synthetase/amidase
b4187	1	1	1	1	1	1	1	1	1	1	1	1	1	1	1	1	1	1	1	1	1	1	0	1	1	1	0	1	1	0	1	1	0	1	1	1	1	1	1	1	1	1	1	aidB	putative acyl coenzyme A dehydrogenase
b4188	1	1	1	1	1	1	1	1	1	1	1	1	1	1	1	1	1	1	1	1	1	1	1	1	1	1	1	1	1	0	1	1	1	1	1	1	1	1	1	1	1	1	1	yjfN	orf, hypothetical protein
b4190	1	1	1	1	1	1	1	1	1	1	1	1	1	1	1	1	1	1	1	1	1	1	0	1	1	1	1	1	1	1	1	1	1	1	1	1	1	1	1	1	1	1	1	yjfP	orf, hypothetical protein
b4192	1	1	1	1	1	1	1	1	1	1	1	1	1	1	1	1	1	1	1	1	1	1	0	1	1	1	1	1	1	1	1	1	1	1	1	1	1	1	1	1	1	1	1	yjfR	orf, hypothetical protein
b4193	1	1	1	1	1	1	1	1	1	1	1	1	1	1	1	1	1	1	1	1	1	1	1	1	1	1	1	1	1	0	1	1	0	1	1	1	1	1	1	1	0	1	1	sgaT	orf, hypothetical protein

b4194	1	1	1	1	1	1	1	1	1	1	1	1	1	1	1	1	1	1	1	1	1	1	1	1	1	1	1	1	1	0	1	1	1	1	1	1	1	1	1	1	1	1	1	sgaB	orf, hypothetical protein
b4195	1	1	1	1	1	0	0	0	0	1	1	1	0	1	1	1	1	0	1	1	1	0	1	1	1	1	1	1	1	0	1	1	0	1	1	1	1	1	1	1	1	1	1	ptxA	putative PTS system enzyme II A component
b4196	1	1	1	1	1	0	1	1	1	1	1	1	1	1	1	1	1	1	1	1	1	1	1	1	1	1	1	1	1	0	1	1	1	1	1	1	1	1	1	1	1	1	1	sgaH	probable hexulose-6-phosphate synthase
b4197	1	1	1	1	1	0	0	0	1	1	1	1	1	1	1	1	1	1	1	1	1	1	1	1	0	0	0	1	0	0	1	1	0	1	1	1	1	1	1	1	0	1	1	sgaU	putative hexulose-6-phosphate isomerase
b4198	1	1	1	1	1	1	1	1	1	1	1	1	1	1	1	1	1	1	1	1	1	1	1	1	1	1	1	1	1	0	1	1	1	1	1	1	1	1	1	1	1	1	1	sgaE	putative epimerase/aldolase
b4204	1	0	0	1	0	1	1	1	1	1	1	1	1	1	1	1	1	1	1	1	1	1	1	1	1	1	1	1	1	1	1	1	1	1	1	1	1	1	1	1	1	1	1	yjfZ	orf, hypothetical protein
b4205	1	1	1	1	1	1	1	1	1	1	1	1	1	1	1	1	1	1	1	1	1	1	1	1	1	0	1	1	0	0	0	1	0	1	1	1	1	1	1	1	0	1	1	ytfA	orf, hypothetical protein
b4206	1	1	1	1	1	1	1	1	1	1	1	1	1	1	1	1	1	1	1	1	1	1	1	1	1	1	1	1	1	1	1	1	1	0	0	1	1	1	1	1	0	1	1	ytfB	orf, hypothetical protein
b4208	1	1	1	1	1	1	1	1	1	1	1	1	1	1	1	1	1	1	1	1	1	1	0	1	1	1	1	1	1	1	1	1	1	1	1	1	1	1	1	0	1	1	1	cycA	transport of D-alanine, D-serine, and glycine
b4211	1	0	1	1	1	1	1	1	1	1	1	1	1	1	1	1	1	1	1	1	1	1	1	1	1	1	1	1	1	1	1	1	1	1	1	1	1	1	1	1	1	1	1	ytfG	putative oxidoreductase
b4213	1	1	1	1	1	1	1	1	1	1	1	1	1	1	1	1	1	1	1	1	1	1	0	1	1	1	1	1	1	1	1	1	1	1	1	0	1	1	1	1	1	1	1	cpdB	2':3'-cyclic-nucleotide 2'-phosphodiesterase
b4215	0	0	1	1	0	1	1	1	1	1	1	1	1	1	1	1	1	1	1	1	1	1	1	1	0	0	1	1	1	0	1	1	0	1	1	1	1	1	1	1	1	1	1	ytfI	orf, hypothetical protein
b4224	1	0	0	1	0	0	0	0	0	0	0	0	0	0	0	0	0	0	0	0	0	0	0	0	1	1	1	1	0	1	1	1	0	0	0	0	0	0	0	0	0	0	0	chpS	suppressor of inhibitory function of ChpB, PemI-like, autoregulated
b4225	1	1	0	1	1	1	1	0	0	1	1	1	1	0	1	0	1	1	1	1	1	1	1	1	1	1	1	1	1	1	1	1	0	0	0	1	1	1	1	1	1	1	1	chpB	probable growth inhibitor, PemK-like, autoregulated
b4228	1	1	1	1	1	1	1	1	1	1	1	1	1	1	1	1	1	1	1	1	1	1	1	1	1	1	1	1	1	1	1	1	0	1	1	1	1	1	1	1	1	1	1	ytfR	putative ATP-binding component of a transport system
b4229	1	1	1	1	1	0	1	1	1	1	1	1	1	1	1	1	1	1	1	1	1	1	1	1	0	1	0	1	0	1	1	1	0	1	1	1	1	1	1	0	0	1	1	ytfS	putative ATP-binding component of a transport system
b4231	1	1	1	1	1	0	1	1	1	1	1	1	1	1	1	1	0	1	1	1	1	1	0	1	1	1	1	1	1	1	1	1	1	1	1	1	1	1	1	1	0	1	1	yjfF	putative transport system permease protein
b4235	1	1	1	1	1	1	1	1	1	1	1	1	1	1	1	1	1	1	1	1	1	1	0	1	1	1	1	1	1	1	1	1	1	1	1	1	1	1	1	1	1	1	1	pmbA	maturation of antibiotic MccB17, see tld genes
b4238	1	1	1	1	1	1	1	1	1	1	1	1	1	1	1	1	1	1	1	1	1	1	0	1	1	1	1	1	1	1	1	1	1	1	1	1	1	1	1	1	1	1	1	nrdD	anaerobic ribonucleoside-triphosphate reductase
b4239	1	1	1	0	1	1	1	1	1	1	1	1	1	1	1	1	1	1	1	1	1	1	1	1	1	1	1	1	1	1	1	1	1	1	1	1	1	1	1	1	1	1	1	treC	trehalase 6-P hydrolase
b4240	1	1	1	0	1	1	1	1	1	1	1	1	1	1	1	1	1	1	1	1	1	1	1	1	1	1	1	1	1	1	1	1	1	1	1	1	1	1	1	1	1	1	1	treB	PTS system enzyme II, trehalose specific
b4245	1	1	1	1	1	1	1	1	1	1	1	1	1	1	1	1	1	1	1	1	1	1	0	1	1	1	1	1	1	1	1	1	1	1	1	1	1	1	1	1	1	1	1	pyrB	aspartate carbamoyltransferase, catalytic subunit
b4247	1	0	1	1	1	1	0	1	1	1	1	1	0	1	1	1	1	1	1	1	1	1	1	1	1	1	1	1	1	1	1	1	1	1	1	1	1	1	1	1	1	1	1	yjgG	orf, hypothetical protein
b4248	1	0	1	1	1	1	1	1	1	1	1	1	1	1	1	1	1	1	1	1	1	1	1	1	1	1	1	1	1	1	1	1	1	1	1	1	1	1	1	1	1	1	1	yjgH	orf, hypothetical protein
b4249	1	0	1	1	1	1	1	1	1	1	1	1	1	1	1	1	1	1	1	1	1	1	1	1	1	1	1	1	1	1	1	1	1	1	1	1	1	1	1	1	1	1	1	yjgI	putative oxidoreductase
b4250	1	0	1	1	1	1	1	1	1	1	1	1	1	1	1	1	1	1	1	1	1	1	1	1	1	1	1	1	1	1	1	1	1	1	1	1	1	1	1	1	1	1	1	b4250	orf, hypothetical protein
b4251	1	0	1	1	1	1	1	0	1	1	1	1	1	1	1	1	1	1	1	1	1	1	1	1	1	1	1	1	1	1	1	1	1	1	1	1	1	1	1	1	1	1	1	yjgJ	orf, hypothetical protein
b4253	0	0	1	1	0	1	1	1	1	1	1	1	1	1	1	0	1	0	1	1	1	1	1	1	1	1	1	1	1	1	1	1	1	1	1	1	1	1	1	0	1	1	1	yjgL	orf, hypothetical protein
b4254	1	1	1	1	1	1	0	0	0	1	1	1	0	1	1	1	1	1	1	1	1	1	0	1	1	0	0	1	0	0	0	1	0	1	1	1	1	1	1	1	1	1	1	argI	ornithine carbamoyltransferase 1
b4257	1	0	1	1	1	1	1	1	1	1	1	1	1	1	1	1	1	0	0	0	0	0	0	0	1	1	1	1	1	1	1	1	1	1	1	1	1	1	1	1	0	1	1	yjgN	orf, hypothetical protein
b4261	1	1	1	1	1	1	1	1	1	1	1	1	1	1	1	1	1	1	1	1	1	1	0	1	1	1	1	1	1	1	1	1	1	1	1	1	1	1	1	1	1	1	1	yjgP	orf, hypothetical protein
b4262	1	1	1	1	1	1	1	1	1	1	1	1	1	1	1	1	1	1	1	1	1	1	0	1	1	1	1	1	1	1	1	1	1	1	1	1	1	1	1	1	1	1	1	yjgQ	orf, hypothetical protein
b4263	1	1	1	1	1	1	1	1	1	1	1	1	1	1	1	1	1	1	1	1	1	1	1	1	1	0	0	1	0	0	1	1	1	1	1	1	1	1	1	0	1	1	1	yjgR	orf, hypothetical protein
b4264	0	0	1	0	0	0	0	0	0	0	0	0	0	0	0	0	0	0	0	0	0	0	0	0	0	0	0	0	0	0	0	0	0	0	0	0	0	0	0	0	0	0	0	idnR	L-idonate transcriptional regulator
b4265	0	0	1	0	0	0	0	0	0	0	0	0	0	0	0	0	0	0	0	0	0	0	0	0	0	0	0	0	0	0	0	0	0	0	0	0	0	0	0	0	0	0	0	idnT	L-idonate transporter
b4266	0	0	1	0	0	0	0	0	0	0	0	0	0	0	0	0	0	0	0	0	0	0	0	0	0	0	0	0	0	0	0	0	0	0	0	0	0	0	0	0	0	0	0	idnO	5-keto-D-gluconate 5-reductase
b4267	0	0	1	1	0	0	0	0	0	0	0	0	0	0	0	0	0	0	0	0	0	0	0	0	0	0	0	0	0	0	0	0	0	0	0	0	0	0	0	0	0	0	0	idnD	L-idonate dehydrogenase
b4268	0	0	1	1	0	0	0	0	0	0	0	0	0	0	0	0	0	0	0	0	0	0	0	0	0	0	0	0	0	0	0	0	0	0	0	0	0	0	0	0	0	0	0	idnK	gluconate kinase, thermosensitive glucokinase
b4269	1	1	1	1	0	1	1	1	1	1	1	1	1	1	1	1	1	1	1	1	1	1	0	1	1	1	1	1	1	1	1	1	1	1	1	1	1	1	1	1	1	1	1	yjgB	putative oxidoreductase
b4271	0	0	0	0	0	0	0	0	0	0	0	1	0	1	0	0	0	0	0	0	0	0	0	0	0	0	0	0	0	0	0	0	0	0	0	0	0	0	0	0	0	0	0	intB	prophage P4 integrase
b4274	0	0	0	1	0	0	1	1	1	1	1	1	1	0	1	1	1	1	1	1	1	1	0	1	1	1	1	1	1	1	1	1	1	0	0	1	1	1	0	1	0	1	1	yjgW	orf, hypothetical protein
b4275	0	0	1	0	0	0	0	0	0	0	0	0	0	0	0	0	0	0	0	0	0	0	0	0	0	0	0	0	0	0	0	0	0	0	0	0	0	0	0	0	0	0	0	yjgX	orf, hypothetical protein
b4276	0	0	0	0	0	0	0	0	0	0	0	0	0	0	0	0	0	0	0	0	0	0	0	0	0	0	0	0	0	0	0	0	0	0	0	0	0	0	0	0	0	0	0	yjgY	orf, hypothetical protein
b4277	0	0	0	0	0	0	0	0	0	0	0	0	1	0	0	0	0	0	0	1	1	0	1	1	1	0	0	0	0	0	1	1	0	0	0	0	0	0	0	0	0	0	0	yjgZ	orf, hypothetical protein
b4278	1	0	1	0	1	1	1	1	1	1	1	1	0	1	1	1	1	1	1	1	1	1	1	1	1	1	1	1	1	1	1	1	1	1	1	1	1	1	1	1	1	1	1	yi41	IS4 hypothetical protein
b4279	0	0	0	0	0	0	0	0	0	0	0	0	0	0	0	0	0	0	0	0	0	0	0	0	0	0	0	0	0	0	0	0	0	0	0	0	0	0	0	0	0	0	0	yjhB	putative transport protein
b4280	0	1	0	1	1	1	0	1	1	1	1	1	1	0	1	1	1	1	1	1	1	1	1	1	0	1	0	0	1	1	1	1	1	0	0	1	1	1	1	1	0	0	1	yjhC	putative dehydrogenase
b4281	0	0	0	0	0	0	0	0	0	0	0	0	0	0	0	0	0	0	0	0	0	0	0	0	0	0	0	0	0	0	0	0	0	0	0	0	0	0	0	0	0	0	0	yjhD	orf, hypothetical protein
b4282	0	0	0	0	0	0	0	0	0	0	0	0	0	0	0	0	0	0	0	0	0	0	0	0	0	0	0	0	0	0	0	1	0	0	0	0	0	0	0	0	0	0	0	yjhE	orf, hypothetical protein
b4285	1	0	1	0	1	1	1	1	1	1	1	1	1	1	1	1	1	1	1	1	1	1	1	1	1	0	1	1	1	1	1	1	1	1	1	1	1	1	1	1	1	1	1	b4285	putative transposase
b4286	1	0	0	0	0	1	0	0	0	1	1	1	0	0	0	0	1	0	0	1	1	1	1	0	0	1	1	1	0	0	0	1	0	0	0	0	0	0	0	0	0	0	0	b4286	orf, hypothetical protein
b4287	1	1	1	0	0	0	0	0	0	1	0	0	0	0	0	0	0	1	0	0	0	0	0	0	0	1	1	1	0	0	0	1	0	0	0	0	0	0	0	0	0	0	0	fecE	ATP-binding component of citrate-dependent iron(III) transport protein
b4288	1	0	1	0	0	0	0	0	0	0	0	0	0	0	0	0	0	0	0	0	0	0	0	0	0	1	1	1	0	0	0	1	0	0	0	0	0	0	0	0	0	0	0	fecD	citrate-dependent iron transport, membrane-bound protein
b4289	1	0	1	0	0	0	0	0	0	0	0	0	0	0	0	0	0	0	0	0	0	0	0	0	0	1	1	1	0	0	0	1	0	0	0	0	0	0	0	0	0	0	0	fecC	citrate-dependent iron(III) transport protein, cytosolic
b4290	1	0	1	0	0	0	0	0	0	0	0	0	0	0	0	0	0	0	0	0	0	0	0	0	0	1	1	1	0	0	0	1	0	0	0	0	0	0	0	0	0	0	0	fecB	citrate-dependent iron transport, periplasmic protein
b4291	1	1	1	1	1	1	1	1	1	1	1	1	1	0	1	1	1	1	1	1	1	1	1	1	1	1	1	1	1	1	1	1	1	0	0	1	1	1	1	1	1	1	1	fecA	outer membrane receptor; citrate-dependent iron transport, outer membrane receptor
b4292	1	0	1	0	0	0	0	0	0	0	0	0	0	0	0	0	0	0	0	0	0	0	0	0	0	1	1	1	0	0	0	1	0	0	0	0	0	0	0	0	0	0	0	fecR	regulator for fec operon, periplasmic
b4293	1	0	1	0	0	0	0	0	0	0	0	0	0	0	0	0	0	0	0	0	0	0	0	0	0	1	1	1	0	0	0	1	0	0	0	0	0	0	0	0	0	0	0	fecI	probable RNA polymerase sigma factor
b4295	0	0	0	0	0	0	0	0	0	0	0	0	0	0	0	0	0	0	0	0	0	0	0	0	0	0	0	0	0	0	0	0	0	0	0	0	0	0	0	0	0	0	0	yjhU	orf, hypothetical protein
b4296	0	0	0	0	0	0	0	0	0	0	0	0	0	0	0	0	0	0	0	0	0	0	0	0	0	0	0	0	0	0	0	0	0	0	0	0	0	0	0	0	0	0	0	yjhF	putative transport system permease
b4297	0	0	0	0	0	0	0	0	0	0	0	0	0	0	0	0	0	0	0	0	1	0	0	0	0	0	0	0	0	0	0	0	0	0	0	0	0	0	0	0	0	0	0	yjhG	putative dehydratase
b4298	0	0	0	0	0	0	0	0	0	0	0	0	0	0	0	0	0	0	0	0	0	0	0	0	0	0	0	0	0	0	0	0	0	0	0	0	0	0	0	0	0	0	0	yjhH	putative lyase/synthase
b4299	0	0	0	0	0	0	0	0	0	0	0	0	0	0	0	0	0	0	0	0	0	0	0	0	0	0	0	0	0	0	0	0	0	0	0	0	0	0	0	0	0	0	0	yjhI	putative regulator
b4300	0	0	0	0	0	0	0	0	0	0	0	0	0	0	0	0	0	0	0	0	0	0	0	0	0	0	0	0	0	0	0	0	0	0	0	0	0	0	0	0	0	0	0	sgcR	putative DEOR-type transcriptional regulator
b4301	0	0	0	0	0	0	0	0	0	0	0	0	0	0	0	0	0	0	0	0	0	0	0	0	0	0	0	0	0	0	0	0	0	0	0	0	0	0	0	0	0	0	0	sgcE	putative epimerase
b4302	0	0	0	0	0	0	0	0	0	0	0	0	0	0	0	0	0	0	0	0	0	0	0	0	0	0	0	0	0	0	0	0	0	0	0	0	0	0	0	0	0	0	0	sgcA	putative PTS system enzyme II A component
b4303	0	0	0	0	0	0	0	0	0	0	0	0	0	0	0	0	0	0	0	0	0	0	0	0	0	0	0	0	0	0	0	0	0	0	0	0	0	0	0	0	0	0	0	sgcQ	putative nucleoside triphosphatase
b4304	0	0	0	0	0	0	0	0	0	0	0	0	0	0	0	0	0	0	0	0	0	0	0	0	0	0	0	0	0	0	0	0	0	0	0	0	0	0	0	0	0	0	0	sgcC	putative PTS system enzyme IIC component
b4305	0	0	0	0	0	0	0	0	0	0	0	0	0	0	0	0	0	0	0	0	0	0	0	0	0	0	0	0	0	0	0	0	0	0	0	0	0	0	0	0	0	0	0	sgcX	putative lyase/synthase
b4306	0	0	0	0	0	0	0	0	0	0	0	0	0	0	0	0	0	0	0	0	0	0	0	0	0	0	0	0	0	0	0	0	0	0	0	0	0	0	0	0	0	0	0	yjhP	putative methyltransferase
b4307	0	0	0	0	0	0	0	0	0	0	0	0	0	0	0	0	0	0	0	0	0	0	0	0	0	0	0	0	0	0	0	0	0	0	0	0	0	0	0	0	0	0	0	yjhQ	orf, hypothetical protein
b4308	0	0	1	0	0	0	0	0	0	1	0	0	0	0	0	0	1	0	0	0	1	1	1	1	0	0	0	0	0	0	0	1	0	0	1	0	0	0	0	0	0	0	0	yjhR	putative frameshift suppressor
b4309	0	0	1	1	1	0	0	0	0	0	0	0	1	1	1	1	1	0	0	0	1	0	1	0	1	0	1	1	1	1	1	0	1	1	1	1	1	1	1	1	1	1	1	yjhS	orf, hypothetical protein
b4310	0	1	1	1	1	0	0	0	0	0	0	0	1	1	1	1	1	0	0	0	1	0	1	0	0	0	1	1	0	0	1	0	1	1	1	1	1	1	1	1	1	1	1	yjhT	orf, hypothetical protein
b4311	0	1	1	1	1	1	0	1	1	1	1	1	1	1	1	1	1	1	1	1	1	1	1	1	1	1	1	1	1	0	1	1	1	1	1	1	1	1	1	1	1	1	1	yjhA	orf, hypothetical protein
b4312	0	1	0	0	1	0	0	0	0	0	0	0	0	1	1	1	0	0	0	0	1	0	1	0	1	0	1	1	0	0	1	0	1	1	1	1	1	1	1	1	1	1	0	fimB	recombinase involved in phase variation; regulator for fimA
b4313	0	1	0	0	1	0	0	0	0	0	0	0	0	1	1	1	0	0	0	0	1	0	1	0	1	0	1	1	0	0	1	0	1	1	1	1	1	1	1	1	1	1	1	fimE	recombinase involved in phase variation; regulator for fimA
b4314	0	1	0	0	0	0	0	0	0	0	0	0	0	1	1	1	0	0	0	0	1	0	1	1	1	0	1	1	0	0	1	0	1	1	1	1	1	1	1	1	0	1	1	fimA	major type 1 subunit fimbrin (pilin)
b4315	0	1	0	0	0	0	1	1	1	1	1	1	0	1	1	1	0	0	0	0	1	0	1	1	1	0	1	1	0	0	1	0	1	1	1	1	1	1	1	1	1	1	1	fimI	fimbrial protein
b4316	0	1	0	1	0	0	1	1	1	0	1	1	0	1	1	1	0	0	0	0	1	0	1	1	1	0	1	1	0	0	1	0	1	1	1	1	1	1	1	1	0	1	1	fimC	periplasmic chaperone, required for type 1 fimbriae
b4317	0	1	0	1	1	0	1	1	1	0	1	1	0	1	1	1	0	0	0	0	1	0	1	1	1	0	1	1	0	0	1	0	1	1	1	1	1	1	1	1	1	1	1	fimD	outer membrane protein; export and assembly of type 1 fimbriae, interrupted
b4318	0	1	0	1	1	0	1	1	1	1	1	1	0	1	1	1	0	0	0	0	1	0	1	1	1	1	1	1	1	0	1	0	1	1	1	1	1	1	1	1	1	1	1	fimF	fimbrial morphology
b4319	0	1	0	1	1	0	1	1	1	1	1	1	1	1	1	1	0	0	0	0	1	0	1	1	1	1	1	1	1	0	1	0	1	1	1	1	1	1	1	1	1	1	1	fimG	fimbrial morphology
b4320	0	1	0	1	1	0	1	1	1	1	1	1	1	1	1	1	0	0	0	0	1	0	1	1	1	1	1	1	1	0	1	0	1	1	1	1	1	1	1	1	1	1	1	fimH	minor fimbrial subunit, D-mannose specific adhesin
b4321	0	1	1	1	0	0	0	0	0	0	0	1	0	0	0	0	0	0	0	0	1	0	1	1	1	1	1	1	1	0	1	0	1	1	1	1	1	1	1	1	1	1	1	gntP	gluconate transport system permease 3
b4322	0	1	1	1	1	0	1	1	1	1	1	1	0	1	1	1	0	0	0	0	1	0	1	1	1	1	1	1	1	0	1	0	1	1	1	1	1	1	1	1	1	1	1	uxuA	mannonate hydrolase
b4323	0	1	1	1	1	0	0	0	0	1	1	1	1	1	1	1	0	1	1	1	1	1	1	1	1	1	1	1	0	1	1	1	1	1	1	1	1	1	1	1	0	1	1	uxuB	D-mannonate oxidoreductase
b4324	0	1	1	1	1	0	1	1	1	1	1	1	0	1	1	1	0	0	0	0	1	0	0	1	0	0	1	1	1	0	1	0	1	1	1	1	1	1	1	1	0	1	1	uxuR	regulator for uxu operon
b4325	0	0	1	1	1	1	1	1	1	1	1	1	0	1	1	1	0	0	0	0	1	0	0	1	1	1	1	1	1	0	1	0	0	1	1	1	1	1	1	1	1	1	1	yjiC	orf, hypothetical protein
b4326	0	1	0	1	1	1	1	1	1	1	1	1	0	1	1	1	1	0	0	0	1	0	0	1	1	1	1	1	1	1	1	0	1	1	1	1	1	1	1	1	1	1	1	yjiD	orf, hypothetical protein
b4327	0	0	0	1	1	1	1	1	1	1	1	1	0	1	1	1	1	0	0	0	1	0	0	1	1	1	0	1	0	1	0	0	0	1	1	1	1	1	1	1	1	1	1	yjiE	putative transcriptional regulator LYSR-type
b4328	0	1	0	1	1	1	1	1	1	1	1	1	0	1	1	1	1	0	0	0	1	0	0	1	1	1	1	1	1	1	1	0	1	1	1	1	1	1	1	1	1	1	1	iadA	isoaspartyl dipeptidase
b4329	0	1	0	1	1	1	0	0	1	1	1	1	0	1	1	1	1	0	0	0	1	0	1	1	1	1	1	1	1	1	1	0	1	1	1	1	1	1	1	1	1	1	1	yjiG	orf, hypothetical protein
b4330	0	1	0	1	1	1	1	1	1	1	1	1	0	1	1	1	1	0	0	0	1	0	0	1	1	1	1	1	1	1	1	0	1	1	1	1	1	1	1	1	1	1	1	yjiH	orf, hypothetical protein
b4331	0	0	0	1	1	1	1	1	1	1	1	1	0	1	1	1	1	0	0	0	1	0	0	1	1	1	1	1	1	1	1	0	1	1	1	1	1	1	1	1	1	1	1	yjiI	orf, hypothetical protein
b4332	0	0	0	1	1	1	1	1	1	1	1	1	0	1	1	1	1	0	0	0	1	0	0	1	1	1	1	1	1	1	1	0	0	0	0	0	0	0	0	0	0	0	0	yjiJ	putative transport protein
b4333	1	0	0	1	1	1	1	1	1	1	1	1	0	1	1	1	1	0	0	0	1	0	0	1	1	1	1	1	1	1	1	0	0	0	0	0	0	0	0	0	0	0	0	yjiK	orf, hypothetical protein
b4334	1	1	0	1	1	1	1	1	1	1	1	1	0	1	1	1	1	0	0	0	1	0	0	1	1	1	1	1	1	1	1	0	0	0	0	0	0	0	0	0	0	0	0	yjiL	putative enzyme
b4335	1	1	0	1	1	1	1	1	1	1	1	1	0	1	1	1	1	0	0	0	1	0	0	1	1	1	1	1	1	1	1	0	0	0	0	0	0	0	0	0	0	0	0	yjiM	orf, hypothetical protein
b4336	1	1	0	1	1	1	1	1	1	1	1	1	0	1	1	1	1	0	0	0	1	0	0	1	1	1	1	1	1	1	1	0	0	0	0	0	0	0	0	0	0	0	0	yjiN	orf, hypothetical protein
b4337	1	0	0	1	1	1	1	1	1	1	1	1	0	1	1	1	1	1	1	1	1	0	0	1	1	1	1	1	1	1	1	0	0	0	0	0	0	0	0	0	0	0	0	yjiO	putative transport protein
b4339	1	1	0	1	1	1	1	1	1	1	1	1	0	1	1	1	1	0	1	1	1	0	0	1	1	0	1	1	1	0	0	0	0	0	0	0	0	0	0	0	0	0	0	yjiQ	orf, hypothetical protein
b4340	1	0	0	1	1	1	1	1	1	1	1	1	0	0	0	0	1	0	0	0	1	0	0	1	1	1	1	1	1	0	1	1	0	0	0	0	0	0	0	0	0	0	0	yjiR	putative regulator
b4341	1	1	0	1	1	1	1	0	0	1	1	1	0	0	1	1	0	1	1	1	1	0	0	0	1	1	1	1	1	0	1	0	0	0	0	0	0	0	0	0	0	0	0	yjiS	orf, hypothetical protein
b4342	0	0	0	0	1	0	0	1	0	0	0	0	0	0	1	0	1	0	0	1	1	1	1	1	0	0	0	0	0	0	1	1	0	0	0	0	0	0	0	0	0	0	0	yjiT	orf, hypothetical protein
b4343	0	0	0	0	1	0	0	0	0	0	0	0	0	0	0	0	0	0	0	0	0	0	0	0	0	1	0	0	0	0	0	0	0	0	0	0	0	0	0	0	0	0	0	yjiU	orf, hypothetical protein

b4344	0	0	0	0	0	0	0	0	0	0	0	0	0	0	0	0	0	0	0	0	0	0	0	0	0	0	0	0	0	0	0	0	0	0	0	0	0	0	0	0	0	0	0	mcrD	inhibits McrE 5-methylcytosine restriction system
b4345	0	0	0	0	0	0	0	0	0	0	0	0	0	0	0	0	0	0	0	0	0	0	0	0	0	0	0	0	0	0	0	0	0	0	0	0	0	0	0	0	0	0	0	mcrC	component of McrBC 5-methylcytosine restriction system, expands range of sequences restricted
b4346	0	0	0	0	0	0	0	0	0	0	0	0	0	0	0	0	0	0	0	0	0	0	0	0	0	0	0	0	0	0	0	0	0	0	0	0	0	0	0	0	0	0	0	mcrB	component of McrBC 5-methylcytosine restriction system
b4347	0	0	0	1	0	0	0	0	0	0	0	0	0	0	0	0	0	0	0	0	0	0	0	0	1	1	1	1	1	1	1	1	1	1	1	1	1	1	1	1	0	0	0	yjiW	orf, hypothetical protein
b4348	0	0	0	0	0	0	0	0	0	0	0	0	0	0	0	0	0	0	0	0	0	0	0	0	0	0	0	0	0	0	0	0	0	0	0	0	0	0	0	0	0	0	0	hsdS	specificity determinant for hsdM and hsdR
b4349	0	0	0	0	0	0	0	0	0	0	0	0	0	0	0	0	0	0	0	0	0	0	0	0	0	0	0	0	0	0	0	0	0	0	0	0	0	0	0	0	0	0	0	hsdM	host modification; DNA methylase M
b4350	0	0	0	0	0	0	0	0	0	0	0	0	0	0	0	0	0	0	0	0	0	0	0	0	0	0	0	0	0	0	0	0	0	0	0	0	0	0	0	0	0	0	0	hsdR	host restriction; endonuclease R
b4351	0	0	0	0	0	0	0	0	0	0	0	0	0	0	0	0	0	0	0	0	0	0	0	0	0	0	0	0	0	0	0	0	0	0	0	0	0	0	0	0	0	0	0	mrr	restriction of methylated adenine
b4352	1	1	1	1	1	0	1	0	0	1	1	1	1	1	1	1	1	0	1	1	1	1	0	1	1	0	1	1	0	1	0	1	0	1	1	1	1	1	1	1	0	1	1	yjiA	orf, hypothetical protein
b4353	1	1	1	1	1	1	0	1	1	1	1	1	1	1	1	1	1	1	1	1	1	1	1	1	0	1	0	1	1	0	1	1	1	1	1	1	1	1	1	1	0	1	1	yjiX	orf, hypothetical protein
b4355	1	0	1	1	1	1	1	1	1	1	1	1	1	1	1	1	1	1	1	1	1	1	1	1	1	1	1	1	1	1	1	1	1	1	1	1	1	1	1	1	1	1	1	tsr	methyl-accepting chemotaxis protein I, serine sensor receptor
b4357	0	1	1	1	1	0	0	0	0	0	0	0	1	1	1	1	1	1	1	1	1	1	1	1	0	1	1	1	1	1	1	1	1	1	1	1	1	1	1	1	1	0	0	yjjM	orf, hypothetical protein
b4365	1	1	1	1	1	0	1	1	1	1	1	1	1	1	1	1	1	1	1	1	1	1	1	1	1	1	1	1	1	1	1	0	1	1	1	1	1	1	1	1	1	0	1	yjjQ	putative regulator
b4367	1	1	1	1	1	1	1	1	1	1	1	1	1	1	1	1	1	1	1	1	1	1	1	1	0	0	1	1	0	0	0	1	1	1	1	1	1	1	1	1	1	1	1	fhuF	orf, hypothetical protein
b4372	1	1	1	1	1	1	1	1	1	1	1	1	1	1	1	1	1	1	1	1	1	1	0	1	1	1	1	1	1	1	1	1	1	1	1	1	1	1	1	1	1	1	1	holD	DNA polymerase III, psi subunit
b4374	1	1	1	1	1	1	1	1	1	1	1	1	1	1	1	1	1	1	1	1	1	1	0	1	1	1	1	1	1	1	1	1	1	1	1	1	1	1	1	1	1	1	1	yjjG	putative phosphatase
b4376	1	1	1	1	1	1	1	1	1	1	1	1	1	1	1	1	1	1	1	1	1	1	0	1	1	1	1	1	1	1	1	1	1	1	1	1	1	1	1	1	1	1	1	osmY	hyperosmotically inducible periplasmic protein
b4378m	1	1	1	1	1	1	1	1	1	1	1	1	1	1	1	1	1	1	1	1	1	1	0	1	1	1	1	1	1	1	1	1	0	1	1	1	1	1	1	0	1	1	1	yjjV	Mg-dependent DNase
b4385	0	0	0	0	0	0	0	0	0	0	0	0	0	0	0	0	0	0	0	0	1	0	0	0	0	0	0	0	0	0	0	0	0	0	0	0	0	0	0	0	0	0	0	yjjJ	orf, hypothetical protein
b4388	1	1	1	1	1	1	1	1	1	1	1	1	1	1	1	1	1	1	1	1	1	1	1	1	1	1	1	1	1	1	1	1	0	1	1	1	1	1	1	1	1	1	1	serB	3-phosphoserine phosphatase
b4393	1	1	1	1	1	1	0	1	1	1	1	1	1	1	1	1	1	1	1	1	1	1	1	1	1	1	1	1	1	1	1	1	1	1	1	1	1	1	1	1	1	1	0	trpR	regulator for trp operon and aroH; trp aporepressor

1:present
0:absent
