# Supplementary material for: The use of comparative genomic hybridization to characterize genome dynamics and diversity among the serotypes of Shigella
Source: BMC Genomics. 2006 Aug 29;7:218. doi: 10.1186/1471-2164-7-218 (PMC3225857; doi:10.1186/1471-2164-7-218)
Supplement: Additional File 2 — ORFs in each Shigella genome related to main clinical biochemical reaction. [file 1471-2164-7-218-S2.pdf]

ORFs in each Shigella genome related to main clinical biochemical reaction

Reaction	Gene	Product	D1	B13	SS	D10	D8	B3	B18	B8	B10	D5	B6	B1	B14	B4	F6	B2	D7	D12	D13	D11	D9	D4	D6	D3	B12	F5	F2a	Fy	Fx	F1a	F1b	F2b	F3	F4a	F4b	B17	B11	B16	B5	B7	B15	B9	D2
Indol	tnaA	trytophanase	0	1	0	1	1	0	1	1	1	1	1	1	0	1*	0	0	1	1	1	1	1	1	1	1	1	1	1	0	1	1	1	1	1	1	1	1	1	1	1	1	1	1	1
Ornithine	speC	ornithine decarboxylase	1	1	1	1	1	1	1	1	1	1	1	1	1	1*	1	1	1	1	1	1	1	1	1	1	1	1	1	1	1	1	1	1	1	1	1	1	1	1	1	1	1	1	1
Arginine	adiA	biodegradative arginine decarboxylase	1	1	1	1	1	1	1	1	1	1	1	1	1	1	1	1	1	1	1	1	1	1	1	1	1	1	1	1	1	1	1	1	1	1	1	1	1	1	1	1	1	1	1
	speA	biosynthetic arginine decarboxylase	1	1	1	1	1	1	1	1	1	1	1	1	1	1	1	1	1	1	1	1	1	1	1	1	1	1	1	1	1	1	1	1	1	1	1	1	1	1	1	1	1	1	1
Lactose	lacY	galactoside permease	1*	0	1*	0	0	0	0	0	0	0	0	0	0	0	0	0	0	0	0	0	0	0	0	0	0	0	0	0	0	0	0	0	0	0	0	0	0	0	0	0	1	1	0
	lacZ	beta-D-galactosidase	1	1	1	0	1	1	1	1	1	0	1	1	1	0	0	1	1	0	0	1	1	0	0	0	1	0	0	0	0	0	1	1	1	1	0	1	0	0	0	1	1	1	0
Lysine	cadA	lysine decarboxylase	1*	1	1*	0	1	0	0	0	0	0	0	0	0	0	0	0	0	0	0	0	0	0	0	0	0	1	0	0	0	0	0	0	0	0	0	0	1	0	0	0	0	0	0
	cadB	lysine/caverine transport protein	1*	1	1*	0	1	1	0	1	1	0	1	1	1	0	0	1	1	0	0	0	1	0	1	0	1	1	0	1	1	1	1	1	0	1	0	1	1	1	1	1	1	1	1
Citric acid	citF	citrate lyase alpha chain	0	1	1	1	1	1	1	1	1	1	1	1	1	1	1	1	1	1	1	1	1	1	1	1	1	1	1	1	1	1	1	1	1	1	1	0	1	1	1	1	1	1	1
	citE	citrate lyase beta chain (acyl lyase subunit)	0	1	1	1	1	1	1	1	1	1	1	1	1	1	1	1	0	0	0	0	0	0	0	0	1	1	1	1	1	1	1	1	1	1	1	1	1	1	1	1	1	1	1
	citD	citrate lyase acyl carrier protein (gamma chain)	0	1	1	1	1	1	1	1	1	1	1	1	1	1	1	1	1	1	1	1	1	1	1	1	1	1	1	1	1	1	1	1	1	1	1	1	1	1	1	1	1	1	1
	citC	citrate lyase synthetase	0	1	1*	1	1	1	1	1	1	1	1	1	1	1	1	1	0	1	1	1	1	1	0	1	1	1	1	1	1	1	1	1	1	1	1	1	1	1	1	1	1	1	1
	citA	sensory histidine kinase,regulation of citrate fermentation,sense citrate	0	1	1	1	1	1	1	1	1	1	1	1	1	1*	1	1	0	1	1	1	1	1	0	1	1	0	0	0	0	0	0	0	1	0	0	1	1	1	1	1	1	1	1
	citB	reponse regulator,regulation of citrate fermentation	0	1	1	1	1	1	1	1	1	1	1	1	1	1	1	1	0	1	1	1	1	1	0	1	1	1	1*	1	1	1	1	1	1	1	1	1	1	1	1	1	1	1	1
Acetate	aceB	malate synthase A	1*	1	1	1	1	1	1	1	1	1	1	1	1	1	1	1	1	1	1	1	1	1	1	1	1	1	1*	1	1	1	1	1	1	1	1	1	1	1	1	1	1	1	1
	aceA	isocitrate lyase	1	1	1	1	1	1	1	1	1	1	1	1	1	1*	1	1	1	1	1	1	1	1	1	1	1	1	1	1	1	1	1	1	1	1	1	1	1	1	1	1	1	1	1
	aceK	isocitrate dehydrogenase kinase/phosphatase	1	1	0	1	1	1	1	1	1	1	1	1	1	1	1	1	1	1	1	1	1	1	1	1	1	1	1*	1	1	1	1	1	1	1	1	1	1	0	1	1	1	1	1
D-mannitol	cmtA	PTS system, mannitol permease II, BC component	1	1	1*	1	1	1	1	1	1	1	1	1	1	1	1	1	1	1	1	1	1	1	1	1	0	0	0	0	0	0	0	0	0	1	1	1	1	1	1	1	1	1	0
	cmtB	PTS system, mannitol permease II, A component	1	1	1	1	1	1	1	1	1	1	1	1	1	1	1	1	1	1	1	1	1	1	1	1	0	0	0	0	0	0	0	0	0	1	1	1	1	1	1	1	1	1	1
	mtlA	PTS system, mannitol permease II, ABC components	0	1	1	1	1	1	1	1	1	1	1	1	0	1	0	1	1	0	1	1	1	1	1	1	1	1	1	1	0	1	1	1	1	1	0	1	1	1	1	1	1	1	1
	mtlD	mannitol-1-phosphate dehydrogenase	0	1	1	0	0	1	1	1	1	0	1	1	0	1	0	1	0	0	0	1	1	1	1	0	0	1	1	1	1	1	1	1	1	1	1	1	1	0	1	0	1	1	1
D-sorbitol	srlA	PTS system, glucitol/sorbitol-specific II, C component	1	1	1	1	1	1	1	1	1	1	1	1	1	1	1	1	1	1	1	1	1	1	1	1	1	1	1*	1	1	1	1	1	1	1	1	1	1	1	1	1	1	1	1
	srlE	PTS system, glucitol/sorbitol-specific II, B component	1*	1	1	1	1	1	1	1	1	1	1	1	1	1	1	1	1	1	1	1	1	1	1	1	1	1	1	1	1	1	1	1	1	1	1	1	1	1	1	1	1	1	1
	srlB	PTS system, glucitol/sorbitol-specific enzyme II, A component	1	1	1	1	1	1	1	1	1	1	1	1	1	1	1	1	1	1	1	1	1	1	1	1	1	1	1	1	1	1	1	1	1	1	1	1	1	1	1	1	1	1	1
	srlD	glucitol (sorbitol)-6-phosphate	1	1	1*	1	1	1	1	1	1	1	1	1	1	1	0	1	1	1	1	1	1	1	1	1	1	1	1	1	1	1	1	1	1	1	1	1	1	1	1	1	1	1	1
D-xylose	xylB	xylulokinase	0	0	1	1	1	1	1	1	1	1	1	1	1	1	1	1	1	1	1	1	1	0	0	1	1	1	1	1	1	1	1	1	1	1	1	1	1	1	1	1	1	1	1
	xylA	D-xylose isomerase	0	0	1	1	1	1	1	1	1	1	1	1	1	1	1	1	1	1	1	1	1	0	0	0	1	1	1*	1	1	1	1	1	1	1	1	1	1	1	1	1	1	1	1
	xylF	D-xylose transport system substrate-binding protein	1*	0	0	1	1	1	1	1	1	1	1	1	1	1	1	1	1	1	1	1	1	0	0	0	1	1	1	1	1	1	1	1	1	1	1	1	1	1	1	1	1	1	1
	xylG	D-xylose transport system ATP-binding protein	0	0	0	1	1	1	1	1	1	1	1	1	1	1	1	1	1	1	1	1	1	1	0	0	1	1	1	1	1	1	1	1	1	1	1	1	1	1	1	1	0	1	1
	xylH	D-xylose transport system permease protein	0	0	0	1	1	1	1	1	1	1	1	1	1	1	1	1	1	1	1	1	1	1	0	0	1	1	1	1	1	1	1	1	1	1	1	1	1	1	1	1	0	1	1
Dulcitol	gatD	galactitol-1-phosphate dehydrogenase	1	0	1	1	1	1	1	1	1	1	1	1	1	1	1	1	1	1	1	1	1	1	1	1	1	1	1	1	1	1	1	1	1	1	1	1	1	1	1	1	1	1	1
	gatC	PTS system galactitol-specific enzyme IIC	1*	1	1	1	1	1	1	1	1	1	1	1	1	1	1	1	1	1	1	1	1	1	1	1	1	1	1	1	1	1	1	1	1	1	1	1	1	1	1	1	1	1	1
	gatB	galactitol-specific enzyme IIB of phosphotransferase system	1	0	1	1	1	1	1	1	1	1	1	1	1	1	1	1	1	1	1	1	1	1	1	1	1	1	1	1	1	1	1	1	1	1	1	1	1	1	1	1	1	1	1
	gatA	galactitol-specific enzyme IIA of phosphotransferase system	1*	0	1*	1	1	1	1	1	1	1	1	1	1	1	1	1	1	1	1	1	1	1	1	1	1	1	1	1	1	1	1	1	1	1	1	1	1	1	1	1	1	1	1
	gatZ	putative tagatose 6-phosphate kinase 1	1*	0	1*	1	1	1	1	1	1	1	1	1	1	1*	1	1	1	1	1	1	1	1	1	1	1	1	1	1	1	1	1	1	1	1	1	1	1	1	1	1	1	1	1
	gatY	tagatose-bisphosphate aldolase 1	1	0	1	1	1	1	1	1	1	1	1	1	1	1	1	1	1	1	1	1	1	1	1	1	1	1	1	1	1	1	1	1	1	1	1	1	1	1	1	1	1	1	1
Rhamnose	rhaD	rhamnulose-phosphate aldolase	1	0	1	1	1	1	1	1	1	1	1	1	0	1	1	1	1	1	1	1	1	1	1	1	1	1	1	1	1	1	1	1	0	1	1	1	1	1	1	1	1	1	1
	rhaA	L-rhamnose isomerase	1*	0	1	1	1	0	0	0	0	0	0	0	1	1	1	1	1	1	1	1	1	1	1	1	1	1	1	1	1	1	1	1	1	1	1	1	1	1	1	1	1	1	1
	rhaB	rhamnulokinase	1	1	1	1	1	1	1	1	1	1	1	1	1	1	1	0	1	1	1	1	1	1	1	1	1	1	1	1	1	1	1	1	1	1	1	1	1	1	1	1	1	1	1
Glycerol	glpA	sn-glycerol-3-phosphate dehydrogenase (anaerobic), large subunit	1	1	1	1	1	0	1	1	1	1	1	1	1	1	1	1	1	1	1	1	1	1	1	1	1	1	1	1	1	1	1	1	1	1	1	1	1	1	1	1	1	1	1
	glpB	sn-glycerol-3-phosphate dehydrogenase (anaerobic), membrane anchor subunit	1	1	1	1	1	0	1	1	1	1	1	1	1	1	1	1	1	1	1	1	1	1	1	1	1	1	1	1	1	1	1	1	1	1	1	1	1	1	1	1	1	1	1
	glpC	sn-glycerol-3-phosphate dehydrogenase (anaerobic), K-small subunit	1	1	1	1	1	1	1	1	1	1	1	1	1	1	1	1	1	1	1	1	1	1	1	1	1	1	1	1	1	1	1	1	1	1	1	1	1	1	1	1	1	1	1
	glpD	sn-glycerol-3-phosphate dehydrogenase (aerobic)	1	1	1	1	1	1	0	0	0	1	1	1	0	1	1	1	1	0	1	1	1	1	1	1	0	1	1	1	1	1	1	1	1	1	1	0	1	0	0	0	0	0	1
	glpK	glycerol kinase	1	1	1	1	1	1	1	1	1	1	1	1	1	1	1	1	1	1	1	1	1	1	1	1	1	1	1	1	1	1	1	1	1	1	1	1	0	0	1	0	1	1	1
Melibiose	melA	alpha-galactosidase	1	0	1	1	1	1	1	1	1	1	1	1	1	1	1	1	1	0	0	0	0	0	0	0	1	1	1	1	1	1	1	1	1	1	1	1	0	1	1	1	1	1	1
	melB	melibiose permease II	1*	0	1*	1	1	0	0	0	0	0	0	0	0	0	0	0	0	0	0	0	0	0	0	0	1	1	1	1	1	1	1	1	1	1	1	1	1	1	1	1	1	1	1
Arabinose	araD	L-ribulose-5-phosphate 4-epimerase	1	1	1	1	1	1	1	1	1	1	1	1	1	1	1	1	1	1	1	1	1	1	1	1	1	1	1	1	1	1	1	1	1	1	1	1	1	1	1	1	1	1	1
	araA	L-arabinose isomerase	1	1	1	1	1	1	1	1	1	1	1	1	1	1	1	1	1	1	1	1	1	1	1	1	1	1	1*	1	1	1	1	1	1	1	1	1	1	1	1	1	1	1	1
	araB	L-ribulokinase	1	1	1	1	1	1	1	1	1	1	1	1	1	1	1	1	1	1	1	1	1	1	1	1	1	1	1	1	1	1	1	1	1	1	1	1	1	1	1	1	1	1	1
	araC	transcriptional regulator for ara operon	1*	1	1	1	1	1	1	1	1	1	1	1	1	1	1	1	1	1	1	1	1	1	1	1	1	1	1	1	1	1	1	1	1	1	1	1	1	1	1	1	1	1	1
	araH	ABC-type arabinose transport system, permease component	1	1	1	1	1	1	1	1	1	1	1	1	1	1	1	1	1	1	1	1	1	1	1	1	1	1	1	1	1	1	1	1	0	1	1	1	1	1	0	1	1	1	1
	araG	ATP-binding component of high-affinity L-arabinose transport system	1	1	1	1	1	1	1	1	1	1	1	1	1	1	1	1	1	1	1	1	1	1	1	1	1	1	1	1	1	1	1	1	0	1	1	1	1	1	0	1	1	1	1
	araF	L-arabinose-binding periplasmic protein	1	1	1	1	1	1	1	1	1	1	1	1	1	1*	1	1	1	1	1	1	1	1	1	1	1	1	1*	1	1	1	1	1	1	1	1	1	1	1	1	1	1	1	1

1:present 0:absent
* pseudogene
