# Supplementary material for: The use of comparative genomic hybridization to characterize genome dynamics and diversity among the serotypes of Shigella
Source: BMC Genomics. 2006 Aug 29;7:218. doi: 10.1186/1471-2164-7-218 (PMC3225857; doi:10.1186/1471-2164-7-218)
Supplement: Additional File 4 — Difference between F6 and other S. flexneri members. [file 1471-2164-7-218-S4.pdf]

ORFs that are present in F6 but absent in other S.flexneri strains

Gene	Synonym	Product
htrE	b0139	probable outer membrane porin protein involved in fimbrial assembly
ecpD	b0140	probable pilin chaperone similar to PapD
yadN	b0141	putative fimbrial-like protein
yaeI	b0164	orf, hypothetical protein
yagF	b0269	putative dehydratase
yagI	b0272	putative regulator
ykgJ	b0288	putative ferredoxin
yagW	b0290	putative receptor
yagY	b0292	orf, hypothetical protein
yagZ	b0293	orf, hypothetical protein
ykgK	b0294	putative regulator
eaeH	b0297	attaching and effacing protein, pathogenesis factor
ykgA	b0300	putative ARAC-type regulatory protein
b0499	b0499	orf, hypothetical protein
ybbD	b0500	orf, hypothetical protein
b0501	b0501	orf, hypothetical protein
b0502	b0502	orf, hypothetical protein
ybfP	b0689	putative pectinase
ybfG	b0690	orf, hypothetical protein
ybfH	b0691	orf, hypothetical protein
rhsC	b0700	rhsC protein in rhs element
b0703	b0703	orf, hypothetical protein
putA	b1014	proline dehydrogenase, P5C dehydrogenase
putP	b1015	major sodium/proline symporter
msyB	b1051	acidic protein suppresses mutants lacking function of protein export
ycfA	b1156	orf, hypothetical protein
b1157	b1157	putative tail fiber protein
b1169	b1169	putative ATP-binding component of a transport system
ychG	b1239	orf, hypothetical protein
yciQ	b1268	orf, hypothetical protein
ydaH	b1336	putative pump protein (transport)
ydaJ	b1338	putative aminohydrolase (EC 3.5.1.14)
ydaK	b1339	putative transcriptional regulator LYSR-type
recE	b1350	exonuclease VIII, ds DNA exonuclease, 5' --> 3' specific
b1365	b1365	orf, hypothetical protein
b1391	b1391	orf, hypothetical protein
b1433	b1433	putative membrane transport protein
ydcN	b1434	orf, hypothetical protein
ydcP	b1435	putative collagenase
-	b1436	orf, hypothetical protein
-	b1438	orf, hypothetical protein
b1451	b1451	putative outer membrane receptor for iron transport
b1452	b1452	putative receptor
ansP	b1453	L-asparagine permease

ORFs that are absent in F6 but present in other S.flexneri strains

Gene	Synonym	Product
yaaV	b0033a	Uncharacterized conserved protein
caiF	b0034	transcriptional regulator of cai operon
caiD	b0036	carnitine racemase
caiC	b0037	probable crotonobetaine/carnitine-CoA ligase
caiB	b0038	l-carnitine dehydratase
caiA	b0039	probable carnitine operon oxidoreductase
caiT	b0040	probable carnitine transporter
fixX	b0044	putative ferredoxin
fhuA	b0150	outer membrane protein receptor for ferrichrome, colicin M, and phages T1, T5, and phi80
yafN	b0232	orf, hypothetical protein
yafO	b0233	orf, hypothetical protein
ylbB	b0516	putative hydantoin utilization protein
nmpC	b0553	outer membrane porin protein; locus of qsr prophage
ybcH	b0567	orf, hypothetical protein
nfrA	b0568	bacteriophage N4 receptor, outer membrane protein
nfrB	b0569	bacteriophage N4 receptor, outer membrane protein
ybcZ	b0570	putative 2-component sensor protein
ylcA	b0571	putative 2-component transcriptional regulator
ylcB	b0572	putative resistance protein
ybdO	b0603	putative transcriptional regulator LYSR-type
ybeT	b0647	orf, hypothetical protein
ybeU	b0648	putative tRNA ligase
hrsA	b0731	protein modification enzyme, induction of ompC
ybhH	b0769	orf, hypothetical protein
ybhI	b0770	putative membrane pump protein
ybiI	b0803	orf, hypothetical protein
b0805	b0805	putative outer membrane receptor for iron transport
ybiM	b0806	orf, hypothetical protein
ycbS	b0940	putative outer membrane protein
b0941	b0941	homolog of Salmonella FimH protein
b0942	b0942	putative fimbrial-like protein
b0943	b0943	putative fimbrial-like protein
agp	b1002	periplasmic glucose-1-phosphatase
wrbA	b1004	trp repressor binding protein; affects association of trp repressor and operator
ycdF	b1005	orf, hypothetical protein
ycdG	b1006	putative transport protein
b1007	b1007	orf, hypothetical protein
b1009	b1009	putative acetyltransferase
b1010	b1010	orf, hypothetical protein
b1011	b1011	putative synthetase
b1012	b1012	orf, hypothetical protein
yciG	b1259	orf, hypothetical protein
ycjU	b1317	putative beta-phosphoglucomutase
ycjV	b1318	putative ATP-binding component of a transport system

b1486	b1486	putative transport system permease protein	ompG	b1319	outer membrane protein
b1487	b1487	putative hemin-binding lipoprotein	ycjX	b1321	putative EC 2.1 enzymes
ydeH	b1535	orf, hypothetical protein	narZ	b1468	cryptic nitrate reductase 2, alpha subunit
dicB	b1575	inhibition of cell division	narU	b1469	nitrite extrusion protein 2
yoaG	b1796	orf, hypothetical protein	b1470	b1470	orf, hypothetical protein
yeaR	b1797	orf, hypothetical protein	b1471	b1471	putative glycoportein
yeaS	b1798	orf, hypothetical protein	ydeY	b1514	putative transport system permease protein
yeaT	b1799	putative transcriptional regulator LYSR-type	b1516	b1516	putative LACI-type transcriptional regulator
yeaU	b1800	putative tartrate dehydrogenase	yneB	b1517	orf, hypothetical protein
yeaV	b1801	putative transport protein	b1518	b1518	orf, hypothetical protein
yodB	b1974	putative cytochrome	b1519	b1519	putative enzyme
b1978	b1978	putative factor	b1520	b1520	orf, hypothetical protein
b2246	b2246	putative transport protein	uxaB	b1521	altronate oxidoreductase
b2247	b2247	putative racemase	b1522	b1522	orf, hypothetical protein
b2248	b2248	putative regulator	b1523	b1523	orf, hypothetical protein
b2353	b2353	orf, hypothetical protein	yneH	b1524	putative glutaminase
b2354	b2354	orf, hypothetical protein	b1525	b1525	putative aldehyde dehydrogenase
b2430	b2430	putative beta-lactamase	b1527	b1527	orf, hypothetical protein
b2459	b2459	orf, hypothetical protein	ydeA	b1528	putative resistance / regulatory protein
b2520	b2520	orf, hypothetical protein	ydeB	b1529	orf, hypothetical protein
chpA	b2782	probable growth inhibitor, PemK-like, autoregulated	marA	b1531	multiple antibiotic resistance; transcriptional activator of defense systems
chpR	b2783	suppressor of inhibitory function of ChpA, PemI-like, autoregulated	marB	b1532	multiple antibiotic resistance protein
ygeV	b2869	putative transcriptional regulator	ydeD	b1533	orf, hypothetical protein
ygeY	b2872	putative deacetylase	ydeF	b1534	putative transport protein
b2873	b2873	orf, hypothetical protein	rem	b1561	orf, hypothetical protein
yqeA	b2874	putative kinase	relE	b1563	orf, hypothetical protein
b2875	b2875	putative synthases	relB	b1564	negative regulator of translation
ygfJ	b2877	orf, hypothetical protein	b1565	b1565	orf, hypothetical protein
yghD	b2968	putative secretion pathway protein	rspA	b1581	starvation sensing protein
yghE	b2969	putative general secretion pathway for protein export (GSP)	b1583	b1583	orf, hypothetical protein
b2970	b2970	putative general secretion pathway for protein export (GSP)	b1690	b1690	putative transport system permease protein
envR	b3264	putative transcriptional regulator	b1730	b1730	orf, hypothetical protein
yrhB	b3446	orf, hypothetical protein	b1731	b1731	orf, hypothetical protein
rhsB	b3482	rhsB protein in rhs element	katE	b1732	catalase; hydroperoxidase HPII(III)
rhsA	b3593	rhsA protein in rhs element	ydjC	b1733	orf, hypothetical protein
yibJ	b3595	orf, hypothetical protein	celF	b1734	phospho-beta-glucosidase; cryptic
yibG	b3596	orf, hypothetical protein	b1762	b1762	orf, hypothetical protein
yifN	b3777m	Uncharacterized conserved protein	ydjE	b1769	putative transport protein
yiiG	b3896	orf, hypothetical protein	b1770	b1770	putative DEOR-type transcriptional regulator
htrC	b3989	heat shock protein htrC	b1771	b1771	orf, hypothetical protein
yjfI	b4181	orf, hypothetical protein	b1772	b1772	putative kinase
yjfJ	b4182	putative alpha helical protein	b1773	b1773	putative aldolase
yjfK	b4183	orf, hypothetical protein	ydjJ	b1774	putative oxidoreductase
yjiJ	b4332	putative transport protein	b1775	b1775	putative transport protein
yjiK	b4333	orf, hypothetical protein	b1776	b1776	putative oxidoreductase
yjiL	b4334	putative enzyme	amn	b1982	AMP nucleosidase

yjiM	b4335	orf, hypothetical protein	nac	b1988	nitrogen assimilation control protein
yjiN	b4336	orf, hypothetical protein	wzzB	b2027	regulator of length of O-antigen component of lipopolysaccharide chains
yjiO	b4337	putative transport protein	wcaJ	b2047	putative colanic acid biosynthsis UDP-glucose lipid carrier transferase
yjiQ	b4339	orf, hypothetical protein	yohM	b2106	orf, hypothetical protein
yjiS	b4341	orf, hypothetical protein	b2107	b2107	orf, hypothetical protein
yjiT	b4342	orf, hypothetical protein	yehA	b2108	putative type-1 fimbrial protein
SBO0564	SBO0564	orf, hypothetical protein	yehB	b2109	putative outer membrane protein
SBO4343	SBO4343	orf, conserved hypothetical protein	yehC	b2110	putative chaperone
SBO4397	SBO4397	putative protein encoded within IS	yehD	b2111	putative fimbrial-like protein
SBO0748	SBO0748	repressor protein	yehE	b2112	orf, hypothetical protein
SBO0749	SBO0749	orf, conserved hypothetical protein	molR_1	b2115	molybdate metabolism regulator, first fragment
SBO0752	SBO0752	possible endonuclease	molR_2	b2116	molybdate metabolism regulator, second fragment 2
SBO0753	SBO0753	putative damage-inducible protein	yeiL	b2163	putative transcriptional regulator
SBO0754	SBO0754	putative phage-related protein	b2229	b2229	orf, hypothetical protein
SBO0755	SBO0755	putative phage-related protein	b2350	b2350	orf, hypothetical protein
SBO0757	SBO0757	probable capsid portal protein (fragment)	b2420	b2420	orf, hypothetical protein
SBO0767	SBO0767	putative regulatory protein	b2504	b2504	orf, hypothetical protein
SBO0768	SBO0768	putative phage tail protein	pheA	b2599	chorismate mutase-P and prephenate dehydratase
SBO0775	SBO0775	probable major tail tube protein	smpA	b2617	small membrane protein A
SBO0776	SBO0776	putative phage tail protein	ygaE	b2664	putative transcriptional regulator
SBO0777	SBO0777	putative bacteriophage tail protein	stpA	b2669	DNA-binding protein; H-NS-like protein; chaperone activity; RNA splicing?
SBO0779	SBO0779	putative bacteriophage late gene regulator	nrdE	b2675	ribonucleoside-diphosphate reductase 2, alpha subunit
SBO0780	SBO0780	putative bacteriophage late gene regulator	srlD	b2705	glucitol (sorbitol)-6-phosphate dehydrogenase
SBO0856	SBO0856	putative nucleotide sugar epimerase	hypF	b2712	transcriptional regulatory protein
wzy	SBO0860	O-antigen polymerase	pphB	b2734	protein phosphatase 2
SBO0907	SBO0907	orf, hypothetical protein	ygbO	b2745	putative hydrogenase subunit
SBO0959	SBO0959	putative tail protein	ygcM	b2765	putative 6-pyruvoyl tetrahydrobiopterin synthase
SBO0971	SBO0971	unknown protein encoded by prophage	ygcY	b2788	putative glucarate dehydratase
SBO0973	SBO0973	putative transcriptional regulator	fucK	b2803	L-fuculokinase
SBO1136	SBO1136	orf, hypothetical protein	b2809	b2809	orf, hypothetical protein
SBO1137	SBO1137	orf, hypothetical protein	ygdK	b2811	orf, hypothetical protein
SBO1138	SBO1138	orf, conserved hypothetical protein	mltA	b2813	membrane-bound lytic murein transglycosylase A
SBO1139	SBO1139	transcriptional regulatory protein	kduI	b2843	homolog of pectin degrading enzyme 5-keto 4-deoxyuronate isomerase
SBO1140	SBO1140	putative integrase	ygfF	b2902	putative oxidoreductase
SBO1171	SBO1171	putative alpha-mannosidase	visC	b2906	orf, hypothetical protein
SBO1195	SBO1195	putative tail fiber protein	ygfE	b2910	orf, hypothetical protein
SBO1222	SBO1222	putative integrase	ygfA	b2912	putative ligase
SBO1384	SBO1384	orf, hypothetical protein	yqfE	b2915	orf, hypothetical protein
SBO1385	SBO1385	putative bacteriophage late gene regulator	epd	b2927	D-erythrose 4-phosphate dehydrogenase
SBO1386	SBO1386	putative tail sheath protein	yggD	b2929	putative transcriptional regulator
SBO1387	SBO1387	putative tail tube protein	b2931	b2931	putative oxidoreductase
SBO1396	SBO1396	probable phage baseplate assembly protein	glcB	b2976	malate synthase G
SBO1398	SBO1398	putative phage baseplate assembly protein	glcF	b2978	glycolate oxidase iron-sulfur subunit
SBO1399	SBO1399	putative phage tail completion protein	glcD	b2979	glycolate oxidase subunit D
SBO1400	SBO1400	putative phage tail protein	glcC	b2980	transcriptional activator for glc operon
SBO1401	SBO1401	orf, hypothetical protein	b2981	b2981	orf, hypothetical protein

SBO1402	SBO1402	putative endolysin	pitB	b2987	low-affinity phosphate transport
SBO1403	SBO1403	probable phage tail protein	b3027	b3027	orf, hypothetical protein
SBO1404	SBO1404	putative capsid completion protein	ugpB	b3453	sn-glycerol 3-phosphate transport system; periplasmic binding protein
SBO1405	SBO1405	putative phage terminase	t150	b3558	IS150 putative transposase
SBO1406	SBO1406	major capsid protein	yibH	b3597	putative membrane protein
SBO1409	SBO1409	probable capsid portal protein	mtlD	b3600	mannitol-1-phosphate dehydrogenase
SBO1410	SBO1410	putative phage-related protein	yibL	b3602	orf, hypothetical protein
SBO1411	SBO1411	putative stability/partitioning protein	htrL	b3618	involved in lipopolysaccharide biosynthesis
SBO1414	SBO1414	orf, conserved hypothetical protein	yidL	b3680	putative ARAC-type regulatory protein
SBO1923	SBO1923	orf, conserved hypothetical protein	dgoT	b3691	D-galactonate transport
SBO1928	SBO1928	orf, conserved hypothetical protein	dgoA	b3692	2-oxo-3-deoxygalactonate 6-phosphate aldolase and galactonate dehydratase
SBO1938	SBO1938	putative integrase	yidX	b3696	putative replicase EC 2.7.-
SBO3440	SBO3440	putative transposase	bglB	b3721	phospho-beta-glucosidase B; cryptic
SBO2037	SBO2037	orf, conserved hypothetical protein	bglF	b3722	PTS system beta-glucosides, enzyme II, cryptic
SBO2123	SBO2123	orf, conserved hypothetical protein	yigF	b3817	orf, hypothetical protein
SBO2125	SBO2125	major capsid protein	yigG	b3818	orf, hypothetical protein
SBO2133	SBO2133	orf, hypothetical protein	yihL	b3872	putative transcriptional regulator
SBO2134	SBO2134	putative CP4-57-type integrase	yihS	b3880	orf, hypothetical protein
SBO2369	SBO2369	orf, conserved hypothetical protein	yihT	b3881	putative aldolase
SBO2700	SBO2700	putative phosphoglycerate dehydrogenase	yihU	b3882	putative dehydrogenase
SBO2704	SBO2704	cystathionine beta-lyase; maltose regulon modulator	yihV	b3883	putative kinase
SBO2706	SBO2706	orf, conserved hypothetical protein	yihW	b3884	putative DEOR-type transcriptional regulator
ygcB	SBO2759	putative helicase	arp	b4017	regulator of acetyl CoA synthetase
SBO2760	SBO2760	putative cytoplasmic protein	xylE	b4031	xylose-proton symport
SBO2761	SBO2761	orf, conserved hypothetical protein	malG	b4032	part of maltose permease, inner membrane
SBO2764	SBO2764	orf, conserved hypothetical protein	malF	b4033	part of maltose permease, periplasmic
ygbF	SBO2766	orf, conserved hypothetical protein	malE	b4034	periplasmic maltose-binding protein; substrate recognition for transport and chemotaxis
gspD	SBO3012	putative type II secretion protein	lamB	b4036	phage lambda receptor protein; maltose high-affinity receptor
gspE	SBO3013	putative type II secretion protein	malM	b4037	periplasmic protein of mal regulon
gspF	SBO3014	putative type II secretion protein	melB	b4120	melibiose permease II
gspG	SBO3015	putative type II secretion protein	gntP	b4321	gluconate transport system permease 3
gspH	SBO3016	putative type II secretion protein	safA	SF0202	putative cytoplasmic protein
gspI	SBO3017	putative type II secretion protein	safB	SF0203	periplasmic chaperone of fimbral assembly machinery
gspJ	SBO3018	putative type II secretion protein	safC	SF0204	outer membrane usher protein
gspK	SBO3019	putative type II secretion protein	sat1	SF0206	putative cytoplasmic protein
SBO4079	SBO4079	putative transposase	sat2	SF0207	putative cytoplasmic protein
SBO3125	SBO3125	putative ribose ABC transporter	SF0208	SF0208	putative cytoplasmic protein
SBO3127	SBO3127	Ribose transport system permease protein rbsC	SF0215	SF0215	putative terminase large subunit
SBO3129	SBO3129	possible carbohydrate kinase	SF0265	SF0265	putative Rhs-family protein
SBO3130	SBO3130	orf, conserved hypothetical protein	SF0592	SF0592	orf, hypothetical protein
SBO3247	SBO3247	orf, conserved hypothetical protein	SF0593	SF0593	putative Rhs-family protein (fragment)
SBO3248	SBO3248	putative tagatose-bisphosphate aldolase	SF0669	SF0669	putative bacteriophage protein
SBO3250	SBO3250	possible carbohydrate kinase	SF0675	SF0675	putative bacteriophage protein
SBO4398	SBO4398	putative protein encoded within IS	SF0676	SF0676	orf, conserved hypothetical protein
SBO3417	SBO3417	orf, conserved hypothetical protein	SF0679	SF0679	putative bacteriophage protein
SBO3418	SBO3418	orf, conserved hypothetical protein	SF1131	SF1131	putative integrase of prophage CP-933C

SBO3419	SBO3419	orf, conserved hypothetical protein
SBO3421	SBO3421	hypothetical membrane protein
lpfA	SBO3546	putative major fimbrial subunit
waaW	SBO3628	UDP-galactose:(galactosyl) LPS alpha1,2-galactosyltransferase
waaY	SBO3629	lipopolysaccharide core biosynthesis
waaJ	SBO3630	lipopolysaccharide 1,2-glucosyltransferase
waaI	SBO3631	UDP-D-galactose:(glucosyl)lipopolysaccharide- alpha-1,3-D-galactosyltr
SBO3649	SBO3649	hypothetical bacteriophage protein
SBO3650	SBO3650	putative anti-repressor protein
SBO3735	SBO3735	orf, hypothetical protein
SBO3736	SBO3736	orf, hypothetical protein
SBO3737	SBO3737	orf, hypothetical protein
SBO3738	SBO3738	orf, hypothetical protein
yihW	SBO3885	putative DEOR-type transcriptional regulator
yihV	SBO3886	putative kinase
rspB	SBO3891	putative dehydrogenase
SBO3897	SBO3897	putative transport protein, shikimate
SBO3898	SBO3898	orf, conserved hypothetical protein
SBO4160	SBO4160	putative type I restriction-modification system specificity subunit
SBO4163	SBO4163	putative phage-related protein
SBO4165	SBO4165	putative DNA primase
SBO4166	SBO4166	putative protein encoded in prophage
SBO4171	SBO4171	phage polarity suppression protein
SBO4172	SBO4172	orf, hypothetical protein
SBO4243	SBO4243	orf, conserved hypothetical protein
SBO4247	SBO4247	transport protein of hexuronates
SBO4323	SBO4323	orf, hypothetical protein
SBO4353	SBO4353	orf, conserved hypothetical protein
SBO4355	SBO4355	orf, hypothetical protein
SBO4393	SBO4393	putative penicillin G acylase
SBO4399	SBO4399	orf, conserved hypothetical protein
SSO0240	SSO0240	orf, conserved hypothetical protein
SSO0323	SSO0323	putative dehydrogenase
cjrA	SSO2662	putative iron-regulated protein
cjrB	SSO2663	putative membrane protein, TonB-like, colicin Js sensitive
senB	SSO2665	putative enterotoxin

SF1140	SF1140	putative head portal protein
SF1141	SF1141	putative head-tail adaptor
SF1145	SF1145	hypothetical bacteriophage protein
SF1146	SF1146	putative terminase of prophage CP-933C
SF1180	SF1180	invasion plasmid antigen fragment
SF1355	SF1355	putative bacteriophage protein
SF1544	SF1544	antitermination protein, homolog of cryptic prophage
SF1756	SF1756	orf, conserved hypothetical protein
SF1849	SF1849	putative integrase
SF1929	SF1929	orf, hypothetical protein
SF2039	SF2039	putative Q antiterminator of prophage
SF2047	SF2047	putative integrase
SF2092	SF2092	orf, conserved hypothetical protein
SF2093	SF2093	orf, conserved hypothetical protein
SF2094	SF2094	orf, conserved hypothetical protein
rfbJ	SF2095	glycosyl transferase
rfbI	SF2096	glycosyl translocase
rfbG	SF2098	dTDP-rhamnosyl transferase
rfbF	SF2099	dTDP-rhamnosyl transferase
rfbE	SF2100	polysaccharide biosynthesis protein
SF2408	SF2408	orf, conserved hypothetical protein
SF2409	SF2409	similar to fimbrial protein
SF2495	SF2495	orf, conserved hypothetical protein
SF2496	SF2496	orf, conserved hypothetical protein
SF2497	SF2497	orf, conserved hypothetical protein
SF2498	SF2498	orf, conserved hypothetical protein
SF2499	SF2499	putative amino acid antiporter
SF2500	SF2500	similar to glutamate racemase
SF2619	SF2619	putative bacteriophage protein
SF2622	SF2622	orf, conserved hypothetical protein
SF2758	SF2758	putative phage transposase
SF2759	SF2759	putative DNA-binding protein
SF2924	SF2924	putative serine protease
SF2947	SF2947	orf, conserved hypothetical protein
SF2948	SF2948	orf, conserved hypothetical protein
SF2949	SF2949	orf, conserved hypothetical protein
SF3004	SF3004	orf, hypothetical protein
SF3434	SF3434	PemK protein
waaI	SF3662	lipid A-core, surface polymer ligase
waaD	SF3663	lipopolysaccharide 1,2-N- acetylglucosaminetransferase
waaJ	SF3664	putative UDP-glucose:(galactosyl) LPS alpha1,2-glucosyltransferase
waaY	SF3665	putative LPS biosynthesis protein
waaL	SF3666	UDP-D-galactose:(glucosyl)lipopolysaccharide- alpha-1,3-D-galactosyltransferase
SF3698	SF3698	putative integrase
shiC	SF3704	orf, conserved hypothetical protein
SF3707	SF3707	orf, conserved hypothetical protein

	SF3710	SF3710	orf, conserved hypothetical protein
	lpfC	SF3723	putative long polar fimbriae
	lpfA	SF3807	putative major fimbrial subunit
	SF3884	SF3884	orf, partial conserved hypothetical protein
	SF4376	SF4376	orf, conserved hypothetical protein
	hpaA	SF4377	putative 4-hydroxyphenylacetate 3-monooxygenase operon regulatory protein
	hpaX	SF4378	putative 4-hydroxyphenylacetate permease
