# Supplementary material for: The use of comparative genomic hybridization to characterize genome dynamics and diversity among the serotypes of Shigella
Source: BMC Genomics. 2006 Aug 29;7:218. doi: 10.1186/1471-2164-7-218 (PMC3225857; doi:10.1186/1471-2164-7-218)
Supplement: Additional File 5 — Strains used in previous study. [file 1471-2164-7-218-S5.pdf]

Bacterial strains used from previous study

Strain	Abbreviation	Serotype	Source*
E. coli EHEC O157 Sakai	O157 Sakai	O157:H7	RIMD
E. coli EHEC O26	EHEC	O26	RIMD
E. coli EPEC B171	EPEC-1	O111:NM	RIMD
E. coli EPEC E2348/69	EPEC-2	O127:H6	RIMD
E. coli EPEC 5513-51	EPEC-3	O55:H6	RIMD
E. coli EPEC 4394-57	EPEC-4	O114:NM	RIMD
E. coli EPEC 1929-55	EPEC-5	O126:NM	RIMD
E. coli EPEC 1157-54	EPEC-6	O119:H6	RIMD
E. coli EPEC 1181-83	EPEC-7	O142:H6	RIMD
E. coli ETEC H10407	ETEC-1	O78:H11	RIMD
E. coli ETEC 31-10	ETEC-2	O25:H-	RIMD
E. coli EIEC 931-78	EIEC-1	O124	RIMD
E. coli EIEC 14185-83HU	EIEC-2	O28ac	RIMD
E. coli EIEC 127-82FAV	EIEC-3	O29	RIMD
E. coli EIEC 282-83FAV	EIEC-4	O136	RIMD
E. coli UPEC Z42	UPEC-1	O2:H6	RIMD
E. coli UPEC C72	UPEC-2	O46:H52	RIMD
E. coli UPEC P17	UPEC-3	O129:NT	RIMD
E. coli REPEC REPEC-1	REPEC	O103:K-:H6	RIMD
S. flexneri 2a YSH6000	S.f		IMS
S. boydii IID627	S.b		IMS
S. sonnei phaseI IID969	S.s		IMS

*NAIST, Nara Institute of Science and Technology, Nara, Japan; RIMD, Research Institute for Microbial Diseases, Osaka University, Osaka, Japan; IMS,Laboratory of Culture Collection, Institute of Medical Science, University of Tokyo, Tokyo, Japan.
